# SARS-CoV-2 ORF3a blocks lysosomal cholesterol egress by disrupting VPS39-regulated NPC2 trafficking and BMP metabolism

**DOI:** 10.1016/j.celrep.2026.117544

**Published:** 2026-06-13

**Authors:** Baley A. Goodson, Valeria Montenegro Vazquez, Aliza Doyle, Oralia M. Kolaczkowski, Rui Liu, Jingyue Jia, Morié Ishida, Chunyan Ye, Alison M. Kell, Steven B. Bradfute, Monica Rosas Lemus, Hu Wang, Xianlin Han, Jing Pu

**Affiliations:** 1 Department of Molecular Genetics and Microbiology, University of New Mexico Health Sciences Center, Albuquerque, NM, USA; 2 Department of Pharmaceutical Sciences, College of Pharmacy, University of New Mexico Health Sciences Center, Albuquerque, NM, USA; 3 Department of Internal Medicine, University of New Mexico Health Sciences Center, Albuquerque, NM, USA; 4 Neurosciences and Cellular and Structural Biology Division, Eunice Kennedy Shriver National Institute of Child Health and Human Development, National Institutes of Health, Bethesda, MD, USA; 5 Department of Medicine, UT Health San Antonio Long School of Medicine, San Antonio, TX, USA; 6 Lead contact

## Abstract

Cholesterol homeostasis depends on lysosomes liberating cholesterol from degraded lipids. We show that SARS-CoV-2 blocks lysosomal cholesterol egress through the viral protein ORF3a. ORF3a binds the HOPS subunit VPS39 via the W193 and Y184 residues. Disrupting this interface restores cholesterol trafficking. Mechanistically, the ORF3a-VPS39 interaction exerts dual effects. First, it traps the retromer complex on endolysosomes, preventing endosome-to-Golgi recycling and mislocalizing the cholesterol transporter NPC2. Retromer deletion reproduced these defects, whereas the ORF3a W193A mutant restored retromer trafficking. Second, ORF3a-VPS39 interaction reduces bis(monoacylglycerol)phosphates (BMPs), lysosomal lipids required for cholesterol egress, by disrupting the transfer of their precursor, phosphatidylglycerols, from mitochondria. Lipidomics revealed increased mitochondrial and decreased lysosomal phosphatidylglycerol metabolites. Disturbing autophagy or mitochondrion-derived vesicles did not alter BMP levels, whereas ORF3a reduced mitochondrion-lysosome membrane contacts. These findings identify dual functions of VPS39—regulating retromer trafficking and BMP biosynthesis—and also reveal how ORF3a blocks lysosomal cholesterol egress.

## In brief

Goodson et al. show that SARS-CoV-2 ORF3a hijacks VPS39 to disrupt lysosomal cholesterol egress through coordinated defects in retromer-dependent NPC2 trafficking and mitochondrion-lysosome-contact-dependent BMP production, uncovering dual pathways controlling lysosomal lipid homeostasis.

## Graphical Abstract

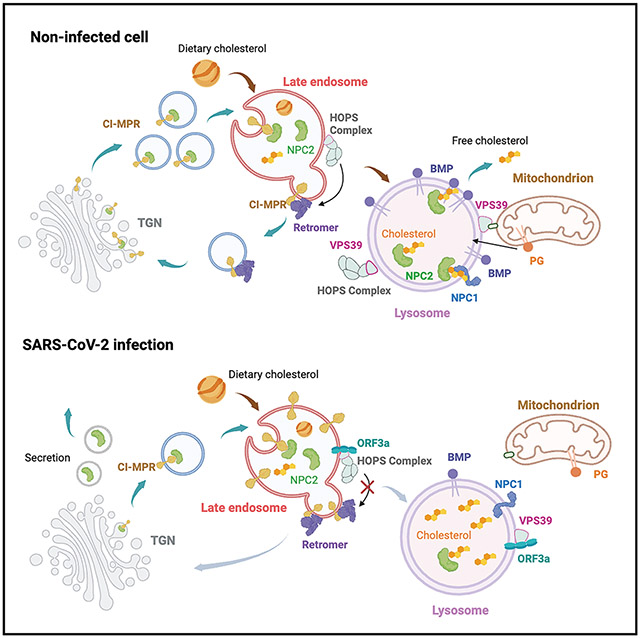

## INTRODUCTION

Cholesterol plays a vital role in maintaining cellular structure and functions. Thus, cells tightly regulate cholesterol levels and distribution through multiple pathways. Cholesterol pathways are not only disturbed in metabolic disorders but also hijacked in infectious diseases. COVID-19, caused by the SARS-CoV-2 virus, has been associated with significant disruptions in cholesterol metabolism.^1-5^ In cases of long COVID, an increased risk of dyslipidemia and cardiovascular diseases is marked by elevated low-density lipoprotein (LDL) cholesterol and reduced high-density lipoprotein (HDL) cholesterol levels.^4,5^

Lysosomes are central to cholesterol regulation by degrading internalized lipoproteins and cellular membrane delivered through endocytosis and autophagy, respectively, releasing free cholesterol for redistribution.^6^ The released cholesterol is then distributed to other cellular compartments to support structural and metabolic demands.^7,8^ For example, the liberated cholesterol is transported to the plasma membrane to maintain local membrane fluidity and stability.^9^ SARS-CoV-2 exploits plasma membrane cholesterol to enhance infectivity,^10^ as evidenced by a reduced binding between the viral spike protein and its host receptor after plasma membrane cholesterol depletion.^11^ Therefore, the lysosomal cholesterol transport is central to cellular cholesterol homeostasis and may also play a role in SARS-CoV-2 infection.

Previous studies have shown that SARS-CoV-2 disrupts lysosomal pH,^12^ membrane integrity,^13^ small GTPases,^14^ autophagic flux,^15^ and exocytosis,^15,16^ primarily through the viral protein ORF3a. ORF3a is a lysosome-localized transmembrane protein that interacts with the host homotypic fusion and protein sorting (HOPS) complex subunit VPS39,^15,17,18^ thereby inhibiting HOPS-mediated lysosomal fusion events.^15,18^ Despite extensive reports of lysosomal dysfunction during SARS-CoV-2 infection and ORF3a expression, whether and how the virus alters lysosomal cholesterol distribution remains unknown.

The first step in lysosomal cholesterol distribution is cholesterol egress. This process is primarily mediated by the lysosomal transmembrane cholesterol transporter Niemann-Pick C1 (NPC1) and the lumenal cholesterol-binding protein Niemann-Pick C2 (NPC2), which act cooperatively to transfer cholesterol out of lysosomes.^8^ The proper delivery of newly synthesized lysosome transmembrane proteins depend on sorting signals and clathrin coats,^19^ and the lysosome lumenal proteins by sorting receptors, such as the mannose-6-phosphate receptors (M6PRs).^19^ These receptors deliver lysosome lumenal proteins from the trans-Golgi network (TGN) to endosomes and are subsequently recycled back to the TGN. This recycling requires retromer complexes at endosomes and lysosomes^20-23^ and Golgi-associated retrograde protein (GARP) complex at the TGN.^24^ Disruption of these transport pathways by impairing M6PR or GARP functions compromises the delivery of newly synthesized proteins to lysosomes and results in accumulation of cholesterol.^22,25,26^ Our recent work demonstrated that the HOPS complex is required for lysosomal cholesterol egress by maintaining cation-independent mannose 6-phosphate receptor (CI-MPR) trafficking,^22^ yet the molecular mechanism by which HOPS regulates this process remains unclear.

In addition to protein-mediated cholesterol transport, the phospholipid bis(monoacylglycero)phosphates (BMPs, also called lysobisphosphatidic acids or LBPAs), play an important role in lysosomal cholesterol egress. BMPs are highly enriched in the late endosome and lysosome membrane, where they contribute to lysosome sorting and degradation functions^27-30^ and viral fusion during infection.^31-34^ Beyond these functions, BMPs directly interact with NPC2 to promote lysosomal cholesterol egress.^35,36^ BMPs are synthesized from mitochondrial precursors, phosphatidylglycerols (PGs),^37-40^ implicating mitochondria-lysosome communication in BMP biogenesis. However, the mechanism underlying PG transfer and the coordination between lysosomal and mitochondrial lipid metabolism are poorly understood.

In this study, we uncover that SARS-CoV-2 infection drives cholesterol sequestration in lysosomes through dual disruption of VPS39-dependent pathways. The viral protein ORF3a binds VPS39 to simultaneously trap the retromer complex on endolysosomes and reduce mitochondrion-lysosome membrane contact sites (MCSs), impairing both CI-MPR recycling and PG-derived BMP synthesis. By linking VPS39 to the regulation of retromer trafficking and mitochondrial lipid transfer, our work reveals an unrecognized mechanism by which SARS-CoV-2 ORF3a reprograms lysosomal lipid metabolism, advancing our understanding of how viral infection rewires host cholesterol homeostasis.

## RESULTS

### Increased lysosomal cholesterol in SARS-CoV-2-infected cells

**Figure 1.**
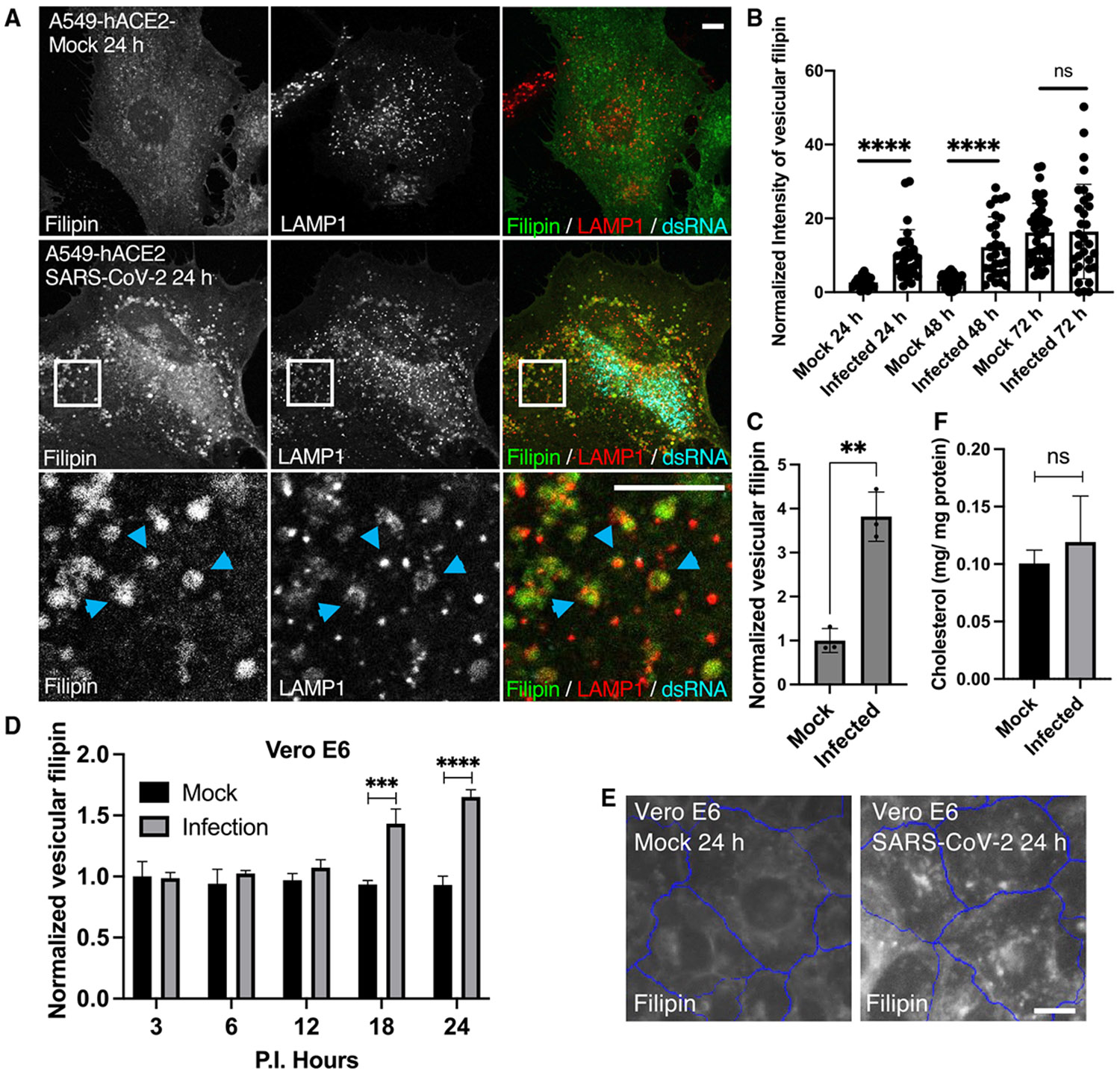
Characterization of cellular free cholesterol in SARS-CoV-2 infected cells (A–C) A549 cells stably expressing human ACE2 (A549-hACE2) were infected with SARS-CoV-2 and analyzed at the indicated times post-infection. Free cholesterol was visualized using filipin, and infection and lysosomal markers were detected using dsRNA and LAMP1, respectively. Representative images at 24 h post-infection are shown in (A), with colocalization between filipin and LAMP1 indicated by arrows. Quantification of filipin signal per cell is shown in (B). Vesicular filipin signal at 24 h post-infection was quantified by high-content imaging system across the mock and infected cells (>1,000 cells from each group, *n* = 3), and pooled results are shown in (C). (D and E) Similar analyses were performed in Vero E6 cells, and filipin signal was quantified at the indicated time points. (F) Free cholesterol levels were measured in mock- and SARS-CoV-2-infected A549-hACE2 cells by GC-MS. Data are presented as mean ± SD. Statistical significance was determined using the indicated tests. **p < 0.001, ***p < 0.001, ****p < 0.0001; n.s., not significant. Scale bars, 5 μm.

To examine how SARS-CoV-2 infection alters host cholesterol at cellular levels, we infected human lung carcinoma epithelial cells A549, stably expressing the viral entry receptor ACE2 of human species (A549-hACE2), with the Washington strain of SARS-CoV-2. We confirmed infection in approximately 80% of cells by immunostaining for double-stranded RNA (dsRNA) (Figures 1A and S1A). Using filipin staining to detect free cholesterol, we observed a marked increase in filipin signal in infected cells, appearing as punctate structures, compared to mock-treated cells (Figure 1A). Similar observations were made in a different ACE2-overexpressing A549 cell line (BEI, NR-53821) (Figure S1B). Co-staining with an antibody against late endosomal and lysosomal membrane protein LAMP1 revealed that the filipin puncta colocalized with LAMP1-positive vesicles (Figure 1A), indicating that cholesterol was sequestrated in the late endosomes and lysosomes. Due to the similarity of these two organelles, we refer to them as lysosomes hereafter. Quantification of confocal images showed that lysosomal cholesterol levels significantly increased at 24 and 48 h post-infection (Figure 1B). Although filipin puncta were also present at 72 h post-infection, mock cells showed elevated filipin signal, likely due to cell responses associated with high cell density^41^ (Figure 1B). Further analysis of over 1,500 infected cells using a high-content imaging system confirmed a consistent increase in filipin-positive puncta (Figure 1C). To validate this cholesterol alteration, we infected monkey kidney cells Vero E6 with SARS-CoV-2, resulting in approximately 80% infection rate (Figure S1C). High-content imaging revealed that the infected cells displayed a significant 50%–60% increase in cholesterol relative to LAMP1, compared with mock-treated cells, at 18 and 24 h post-infection. (Figures 1D and 1E). This change persisted beyond the viral eclipse phase (~10 h).^42^ Lysosome numbers remained similar to that before 18 h post-infection and were modestly reduced at 24 h post-infection compared with mock-infected controls (Figure S1D). Despite this decrease, lysosomal cholesterol levels were increased (Figures 1D and 1E), indicating that cholesterol accumulation is not attributable to increased lysosome abundance. Gas chromatography-mass spectrometry (GC-MS) analysis of total cholesterol levels in mock and infected A549 cells showed no significant difference (Figure 1F), suggesting that SARS-CoV-2 infection mainly alters cholesterol distribution within cells, possibly due to impaired lysosomal cholesterol transport.

### Viral protein ORF3a is responsible for increased lysosomal cholesterol

**Figure 2.**
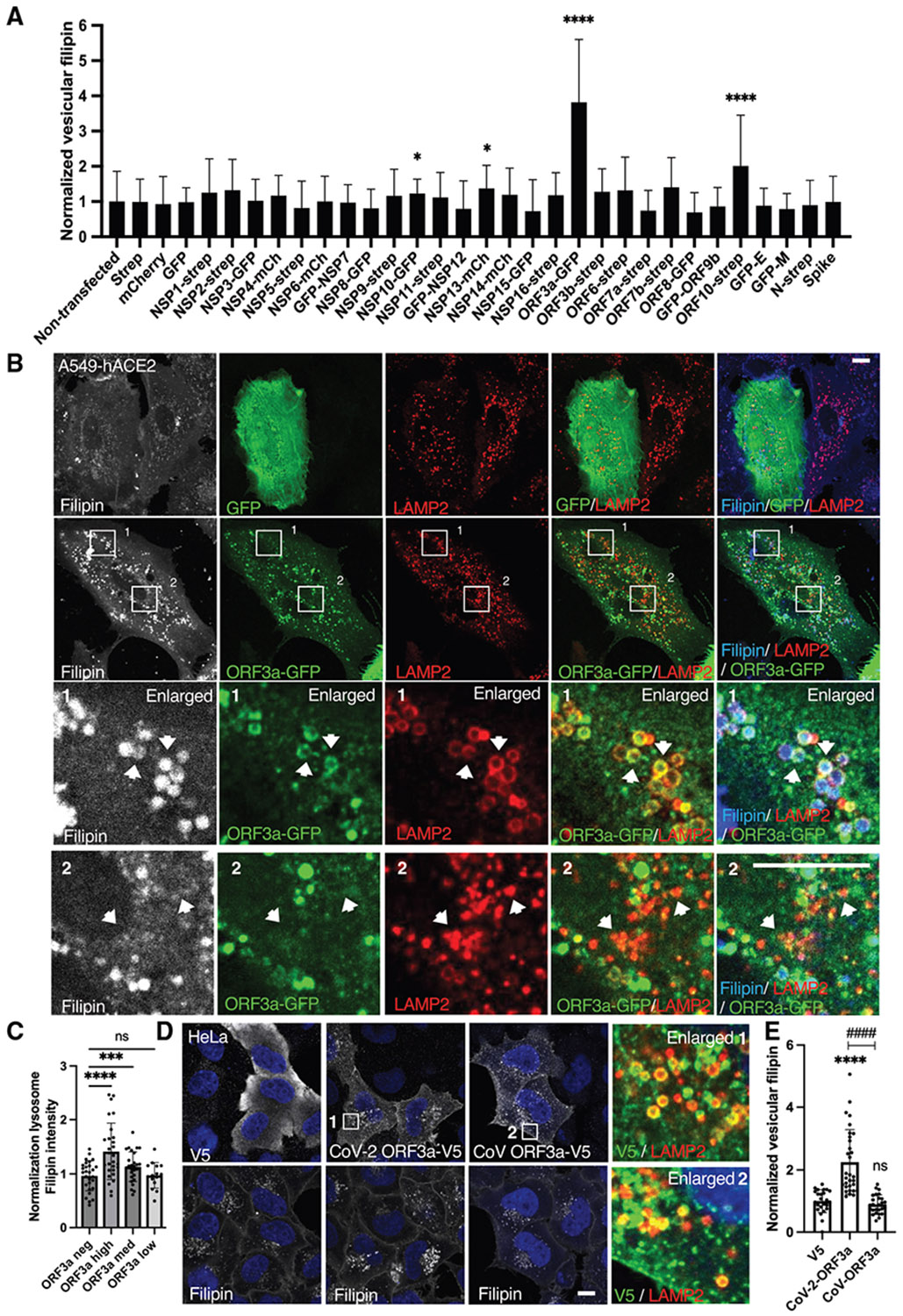
Identification of SARS-CoV-2 proteins responsible for lysosome cholesterol sequestration (A) A549 cells stably expressing human ACE2 (A549-hACE2) were individually transfected with plasmids encoding SARS-CoV-2 proteins. Free cholesterol was visualized using filipin and antibodies against the epitope tags were used to identify transfected cells. Spike protein has no tag and was identified with an antibody. Filipin intensity was quantified with FIJI in twenty to fifty cells from two independent experiments. (B) A549-hACE2 cells were transfected with ORF3a-GFP plasmid, as in (A), and co-stained with filipin and GFP and LAMP2 antibodies. (C) HeLa cells expressing ORF3a-GFP were stratified by GFP expression level (negative, high: top 33%, medium: middle 33%, or low: bottom 33%) and lysosomal filipin intensity was quantified within LAMP2-defined regions. (D and E). HeLa cells were transfected with plasmids encode V5 fused ORF3a proteins from SARS-CoV or SARS-CoV-2. V5 alone served as a control. Bar graphs are presented as mean ± SD vesicular filipin. *p* values were determined using *t* test or one-way ANOVA test. *p < 0.05, ****p < 0.0001 (vs. control), ####*p* < 0.0001 (CoV vs. CoV-2); n.s., not significant. Scale bars, 5 μm. Framed images in (C) were enlarged 9.36-folds.

To identify the specific viral proteins responsible for lysosomal cholesterol sequestration, we transfected A549-hACE2 cells with 28 plasmids individually, which encode SARS-CoV-2 proteins, and quantified filipin puncta 24 h post-transfection. Most viral proteins (24 out of 28) did not show a significant effect on cholesterol distribution. NSP10 and NSP13 had modest effects, ORF10 doubled the filipin signal, and ORF3a produced the strongest increase in filipin puncta (Figure 2A). ORF3a, expressed from a SARS-CoV-2 subgenomic RNA, is an accessory protein. Unlike NSP10, NSP13, or ORF10, which were predominantly cytoplasmic (Figure S1E), ORF3a displayed punctate or halo-like structures that partially co-localized with LAMP2 (Figure 2B), consistent with previous reports of ORF3a localization to endosomes/lysosomes.^12,15,18^ Remarkably, ORF3a-positive lysosomes appeared swollen and were filled with filipin (Figure 2B, arrows in enlarged frame 1), in contrast to ORF3a-negative lysosomes in the same cells, which remained small and displayed minimal filipin signal (Figure 2B, arrows in enlarged frame 2). This suggests that ORF3a, particularly when localized on lysosomes, plays a critical role in cholesterol sequestration. To further test this, we used an ORF3a mutant harboring a disruption of the tyrosine-based sorting motif (Y160A), which impairs ORF3a lysosomal targeting.^14,43,44^ Compared with wild-type ORF3a, the ORF3a-Y160A mutant showed reduced lysosomal localization and failed to increase cholesterol levels (Figures S1F and S1G). Moreover, quantification at the individual lysosome level revealed increased cholesterol accumulation in ORF3a-positive lysosomes, with higher levels observed at increased ORF3a expression (Figure 2C). Together, these results indicate that ORF3a-induced lysosomal cholesterol sequestration requires its localization to lysosomes.

To further validate this observation, we transfected HeLa cells with an ORF3a plasmid tagged with a V5 epitope. ORF3a-V5 also formed punctate or halo structures, partially co-localized with LAMP2, and significantly increased filipin signal (Figure 2D and 2E), demonstrating that ORF3a enhances lysosomal cholesterol accumulation across different cell types. Additionally, we included ORF3a from SARS-CoV, which shares over 70% sequence identity with SARS-CoV-2 ORF3a, as a comparison. Interestingly, while SARS-CoV ORF3a also displayed a punctate distribution, it did not increase filipin (Figure 2D and 2E), suggesting a specificity in cholesterol sequestration by SARS-CoV-2 ORF3a.

Together, these results demonstrate that SARS-CoV-2 infection sequesters host lysosomal cholesterol primarily through the action of ORF3a. Expression of ORF3a alone is sufficient to drive cholesterol accumulation in lysosomes, which depends on its lysosomal localization. The differential effects on lysosomal cholesterol between ORF3a proteins from SARS-CoV and SARS-CoV-2 may offer insights into the underlying mechanisms.

### The structural difference between SARS-CoV and SARS-CoV-2 ORF3a

**Figure 3.**
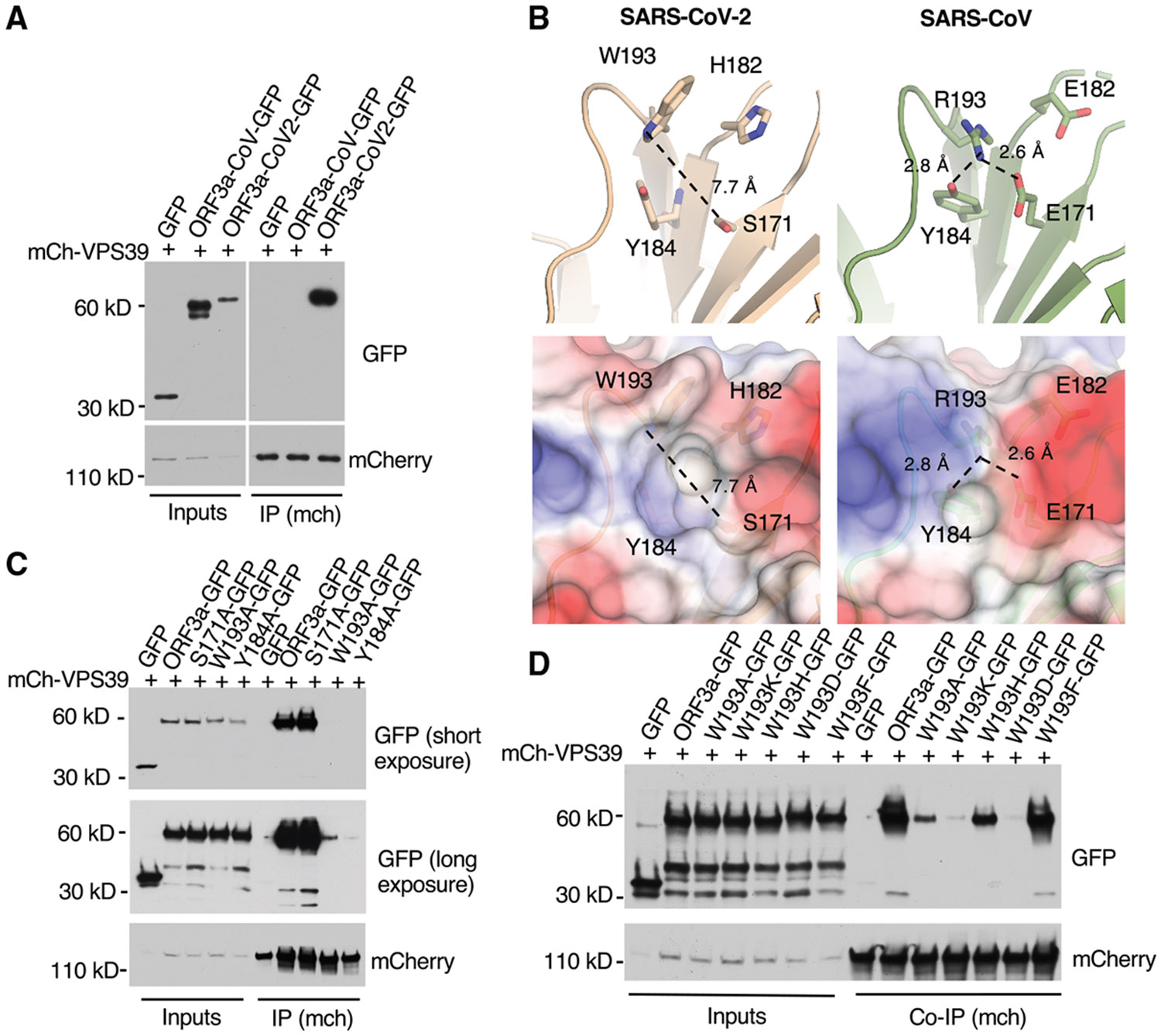
Structural analysis of ORF3a-VPS39 interaction interface in ORF3a (A) HeLa cells were co-expressed with mCherry-tagged VPS39 and GFP-tagged ORF3a constructs. Interaction between ORF3a and VPS39 was assessed by coimmunoprecipitation and immunoblotting for GFP. (B) The structures of ORF3a proteins from SARS-CoV-2 (beige) and SARS-CoV (green) are shown with cartoon representation of the indicated residues (top). The side chains are depicted as sticks with color code for carbon (beige or green), nitrogen (blue), and oxygen (red). The presence of W193 in SARS-CoV-2 induces an open conformation of the divergent loop that is 7.7 Å away from S171 (dotted line), while R193 in SARS-CoV forms hydrogen bonds with D171 or Y184 (dotted lines). The images at the bottom represent overlays between electrostatics surface and the cartoon representations. Positively charged surface is depicted in blue, hydrophobic surface in white, and negatively charged surface in red. All images and measurements were performed using PyMol v.2.4.0. (C and D) Transfection and co-immunoprecipitation was performed as described in (A) to detect the interaction between mCh-VPS39 and GFP-tagged SARS-CoV-2 ORF3a and its mutants.

Previous studies revealed that SARS-CoV-2 ORF3a, but not SARS-CoV ORF3a, interacts with the host protein VPS39^12,15,18,45^ (Figure 3A), a subunit of the HOPS complex. Our recent work, using VPS39 CRISPR-knockout (KO) cells, showed that the HOPS complex is essential for lysosomal cholesterol egress.^22^ SARS-CoV-2 infection did not downregulate the expression of VPS39 or VPS11, another HOPS subunit (Figure S2A). These results suggest that SARS-CoV-2 ORF3a disrupts lysosomal cholesterol egress by inhibiting VPS39 functions. To test this, we aimed to disrupt the ORF3a-VPS39 interaction by introducing specific point mutations.

Miao et al. identified the residues S171 and W193 in SARS-CoV-2 ORF3a as crucial for its interaction with VPS39, as mutating these residues to those in SARS-CoV ORF3a abolished the interaction.^18^ Structural analysis of SARS-CoV-2 ORF3a^46,47^ revealed that W193, Y184, S171, and H182 cluster near each other (Figure 3B, top), with W193 and Y184 forming a hydrophobic surface likely involved in protein-protein interactions, while S171 and H182 are positioned outside this hydrophobic surface (Figure 3B, bottom). In our alanine mutation screen, we found that W193A and Y184A mutations weakened or disrupted the interaction, whereas S171A or H182A mutations did not (Figures 3C, S2C, and S2D). This indicates that W193 and Y184, but not S171 or H182, are essential for binding. Further structural analysis indicates that the hydrophobic surface formed by W193 and Y184 (7.7 Å) in SARS-CoV-2 ORF3a is occluded in SARS-CoV ORF3a due to a hydrogen bond between R193 and E171 (2.6 Å) and Y184 (2.8 Å), resulting a “closed conformation” (Figure 3B, top). This model is supported by the observation that the S171E mutation impairs the interaction,^18^ likely due to the large hydrophilic side chain of glutamic acid obstructing the hydrophobic surface formed by W193 and Y184, thereby blocking the interaction. Collectively, these findings suggest that W193 and Y184 form the primary binding site, while S171 and H182 contribute to an optimal binding environment.

Given that both W193 and Y184 are aromatic amino acids, we hypothesized that their aromatic rings play a role in the interaction. To test this, we mutated W193 to various amino acids and evaluated the effects on binding. Replacing W193 with another hydrophobic aromatic amino acid, such as phenylalanine, preserved the interaction (Figure 3D). However, substituting W193 with a charged aromatic amino acid, like histidine, or with a hydrophobic but non-aromatic amino acid, like alanine, reduced binding. Furthermore, replacing W193 with a charged, non-aromatic amino acid, such as lysine or aspartic acid, nearly abolished the interaction (Figure 3D). These results suggest that both the aromatic ring and hydrophobicity of W193 are critical for the binding of SARS-CoV-2 ORF3a to VPS39. Importantly, this region and its surrounding residues are highly conserved across clinically relevant SARS-CoV-2 variants, including Beta, Gamma, Delta, and Omicron (Figure S2E), suggesting that this interaction is evolutionarily conserved and likely plays a fundamental role in ORF3a function.

### ORF3a-VPS39 interaction interrupts NPC2 trafficking

**Figure 4.**
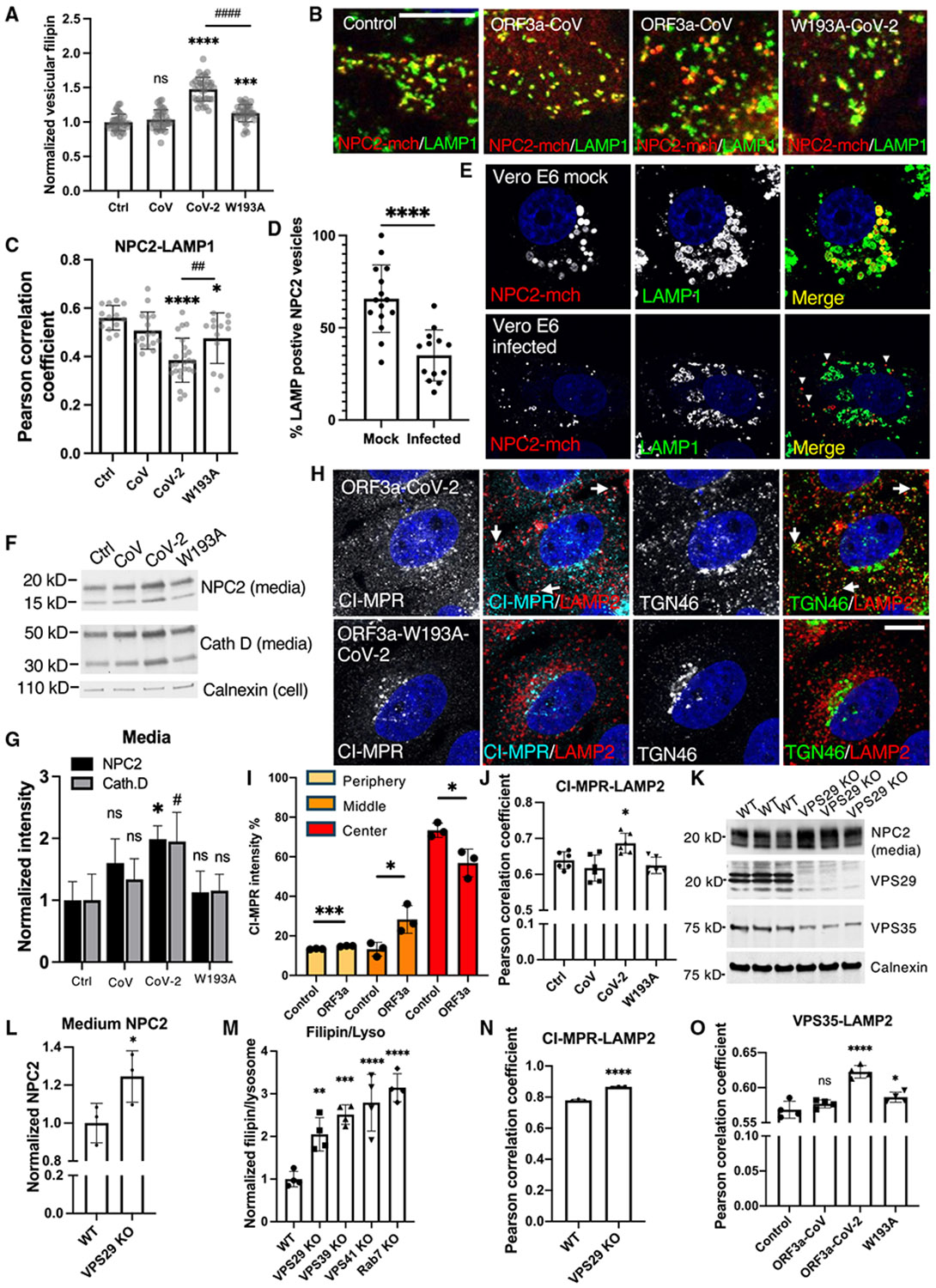
Characterization of NPC and endosome-to-Golgi trafficking proteins in ORF3a-expressed cells (A) The indicated HeLa-Flp-In cells were quantified for free cholesterol by high-content imaging. (B and C) The indicated cells were transfected with NPC2-mCherry and immunostained with the indicated antibodies. Colocalization of NPC2-mCherry with LAMP1 was assessed by confocal microscopy quantified. (D and E) Vero E6 cells were transfected with NPC2-mcherry and then infected with SARS-CoV-2, fixed, and immunostained 24 h post infection. Colocalization of NPC2-mCherry with LAMP1 was assessed by confocal microscopy and quantified with FIJI. (F and G) Equal volumes of serum-free media was placed for the Flp-In cell culture and subjected to immunoblotting using the antibodies indicated. Cell input was controlled using calnexin in corresponding lysates. Quantification was based on three independent experiments. (H–J) The indicated cells were analyzed for protein localization and colocalization by confocal microscopy (H) and high-content imaging (I). Periphery was defined as a ring area shrunk from cell boundary with a gap distance 15 pixels. Center was defined as a dilated circle from nuclear boundary with a distance of 20 pixels. Middle was defined as the area between periphery and center (I). Pearson correlation coefficient of CI-MPR LAMP2 was quantified with high-content imaging in the indicated cells (J). (K and L) Secretion and cellular levels of indicated proteins were analyzed in wild-type and VPS29 knockout HeLa cells. (M) Vesicular filipin was measured by high-content imaging and normalized to lysosomes in the indicated cells. (N and O) Pearson correlation coefficient was measured with high-content imaging in the indicated cells. Bar graphs are presented as mean ± SD. *p* values were determined using *t* test or one-way ANOVA test. *p < 0.05, **p < 0.01 (vs. control), ##*p* < 0.01 (CoV-2 vs. W193A), ***p < 0.001, ****p < 0.0001 (vs. control), ####*p* < 0.0001 (CoV-2 vs. W193A); n.s., not significant. Scale bars, 5 μm. Control (ctrl) and linker peptides; CoV, SARS-CoV ORF3a; CoV-2, SARS-CoV-2 ORF3a; and W193A, SARS-CoV-2 ORF3a-W193A mutant.

With the understanding of structural basis of the interaction interface, we chose W193A mutation to block the interaction between ORF3a and VPS39. To minimize potential artifacts from overexpression, we individually introduced SARS-CoV ORF3a, SARS-CoV-2 ORF3a, the SARS-CoV-2 ORF3a W193A mutant, and a linker peptide that serves as a control into HeLa-Flp-In cells to generate tetracycline-inducible protein-expression cell lines. This system controls protein expression levels via a preengineered recombination site in the cells.^48,49^ Likely due to low protein expression, we could not detect the ORF3a proteins by immunofluorescence (Figure S3A), but immunoblotting showed expected bands in response to induction by the tetracycline analog doxycycline (Figure S3B). Using immunoprecipitation, we confirmed that in the Flp-In cells, CoV-2 ORF3a interacted with endogenous VPS39 and the W193A mutation abolished this interaction (Figure S3C). High-content imaging quantification revealed that doxycycline-induced expression of ORF3a increased filipin signaling by 50%–60% compared to the control cells (Figures 4A, S3D, and S3E). Importantly, the W193A mutant significantly reduced ORF3a-induced cholesterol accumulation (Figures 4A, S3D, and S3E), demonstrating that the ORF3a-VPS39 interaction is responsible for the cholesterol defect.

Previous research has shown that ORF3a expression increases lysosome pH^12^ and compromises lysosomal membrane integrity,^13^ raising the question of whether lysosomal membrane damage or dysfunction leads to cholesterol sequestration. To investigate this, we permeabilized lysosomal membranes using the lysosomotropic agent Leu-Leu-*O*-Me (LLOMe) and confirmed membrane damage with the marker galectin-3^50^ (Figure S3F). Elevated lysosomal cholesterol was detected in the LLOMe treatment as well as in the treatment by lysosome deacidifying reagents, such as the ionophore monensin or V-ATPase inhibitors bafilomycin and chloroquine (Figure S3G). These results showed that lysosome damage and pH increase could sequestrate cholesterol. However, we did not detect galectin-3 puncta in the Flp-in cells (Figure S3H), suggesting that ORF3a does not markedly damage lysosomes in these cells, and that there are other mechanisms that interrupt cholesterol independent of lysosome damage.

We next examined the lysosomal cholesterol transporters NPC proteins in the Flp-In cells. Immunoblotting the endogenous proteins did not show any appreciable difference in NPC1 levels among the four Flp-In cell lines (Figure S4A). Immunofluorescence confocal microscopy showed that NPC1 colocalized with LAMP2 (Figure S4B), and high-content imaging analysis confirmed no significant difference in Pearson correlation coefficients across conditions (Figure S4C). In contrast, transfected NPC2-mCherry displayed marked mislocalization from the lysosomes in the CoV-2-ORF3a cells (Figure 4B), which was quantitatively supported by a reduced Pearson correlation coefficient between NPC2 and LAMP1 (Figure 4C). This defect was largely rescued by the ORF3a-W193A mutant (Figure 4B and 4C). Consistently, in SARS-CoV-2-infected Vero E6 cells, LAMP1-positive NPC2-mCherry vesicles were significantly decreased, compared to the mock-infection cells (Figure 4D and 4E). To assess endogenous NPC2 localization, we performed Lyso-IP^51^ in the Flp-In cells stably expressing a lysosomal membrane tag. Immunoblotting showed a modest reduction of NPC2 in isolated lysosomes from ORF3a-expressing cells compared with control and W193A mutant cells (Figures S4D and S4E), further supporting that ORF3a redistributes NPC2 away from lysosomes.

Our previous work demonstrated that mislocalized NPC2 is retained in Rab7-positive endosomes or secreted extracellularly in VPS39-KO cells.^22^ Similarly, in CoV-2 ORF3a cells, we observed a significant increase in Rab7-positive, LAMP2-negative NPC2 vesicles (Figures S4F and S4G), along with increased NPC2 secretion (Figure 4F and 4G). Consistent with enhanced secretion, total cellular NPC2 levels in CoV-2 ORF3a cells showed a decreasing trend compared with other Flp-In cells (Figures S4A and S4H). Together, these findings suggest that ORF3a redistributes NPC2 to non-lysosomal compartments, including Rab7 endosomes and secretory vesicles, indicative of impaired lysosomal trafficking and altered endosomal maturation.

These results prompted us to examine whether lysosomal transmembrane proteins, such as LAMPs, are properly localized to lysosomes. We used the Ragulator complex as a lysosomal marker, as it associates with the lysosomal membrane through lipidation independently of the biosynthetic pathway.^52^ Measurement of colocalization between LAMP2 and the Ragulator subunit LAMTOR4 revealed no differences among the Flp-In cell lines (Figures S4I and S4J), indicating that lysosomal membrane protein localization is preserved. In contrast, lysosomal luminal proteins were affected—in addition to NPC2, cathepsin D showed increased secretion in ORF3a-expressing cells (Figure 4F and 4G). Together, these findings indicate that ORF3a selectively impairs the trafficking of lysosomal luminal proteins, likely through disruption of VPS39/HOPS-dependent endosome-lysosome maturation and protein transport between the TGN and endosomes.

### ORF3a disturbs the endosome-to-TGN transport

The NPC2 trafficking defect observed in CoV-2-ORF3a cells resembled what was observed in VPS39-deleted cells in our previous study, in which the sorting receptor for lysosomal lumenal proteins, CI-MPR, is abnormally degraded in lysosomes.^22^ In contrast, Flp-In CoV-2-ORF3a cells did not exhibit a reduction in total CI-MPR by immunoblotting (Figure S4A), likely reflecting only partial inhibition of VPS39 due to relatively low ORF3a expression rather than complete loss of VPS39 function. In a transient transfection experiment, which typically yields higher protein expression, we quantified CI-MPR intensity by high-content imaging. Under these conditions, CI-MPR levels were reduced in ORF3a-expressing cells, whereas the ORF3a-W193A mutant partially restored CI-MPR levels (Figure S5A).

Although the expression level remained unchanged in the CoV-2-ORF3a Flp-In cells, CI-MPR dispersed from its usual location near the microtubule-organizing center (MTOC) to the cell periphery (Figure 4H). In addition, a more severe dispersion of the TGN protein TGN46 was observed in CoV-2-ORF3a cells (Figure 4H). The positioning changes of CI-MPR and TGN46 was confirmed by high-content imaging analysis (Figures S4I and S5C), in which cellular area between MTOC and cell boundary was divided into ‘‘center’’, ‘‘middle’’, and ‘‘periphery’’ to reflect protein cellular distribution.^53^ We also examined Golgi matrix protein 130 (GM130) but did not see a change in dispersion (Figure S5D), indicating that the dispersion of CI-MPR and TGN46 was not due to Golgi fragmentation.^54,55^ The dispersed CI-MPR and TGN46 were partially colocalized with LAMP2 (Figure 4H, arrows), and quantitative results showed an increased Pearson correlation coefficient between CI-MPR and LAMP2 (Figure 4J). Consistently, CoV-2-ORF3a cells had an increased localization between TGN46 and Rab7 (Figures S5E and S5F). These results indicate that CI-MPR and TGN46 were retained within the LAMP2- or Rab7-positive endosomes and lysosomes in the CoV-2-ORF3a cells, suggesting that TGN-to-endosome transport was increased and/or retrieval from endosomes and lysosomes was decreased, resulting in protein prolonged retention within the endosome-lysosome system.

Retrieval of endosomal proteins relies on the retromer^20,56^ and retriever^57^ complexes. KO of their shared subunit VPS29 resembled the defects observed in CoV-2 ORF3a cells, including increased NPC2 secretion (Figure 4K and 4L), lysosomal cholesterol accumulation (Figure 4M), and enhanced colocalization of CI-MPR (Figure 4N) and TGN46 (Figure S5G) with LAMPs. These results indicate that disruption of endosomal protein retrieval impairs lysosome cholesterol egress. We next examined the Flp-In cells and found that the retromer subunit VPS35, which mediates endosome-to-Golgi transport, exhibited increased colocalization with LAMP2 in CoV-2-ORF3a cells (Figures S4O and S5H). These results are in line with previous reports showing increased CI-MPR and VPS35 in Rab7 vesicles in ORF3a-expressed cells.^14^ Together, these findings indicate that the ORF3a-VPS39 interaction disrupts endosome-to-TGN transport by retaining retromer in LAMP2-positive endosomes/lysosomes, resulting in reduced recycling of CI-MPR and targeting of NPC2 to lysosomes.

### ORF3a reduced BMPs

**Figure 5.**
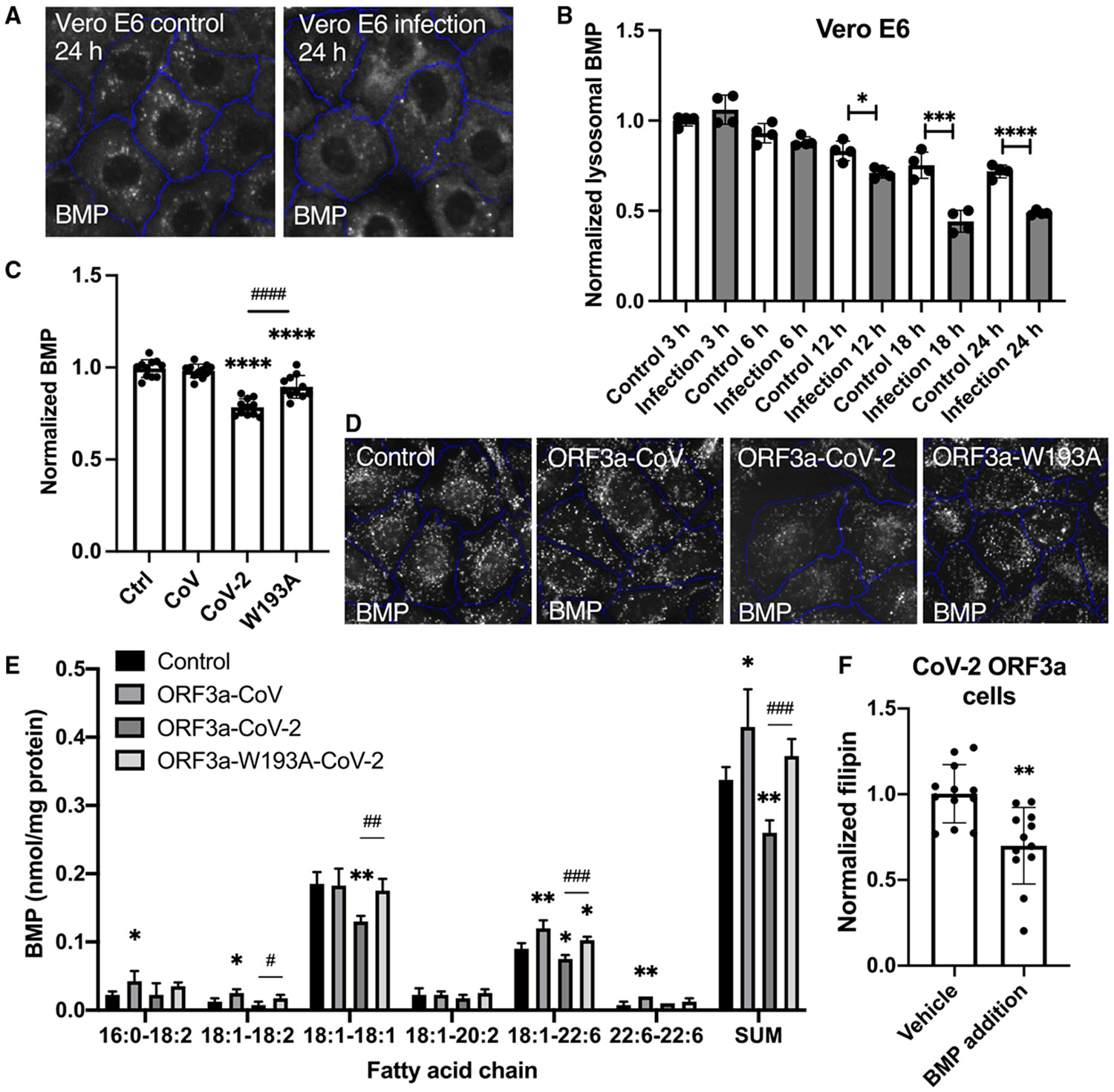
Analysis of BMP levels (A and B) Vero E6 cells were infected with SARS-CoV-2, fixed at the indicated time points post-infection, and immunostained with BMP and LAMP1 antibodies. LAMP1-positive BMP puncta were quantified with a high-content imaging system. Representative images were shown in (B). (C and D) BMP in the HeLa Flp-In cells were measured as described in (A). (E) The indicated cells were harvested and lysed for total lipid extraction. Shotgun lipidomics was performed to identify and quantify bis(monoacylglycero)phosphate (BMP) species based on the fatty acid chains, labeled as carbon number: double-bond number. BMP was normalized to protein concentrations. (F) Cells were incubated with serum-free media containing 1% BSA and 1 μM BMP for 2 h, fixed, and stained with filipin. Filipin levels were measured with high-content imaging. Bar graphs are presented as mean ± SD. *p* values were determined using *t* test or one-way ANOVA test *p < 0.05, **p < 0.01, ##*p* < 0.01, ***p < 0.001, ****p < 0.0001, ####*p* < 0.0001; n.s., not significant. Scale bars, 5 μm. Control (ctrl) and linker peptides; CoV, SARS-CoV ORF3a; CoV-2, SARS-CoV-2 ORF3a; and W193A, SARS-CoV-2 ORF3a-W193A mutant.

BMPs, localized exclusively on late endosome and lysosome membranes, physically interact with NPC2 to facilitate cholesterol egress.^35,36^ Using an antibody against BMPs,^27^ we observed a significant reduction in BMP levels in SARS-CoV-2-infected Vero E6 cells, starting 12 h post-infection (Figure 5A and 5B). To test whether this alteration was related to ORF3a, we examined the Flp-In cells and found that the BMP level was reduced by 20% in CoV-2-ORF3a cells, compared with the control cells (Figure 5C and 5D). This reduction was partially rescued in W193A cells, while the CoV-ORF3a cells exhibited no change (Figure 5C and 5D). Shotgun lipidomics of total lipids extracted from the Flp-In cells confirmed these results—the two most abundant BMP species, 18:1-18:1 and 18:1-22:6, were reduced by approximately 20% in CoV-2-ORF3a cells and restored in W193A cells (Figure 5E). These results indicate that CoV-2-ORF3a alone can decrease BMP levels, which is dependent on the ORF3a-VPS39 interaction, aligned with the observed cholesterol sequestration. Furthermore, exogenous BMP treatment for 2 h in serum-free media decreased the lysosomal cholesterol levels by 25% in CoV-2-ORF3a cells (Figure 5F). Together, these results suggest that reduced BMP levels constitute an additional mechanism underlying SARS-CoV-2-induced cholesterol sequestration.

### ORF3a interrupted lysosome-mitochondrion interaction

To investigate the mechanism underlying BMP reduction, we examined key enzymes involved in BMP biosynthesis and turnover, including CLN5,^39^ PLD3, PLD4,^40^ ABHD6,^37^ and PLA2G15.^58^ With the exception of PLA2G15, which did not display a punctate pattern upon transfection, we did not observe a reduced colocalization with lysosomes (Figures S6A-S6H) or reduced expression levels (Figure S6I) of these proteins, suggesting that these enzymes are not responsible for the BMP reduction in CoV-2-ORF3a cells.

**Figure 6.**
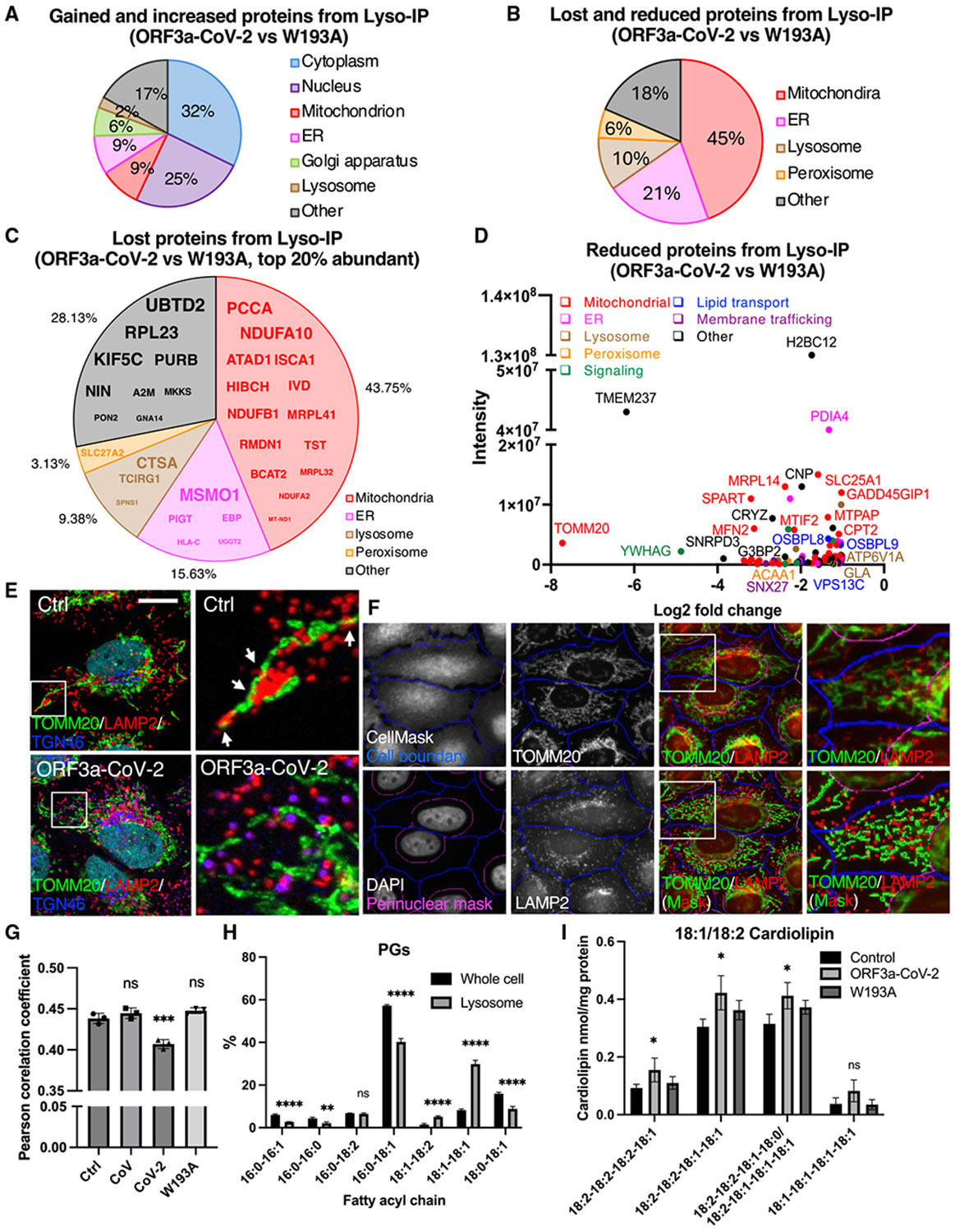
Decreased lysosome-mitochondrion interaction (A–D) Lysosomal proteomes from ORF3a- and W193A-expressing cells were analyzed by mass spectrometry. Proteins enriched or uniquely present in ORF3a lysosomes are shown in (A), while proteins reduced or lost are shown in (B). The most abundant lost proteins are highlighted in (C), and reduced proteins are summarized in (D). Proteins are categorized based on cellular localization or function. (E) Representative images showing lysosome-mitochondrion interactions in control and CoV-2 ORF3a cells, with interactions indicated by arrows. Scale bars, 5 μm. (F and G) The indicated cells were stained for TOMM20 (mitochondria) and LAMP2 (lysosomes) and imaged by high-content microscopy. Representative images and analysis masks are shown (F). Colocalization between lysosomes and mitochondria was quantified using Pearson’s correlation coefficient (G). (H and I) Total lipids were extracted from the indicated whole cells and isolated lysosomes and subjected to shotgun lipidomics analysis. PG, phosphatidylglycerol. Bar graphs are presented as mean ± SD. *p* values were determined using *t*. test or one-way ANOVA test. *p < 0.05, **p < 0.01, ****p < 0.0001; n.s., not significant.

We then isolated the lysosomes by Lyso-IP^51^ followed by mass spectrometry of lysosomal proteomes from the control, CoV-2-ORF3a, and W193A Flp-In cells (Table S1). Comparison revealed 277 proteins uniquely present in CoV-2-ORF3a samples (gained, Table S2), and 348 proteins increased at least 2-fold relative to W193A cells (increased, Table S3). In contrast, 160 proteins were undetected (lost, Table S2), and 87 proteins decreased by ≥ 50% in CoV-2-ORF3a samples (decreased, Table S3). Gene Ontology analysis using DAVID bioinformatics resources indicated that the gained and increased proteins largely originated from cytoplasm (32%) and nuclei (25%) (Figure 6A), while the lost and decreased proteins were predominantly mitochondrial (45%) and ER-derived (21%) (Figure 6B). Among the top 20% of lost proteins, 43.7% proteins were mitochondrial, including outer membrane, inner membrane, and matrix proteins (Figure 6C). Similarly, in the reduced proteins, the majority was from mitochondria, regardless of their mitochondrial localization (Figure 6D). These results suggest a reduced lysosome-mitochondrion interaction in CoV-2-ORF3a cells.

Confocal imaging showed frequent lysosome-mitochondria interactions in control cells (Figure 6E, top, arrows), while CoV-2 ORF3a cells exhibited fewer physical contacts, though organelles remained proximal (Figure 6E, bottom). To quantify the physical interactions, we defined the cytoplasm area in each cell and excluded the perinuclear clouds to reduce the false overlap due to a crowding of organelles in that area (Figure 6F). Indicated by Pearson correlation coefficient, the interactions between mitochondria and lysosomes were significantly decreased in the CoV-2-ORF3a cells and were rescued in the W193A cells (Figure 6G). High-content imaging of TOMM20 revealed slightly increased mitochondrial abundance and similar morphology in both CoV-2-ORF3a and W193A cells (Figures S7A, S7B, and S7C), confirming that the reduced lysosome-mitochondrion interaction was not due to fewer mitochondria or altered mitochondrial morphology.

We hypothesized that impaired mitochondrion-lysosome interaction may limit the mitochondrial transfer of phosphatidylglycerols (PGs), the precursors for BMP biosynthesis. Lipidomic analysis revealed no change in total PG levels among Flp-In cells (Figure S7D). However, comparison of PG species between whole-cell and isolated lysosomal fractions showed that lysosomes were enriched in 18:1-18:2 and 18:1-18:1 PGs, whereas other species were unchanged or decreased (Figure 6H). This pattern indicates a selective mitochondrion-to-lysosome transport of specific PG species. Notably, the 18:1-18:1 PG species was predominantly increased in lysosomes (Figure 6H), consistent with the most abundant BMP species being 18:1-18:1 (Figure 5E). We next analyzed cardiolipins, which are mitochondrial PG-derived lipids. Although total cardiolipin levels were not altered (Figure S7E), the species containing 18:1-18:1 fatty acyl chains were significantly increased in CoV-2 ORF3a-expressing cells (Figure 6I), inversely correlating with the reduction of 18:1-18:1 BMP (Figure 5E). These results suggest that disruption of PG export from mitochondria leads to increased local PG conversion to cardiolipin and reduced BMP synthesis in lysosomes.

### ORF3a disturbed mitochondrion-lysosome membrane contact sites

**Figure 7.**
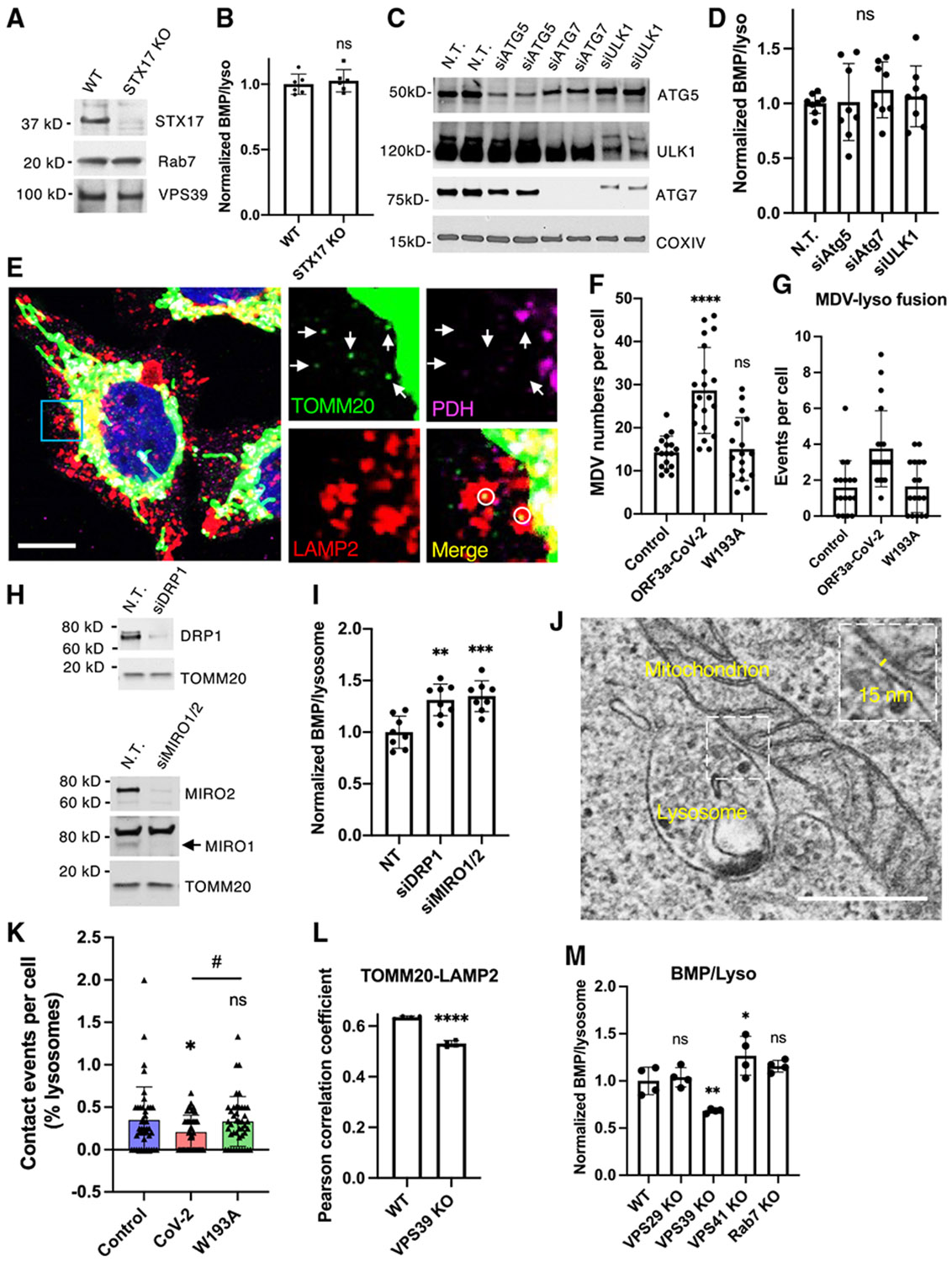
Involvement of impaired lysosome-mitochondrion membrane contact sites in BMP reduction (A and B) Wild-type and STX17 knockout HeLa cells were analyzed for protein levels by immunoblotting (A) and BMP levels by immunostaining and high-content imaging (B). (C and D) Wild-type HeLa cells were transfected with siRNA targeting the indicated autophagy genes. Non-targeting siRNA (NT) served as a control. Protein levels were examined by immunoblotting (C), and the BMP levels were measured by immunostaining followed by high-content imaging (D). (E–G) The Flp-In cells were fixed and immunostained with a TOMM20 (marker of mitochondrion-derived vesicles, MDVs), PDH (maker of mitochondria but not MDVs), and LAMP2 (lysosome marker) antibodies. Confocal microscopy was performed to identify MDVs (arrows) and the MDVs fused with lysosomes (circles) (E). The MDV numbers (F) and the number of lysosome-fused MDVs (G) were quantified using FIJI. Scale bars, 5 μm. (H and I) HeLa cells were transfected with the siRNAs of N.T., DRP1, or combined MIRO1 and MIRO2 and subjected to immunoblotting (H) or high-content imaging quantification of lysosomal BMPs (I). (J and K) Mitochondrion-lysosome membrane contact sites were visualized by electron microscopy (J) and quantified from 40 cells in each condition (K). Scale bars, 0.5 μm. (L) Wild-type and VPS39-knockout (KO) HeLa cells were immunostained and subjected to high-content imaging quantification of Pearson correlation coefficient between TOMM20 and LAMP2. (M) Wild-type (WT) and indicate KO HeLa cells were stained with BMP antibody and analyzed by high-content imaging. Bar graphs are presented as mean ± SD. *p* values were determined using *t.* test or one-way ANOVA test. *p < 0.05, **p < 0.01, ***p < 0.001, ****p < 0.0001; n.s., not significant.

The direct physical interactions between mitochondria and lysosomes can be achieved through autophagy, mitochondrion-derived vesicles (MDVs), and mitochondrion-lysosome MCSs. It was shown that autophagy flux is interrupted by ORF3a-VPS39 interaction, which inhibits HOPS-mediated fusion between autophagosomes and lysosomes.^34^ We observed the same defect shown as increased LC3-II levels in CoV-2-ORF3a cells (Figure S7F). However, inhibiting autophagy fusion by STX17 KO (Figure 7A) or interrupting earlier autophagy steps by knockdown (KD) of ATG5, ATG7, or ULK1 (Figure 7C) did not alter BMP levels (Figures 7B and 7D). These results excluded autophagy as a PG transport pathway.

We next tested MDVs identified by a combined staining of the MDV marker TOMM20 and a mitochondrial, but not MDV, protein, pyruvate dehydrogenase (PDH) (Figure 7E, arrows). We also included a lysosome marker to visualize the fusion between MDV and lysosomes (Figure 7E, circles). CoV-2-ORF3a cells exhibited more MDVs and MDV-lysosome fusion events (Figure 7F and 7G), which did not align with the BMP reduction (Figure 5C). Furthermore, KD of MDV biogenesis genes, DRP1 or MIRO1/2^59^ (Figure 7H), did not decrease BMP levels (Figure 7I), indicating that MDVs do not mediate PG delivery to lysosomes.

Last, we applied electron microscopy to assess mitochondrion-lysosome MCS. We randomly chose cells at low magnification and image areas containing abundant mitochondria at higher magnification. Lysosome-to-mitochondrion ratios were similar across cell types (Figure S7G), but MCS events, identified by intact membrane within 10–30 nm distance (Figure 7J), significantly decreased in CoV-2-ORF3a cells and were rescued in the W193A cells (Figure 7K). VPS39 is known to mediate the MCS formation in a HOPS-independent manner in yeast.^60^ Consistently, VPS39 KO decreased TOMM20-LAMP2 overlap (Figure 7L) and BMP level, whereas VPS29, VPS41, or Rab7 KO did not (Figure 7M). These findings suggest that ORF3a inhibited VPS39-mediated lysosome-mitochondrion MCS formation and impaired PG transportation, consequently reducing BMP synthesis.

## DISCUSSION

Our study identifies SARS-CoV-2 ORF3a as a potent disruptor of lysosomal cholesterol transport through dual interference with NPC2 trafficking and BMP biogenesis. By binding VPS39, ORF3a blocks retromer-mediated retrieval of CI-MPR and reduces mitochondrion-to-lysosome lipid transfer, thereby sequestering cholesterol within lysosomes. These findings expand the current understanding of cellular cholesterol regulation, uncover a regulatory pathway for BMP biosynthesis, and redefine the functions of HOPS and VPS39, revealing a viral strategy that links lysosomal lipid metabolism to SARS-CoV-2 pathogenesis.

Cholesterol is not only a structural component of cellular membranes but also a dynamic signaling molecule whose intracellular distribution dictates multiple metabolic pathways. Our results demonstrate that SARS-CoV-2 impedes lysosomal cholesterol egress (Figure 1), reinforcing the concept that compartmentalized cholesterol pools, rather than bulk cholesterol, drive cell physiology.^61,62^ ORF3a′s ability to retain cholesterol within lysosomes (Figures 2, 4A, S3D, and S3E) reveals a viral mechanism to perturb host lipid homeostasis and may underlie the systemic dyslipidemia observed in COVID-19 patients.^1-5^ Importantly, sequence analysis indicates that ORF3a is highly conserved across clinically relevant SARS-CoV-2 variants, including Beta, Gamma, Delta, and Omicron. The VPS39-interacting region required for ORF3a-mediated cholesterol retention is also conserved (Figure S2E), suggesting that this function is maintained across circulating strains. These findings support the clinical relevance of our observations and indicate that ORF3a-mediated disruption of lysosomal cholesterol trafficking is likely a conserved viral strategy. More broadly, our findings place lysosomal cholesterol transport at the crossroads of host-pathogen interactions and suggest that this pathway may represent a therapeutic vulnerability.

BMPs play essential roles in lysosomal biology and have been implicated in a wide range of human diseases,^29,30^ such as infectious disease^32,34,63,64^ and neurodegenerative disorders.^65,66^ Here, we demonstrate that SARS-CoV-2 and its ORF3a protein reduce BMP levels (Figures 5A-5E), and that restoring BMP partially rescues cholesterol egress (Figure 5F). This finding highlights BMP as a limiting factor for lysosomal cholesterol export, in agreement with prior studies.^35,36^ Notably, ORF3a alters PG metabolism by decreasing its lysosomal products (BMPs) and increasing its mitochondrial products (cardiolipins) (Figure 6I). This shift uncovers a previously unrecognized regulatory mechanism in BMP homeostasis, the supply of mitochondrial precursors. Although BMP levels were previously attributed mainly to lysosomal synthetic and hydrolytic enzymes,^37,39,40,58^ our data indicate that cross-organelle lipid transfer provides an additional layer of control. By systematically testing multiple routes, we found that mitochondrion-lysosome MCS likely serve as the pathway for PG transport, rather than autophagy or mitochondrial-derived vesicles (Figure 7). These findings position BMP regulation as a point of crosstalk between mitochondrial phospholipid metabolism and lysosomal cholesterol transport, with broad implications for both cellular physiology and viral pathogenesis.

The canonical view of HOPS has centered on its role as a tethering complex that mediates late endosome-lysosome and autophagosome-lysosome fusion.^21^ By leveraging the VPS39-ORF3a interaction and the W193A mutant, our study expands this paradigm by showing that VPS39 also regulates two distinct aspects of lysosomal activities, regulation of retromer-dependent trafficking (Figure 4) and formation of mitochondrion-lysosome MCS necessary for BMP synthesis (Figures 7J-7M). These dual roles highlight VPS39 as a multifunctional coordinator, integrating protein trafficking and lipid exchange. ORF3a′s simultaneous disruption of both processes reframes HOPS/VPS39 as a broader regulatory hub for lysosomal homeostasis rather than solely a membrane fusion mediator. Notably, VPS39 KO, but not VPS41 or RAB7 KO, reduced MCS formation and BMP levels (Figures 7L and 7M), suggesting that VPS39 can regulate these processes independently of canonical HOPS complex activity. Consistent with this, previous studies have reported that VPS39 can interact with alternative Rab GTPases in different cellular contexts^67,68^ and function at MCS in a HOPS-independent manner.^60^

Our study establishes ORF3a as a viral effector that directly manipulates host lipid metabolism. Unlike SARS-CoV ORF3a,^69^ its SARS-CoV-2 counterpart acquired the ability to bind VPS39,^15,17,18^ suggesting an evolutionary adaptation to exploit lysosomal cholesterol regulation. Since plasma membrane cholesterol promotes SARS-CoV-2 entry,^10,11^ restricting cholesterol export from lysosomes could reduce cholesterol at the plasma membrane and thereby limit the viral entry. Consistent with this model, pharmacological blockade of NPC1 with U18666A sequesters cholesterol in lysosomes and decreases SARS-CoV-2 infection (Figures S7H-S7J). In addition, BMPs are required for viral fusion within endo- somes/lysosomes,^64^ and reduced BMP levels may impair this step of the viral life cycle. Importantly, ORF3a is an accessory protein that is not incorporated into virions but translated only after infection from viral subgenomic RNAs.^70^ Thus, its interference with cholesterol export and BMP synthesis is unlikely to affect the ongoing infection but may restrict secondary infection events. Furthermore, ORF3a′s previously reported activities, including increasing lysosomal pH and damaging lysosomal membranes, may promote lysosome exocytosis, a route for viral egress.^12,16^ Together, these findings suggest that SARS-CoV-2 uses ORF3a to modulate multiple lysosomal functions, integrating cholesterol trafficking, lipid metabolism, and membrane dynamics to optimize different stages of its life cycle.

### Limitations of the study

Our study has several limitations. First, although we demonstrate ORF3a-induced cholesterol sequestration and identify VPS39-dependent mechanisms, the downstream consequences for viral replication, immune responses, and cell survival remain to be fully elucidated. Second, we show that ORF3a-mediated reduction of mitochondria-lysosome contacts is VPS39 dependent; however, the underlying mechanism remains unclear. Identifying VPS39-interacting partners on mitochondria will be important for defining this process. Third, while we observed reduced mitochondrion-lysosome contacts and altered BMP levels, the molecular intermediates mediating PG transfer remain undefined. Finally, the interplay between cholesterol sequestration and systemic lipid dysregulation observed in COVID-19 patients requires further integration with clinical data. Notably, SARS-CoV-2 infects hepatocytes,^71^ and given the conserved role of HOPS in lysosomal trafficking, similar lipid defects may occur in the liver, a central regulator of cholesterol and lipoprotein metabolism. Future work will, therefore, examine hepatocytes to determine whether ORF3a-mediated lysosomal disruptions contribute to the lipoprotein abnormalities observed in COVID-19 and long COVID.

In summary, this work uncovers cholesterol transport and BMP biogenesis as dual regulatory nodes governed by VPS39 and reveals how SARS-CoV-2 ORF3a exploits these pathways to rewire lysosomal lipid metabolism. These findings advance fundamental understanding of lysosomal protein trafficking and lysosome-mitochondrion crosstalk, broaden the functional repertoire of HOPS/VPS39, and provide new perspectives on viral pathogenesis and cholesterol-related diseases.

## STAR★METHODS

### EXPERIMENTAL MODEL AND STUDY PARTICIPANT DETAILS

#### Cell lines

Human male lung epithelial A549 cells and human female cervical adenocarcinoma epithelial cells HeLa cells were maintained in Dulbecco’s Modified Eagle Medium (DMEM) supplemented with 10% heat-inactivated fetal bovine serum (FBS), 25 mM HEPES, and MycoZap Plus-CL (Lonza) at 37°C in 5% CO_2_. A549 and HeLa were obtained from ATCC (CCL-185 & CRM-CCL-2). Vero E6 cells (ATCC CRL-1586) were cultured in DMEM supplemented with 10% heat-inactivated FBS, 1% penicillin/streptomycin, 2 mM L-glutamine, 1% non-essential amino acids, and 1% HEPES. HeLa Flp-In cells were maintained in DMEM supplemented as above, with tetracycline-certified FBS. A549-hACE2 cells were obtained as a gift from Dr. Kajon and from BEI Resources (NR-53821). All cell lines were maintained at 37°C with 5% CO_2_ and routinely tested for mycoplasma contamination using standard laboratory screening methods. Cell lines obtained from ATCC were authenticated by the vendor prior to distribution. No additional authentication was performed after receipt.

#### Viruses

Severe acute respiratory syndrome coronavirus 2 (SARS-CoV-2 isolate USA-WA1/2020) was obtained from BEI Resources (NR-52281). Virus stocks were propagated in Vero E6 cells for 72 h. Supernatants were collected, clarified by centrifugation at 1,000 × g for 10 min, aliquoted, and stored at −80°C. Viral titers were determined by standard plaque assay.^72^ All experiments involving infectious SARS-CoV-2 were performed in a biosafety level 3 (BSL-3) facility in accordance with institutional biosafety guidelines.

### METHOD DETAILS

#### SARS-CoV-2 infection

Coverslips or optic 96-well plates (Agilent, Santa Clara, CA) were coated with collagen solution (0.03–0.04 mg/mL collagen in 0.02 N acetic acid) for at least 30 min and washed twice with PBS prior to seeding. A549-hACE2 cells were seeded at 0.1 × 10^6^ cells per well (coverslips) or in at 0.012 × 10^6^ cells per well (96-well plate) and infected the following day with SARS-CoV-2 in DMEM (4% FBS) at MOI of 4. After 1hr incubated at 37°C cells were then washed twice with 1x PBS and maintained in complete media. Vero E6 cells were seeded in optical 96-well plate at 0.025 × 10^6^ cells per well and infected with SARS-CoV-2 at MOI 0.1 in 2% FBS media. The control cells were incubated with media only. At the indicated times post-infection, cells were fixed with 4% PFA.

#### Plasmid and siRNA transfection

Cells were seeded 24h before transfection and receive plasmids mixed with transfection reagent Lipofectamine 2000 (Thermo Fisher Scientific) or TransIT-LT1 (Mirus Bio) following the manufactures’ instructions. Concentration of DNA was optimized for each plasmid. Cells were analyzed at 24- or 48-h post transfection. On-TARGETplus SMARTpool siRNAs were applied (Horizon Discovery) for two-shot transfection at Day 1 and Day 3 after seeding. The transfection reagents Lipofectamine 2000 (Thermo Fisher Scientific) or TransIT-X2 (Mirus Bio) were used to deliver siRNA, and cells were analyzed 6–7 days after seeding.

#### CRISPR/Cas9 gene editing

Gene knockout cell lines were generated using the CRISPR/Cas9 system. Guide RNAs (gRNAs) were designed using Benchling, and 4–6 candidate 20-bp guides per gene were screened. Selected were cloned into the px458 plasmid containing GFP sequence (Addgene). Cells were transfected with two plasmids, and GFP-positive cells were collected by cell sorter 48 h after transfection. Transformants were kept in normal medium for another 12 days to allow single colony formation. Genomic DNA was extracted from individual colonies, and cleavage of the target sequence was tested by PCR followed by Sanger sequencing and immunoblotting. Three to six single colonies were pooled for experiment use.

Guide RNA target sequences used in this study:

Rab7 KO: TAGTTTGAAGGATGACCTCT and TTGCTGAAGGTTATCATCCT.

VPS29 KO: GCAAACTGTTGCACCGGTGT and CATAACTCTCTTTGGTGCAA.

Stx17 KO: TGGAGAAGACAGCTGTTACCAGGG and TTAAGATAGTAATCCCAACAGACC.

#### Generation of Flp-in cells

HeLa Flp-In host cells were generated with Flp-In Complete System (Thermo Fisher Scientific), following the manufacture instruction, and were transfected with the plasmids encoding FLAG-tagged constructs or linker peptide and subjected to hygromycin selection 48 h post transfection tetracycline-free DMEM. Following 2-week selection, transformants were kept in tet-free medium for another 12 days to allow single colony formation. Protein expression was induced with 1 μg/mL doxycycline for 16 h. Clones with moderate expression levels were selected and pooled based on FLAG immunoblotting.

#### Immunofluorescence, filipin staining, and confocal microscopy

Cells were fixed with 4% PFA and washed three times with PBS. Primary antibodies were diluted in PBS containing 1% BSA and 0.2% saponin and incubated for 1 h at 37^°^C or overnight at 4^°^C. Alexa-conjugated secondary antibodies (Thermo Fisher Scientific) were applied with the same saponin-contained BSA-PBS solution for 30 min at 37^°^C. Coverslips were mounted with DAPI-contained mounting reagents (Electron Microscopy Sciences) and allowed to dry at 37^°^C for at least 1 h. To stain free cholesterol, 25 μg/mL filipin complex (Millipore Sigma) in PBS was applied to fixed cells at 37^°^C for 30 min. When immunostaining was performed with filipin, primary antibodies and Alexa-conjugated secondary antibodies along with DRAQ7 (Novus Biologicals) were sequentially applied to filipin-stained cells without detergent permeabilization. Then cells were mounted with mounting reagents without DAPI (Electron Microscopy Sciences) at 37^°^C for at least 1 h. Images were acquired using a Zeiss LSM800 confocal microscope equipped with the software ZEN.

#### High-content imaging and image analysis

Cells in optical 96-well plates were fixed and stained as described above and maintained in PBS for immediate imaging. Imaging and analysis were performed using the CellInsight Microscope platform (Thermo Fisher Scientific). Nuclei (DAPI or DRAQ7) were used for autofocus and segmentation. Cell boundaries were defined using CellMask staining. Cells were excluded based on criteria including incomplete cell boundaries at image edges, abnormal size or shape, and signs of cell death.

For cellular punctum analysis, the cell boundary-enclosed areas are defined as ROI_A. Filipin, LAMP, or BMP puncta were detected as spots (2 pixels in size) using the software “spot detection” function. Line-shaped filipin signals from the plasma membrane were excluded by setting the validation criteria for shape. Puncta that overlapped with ROI_A were quantified by their fluorescence intensity. To measure colocalized signals, the software function of “colocalization” was applied, and the output feature “ROI_A(B) Correlation Coefficient” was extracted for the quantification of Pearson Correlation Coefficient.

To analyze the infected cells, fixed cells were immunostained with an dsRNA antibody, and its signals were used to set the threshold for detection of infected cells, using the function “Reference” of the software.

#### Transmission electron microscopy

Cells on glass coverslips were fixed in 0.1 M PIPES buffer containing 3% formaldehyde, 2% glutaraldehyde, and 1.5 mM CaCl2, then washed in several buffer changes before secondary fixation in 0.1 M PIPES containing: 1% osmium tetroxide for 15 min, 5 min PIPES rinse, 15 min 1% carbohydrazide in PIPES, 5 min PIPES rinse, and 15 min 1% osmium tetroxide (OCO). Samples were then washed with buffer, followed by ultrapure water, before being dehydrated through a graded series of ethanol to propylene oxide, infiltrated with Epon-Araldite resin, embedded, and heat-cured. The glass was removed from the cured resin blocks so the cells could be re-mounted for *en face* sectioning. Thin sections (60–80 nm) were mounted on Cu grids, post-stained with uranyl acetate and Reynold’s lead citrate, and examined in a Hitachi HT7700 TEM operated at 80 kV. Images were captured with an AMT XR-81 CCD camera.

#### Cholesterol measurement by GC-MS

Cells were collected in PBS with a cell scraper after washing twice with PBS, and the total lipids were extracted by adding lipid- extraction solvent (chloroform:methanol:acetic acid = 50:50:1) of 90% volume of PBS. The organic phase and aqueous phase were separated by 10 minute-centrifugation at 21,000 g. Organic phase was collected and transferred into a new tube and dried with vacuum. The extracted lipids were stored at −80°C for further analysis. Cholesterol calibration standard solutions were prepared from the concentration of 50 μg/mL to 400 μg/mL along with 80 μg/mL 5a-cholestane as the internal standard. The linear calibration curve was obtained with R2 = 0.9884. The dried lipids were dissolved with hexane and mixed with 4 μg 5a-cholestane. The GC-MS analysis was conducted on Agilent 6890/5973 with selected ion monitoring (SIM) mode.

#### Shotgun lipidomics

Lipid species were analyzed using a multidimensional mass spectrometry-based shotgun lipidomics approach.^73^ In brief, each cell sample homogenate containing 0.3 mg of protein which was determined with a Pierce BCA assay was accurately transferred to a disposable glass culture test tube. A premixture of lipid internal standards was added prior to conducting lipid extraction for quantification of the targeted lipid classes. Lipid extraction was performed using a modified Bligh and Dyer procedure (Wang and Han 2014), and each lipid extract was reconstituted in chloroform:methanol (1:1, v/v) at a volume of 400 μL/mg protein. Derivatization of lipid extracts for analysis of free fatty acid (FFA) and bis(monoacylglycero)phosphate (BMP) was performed as described previously^74,75^ before lipidomics analysis. Lyso-phosphatidylglycerol (LPG) in the aqueous phase was enriched using a HybridSPE cartridge. After washing with methanol, lysophospholipids were eluted with methanol/ammonia hydroxide (9:1), dried and reconstituted in methanol for lipidomics analysis.^76^ For shotgun lipidomics, individual lipid samples prepared as aforementioned was further diluted to a final concentration of ~500 fmol total lipids per μL. Mass spectrometric analysis was performed on a triple quadrupole mass spectrometer (TSQ Altis, Thermo Fisher Scientific, San Jose, CA) and a Q Exactive mass spectrometer (Thermo Scientific, San Jose, CA), both of which were equipped with an automated nanospray device (TriVersa NanoMate, Advion Bioscience Ltd., Ithaca, NY) as described.^77^ Identification and quantification of lipid species were performed using an automated software program.^78,79^ Data processing (e.g., ion peak selection, baseline correction, data transfer, peak intensity comparison, and quantitation) was performed as described.^79^ The results were normalized to the protein content (nmol lipid/mg protein).

#### Western Blot analysis

To detect secreted NPC2 and cathepsin D, cells were kept in a minimum volume of DMEM without serum for 6 h after three-time washing with the same medium. The media were collected and centrifuged at 1,000 g for 5 min to pellet any detached cells. The supernatants were mixed with SDS loading buffer and subjected to SDS-PAGE and immunoblotting. After collecting the media, cells were incubated in complete DMEM for 2 h. Cell lysates were obtained by using 2X SDS sample buffer.

#### Lysosome immunoprecipitation (Lyso-IP)

Lyso-IP was performed as previously described.^51^ Briefly, a TMEM192-HA construct was used to generate lentivirus in HEK293 cells, and viral supernatant was used to infect Flp-In cells, followed by antibiotic selection 48 h post-infection. For lysosome isolation, approximately 8 × 10^6^ cells were used per sample. Cells were collected in KPBS buffer and homogenized by passing through a 26-gauge needle. Homogenates were clarified by low-speed centrifugation and incubated with anti-HA magnetic beads for 30 min at 4^°^C. Immunoprecipitates were washed three times with KPBS and eluted twice in 1% SDS sample buffer. Eluates were combined for downstream analyses.

### QUANTIFICATION AND STATISTICAL ANALYSIS

ImageJ was used for quantifying fluorescence signals of randomly acquired images from at least three independent experiments. The signal intensity of vesicular filipin was measured using the function of “Analyze particles”, and co-localization was analyzed with the plug-in “PSC Colocalization”. High-content imaging results were obtained from more than 1,000 cells per well and at least 3 wells per experiment. The number of trials is specified in the legends where appropriate. The bar graphs show the pooled results displayed as mean ± SD. Colocalization results include frequency distribution. Statistical significance was assessed using GraphPad Prism 9 (GraphPad Software). For comparisons between two groups, two-tailed Student’s t-tests (paired or unpaired, as appropriate based on experimental design) were used. For comparisons involving more than two groups, one-way analysis of variance (ANOVA) was performed, followed by appropriate post hoc testing as indicated in the figure legends. All statistical tests were applied assuming approximately normally distributed data and similar variances between groups, as is standard for parametric analyses. Probability values and number of trials are given in the figure captions and the legends where appropriate. The statistical significance is generally denoted as follows: *, *p* < 0.05, **, *p* < 0.01, ***, *p* < 0.001, ****, *p* < 0.0001, and n.s., not significant.

## Supplementary Material

1

2

3

4

Supplemental information can be found online at https://doi.org/10.1016/j.celrep.2026.117544.

## Figures and Tables

**Table:** KEY RESOURCES TABLE

REAGENT or RESOURCE	SOURCE	IDENTIFIER
Antibodies		
ABHD6	Protein Tech	RRID:AB_2878694; 20494-1-AP
ACE2	Cell signaling technologies	RRID: AB_2797606; 4355
ATG5	Protein Tech	RRID:AB_2062045; 10181-2-AP
ATG7	ThermoFisher Scientific	RRID: AB_2809507; MA5-32221
Calnexin	Cell Signaling Technologies	RRID: AB_2228381; 2679s
Cathepsin D	Protein Tech	219361
CD-MPR	Developmental Studies Hybridoma Bank	22d4
CI-MPR	Abcam	RRID:AB_10974087; 124767
CI-MPR	Protein Tech	20253-1-AP
CLN5	Abcam	RRID:AB_3662651; ab170899
COXIV	Cell Signaling Technologies	RRID: AB_2797784: 11967S
dsRNA	Millipore Sigma	MABE1134
DRP1	Cell Signaling Technologies	RRID: AB_11178938: 5391S
FLAG	Cell Signaling Technologies	RRID: AB_10950495: 8146T
FLAG (M2)	Millipore Sigma	A8592
GAPDH	Protein Tech	RRID:AB_2737588; 6004–1
Galectin-3	Biolegend	RRID:AB_1134238; 125402
GFP-HRP	Miltenyi Biotec	RRID:AB_247003; 130-091-833
GFP	ThermoFisher Scientific	RRID: AB_2534023: A10262
GM130	Cell signaling Technologies	RRID: AB_3750507: 70767T
LAMP1	Cell Signaling Technologies	RRID: AB_268779: 9091s
LAMP2	Santa Cruz	RRID:AB_626858; SC-18822
LAMTOR4	Cell Signaling Technologies	RRID: AB_2798129: 13140S
LBPA/BMP	Millipore Sigma	RRID:AB_11127192; MABT837
LC3	Santa Cruz	RRID:AB_10714949; sc-271625
mCherry	ThermoFisher Scientific	RRID:AB_2536611; M11217
MIRO1	Millipore Sigma	HPA010687
MIRO2	Protein Tech	RRID:AB2179539; 11237-1-AP
NPC1	Abcam	RRID:AB_ 2734695; 134113
NPC2	Gift from P. Lobel (Rutgers University)	–
PDI	Cell Signaling Technologies	RRID:AB_ 2156433: 3501S
PDH	Abcam	RRID: AB_10862029; ab110333
PLA2G15 (LYPLA3)	Santa Cruz	RRID:AB_10989761; Sc-376078
Rab7a	Cell Signaling Technologies	RRID: AB_1904103: 9367
STX17	Abcam	RRID:AB_10903821; ab316119
TGN46	BioRad	AHP500GT
TOMM20	Santa Cruz	RRID:AB_628381; sc-17764
TOMM20	Cell Signaling Technologies	RRID: AB_2687663: 42406
ULK1	Cell Signaling Technologies	RRID: AB_11178668: 8054S
V5	Protein Tech	RRID:AB_2878059; 14440-1-AP
VPS11	Santa Cruz	RRID:AB_2687986; sc-515094
VPS29	Cell Signaling Technologies	RRID:AB_2799841; 73540
VPS35	Abcam	RRID:AB_296841; Ab10099
VPS39	Santa Cruz	RRID:AB_2687985; SC-514762
VPS41	Santa Cruz	RRID:AB_2687987; SC-377118
Bacterial and virus strains		
SARS-CoV-2	BEI Resources	NR-52281
Deposited data		
Lipidomics	https://dataverse.harvard.edu/dataset.xhtml?persistentId = doi:10.7910/DVN/OQIWA2	–
Experimental models: Cell lines		
A549-hACE2	Gift from Dr. Kajon	–
A549-hACE2	BEI	NR-53821
HeLa WT	ATCC	CCL-2
Vero E6	ATCC	CRL-1586
Oligonucleotides		
siATG5	Horizon discovery	J-004374-07-0002
siATG7	Horizon discovery	L-020112-00-0005
siDRP1	Horizon discovery	L-012092-00-0005
siMIRO1	Horizon discovery	L-010365-01-0005
siMIRO2	Horizon discovery	L-008340-01-0005
siULK1	Horizon discovery	J-005049-05-0002
Recombinant DNA		
ABHD6	This study	NA
CLN5	This study	NA
GFP-E	Addgene	# 165123
GFP-M	Addgene	#165124
Linker peptide	This study	NA
NPC2	Gift from Dr. P. Lobel (Rutgers University)	NA
SARS-CoV2-NSP1-strep	Addgene	#141367
NSP2-strep	Addgene	#141368
NSP3-GFP	Addgene	#165108
NSP4-mch	Addgene	#165132
NSP5-strep	Addgene	#141370
NSP6-mch	Addgene	#165133
GFP- NSP7	Addgene	#165112
NSP8-GFP	Addgene	#165113
NSP9-strep	Addgene	#141375
NSP10-GFP	Addgene	#165115
NSP11-strep	Addgene	#141377
GFP-NSP12	Addgene	#165117
NSP13-mch	Addgene	#165136
NSP14-mch	Addgene	#165137
NSP15-GFP	Addgene	#165120
ORF3a-CoV-2-GFP	This study	NA
ORF3a-CoV-2-V5	This study	NA
CoV-ORF3a	This study	NA
CoV-2-ORF3a-S171A	This study	NA
CoV-2-ORF3a-Y184A	This study	NA
CoV-2-ORF3a-H182A	This study	NA
CoV-2-ORF3a	Previous study	NA
CoV-2-ORF3a-W193A	This study	NA
CoV-2-ORF3a-W193K	This study	NA
CoV-2-ORF3a-W193H	This study	NA
CoV-2-ORF3a-W193D	This study	NA
CoV-2-ORF3a-W193F	This study	NA
ORF3a-CoV-GFP	Addgene	# 165121
ORF3b-strep	Addgene	#141384
ORF6-strep	Addgene	#141387
ORF7a-strep	Addgene	#141388
ORF7b-strep	Addgene	#141389
ORF8-GFP	Addgene	#169395
GFP-ORF9b	Addgene	#165122
ORF10-strep	Addgene	#141394
PLD3	This study	NA
PLD4	This study	NA
Spike	Addgene	#149540
N-strep	Addgene	#141391
TMEM192-HA	Addgene	#102930
VPS39	Previous study	NA
Software and algorithms		
Graph Pad Prism V 9	GraphPad Software	https://www.graphpad.com
ImageJ V 1.8.0	Schneider et al.	https://imagej.nih.gov/ij/
PyMol V 2.4.0.	–	https://www.pymol.org/
ZEN (Zeiss)	Carl Zeiss	–
CellInsight Software	Thermo Fisher	HCS analysis
Other		
Bafilomycin	Milipore Sigma	88899-55-2
CellMask	Thermos fisher	H32722
Chloroquine	Milipore Sigma	50-63-5
Filipin	Milipore Sigma	F9765
Flp-In^™^ Complete System	ThermoFisher	K601001
(S,S) LBPA/BMP	Echelon bioscience	L-B181
Lipofectamine 2000	Thermo Fisher Scientific	11668019
LLOMe	Milipore Sigma	16689-14-8
Monencin	Milipore Sigma	1445481
U18666A	Milipore Sigma	U3633
TransIT-LT1	Mirus Bio	MIR2300
TransIT-X2	Mirus Bio	MIR 600
Critical Instruments		
CellInsight High-Content Imager	Thermo Fisher Scientific	–
Gas Chromatograph-Mass Spectrometer	Agilent	6890/5973
Leica confocal microscope	Leica Microsystems	TCS-SP8
Mass Spectrometer	Thermo Fisher Scientific	TSQ Altis; Q Exactive
Transmission Electron Microscope	Hitachi	HT7700
TriVersa NanoMate	Advion Biosciences	–
Zeiss Confocal Microscopes	Carl Zeiss	LSM800; LSM900

## Highlights

- SARS-CoV-2 protein ORF3a blocks lysosomal cholesterol egress by targeting host VPS39
- The ORF3a-VPS39 interaction traps retromer on endolysosomes, mislocalizing NPC2
- ORF3a-VPS39 disrupts mito-lysosome contacts, impairing PG transport and BMP synthesis
