# Supplementary material for: SARS-CoV-2 ORF3a blocks lysosomal cholesterol egress by disrupting VPS39-regulated NPC2 trafficking and BMP metabolism
Source: Cell Rep. Author manuscript; Available in PMC 2026 Jul 17. (PMC13378056; doi:10.1016/j.celrep.2026.117544)
Supplement: 1 [file NIHMS2190776-supplement-1.pdf]

Cell Reports, Volume *45*

Supplemental information

SARS-CoV-2 ORF3a blocks lysosomal cholesterol egress by disrupting VPS39-regulated NPC2 trafficking and BMP metabolism

Baley A. Goodson, Valeria Montenegro Vazquez, Aliza Doyle, Oralia M. Kolaczkowski, Rui Liu, Jingyue Jia, Morié Ishida, Chunyan Ye, Alison M. Kell, Steven B. Bradfute, Monica Rosas Lemus, Hu Wang, Xianlin Han, and Jing Pu

SUPPLEMENTAL INFORMATION

Supplemental figures

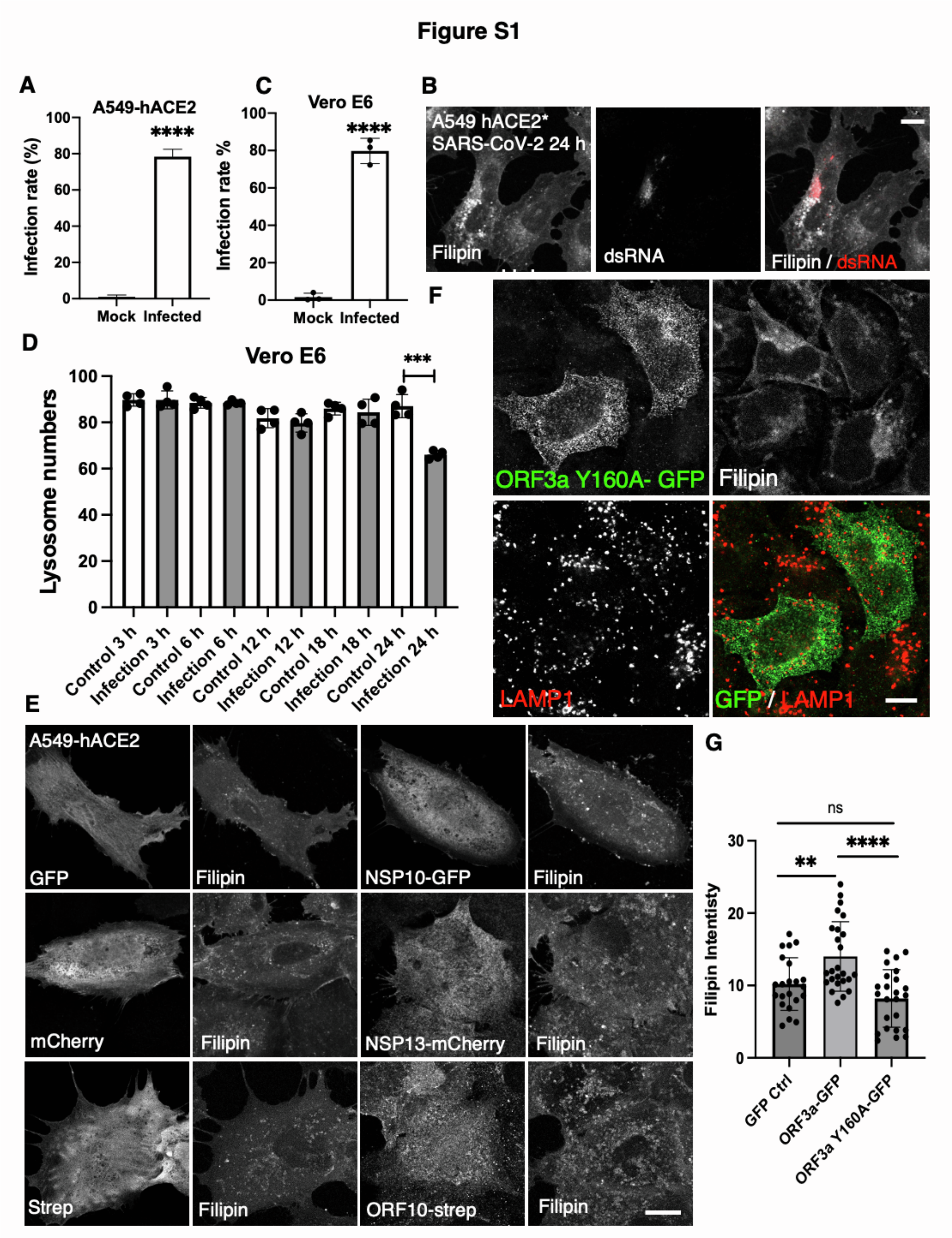

Fig. S1. SARS-CoV-2 infection sequestrates cholesterol in lysosomes

A. A549-hACE2 cells were infected with SARS-CoV-2, and the infection rate was determined by immunostaining of dsRNA and quantified using a high-content imaging system. **B**. An additional line of A549 cells stably expressing human ACE2 (A549-hACE2*) were infected with SARS-CoV-2, fixed at 24 h post-infection, stained with the antibodies against dsRNA and filipin, and imaged with a confocal microscope. Note that the dsRNA-positive cell showed increased filipin intensity, compared to the dsRNA-negative cell. Scale bars, 5 μm. **C.** Vero E6 cells were infected with SARS-CoV-2, and the infection rate was determined by immunostaining of dsRNA and quantified by high-content imaging system. **D.** Vero E6 cells were infected with SARS-CoV-2, fixed, immunostained with a LAMP1 antibody, and analyzed by high-content imaging. **E.** A549-hACE2 cells were transfected with the plasmids that encode NSP10, NSP13, or ORF10 with a different tag. Cells were fixed at 24 h post-transfection, stained with filipin and the antibodies against GFP, mCherry, or strep, and imaged with a confocal microscope. Scale bars, 10 μm. **F, G.** HeLa cells were transfected with SARS-CoV-2 ORF3a-Y160A-GFP, fixed, and stained with GFP and LAMP1 antibodies and Filipin. Imaging was performed using confocal microscopy and total filipin intensity was quantified in GFP positive cells shown in **G.** Bar graphs are presented as mean ± SD. *p* values were e determined using *t* test. **, $p < 0.01$, ***, $p<0.001$. ****, n.s., not significant.

Figure S2

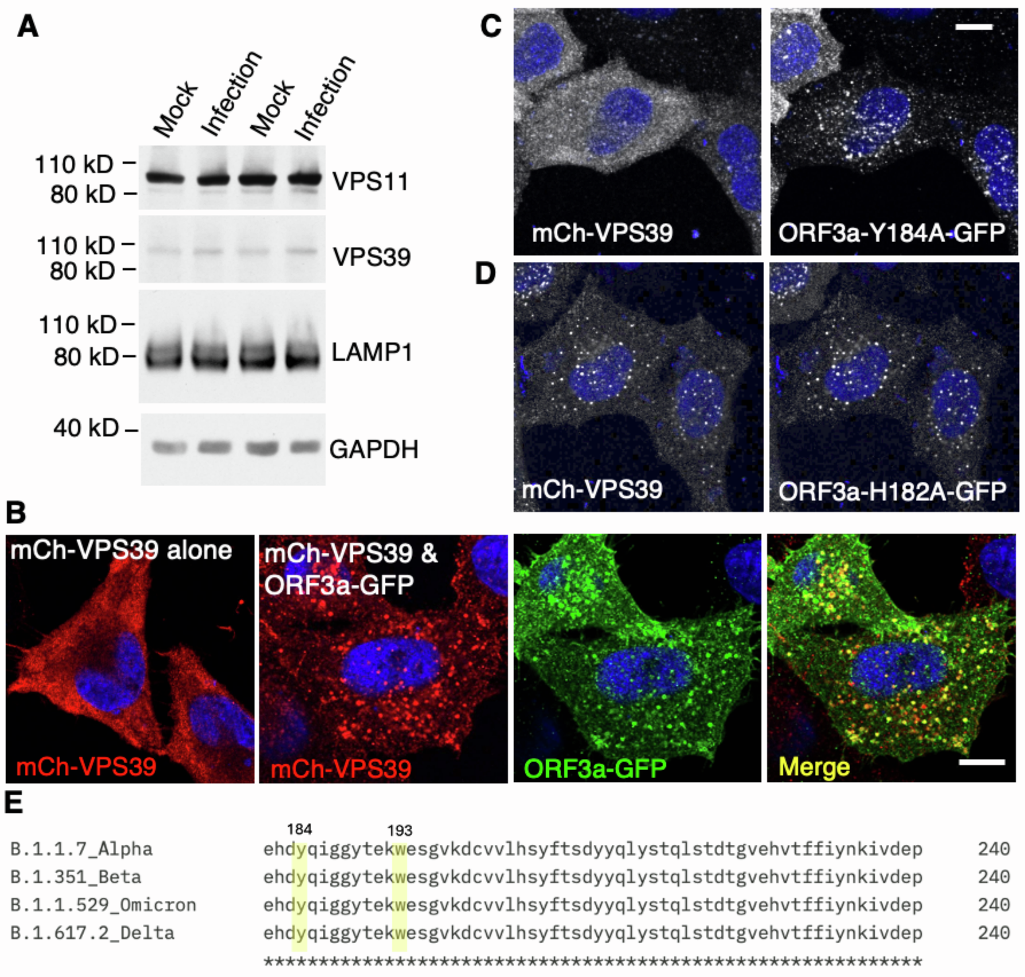

Fig. S2. Characterization of ORF3a-VPS39 interaction

A. A549-hACE2 cells were infected with SARS-CoV-2 and lysed at 24 h post-infection. Cell lysates were subjected to immunoblotting with the indicated antibodies. **B-D.** HeLa cells were transfected with mCh-VPS39 construct alone (**B, left**) or co-transfected with ORF3a-GFP (**B, right**) or ORF3a mutant constructs (**C, D**). The cells were fixed at 24 h post transfection, immunostained with the antibodies against GFP and mCherry, and imaged with a confocal microscope. Note that mCh-VPS39 alone displayed cytosolic distribution (**B, left**). When ORF3a was present, mCh-VPS39 formed puncta, colocalized with ORF3a (**B, right**). This distribution alteration was used as an indicator of ORF3a-VPS39 interaction in examining those ORF3a mutants (**C, D**). Scale bars, 5 μm. **E.** Amino acid sequences of ORF3a from four SARS-CoV-2 variants were aligned using Clustal Omega. The VPS39 binding sites, 184 and 193 residues, are highlighted.

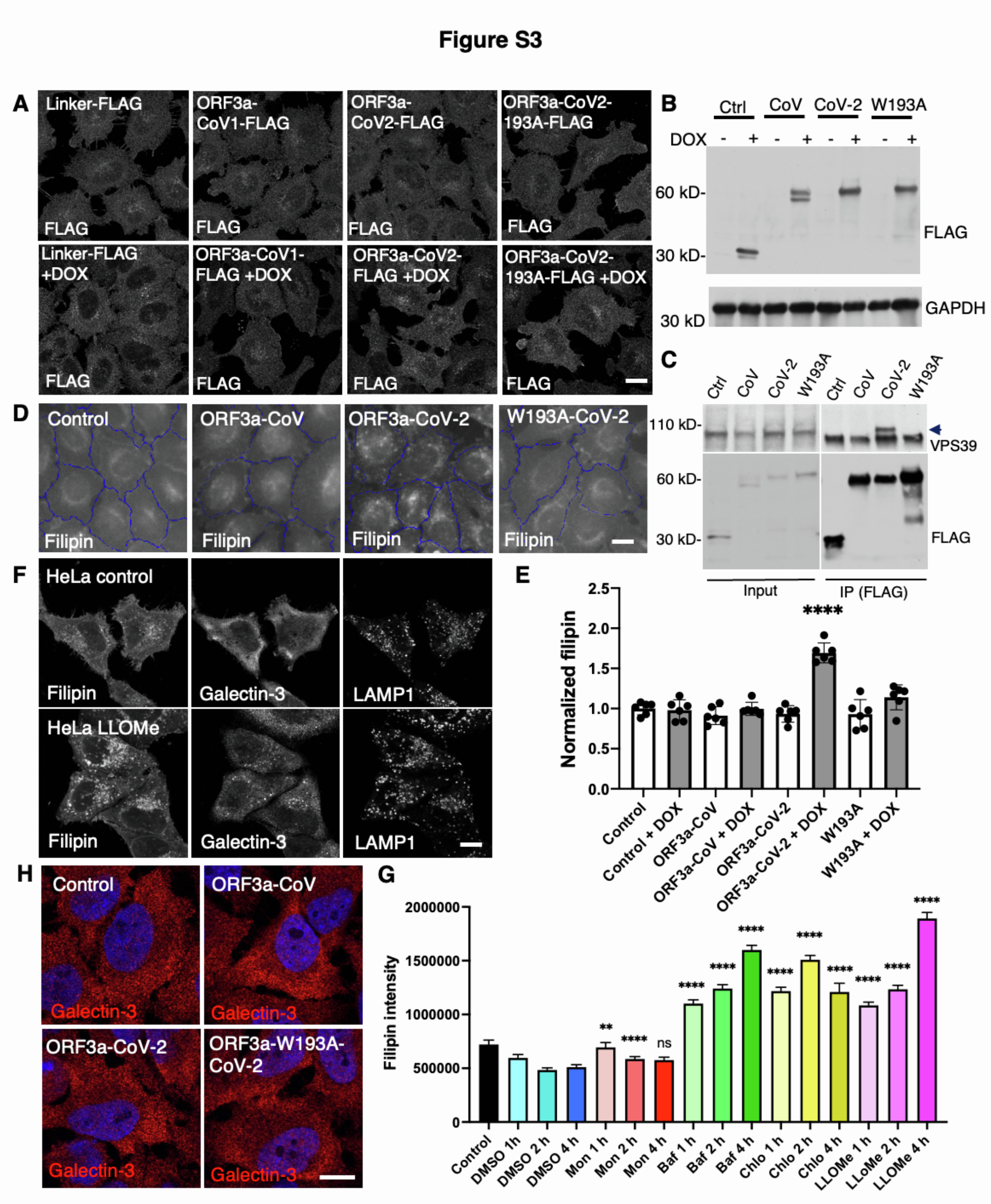

Fig. S3. The impacts of ORF3a-VPS39 interaction on lysosome integrity

A, B. HeLa Flp-In cells were treated with or without doxycycline (DOX), fixed, stained with a FLAG antibody, and imaged with a confocal microscope (**A**) or lysed for western blots with the indicated antibodies (**B**). **C.** Interaction between ORF3a and endogenous VPS39 was assessed by co-immunoprecipitation and immunoblotting. **D.** Cells were

analyzed for free cholesterol distribution using filipin staining and high-content imaging**. E.** The Flp-In cells were treated with or without doxycycline, fixed, stained with filipin, and analyzed by high-content imaging for filipin signals. **F.** HeLa cells were treated with 0.5 mM LLOMe for 1 h, fixed, stained with filipin and a galectin-3 antibody, and imaged with a confocal microscope. **G.** HeLa cells were treated with 10 μM monensin, 100 nM bafilomycin, 100 μM chloroquine, or 0.5 mM LLOMe for the indicated time periods, fixed, stained with filipin, and analyzed with high-content imaging. 0.1% DMSO in cell culture media served as a vehicle control. **H.** Example confocal microscopy images of HeLa Flp-In cells, immunostained with a galectin-3 antibody. Bar graphs are presented as mean ± SD. *p* values were determined using one-way ANOVA. **, $p < 0$.

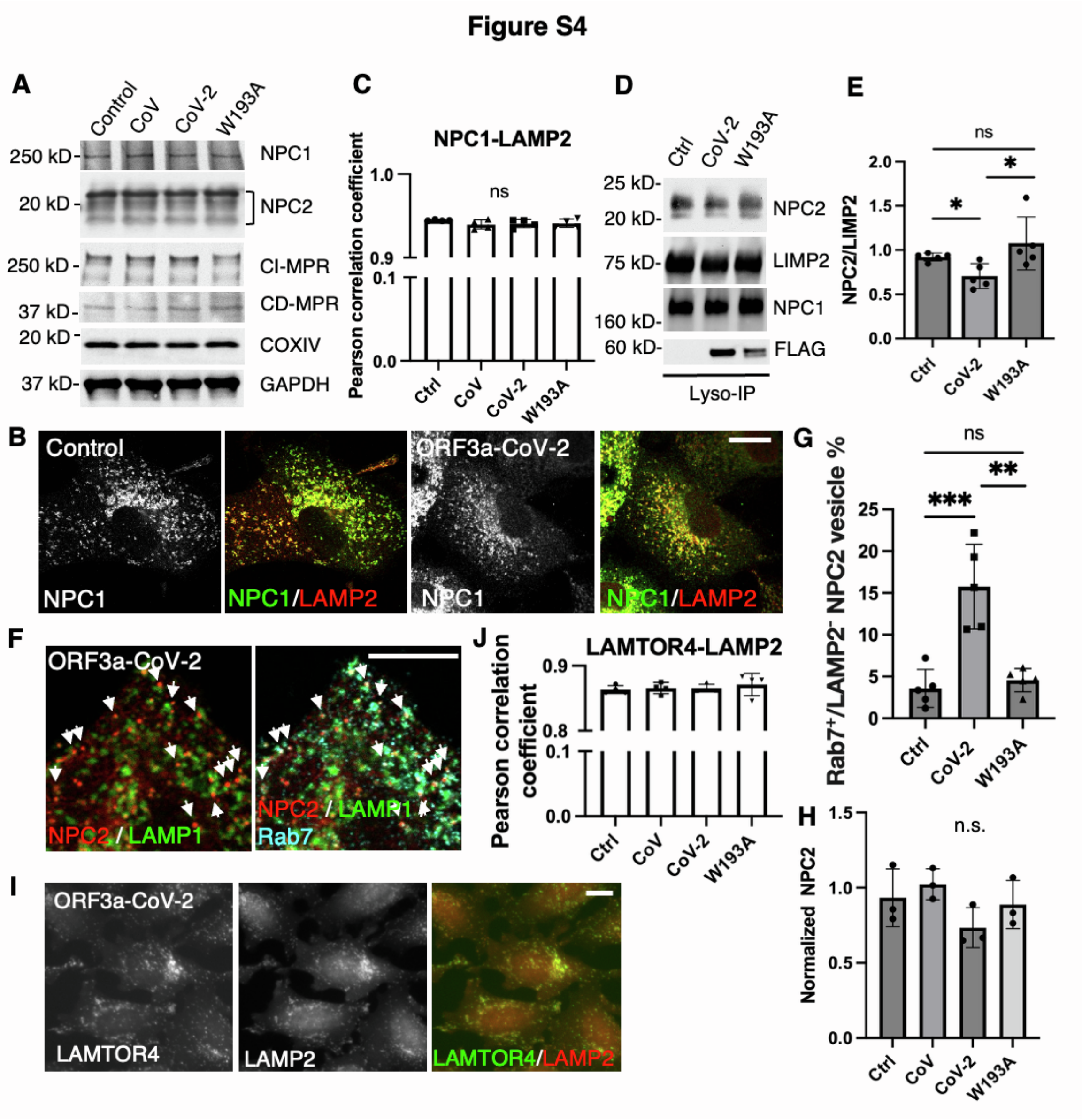

01. ****, $p < 0.0001$. n.s., no significant difference. Scale bars, 5 μm.

Fig. S4. Characterization and localization of lysosomal proteins

- **A.** Cell lysates from HeLa Flp-In cells were subjected to immunoblotting with the indicated antibodies. **B, C.** HeLa Flp-In cells were fixed, immunostained with the indicated antibodies and imaged by confocal microscopy (**B**) or analyzed by high-content imaging system (**C**). **D, E.** Lyso-IP was performed among the Flp-In cell lines, and immunoblotting was performed with the indicated antibodies (**D**). Quantification was based on 5 independent experiments. **F, G**. NPC2 vesicles were characterized with their overlap with LAMP2 and/or Rab7. Five cells from each cell line were quantified. **H.** Densitometric quantification of NPC2 protein

levels was normalized to the loading control and expressed relative to control cells. **I, J.** HeLa Flp-In cells were immunostained with LAMTOR4 and LAMP2 and analyzed by high-content imaging. The colocalization between LAMTOR4 and LAMP2 was quantified and is presented as Pearson colocalization coefficient**.** Scale bars, 5 μm. Bar graphs are presented as mean ± SD. *p* values were determined using one-way ANOVA test. *, $p < 0.05$. **** and ####, $p < 0.0001$. n.s., no significant difference.

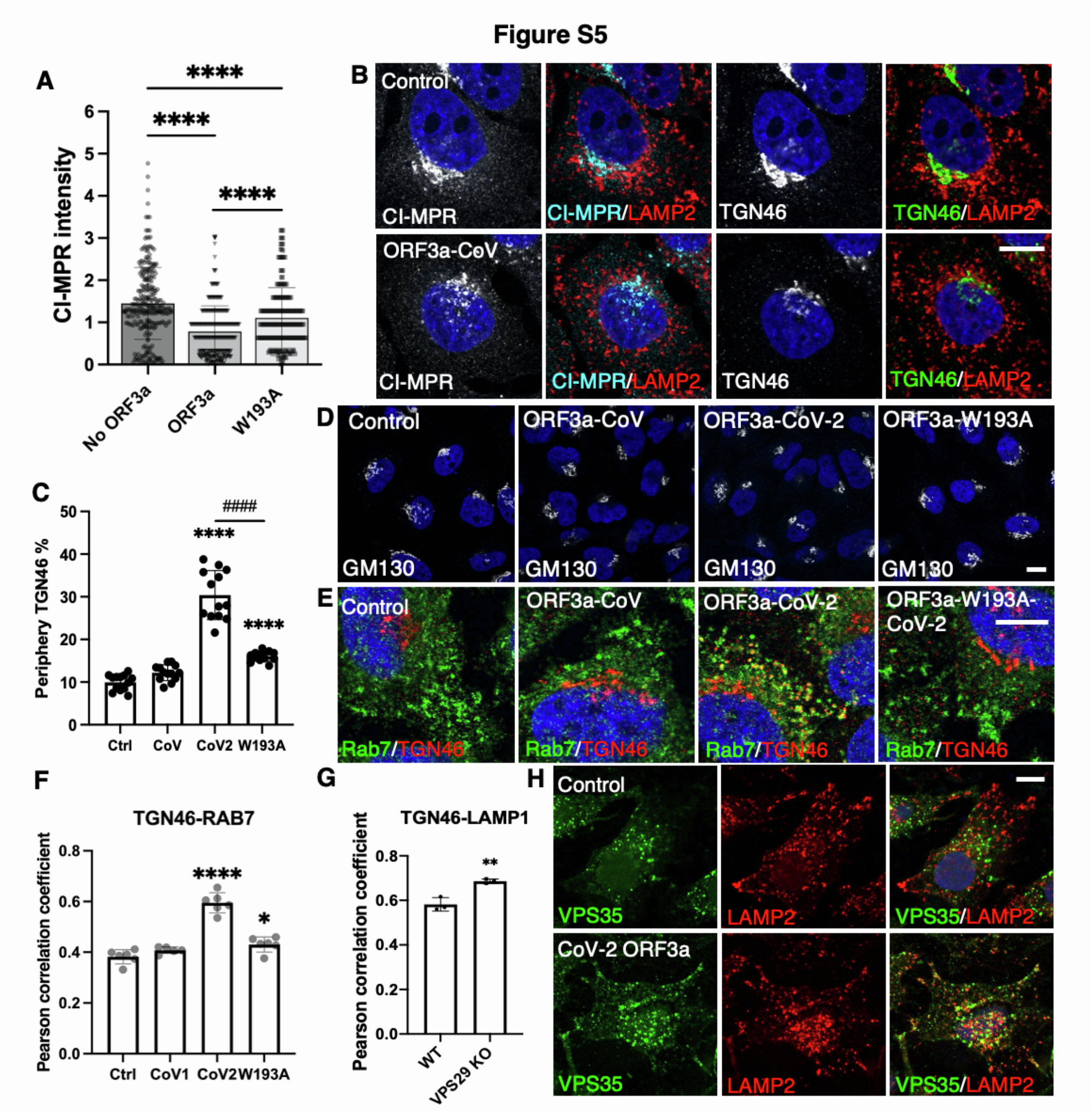

Fig S5. Characterization of TGN protein localization

A. HeLa cells were transfected with the constructs encoding ORF3a-GFP or W193A mutant and stained with CI-MPR and GFP antibodies. CI-MPR intensity was quantified in the transfected cells using high-content imaging system. Approximately 300 cells from each condition were analyzed. **B, C.** The indicated cells were analyzed for protein localization within cell peripheral areas, defined as 15-pixel distance ring area shrunk from cell boundaries. (**B**) and high-content imaging (**C**). Periphery was defined as a ring area shrunk from cell boundary with a gap distance 15 pixels**. D.** Flp-In cells were fixed and immunostained for GM130. **E, F**. Flp-In cells were fixed and immunostained with Rab7 and TGN46 antibodies. Images were taken either with a confocal microscope (**E**) or high-

content imaging system (**F**) for quantification of the colocalization between TGN46 and Rab7. **G.** HeLa and VPS29-KO cells were analyzed for the colocalization between TGN46 and LAMP1. **H**. The indicated cells were fixed and immunostained with indicated antibodies. Scale bars, 5 μm. Bar graphs are presented as mean ± SD. *p* values were determined using one-way ANOVA test. **, $p < 0.01$. **** and ####, $p < 0.0001$.

Figure S6

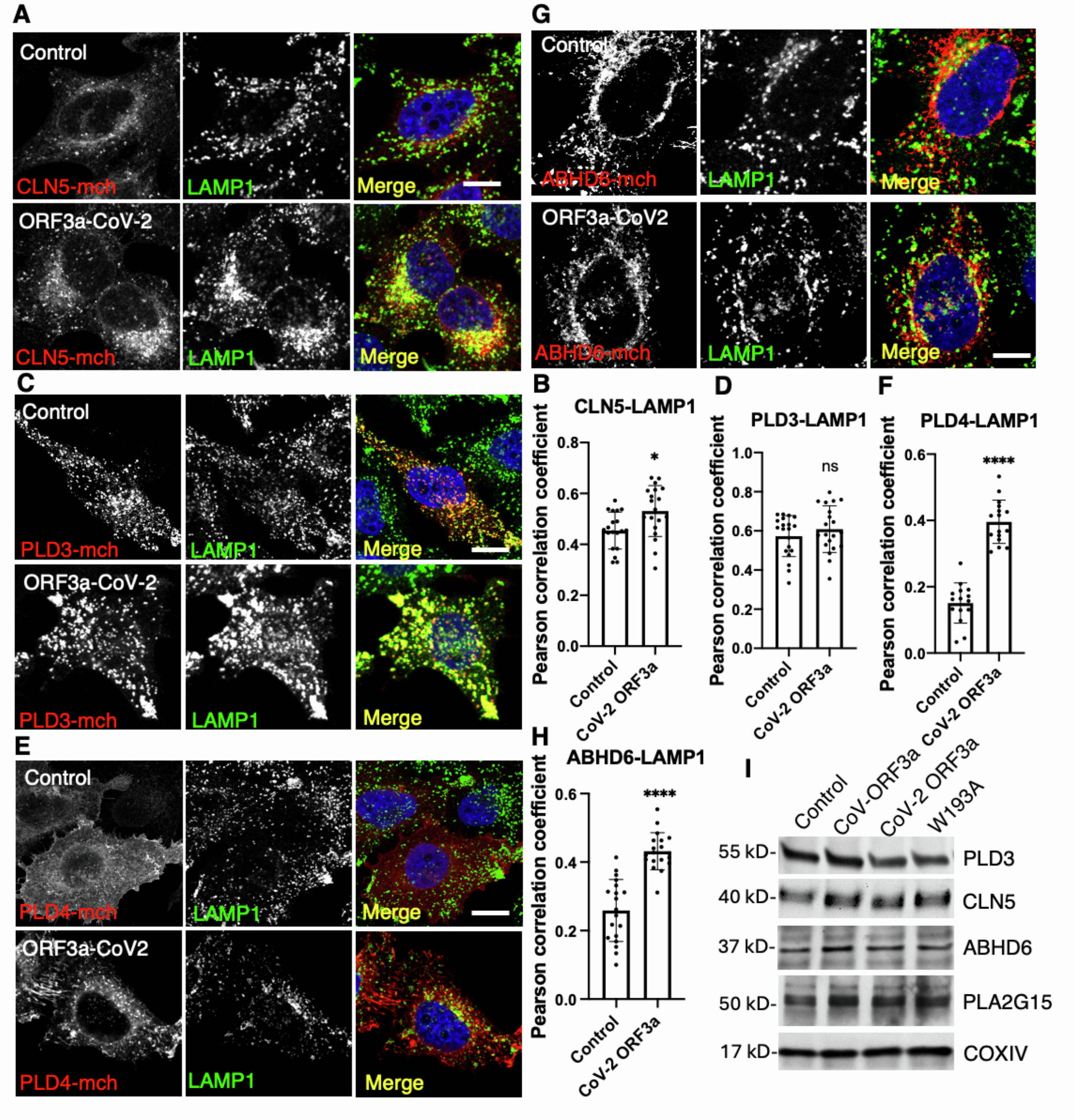

Fig. S6. Characterization of BMP enzymes

HeLa Flp-In control and CoV-2-ORF3a cells were transfected with the plasmids encoding BMP synthesis or turnover enzymes, fixed at 24-h post-transfection, immunostained with mCherry and LAMP1 antibodies. Images were taken with a confocal microscope **(A, C, E, G)** and used for quantification of the colocalization between the enzymes and LAMP1 by FIJI **(B, D, F, H).** Scale bar, 5 µm. **I.** Cell lysates from HeLa Flp-In cells were subjected to immunoblotting with the indicated antibodies. Bar graphs are presented as mean ± SD. *p* values were determined using *t* test. *, $p < 0.05$. ****, $p < 0.0001$. n.s., no significant difference.

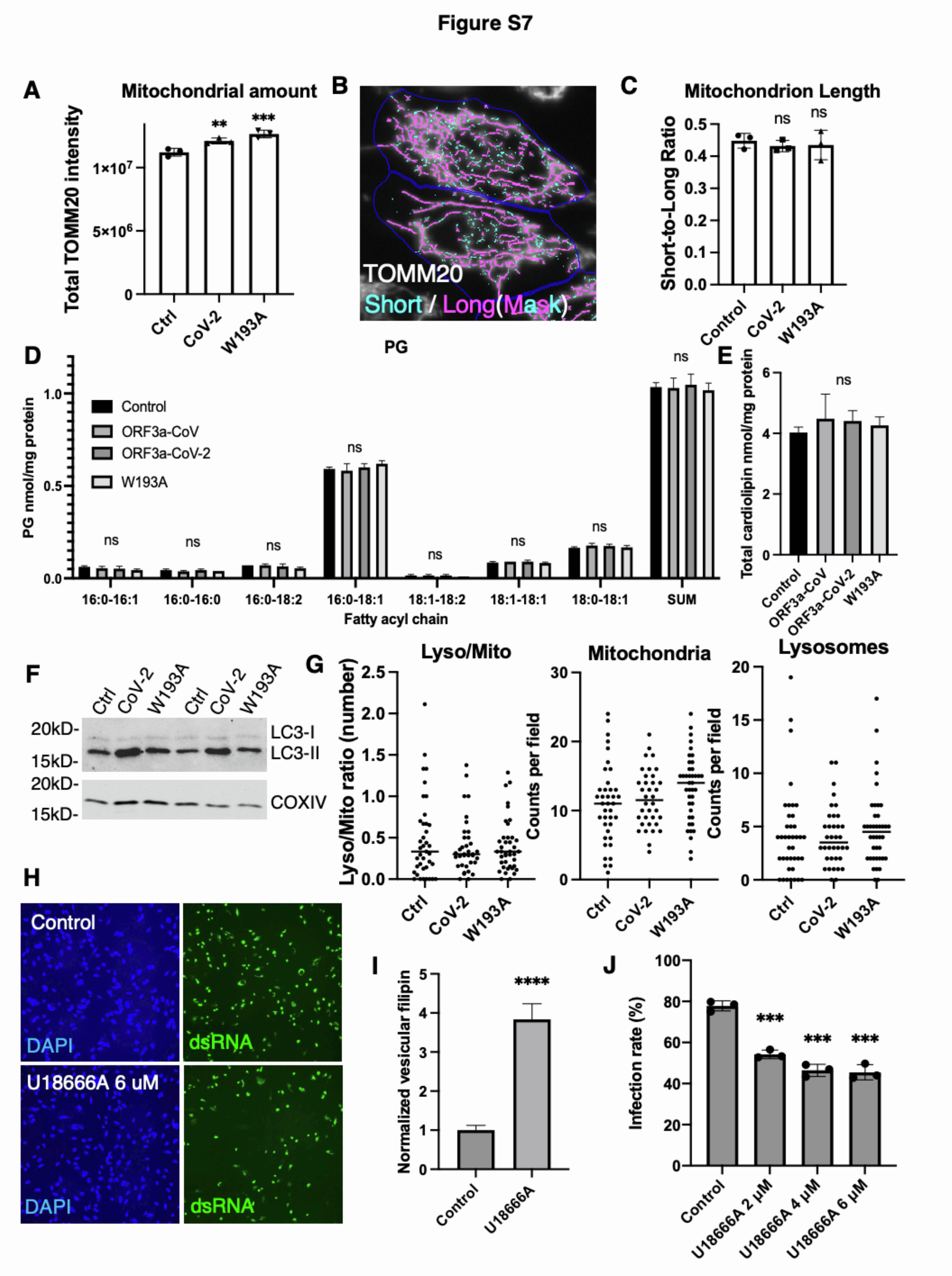

Fig. S7. Mitochondrial quantifications and PG metabolism measurement

A-C. HeLa Flp-In cells were fixed, immunostained with a TOMM20 antibody, and analyzed with high-content imaging. Average total intensity of TOMM20 (**A**) and the

short-to-long ratio of mitochondria (**C**) were quantified. Short mitochondria were defined as those with a length shorter than 1 μm (**B**). **D, E.** Total lipids were extracted from the indicated cells and subjected to shotgun lipidomics analysis. PGs (**D**) and total cardiolipin (**E**) are presented as mean± SD (n=4). **F.** Cell lysates from HeLa Flp-In cells were subjected to immunoblotting with the indicated antibodies. **G.** HeLa Flp-In cells were fixed and imaged by an electron microscope. The numbers of lysosomes and mitochondria were quantified from 40 images of 20 cells of each cell type. **I**. A549-hACE2 cells were treated with 2 μM U18666A for 4 h, fixed, stained with filipin, and quantified for filipin signals with high-content imaging. **H, J.** A549-hACE2 cells were treated with U18666A at the indicated concentration for 4 h and infected with SARS-CoV-2. Control cells were treated with the same amount of DMSO as in 6 μM U18666A for 4 h and infected equally. Cells were fixed at 24 h post-infection and immunostained with a dsRNA antibody to identify the infected cells. DAPI-stained nuclei were used to identify total cell numbers. *p* values were determined using One-way ANOVA test. **, $p<0.01$. ***, $p<0.001$. ****, $p<0.0001$. n.s., not significant.
