# Supplementary material for: SARS-CoV-2 ORF3a blocks lysosomal cholesterol egress by disrupting VPS39-regulated NPC2 trafficking and BMP metabolism
Source: Cell Rep. Author manuscript; Available in PMC 2026 Jul 17. (PMC13378056; doi:10.1016/j.celrep.2026.117544)
Supplement: 2 [file NIHMS2190776-supplement-2.pdf]

Gene Symbol	Unique 1-linker-lyso-IP	Total 1-linker-lyso-IP	Unique 2-3a-lyso-IP	Total 2-3a-lyso-IP	Unique 3-193-lyso-IP	Total 3-193-lyso-IP	Sum Intensity 1-linker-lyso-IP	Sum Intensity 2-3a-lyso-IP	Sum Intensity 3-193-lyso-IP	lowest value	1-linker-lyso-IP	2-3a-lyso-IP	3-193-lyso-IP
PLEC	258	497	238	469	220	381	2.40E+09	1.90E+09	1.10E+09	1.10E+09	2.2	1.7	1.0
AHNAK	95	99	121	132	83	91	1.30E+08	2.10E+08	7.80E+07	7.80E+07	1.7	2.7	1.0
SPTAN1	93	118	86	101	74	83	1.60E+08	1.30E+08	7.10E+07	7.10E+07	2.3	1.8	1.0
CPS1	93	354	80	330	77	369	4.70E+09	2.60E+09	2.70E+09	2.60E+09	1.8	1.0	1.0
LRPPRC	79	157	78	150	75	144	8.00E+08	6.30E+08	4.10E+08	4.10E+08	2.0	1.5	1.0
SPTBN1	67	79	69	88	48	62	1.90E+08	1.60E+08	6.90E+07	6.90E+07	2.8	2.3	1.0
PRKDC	64	65	57	67	52	56	6.20E+07	6.10E+07	3.30E+07	3.30E+07	1.9	1.8	1.0
HSPD1	67	409	62	342	60	368	5.70E+09	3.90E+09	3.50E+09	3.50E+09	1.6	1.1	1.0
FLNA	53	68	52	70	47	66	1.60E+08	1.70E+08	8.20E+07	8.20E+07	2.0	2.1	1.0
VIM	52	255	49	281	44	195	4.10E+09	3.70E+09	1.50E+09	1.50E+09	2.7	2.5	1.0
MYOF	46	48	44	47	38	40	6.80E+07	5.60E+07	3.70E+07	3.70E+07	1.8	1.5	1.0
ACTN4	47	99	52	117	43	84	7.80E+08	7.70E+08	3.60E+08	3.60E+08	2.2	2.1	1.0
CLTC	44	55	40	49	34	43	1.20E+08	9.20E+07	6.10E+07	6.10E+07	2.0	1.5	1.0
HSP90B1	38	63	32	51	35	54	2.90E+08	2.10E+08	1.60E+08	1.60E+08	1.8	1.3	1.0
HSPA9	39	111	39	111	37	107	9.40E+08	7.30E+08	5.70E+08	5.70E+08	1.6	1.3	1.0
IQGAP1	32	37	36	43	32	37	2.60E+07	3.30E+07	1.80E+07	1.80E+07	1.4	1.8	1.0
MYH9	25	26	41	50	24	28	2.20E+07	4.20E+07	1.40E+07	1.40E+07	1.6	3.0	1.0
EEA1	29	33	34	38	34	35	6.30E+07	6.30E+07	5.50E+07	5.50E+07	1.1	1.1	1.0
ATP5F1B	42	124	31	95	29	106	7.20E+08	4.10E+08	4.50E+08	4.10E+08	1.8	1.0	1.1
DYNC1H1	29	30	32	34	23	24	9.30E+06	1.80E+07	5.30E+06	5.30E+06	1.8	3.4	1.0
GANAB	36	45	29	43	28	41	1.20E+08	7.70E+07	6.90E+07	6.90E+07	1.7	1.1	1.0
LONP1	30	42	24	33	28	42	9.10E+07	6.40E+07	5.60E+07	5.60E+07	1.6	1.1	1.0
FLNB	24	25	29	31	19	20	1.50E+07	2.00E+07	6.00E+06	6.00E+06	2.5	3.3	1.0
ATP5F1A	32	92	31	94	28	102	8.60E+08	5.70E+08	4.60E+08	4.60E+08	1.9	1.2	1.0
DHX30	31	34	23	27	22	30	4.50E+07	3.80E+07	2.30E+07	2.30E+07	2.0	1.7	1.0
TFRC	31	48	26	39	28	43	3.20E+08	2.20E+08	1.60E+08	1.60E+08	2.0	1.4	1.0
ALDH18A1	26	43	27	55	26	49	1.20E+08	1.10E+08	7.80E+07	7.80E+07	1.5	1.4	1.0
HSPA8	29	46	26	48	25	49	1.20E+08	1.10E+08	1.00E+08	1.00E+08	1.2	1.1	1.0
NNT	32	44	25	34	26	36	1.40E+08	8.90E+07	6.80E+07	6.80E+07	2.1	1.3	1.0
IMMT	28	44	22	36	21	31	1.20E+08	9.50E+07	6.10E+07	6.10E+07	2.0	1.6	1.0
HYOU1	25	29	22	27	26	29	3.60E+07	2.20E+07	1.90E+07	1.90E+07	1.9	1.2	1.0
SLC3A2	26	51	28	53	25	45	6.80E+08	4.20E+08	2.90E+08	2.90E+08	2.3	1.4	1.0
TRAP1	24	40	23	36	23	39	2.10E+08	1.70E+08	1.50E+08	1.50E+08	1.4	1.1	1.0
HADHA	30	42	25	33	25	31	9.40E+07	4.40E+07	5.50E+07	4.40E+07	2.1	1.0	1.3
HSPA5	28	48	27	48	27	54	2.20E+08	1.80E+08	1.60E+08	1.60E+08	1.4	1.1	1.0
TLN1	20	20	27	31	20	21	1.20E+07	1.30E+07	4.90E+06	4.90E+06	2.4	2.7	1.0
SPTBN2	20	23	25	28	15	17	2.10E+07	2.40E+07	9.90E+06	9.90E+06	2.1	2.4	1.0
HDLBP	23	27	19	25	18	22	2.10E+07	1.70E+07	1.20E+07	1.20E+07	1.8	1.4	1.0
POLRMT	23	28	16	18	20	26	5.40E+07	4.10E+07	3.00E+07	3.00E+07	1.8	1.4	1.0
ATP1A1	24	32	24	31	21	31	1.00E+08	9.20E+07	6.40E+07	6.40E+07	1.6	1.4	1.0
ACO2	20	22	22	27	22	25	3.70E+07	3.00E+07	2.30E+07	2.30E+07	1.6	1.3	1.0
CTNNA1	17	21	23	29	23	28	1.80E+07	1.90E+07	1.00E+07	1.00E+07	1.8	1.9	1.0
AP2B1	23	26	20	25	21	23	2.50E+07	2.80E+07	1.70E+07	1.70E+07	1.5	1.6	1.0
ACTN1	22	27	21	29	13	15	3.60E+07	3.30E+07	1.10E+07	1.10E+07	3.3	3.0	1.0

OGDH	25	30	19	19	17	22	3.60E+07	2.20E+07	1.30E+07	1.30E+07	2.8	1.7	1.0
PNPT1	23	25	20	20	19	21	2.90E+07	1.90E+07	1.20E+07	1.20E+07	2.4	1.6	1.0
LETM1	20	27	22	36	20	30	5.00E+07	3.50E+07	2.70E+07	2.70E+07	1.9	1.3	1.0
KTN1	21	21	18	22	19	21	2.40E+07	2.20E+07	1.80E+07	1.80E+07	1.3	1.2	1.0
MYO1C	19	20	20	22	18	20	2.30E+07	2.80E+07	1.70E+07	1.70E+07	1.4	1.6	1.0
ACTB	23	172	25	193	21	150	2.90E+09	2.20E+09	1.30E+09	1.30E+09	2.2	1.7	1.0
PDIA3	19	29	20	34	21	30	9.60E+07	7.30E+07	5.20E+07	5.20E+07	1.8	1.4	1.0
HSP90AA1	21	29	19	29	16	26	1.30E+08	1.10E+08	8.70E+07	8.70E+07	1.5	1.3	1.0
SDHA	22	36	13	22	13	19	8.00E+07	5.80E+07	4.40E+07	4.40E+07	1.8	1.3	1.0
RRBP1	23	28	19	21	18	18	2.10E+07	1.60E+07	1.10E+07	1.10E+07	1.9	1.5	1.0
IARS2	24	26	18	19	17	18	4.60E+07	2.70E+07	2.00E+07	2.00E+07	2.3	1.4	1.0
CKAP4	22	23	16	19	15	16	3.90E+07	2.10E+07	1.70E+07	1.70E+07	2.3	1.2	1.0
SCRIB	16	17	19	21	17	19	1.10E+07	1.10E+07	6.30E+06	6.30E+06	1.7	1.7	1.0
LIMA1	20	28	20	27	20	30	7.10E+07	5.20E+07	3.60E+07	3.60E+07	2.0	1.4	1.0
GPD2	20	26	18	23	17	22	1.80E+07	1.80E+07	8.90E+06	8.90E+06	2.0	2.0	1.0
GLUD1	22	28	15	20	18	23	6.50E+07	4.20E+07	3.60E+07	3.60E+07	1.8	1.2	1.0
LMNA	19	22	15	19	20	21	2.70E+07	1.90E+07	1.40E+07	1.40E+07	1.9	1.4	1.0
EPRS1	15	16	16	18	16	18	7.50E+06	8.60E+06	7.70E+06	7.50E+06	1.0	1.1	1.0
OXCT1	19	38	20	35	15	36	1.10E+08	6.30E+07	5.50E+07	5.50E+07	2.0	1.1	1.0
GALNT2	19	32	15	23	17	31	4.20E+07	1.40E+07	1.70E+07	1.40E+07	3.0	1.0	1.2
VCP	19	22	19	23	18	20	4.40E+07	2.60E+07	1.70E+07	1.70E+07	2.6	1.5	1.0
COPA	20	23	15	20	14	18	1.40E+07	9.80E+06	7.70E+06	7.70E+06	1.8	1.3	1.0
SND1	20	25	16	17	15	16	2.40E+07	2.00E+07	1.20E+07	1.20E+07	2.0	1.7	1.0
OPA1	18	20	17	20	13	15	1.70E+07	1.40E+07	6.40E+06	6.40E+06	2.7	2.2	1.0
TUFM	21	39	19	36	17	39	2.10E+08	1.20E+08	1.00E+08	1.00E+08	2.1	1.2	1.0
PDIA4	18	23	16	19	17	22	2.80E+07	1.60E+07	4.00E+07	1.60E+07	1.8	1.0	2.5
DHX9	19	22	17	22	12	14	1.70E+07	2.30E+07	7.30E+06	7.30E+06	2.3	3.2	1.0
DSG2	18	22	14	19	15	16	2.70E+07	1.90E+07	1.00E+07	1.00E+07	2.7	1.9	1.0
EIF3A	17	21	13	17	13	17	3.40E+07	1.20E+07	7.10E+06	7.10E+06	4.8	1.7	1.0
HSD17B4	19	22	10	12	15	18	2.20E+07	1.00E+07	1.20E+07	1.00E+07	2.2	1.0	1.2
MYO1B	16	17	16	18	14	14	1.60E+07	1.40E+07	7.50E+06	7.50E+06	2.1	1.9	1.0
SLC25A13	17	24	18	24	18	26	5.40E+07	5.10E+07	3.60E+07	3.60E+07	1.5	1.4	1.0
MTHFD1L	20	25	17	22	17	22	6.40E+07	4.20E+07	3.60E+07	3.60E+07	1.8	1.2	1.0
PC	18	22	10	14	13	15	2.00E+07	1.40E+07	1.10E+07	1.10E+07	1.8	1.3	1.0
AFG3L2	18	18	12	14	14	14	2.90E+07	2.80E+07	1.80E+07	1.80E+07	1.6	1.6	1.0
EPB41L2	16	16	11	14	13	14	1.40E+07	1.00E+07	7.70E+06	7.70E+06	1.8	1.3	1.0
SLC25A5	15	48	15	50	12	45	1.20E+09	8.20E+08	6.90E+08	6.90E+08	1.7	1.2	1.0
POR	17	29	12	20	13	23	3.20E+07	2.40E+07	1.90E+07	1.90E+07	1.7	1.3	1.0
LMNB1	17	23	18	23	18	20	2.50E+07	2.20E+07	1.40E+07	1.40E+07	1.8	1.6	1.0
RPN2	19	25	14	21	12	17	3.50E+07	1.60E+07	1.20E+07	1.20E+07	2.9	1.3	1.0
EPPK1	15	18	15	24	14	17	8.10E+07	9.70E+07	5.20E+07	5.20E+07	1.6	1.9	1.0
CLPX	17	20	15	18	15	15	2.40E+07	2.20E+07	1.60E+07	1.60E+07	1.5	1.4	1.0
FASN	11	12	17	20	12	15	6.30E+06	1.00E+07	3.50E+06	3.50E+06	1.8	2.9	1.0
CNOT1	11	12	18	20	12	12	4.40E+06	6.60E+06	2.10E+06	2.10E+06	2.1	3.1	1.0
ABCC1	14	14	11	11	9	10	1.40E+07	7.70E+06	3.10E+06	3.10E+06	4.5	2.5	1.0
PHB2	15	26	18	28	17	26	2.40E+08	1.50E+08	1.10E+08	1.10E+08	2.2	1.4	1.0
ATP2A2	15	21	17	24	15	25	2.60E+07	2.00E+07	1.40E+07	1.40E+07	1.9	1.4	1.0
IPO5	17	22	14	17	16	25	1.20E+07	6.70E+06	4.70E+06	4.70E+06	2.6	1.4	1.0
CS	15	26	10	19	8	15	8.70E+07	9.80E+07	8.90E+07	8.70E+07	1.0	1.1	1.0
CTNNB1	14	19	15	21	15	19	1.50E+07	1.70E+07	8.50E+06	8.50E+06	1.8	2.0	1.0
ESYT1	16	18	14	15	13	14	9.80E+06	7.60E+06	5.30E+06	5.30E+06	1.8	1.4	1.0

LARS1	17	19	11	11	12	13	8.40E+06	7.20E+06	6.20E+06	6.20E+06	1.4	1.2	1.0
GOLGB1	13	15	10	11	14	14	1.30E+07	4.20E+06	4.80E+06	4.20E+06	3.1	1.0	1.1
GTF2I	9	10	13	13	16	16	1.20E+07	1.50E+07	1.30E+07	1.20E+07	1.0	1.3	1.1
COL12A1	7	7	9	9	13	14	2.00E+06	2.60E+06	3.10E+06	2.00E+06	1.0	1.3	1.6
SERPINH1	14	22	13	16	14	18	4.60E+07	2.90E+07	2.20E+07	2.20E+07	2.1	1.3	1.0
ACOT9	15	17	11	17	12	17	1.30E+07	1.20E+07	8.00E+06	8.00E+06	1.6	1.5	1.0
CPT1A	17	20	9	9	11	13	2.60E+07	9.70E+06	1.20E+07	9.70E+06	2.7	1.0	1.2
PRPF8	9	10	15	17	10	12	2.80E+06	3.50E+06	1.40E+06	1.40E+06	2.0	2.5	1.0
XRCC6	16	17	10	10	9	11	1.30E+07	9.00E+06	3.80E+06	3.80E+06	3.4	2.4	1.0
RANBP2	8	9	16	17	9	10	2.90E+06	4.70E+06	1.80E+06	1.80E+06	1.6	2.6	1.0
MDH2	17	57	17	60	17	62	2.70E+08	2.00E+08	1.60E+08	1.60E+08	1.7	1.3	1.0
SLC25A3	14	40	15	37	15	45	2.80E+08	2.30E+08	2.30E+08	2.30E+08	1.2	1.0	1.0
CANX	14	22	13	26	13	25	9.10E+07	6.60E+07	4.50E+07	4.50E+07	2.0	1.5	1.0
FH	16	23	14	22	12	21	3.80E+07	3.20E+07	3.10E+07	3.10E+07	1.2	1.0	1.0
AIFM1	15	24	15	21	13	21	4.30E+07	3.30E+07	2.30E+07	2.30E+07	1.9	1.4	1.0
ATAD3B	15	19	13	19	11	20	1.10E+08	7.90E+07	7.10E+07	7.10E+07	1.5	1.1	1.0
HSPA1A	13	16	13	22	10	17	5.50E+07	7.40E+07	4.60E+07	4.60E+07	1.2	1.6	1.0
RPN1	16	18	11	15	13	17	3.70E+07	3.10E+07	1.90E+07	1.90E+07	1.9	1.6	1.0
TOMM70	14	16	11	18	10	15	2.90E+07	3.10E+07	2.60E+07	2.60E+07	1.1	1.2	1.0
ACADVL	16	17	13	16	12	13	2.90E+07	2.20E+07	1.70E+07	1.70E+07	1.7	1.3	1.0
MCCC2	17	17	13	14	13	14	1.30E+07	6.90E+06	6.20E+06	6.20E+06	2.1	1.1	1.0
DARS2	14	16	14	16	10	13	1.60E+07	9.70E+06	6.60E+06	6.60E+06	2.4	1.5	1.0
EMC1	16	17	12	13	11	13	1.10E+07	6.20E+06	4.80E+06	4.80E+06	2.3	1.3	1.0
HADHB	13	13	10	12	13	15	3.00E+07	2.00E+07	1.90E+07	1.90E+07	1.6	1.1	1.0
CKAP5	11	11	15	15	10	11	5.90E+06	8.20E+06	3.50E+06	3.50E+06	1.7	2.3	1.0
XRCC5	12	12	12	12	10	12	1.00E+07	8.40E+06	3.60E+06	3.60E+06	2.8	2.3	1.0
DNAJC13	11	12	10	10	10	12	3.50E+06	2.50E+06	2.20E+06	2.20E+06	1.6	1.1	1.0
FLOT1	16	17	9	9	8	8	1.30E+07	5.60E+06	3.60E+06	3.60E+06	3.6	1.6	1.0
COL7A1	9	9	6	6	15	15	6.50E+06	4.60E+06	4.70E+06	4.60E+06	1.4	1.0	1.0
MAN2A1	12	12	8	9	8	8	6.90E+06	5.50E+06	3.70E+06	3.70E+06	1.9	1.5	1.0
VDAC1	16	34	15	27	14	28	3.80E+08	2.00E+08	1.90E+08	1.90E+08	2.0	1.1	1.0
PDHB	13	27	13	31	13	29	5.10E+07	2.80E+07	2.20E+07	2.20E+07	2.3	1.3	1.0
CALR	13	21	12	20	10	21	9.20E+07	4.80E+07	4.70E+07	4.70E+07	2.0	1.0	1.0
NDUFS1	14	16	15	18	12	19	1.70E+07	1.40E+07	1.30E+07	1.30E+07	1.3	1.1	1.0
SLC25A12	14	16	14	18	15	19	3.40E+07	3.40E+07	2.80E+07	2.80E+07	1.2	1.2	1.0
ANXA2P2	13	15	15	21	12	15	5.20E+07	8.20E+07	3.10E+07	3.10E+07	1.7	2.6	1.0
RPS3	16	18	13	17	11	16	3.90E+07	3.40E+07	2.30E+07	2.30E+07	1.7	1.5	1.0
TIMM44	13	14	11	12	13	15	1.90E+07	1.70E+07	1.10E+07	1.10E+07	1.7	1.5	1.0
EEF2	9	9	13	16	10	10	8.80E+06	1.40E+07	5.50E+06	5.50E+06	1.6	2.5	1.0
LMO7	12	12	11	13	7	9	8.30E+06	8.30E+06	2.50E+06	2.50E+06	3.3	3.3	1.0
NSF	12	12	8	8	11	12	9.90E+06	5.10E+06	7.30E+06	5.10E+06	1.9	1.0	1.4
GOT2	13	20	13	19	11	19	1.60E+08	9.10E+07	9.00E+07	9.00E+07	1.8	1.0	1.0
SHMT2	15	21	12	18	11	17	9.30E+07	6.20E+07	5.30E+07	5.30E+07	1.8	1.2	1.0
RAB7A	11	15	13	16	15	21	4.60E+07	3.30E+07	4.00E+07	3.30E+07	1.4	1.0	1.2
G3BP1	15	18	11	15	9	16	1.90E+07	1.10E+07	8.60E+06	8.60E+06	2.2	1.3	1.0
ITGB1	14	20	11	14	11	12	6.80E+07	5.50E+07	2.90E+07	2.90E+07	2.3	1.9	1.0
GFM1	11	13	13	15	13	15	2.40E+07	2.10E+07	1.90E+07	1.90E+07	1.3	1.1	1.0
DBN1	12	14	12	14	10	13	2.70E+07	1.60E+07	1.10E+07	1.10E+07	2.5	1.5	1.0
PPIB	12	17	8	11	10	12	5.60E+07	4.00E+07	3.60E+07	3.60E+07	1.6	1.1	1.0
ACAT1	12	15	10	13	10	11	2.80E+07	1.30E+07	8.70E+06	8.70E+06	3.2	1.5	1.0
NOMO1	13	14	11	12	11	12	9.80E+06	8.20E+06	5.90E+06	5.90E+06	1.7	1.4	1.0

ALDH2	11	13	12	13	11	12	7.70E+06	6.30E+06	1.20E+07	6.30E+06	1.2	1.0	1.9
PARP1	12	13	11	14	10	10	9.30E+06	1.00E+07	5.70E+06	5.70E+06	1.6	1.8	1.0
ALDH7A1	11	13	10	11	10	13	1.80E+07	1.50E+07	1.10E+07	1.10E+07	1.6	1.4	1.0
CTNND1	13	15	9	10	10	11	1.00E+07	8.10E+06	4.90E+06	4.90E+06	2.0	1.7	1.0
TPP2	11	12	13	13	10	10	1.20E+07	9.70E+06	4.30E+06	4.30E+06	2.8	2.3	1.0
TOP2A	10	12	11	14	7	9	8.00E+06	8.30E+06	4.40E+06	4.40E+06	1.8	1.9	1.0
IARS1	11	12	10	11	11	11	6.20E+06	5.40E+06	4.20E+06	4.20E+06	1.5	1.3	1.0
PKM	8	8	13	15	8	9	9.80E+06	1.60E+07	3.70E+06	3.70E+06	2.6	4.3	1.0
RARS1	12	13	10	10	9	9	5.10E+06	7.00E+06	4.40E+06	4.40E+06	1.2	1.6	1.0
MISP	11	12	9	9	11	11	2.40E+07	9.40E+06	6.80E+06	6.80E+06	3.5	1.4	1.0
IGF2R	11	11	11	11	10	10	7.10E+06	6.00E+06	4.00E+06	4.00E+06	1.8	1.5	1.0
UGGT1	11	12	7	7	6	6	5.20E+06	2.20E+06	1.70E+06	1.70E+06	3.1	1.3	1.0
BASP1	9	15	13	24	12	25	7.10E+07	1.50E+08	8.70E+07	7.10E+07	1.0	2.1	1.2
PABPC1	14	16	12	14	11	14	3.30E+07	2.20E+07	1.50E+07	1.50E+07	2.2	1.5	1.0
AP2A1	12	14	12	16	11	14	1.30E+07	8.30E+06	7.10E+06	7.10E+06	1.8	1.2	1.0
ACAA2	12	15	10	13	13	15	1.70E+07	1.40E+07	1.20E+07	1.20E+07	1.4	1.2	1.0
HSP90AB1	13	15	12	15	9	11	4.50E+07	6.30E+07	4.00E+07	4.00E+07	1.1	1.6	1.0
DDX3X	13	14	11	14	9	12	2.20E+07	2.10E+07	1.10E+07	1.10E+07	2.0	1.9	1.0
FASTKD2	13	13	10	12	10	14	2.00E+07	8.20E+06	1.00E+07	8.20E+06	2.4	1.0	1.2
SLC25A24	11	13	10	11	11	12	1.80E+07	9.60E+06	8.30E+06	8.30E+06	2.2	1.2	1.0
ITGAV	9	11	12	14	8	10	5.30E+06	7.70E+06	2.50E+06	2.50E+06	2.1	3.1	1.0
SFXN1	11	12	8	8	12	15	2.40E+07	1.60E+07	2.10E+07	1.60E+07	1.5	1.0	1.3
CSE1L	8	9	12	17	7	8	6.60E+07	7.70E+07	2.00E+06	2.00E+06	33.0	38.5	1.0
HNRNPM	10	10	13	15	7	9	1.10E+07	1.60E+07	8.40E+06	8.40E+06	1.3	1.9	1.0
ALPL	13	17	8	9	8	8	2.50E+07	8.10E+06	4.60E+06	4.60E+06	5.4	1.8	1.0
PLOD1	9	10	9	11	9	11	7.20E+06	1.00E+07	4.90E+06	4.90E+06	1.5	2.0	1.0
LARS2	12	12	10	10	9	9	1.60E+07	6.60E+06	4.80E+06	4.80E+06	3.3	1.4	1.0
CAVIN1	12	12	11	11	7	7	9.60E+06	7.30E+06	3.80E+06	3.80E+06	2.5	1.9	1.0
PXDN	14	14	9	9	5	7	9.40E+06	4.50E+06	1.60E+06	1.60E+06	5.9	2.8	1.0
CLPB	12	12	7	8	8	10	1.50E+07	8.70E+06	1.20E+07	8.70E+06	1.7	1.0	1.4
VWA8	7	7	10	11	10	11	2.90E+06	2.30E+06	2.10E+06	2.10E+06	1.4	1.1	1.0
SUPT16H	9	10	8	11	7	7	7.50E+06	8.90E+06	3.60E+06	3.60E+06	2.1	2.5	1.0
PLCB4	4	6	12	13	9	9	3.50E+06	5.80E+06	3.50E+06	3.50E+06	1.0	1.7	1.0
MRPS22	11	11	7	8	7	7	7.80E+06	6.20E+06	1.20E+07	6.20E+06	1.3	1.0	1.9
CDC42BPB	3	3	12	12	10	10	1.20E+06	5.80E+06	3.40E+06	1.20E+06	1.0	4.8	2.8
PHB1	11	20	13	22	12	24	1.70E+08	1.20E+08	1.40E+08	1.20E+08	1.4	1.0	1.2
DLD	13	18	11	19	10	18	3.20E+07	2.70E+07	2.30E+07	2.30E+07	1.4	1.2	1.0
RPS4X	10	14	9	14	12	17	1.90E+07	1.80E+07	1.20E+07	1.20E+07	1.6	1.5	1.0
ACAD9	11	12	12	15	11	15	1.80E+07	1.60E+07	1.40E+07	1.40E+07	1.3	1.1	1.0
ACSL4	10	12	9	10	11	13	1.90E+07	1.30E+07	1.40E+07	1.30E+07	1.5	1.0	1.1
DDOST	13	15	9	9	8	9	2.10E+07	1.30E+07	1.00E+07	1.00E+07	2.1	1.3	1.0
AARS2	7	7	11	14	11	12	3.00E+06	6.10E+06	4.10E+06	3.00E+06	1.0	2.0	1.4
P4HB	10	13	8	9	11	11	1.60E+07	7.20E+06	6.00E+06	6.00E+06	2.7	1.2	1.0
YME1L1	9	9	11	12	8	9	9.30E+06	1.00E+07	6.90E+06	6.90E+06	1.3	1.4	1.0
NUP210	11	12	9	9	8	8	1.10E+07	3.40E+06	4.00E+06	3.40E+06	3.2	1.0	1.2
DAP3	12	12	8	8	7	9	1.30E+07	7.20E+06	7.10E+06	7.10E+06	1.8	1.0	1.0
EZR	9	10	9	11	6	8	1.50E+07	1.80E+07	8.80E+06	8.80E+06	1.7	2.0	1.0
LAMC1	9	9	9	9	8	9	6.70E+06	5.80E+06	3.30E+06	3.30E+06	2.0	1.8	1.0
CPT2	10	11	7	7	9	9	7.40E+06	2.40E+06	5.10E+06	2.40E+06	3.1	1.0	2.1
EGFR	11	11	9	9	6	6	1.10E+07	1.40E+07	6.70E+06	6.70E+06	1.6	2.1	1.0
ENO1	7	7	11	12	5	5	5.80E+06	1.10E+07	4.60E+06	4.60E+06	1.3	2.4	1.0

HSD17B10	12	27	11	25	12	30	6.90E+07	3.40E+07	3.00E+07	3.00E+07	2.3	1.1	1.0
EEF1A1	8	16	12	34	10	25	6.20E+07	7.80E+07	3.90E+07	3.90E+07	1.6	2.0	1.0
TOMM40	12	23	10	14	10	15	4.30E+07	3.70E+07	2.10E+07	2.10E+07	2.0	1.8	1.0
ATAD3A	11	16	10	18	10	18	3.90E+07	2.70E+07	2.50E+07	2.50E+07	1.6	1.1	1.0
DLAT	8	10	10	18	8	15	6.40E+06	8.60E+06	6.60E+06	6.40E+06	1.0	1.3	1.0
UQCRC2	11	15	10	12	11	13	3.80E+07	2.40E+07	1.90E+07	1.90E+07	2.0	1.3	1.0
SYNCRIP	10	13	9	14	9	12	9.50E+06	6.00E+06	5.10E+06	5.10E+06	1.9	1.2	1.0
CLTCL1	11	15	6	10	9	13	1.50E+07	1.50E+07	8.20E+06	8.20E+06	1.8	1.8	1.0
AP2M1	11	13	11	14	9	11	1.20E+07	9.20E+06	8.30E+06	8.30E+06	1.4	1.1	1.0
SLC25A11	11	15	10	10	9	12	1.60E+07	9.20E+06	9.50E+06	9.20E+06	1.7	1.0	1.0
PTGES2	10	14	6	9	9	12	6.20E+06	4.00E+06	3.10E+06	3.10E+06	2.0	1.3	1.0
GNS	8	10	9	10	10	13	1.90E+07	1.20E+07	1.80E+07	1.20E+07	1.6	1.0	1.5
MATR3	9	11	9	12	6	9	5.50E+06	5.90E+06	2.30E+06	2.30E+06	2.4	2.6	1.0
OAT	11	12	10	12	8	8	1.40E+07	1.00E+07	5.50E+06	5.50E+06	2.5	1.8	1.0
KPNB1	10	11	9	10	9	11	6.70E+06	5.50E+06	3.80E+06	3.80E+06	1.8	1.4	1.0
PRKCSH	8	11	8	9	11	11	1.60E+07	1.40E+07	9.50E+06	9.50E+06	1.7	1.5	1.0
P4HA1	10	10	9	10	9	10	1.20E+07	1.10E+07	8.80E+06	8.80E+06	1.4	1.3	1.0
DDX5	9	11	9	10	8	9	1.60E+07	2.40E+07	9.10E+06	9.10E+06	1.8	2.6	1.0
ATP2A1	8	12	8	9	8	9	1.40E+07	1.70E+07	7.30E+06	7.30E+06	1.9	2.3	1.0
PMPCA	10	11	9	9	9	10	9.40E+06	4.90E+06	3.90E+06	3.90E+06	2.4	1.3	1.0
MARS1	10	11	9	9	9	10	4.00E+06	3.20E+06	2.30E+06	2.30E+06	1.7	1.4	1.0
STOML2	10	12	8	10	7	7	8.90E+06	4.80E+06	3.60E+06	3.60E+06	2.5	1.3	1.0
USO1	10	10	9	10	7	8	6.70E+06	7.70E+06	2.40E+06	2.40E+06	2.8	3.2	1.0
ECH1	10	12	6	7	7	8	3.10E+07	1.50E+07	1.20E+07	1.20E+07	2.6	1.3	1.0
EIF4G1	9	11	8	8	8	8	8.30E+06	7.70E+06	4.50E+06	4.50E+06	1.8	1.7	1.0
EIF3B	10	10	10	10	7	7	7.90E+06	7.10E+06	3.60E+06	3.60E+06	2.2	2.0	1.0
RPS9	11	13	7	7	6	6	2.80E+07	1.60E+07	1.20E+07	1.20E+07	2.3	1.3	1.0
MSN	8	8	7	7	5	7	3.00E+06	3.10E+06	1.10E+06	1.10E+06	2.7	2.8	1.0
NUP205	7	7	9	9	5	5	1.10E+06	3.40E+06	5.00E+05	5.00E+05	2.2	6.8	1.0
MRPL15	11	11	5	5	5	5	1.10E+07	3.40E+06	1.90E+06	1.90E+06	5.8	1.8	1.0
QARS1	7	7	8	8	5	5	2.90E+06	3.30E+06	1.20E+06	1.20E+06	2.4	2.8	1.0
ALDH1B1	10	15	10	14	9	10	1.60E+07	7.10E+06	6.40E+06	6.40E+06	2.5	1.1	1.0
IDH3B	10	12	7	13	7	14	8.90E+06	3.70E+06	1.60E+06	1.60E+06	5.6	2.3	1.0
NONO	8	13	9	14	6	11	1.20E+07	6.70E+06	3.70E+06	3.70E+06	3.2	1.8	1.0
NDUFA9	10	12	9	12	10	13	1.40E+07	9.20E+06	8.00E+06	8.00E+06	1.8	1.2	1.0
RPLP0	10	14	10	12	8	11	1.90E+07	1.60E+07	8.10E+06	8.10E+06	2.3	2.0	1.0
UQCRC1	11	15	8	10	7	11	2.50E+07	1.70E+07	1.40E+07	1.40E+07	1.8	1.2	1.0
TUBB4A	9	13	8	13	6	9	2.00E+07	1.20E+07	6.00E+06	6.00E+06	3.3	2.0	1.0
AGPS	9	17	7	9	7	9	9.80E+06	3.80E+06	4.10E+06	3.80E+06	2.6	1.0	1.1
ABCB6	9	10	10	11	10	13	5.70E+06	3.70E+06	3.00E+06	3.00E+06	1.9	1.2	1.0
SAMM50	8	11	9	12	9	11	1.70E+07	1.20E+07	9.80E+06	9.80E+06	1.7	1.2	1.0
HNRNPU	8	9	8	12	10	12	2.50E+07	1.80E+07	1.40E+07	1.40E+07	1.8	1.3	1.0
RPL7	11	13	8	9	9	9	2.50E+07	2.60E+07	1.10E+07	1.10E+07	2.3	2.4	1.0
GLS	10	12	8	9	8	9	1.30E+07	8.50E+06	6.90E+06	6.90E+06	1.9	1.2	1.0
NDUFS2	10	13	5	9	7	8	1.10E+07	7.90E+06	6.80E+06	6.80E+06	1.6	1.2	1.0
RPL3	9	13	6	7	7	10	2.40E+07	1.30E+07	8.50E+06	8.50E+06	2.8	1.5	1.0
ASPH	9	10	10	11	8	8	1.70E+07	1.10E+07	3.10E+06	3.10E+06	5.5	3.5	1.0
MMUT	8	8	9	10	11	11	7.70E+06	6.60E+06	6.00E+06	6.00E+06	1.3	1.1	1.0
CCT2	8	9	10	11	7	8	5.30E+06	5.30E+06	1.40E+06	1.40E+06	3.8	3.8	1.0
ATP2B1	8	9	10	10	9	9	5.60E+06	5.70E+06	4.00E+06	4.00E+06	1.4	1.4	1.0
TBRG4	11	12	7	8	6	8	1.70E+07	8.80E+06	1.00E+07	8.80E+06	1.9	1.0	1.1

PMPCB	8	9	7	8	9	11	1.70E+07	1.20E+07	1.40E+07	1.20E+07	1.4	1.0	1.2
HADH	10	15	6	7	6	6	2.80E+07	2.40E+07	1.90E+07	1.90E+07	1.5	1.3	1.0
PTPRF	9	10	8	10	6	7	3.10E+06	1.80E+06	9.20E+05	9.20E+05	3.4	2.0	1.0
ELAC2	8	9	6	6	11	12	3.00E+06	2.70E+06	2.60E+06	2.60E+06	1.2	1.0	1.0
COLGALT1	8	8	10	11	8	8	1.20E+07	1.10E+07	6.50E+06	6.50E+06	1.8	1.7	1.0
PDIA6	8	8	8	10	7	8	9.80E+06	1.90E+07	7.70E+06	7.70E+06	1.3	2.5	1.0
NCL	8	8	10	10	8	8	6.10E+06	8.50E+06	3.30E+06	3.30E+06	1.8	2.6	1.0
EIF4A1	8	8	10	10	7	8	1.00E+07	1.00E+07	4.60E+06	4.60E+06	2.2	2.2	1.0
CYB5R3	9	9	7	7	9	9	1.50E+07	1.00E+07	8.50E+06	8.50E+06	1.8	1.2	1.0
AP2A2	8	9	6	8	6	8	4.30E+06	2.20E+06	2.00E+06	2.00E+06	2.2	1.1	1.0
TMPO	6	9	8	9	7	7	4.80E+06	3.40E+06	1.90E+06	1.90E+06	2.5	1.8	1.0
ETFB	10	10	6	6	9	9	1.60E+07	6.60E+06	9.20E+06	6.60E+06	2.4	1.0	1.4
SARS2	9	9	6	6	7	8	5.40E+06	3.60E+06	3.80E+06	3.60E+06	1.5	1.0	1.1
ACSL3	8	11	4	5	7	7	1.10E+07	9.70E+06	5.80E+06	5.80E+06	1.9	1.7	1.0
PRORP	6	6	8	8	9	9	3.90E+06	2.20E+06	2.20E+06	2.20E+06	1.8	1.0	1.0
ABCB7	7	10	4	5	6	6	4.40E+06	3.30E+06	3.10E+06	3.10E+06	1.4	1.1	1.0
ESYT2	4	4	7	7	8	9	1.90E+06	5.50E+06	3.80E+06	1.90E+06	1.0	2.9	2.0
GOLGA2	9	9	4	6	5	5	5.40E+06	3.90E+06	2.80E+06	2.80E+06	1.9	1.4	1.0
H4C16	10	64	9	64	7	56	9.90E+08	7.70E+08	6.00E+08	6.00E+08	1.7	1.3	1.0
HSPE1	9	18	7	13	10	16	1.80E+08	1.10E+08	9.80E+07	9.80E+07	1.8	1.1	1.0
PDHA1	9	17	7	10	8	12	5.80E+07	4.00E+07	3.60E+07	3.60E+07	1.6	1.1	1.0
LRRC59	9	12	8	10	9	12	4.40E+07	2.60E+07	2.60E+07	2.60E+07	1.7	1.0	1.0
PTBP1	7	11	7	12	7	10	9.30E+06	7.20E+06	3.60E+06	3.60E+06	2.6	2.0	1.0
VDAC3	9	12	8	11	7	10	5.20E+07	5.20E+07	3.60E+07	3.60E+07	1.4	1.4	1.0
HNRNPK	9	12	8	11	7	8	1.40E+07	1.00E+07	4.20E+06	4.20E+06	3.3	2.4	1.0
STOM	7	8	9	12	9	11	1.20E+07	2.10E+07	1.50E+07	1.20E+07	1.0	1.8	1.3
PLOD3	7	8	8	10	8	12	1.20E+07	9.90E+06	8.20E+06	8.20E+06	1.5	1.2	1.0
MCAM	8	9	8	10	8	10	1.20E+07	7.90E+06	7.50E+06	7.50E+06	1.6	1.1	1.0
SLC2A1	10	13	6	9	5	7	3.80E+07	3.00E+07	1.60E+07	1.60E+07	2.4	1.9	1.0
DLST	9	10	7	8	9	9	1.30E+07	8.20E+06	8.40E+06	8.20E+06	1.6	1.0	1.0
LAP3	9	9	7	8	8	10	6.80E+06	5.80E+06	5.10E+06	5.10E+06	1.3	1.1	1.0
MRPS35	8	9	7	8	6	8	4.00E+06	2.20E+06	2.30E+06	2.20E+06	1.8	1.0	1.0
SFPQ	10	11	7	8	5	6	2.70E+07	1.90E+07	9.70E+06	9.70E+06	2.8	2.0	1.0
PKN2	5	5	10	11	7	8	1.20E+06	2.60E+06	1.50E+06	1.20E+06	1.0	2.2	1.3
PITRM1	8	9	8	8	7	7	5.00E+06	3.30E+06	2.90E+06	2.90E+06	1.7	1.1	1.0
SUPV3L1	8	9	7	7	7	8	6.50E+06	4.50E+06	3.50E+06	3.50E+06	1.9	1.3	1.0
CYP51A1	6	6	8	11	6	6	4.00E+06	4.50E+06	2.50E+06	2.50E+06	1.6	1.8	1.0
FLOT2	7	7	7	7	9	9	6.70E+06	3.70E+06	4.60E+06	3.70E+06	1.8	1.0	1.2
NDUFS3	8	8	5	6	6	7	7.10E+06	2.80E+06	1.80E+06	1.80E+06	3.9	1.6	1.0
MRPL4	9	10	5	5	5	6	1.10E+07	4.20E+06	4.60E+06	4.20E+06	2.6	1.0	1.1
DARS1	7	7	5	6	7	7	4.80E+06	4.30E+06	3.20E+06	3.20E+06	1.5	1.3	1.0
P4HA2	8	8	5	5	6	6	6.40E+06	2.20E+06	2.30E+06	2.20E+06	2.9	1.0	1.0
EIF3C	5	5	8	9	5	5	2.50E+06	6.00E+06	3.00E+06	2.50E+06	1.0	2.4	1.2
ROBO1	7	8	5	6	4	4	3.30E+06	1.70E+06	9.60E+05	9.60E+05	3.4	1.8	1.0
DDX28	5	6	7	7	5	5	2.40E+06	1.90E+06	9.70E+05	9.70E+05	2.5	2.0	1.0
LAMB1	6	6	6	6	5	5	2.80E+06	4.70E+06	7.60E+06	2.80E+06	1.0	1.7	2.7
FERMT2	9	9	5	5	3	3	2.30E+06	9.50E+05	2.60E+05	2.60E+05	8.8	3.7	1.0
ECHS1	7	7	5	5	5	5	8.10E+06	6.20E+06	3.00E+06	3.00E+06	2.7	2.1	1.0
PIEZO1	5	5	5	6	3	4	9.70E+05	1.20E+06	6.10E+05	6.10E+05	1.6	2.0	1.0
H2BC12	9	54	8	29	9	25	1.60E+08	3.90E+07	1.30E+08	3.90E+07	4.1	1.0	3.3
SSBP1	8	16	7	14	6	16	2.50E+07	2.00E+07	1.10E+07	1.10E+07	2.3	1.8	1.0

TUBB	9	12	8	17	7	16	2.00E+07	2.10E+07	9.40E+06	9.40E+06	2.1	2.2	1.0
TOMM22	9	15	8	16	8	11	1.00E+07	1.00E+07	5.30E+06	5.30E+06	1.9	1.9	1.0
ATP5F1C	8	14	6	12	7	15	4.60E+07	2.70E+07	2.60E+07	2.60E+07	1.8	1.0	1.0
ETFA	8	12	8	13	7	14	2.10E+07	1.20E+07	9.90E+06	9.90E+06	2.1	1.2	1.0
RACK1	9	13	8	12	8	14	2.00E+07	1.80E+07	1.50E+07	1.50E+07	1.3	1.2	1.0
MRPS27	7	8	7	12	9	13	4.30E+06	5.70E+06	5.50E+06	4.30E+06	1.0	1.3	1.3
TSFM	8	11	4	9	7	11	8.50E+06	5.90E+06	6.00E+06	5.90E+06	1.4	1.0	1.0
SLC1A5	8	9	6	11	6	11	2.50E+07	2.90E+07	1.60E+07	1.60E+07	1.6	1.8	1.0
CDH2	8	9	8	13	5	8	5.20E+06	1.90E+07	1.80E+06	1.80E+06	2.9	10.6	1.0
PGAM5	6	8	9	10	9	12	1.50E+07	1.30E+07	1.00E+07	1.00E+07	1.5	1.3	1.0
HNRNPA2B1	6	6	7	12	7	10	1.40E+07	1.10E+07	9.40E+06	9.40E+06	1.5	1.2	1.0
MTHFD2	8	9	7	10	7	7	9.60E+06	9.10E+06	6.30E+06	6.30E+06	1.5	1.4	1.0
HPDL	9	11	7	7	7	8	7.20E+06	3.60E+06	2.30E+06	2.30E+06	3.1	1.6	1.0
SUCLG2	5	6	6	8	9	12	4.30E+06	1.70E+07	3.70E+06	3.70E+06	1.2	4.6	1.0
YARS2	9	11	6	9	5	5	9.10E+06	5.60E+06	2.10E+06	2.10E+06	4.3	2.7	1.0
HNRNPR	8	9	8	9	7	7	1.60E+07	1.30E+07	6.40E+06	6.40E+06	2.5	2.0	1.0
HK1	7	7	7	9	7	8	4.60E+06	4.60E+06	3.00E+06	3.00E+06	1.5	1.5	1.0
NPC1	8	10	5	7	6	7	1.80E+07	1.10E+07	8.30E+06	8.30E+06	2.2	1.3	1.0
NADK2	6	6	8	8	8	9	8.40E+06	8.10E+06	6.70E+06	6.70E+06	1.3	1.2	1.0
HNRNPA1	6	10	5	7	3	6	6.60E+06	3.00E+06	1.90E+06	1.90E+06	3.5	1.6	1.0
KIF23	7	7	8	9	4	6	3.30E+06	4.30E+06	2.30E+06	2.30E+06	1.4	1.9	1.0
SEC63	7	7	6	6	7	8	4.40E+06	2.80E+06	2.80E+06	2.80E+06	1.6	1.0	1.0
ACADM	8	9	5	5	7	7	1.40E+07	1.20E+07	8.00E+06	8.00E+06	1.8	1.5	1.0
CCT3	7	7	8	9	5	5	4.30E+06	6.10E+06	2.00E+06	2.00E+06	2.2	3.1	1.0
APOB	4	4	9	13	4	4	2.00E+06	1.80E+07	2.40E+06	2.00E+06	1.0	9.0	1.2
CNP	8	8	5	6	6	6	2.30E+07	3.30E+06	1.30E+07	3.30E+06	7.0	1.0	3.9
CORO1C	8	8	6	6	5	6	1.40E+07	7.70E+06	4.50E+06	4.50E+06	3.1	1.7	1.0
NES	7	7	9	10	3	3	2.60E+06	2.80E+06	4.80E+05	4.80E+05	5.4	5.8	1.0
NOA1	6	6	6	7	7	7	7.10E+06	7.60E+06	6.50E+06	6.50E+06	1.1	1.2	1.0
ATP6V0A1	7	7	5	5	8	8	3.10E+06	2.40E+06	1.90E+06	1.90E+06	1.6	1.3	1.0
KARS1	6	9	5	5	5	5	5.40E+06	2.10E+06	1.80E+06	1.80E+06	3.0	1.2	1.0
ILF3	6	6	6	6	6	7	3.80E+06	3.90E+06	3.70E+06	3.70E+06	1.0	1.1	1.0
COL5A1	8	8	4	4	4	6	5.20E+06	8.50E+05	1.10E+06	8.50E+05	6.1	1.0	1.3
GAPDH	7	7	6	8	3	3	1.60E+07	1.50E+07	8.00E+06	8.00E+06	2.0	1.9	1.0
TJP1	6	6	7	8	4	4	2.00E+06	2.70E+06	1.10E+06	1.10E+06	1.8	2.5	1.0
SCCPDH	6	6	4	4	7	8	1.10E+07	7.70E+06	1.20E+07	7.70E+06	1.4	1.0	1.6
HSPA4	6	6	7	8	3	3	2.30E+06	2.30E+06	6.50E+05	6.50E+05	3.5	3.5	1.0
RPL6	7	7	5	6	4	4	1.30E+07	9.60E+06	4.20E+06	4.20E+06	3.1	2.3	1.0
PDCD6IP	6	6	6	6	4	4	2.40E+06	3.10E+06	1.10E+06	1.10E+06	2.2	2.8	1.0
ABCC4	5	5	7	7	4	4	1.80E+06	2.00E+06	5.80E+05	5.80E+05	3.1	3.4	1.0
PCYOX1	8	8	3	3	5	5	5.00E+06	1.00E+06	1.60E+06	1.00E+06	5.0	1.0	1.6
CYCS	8	16	5	10	5	13	4.10E+07	2.20E+07	2.50E+07	2.20E+07	1.9	1.0	1.1
BSG	8	11	8	11	8	13	4.20E+07	3.80E+07	3.10E+07	3.10E+07	1.4	1.2	1.0
RPL4	6	9	7	13	6	12	1.50E+07	2.60E+07	1.50E+07	1.50E+07	1.0	1.7	1.0
COX4I1	8	11	6	13	5	10	2.60E+07	2.40E+07	1.90E+07	1.90E+07	1.4	1.3	1.0
TIMM50	8	12	6	10	5	10	1.50E+07	1.10E+07	6.90E+06	6.90E+06	2.2	1.6	1.0
MIA3	4	5	5	16	7	9	1.00E+06	5.40E+06	2.60E+06	1.00E+06	1.0	5.4	2.6
PRDX5	8	8	8	10	8	11	9.70E+06	9.30E+06	7.30E+06	7.30E+06	1.3	1.3	1.0
RPS18	8	10	6	10	6	9	1.90E+07	1.60E+07	9.00E+06	9.00E+06	2.1	1.8	1.0
ATP5PO	7	9	7	9	7	8	3.80E+07	2.90E+07	2.40E+07	2.40E+07	1.6	1.2	1.0
TUBA3C	8	11	5	9	4	5	3.80E+07	3.50E+07	1.70E+07	1.70E+07	2.2	2.1	1.0

ATP5PB	5	7	8	10	6	8	2.10E+07	2.00E+07	1.20E+07	1.20E+07	1.8	1.7	1.0
ILF2	8	9	6	7	7	8	5.00E+06	2.80E+06	1.90E+06	1.90E+06	2.6	1.5	1.0
GLB1	7	9	7	7	7	8	7.80E+06	4.90E+06	4.40E+06	4.40E+06	1.8	1.1	1.0
MRPL37	6	8	7	8	7	8	1.00E+07	8.50E+06	6.30E+06	6.30E+06	1.6	1.3	1.0
RPL7A	8	8	6	7	7	8	6.70E+07	2.40E+07	1.90E+07	1.90E+07	3.5	1.3	1.0
IDH3A	7	7	5	7	6	9	1.00E+07	8.80E+06	5.40E+06	5.40E+06	1.9	1.6	1.0
MTDH	7	8	6	6	8	8	7.10E+06	3.40E+06	5.20E+06	3.40E+06	2.1	1.0	1.5
XPO1	6	6	8	9	4	6	1.90E+06	2.50E+06	6.90E+05	6.90E+05	2.8	3.6	1.0
VPS35	6	6	6	7	8	8	3.10E+06	3.10E+06	2.90E+06	2.90E+06	1.1	1.1	1.0
NDUFV1	6	6	6	7	7	8	6.00E+06	4.30E+06	3.70E+06	3.70E+06	1.6	1.2	1.0
KPNA2	6	7	7	8	5	5	3.00E+06	3.30E+06	1.00E+06	1.00E+06	3.0	3.3	1.0
RANGAP1	6	6	6	8	5	6	2.10E+06	3.00E+06	1.90E+06	1.90E+06	1.1	1.6	1.0
RHOT2	6	6	7	7	7	7	5.40E+06	3.90E+06	3.20E+06	3.20E+06	1.7	1.2	1.0
HDHD5	7	8	6	6	6	6	4.70E+06	2.80E+06	1.80E+06	1.80E+06	2.6	1.6	1.0
ERAL1	8	8	5	5	6	6	5.60E+06	2.50E+06	3.00E+06	2.50E+06	2.2	1.0	1.2
AK2	8	9	5	5	5	5	8.70E+06	2.10E+06	1.80E+06	1.80E+06	4.8	1.2	1.0
CD109	6	6	5	5	7	8	1.40E+06	1.40E+06	1.00E+06	1.00E+06	1.4	1.4	1.0
EIF4G2	3	3	8	8	7	8	1.10E+06	1.80E+06	1.40E+06	1.10E+06	1.0	1.6	1.3
PDPR	7	7	6	6	6	6	9.10E+06	8.10E+06	4.70E+06	4.70E+06	1.9	1.7	1.0
HSDL2	6	6	5	5	7	7	6.60E+06	1.30E+06	3.20E+06	1.30E+06	5.1	1.0	2.5
GTPBP10	6	6	4	5	6	7	2.10E+06	7.60E+05	1.90E+06	7.60E+05	2.8	1.0	2.5
MTPAP	6	7	4	5	6	6	3.80E+06	3.10E+06	7.90E+06	3.10E+06	1.2	1.0	2.5
MRPS9	7	8	4	4	6	6	7.30E+06	2.40E+06	2.90E+06	2.40E+06	3.0	1.0	1.2
SACM1L	7	7	6	6	5	5	4.30E+06	2.70E+06	1.90E+06	1.90E+06	2.3	1.4	1.0
PLOD2	6	6	6	8	4	4	3.40E+06	4.60E+06	1.60E+06	1.60E+06	2.1	2.9	1.0
PALS2	8	8	5	5	5	5	6.50E+06	3.50E+06	2.10E+06	2.10E+06	3.1	1.7	1.0
SLC12A2	5	5	7	7	6	6	4.30E+06	4.60E+06	3.20E+06	3.20E+06	1.3	1.4	1.0
HM13	6	7	5	6	5	5	7.00E+06	6.50E+06	3.50E+06	3.50E+06	2.0	1.9	1.0
GCN1	4	4	7	8	5	5	1.50E+06	1.60E+06	3.10E+05	3.10E+05	4.8	5.2	1.0
RPS3A	6	6	6	7	4	4	9.40E+06	9.90E+06	4.90E+06	4.90E+06	1.9	2.0	1.0
MOGS	7	7	5	5	5	5	2.90E+06	2.20E+06	1.60E+06	1.60E+06	1.8	1.4	1.0
ACOT2	4	7	3	3	5	6	1.10E+06	2.50E+06	2.20E+06	1.10E+06	1.0	2.3	2.0
MACROH2A	5	5	5	5	6	6	2.80E+06	3.60E+06	2.50E+06	2.50E+06	1.1	1.4	1.0
COPB2	5	5	4	4	6	6	8.40E+06	3.60E+06	2.40E+06	2.40E+06	3.5	1.5	1.0
MT-CO2	3	4	3	4	6	7	3.40E+06	3.10E+06	1.70E+06	1.70E+06	2.0	1.8	1.0
SLC12A7	3	3	5	6	6	6	9.80E+05	3.30E+06	2.00E+06	9.80E+05	1.0	3.4	2.0
PI4KA	7	7	4	4	4	4	4.80E+06	9.90E+05	6.40E+05	6.40E+05	7.5	1.5	1.0
MRPL38	4	4	5	5	5	6	2.00E+06	2.20E+06	2.50E+06	2.00E+06	1.0	1.1	1.3
MRPL1	6	6	3	3	5	5	5.10E+06	3.30E+06	3.30E+06	3.30E+06	1.5	1.0	1.0
HEATR1	4	4	5	5	4	5	5.60E+05	6.50E+05	4.90E+05	4.90E+05	1.1	1.3	1.0
SMARCA5	3	3	5	6	5	5	1.20E+06	2.40E+06	8.10E+05	8.10E+05	1.5	3.0	1.0
GRSF1	5	5	2	2	6	6	1.90E+06	1.00E+06	9.60E+05	9.60E+05	2.0	1.0	1.0
NCSTN	5	5	3	3	5	5	4.50E+06	2.30E+06	1.10E+07	2.30E+06	2.0	1.0	4.8
TFB1M	5	5	4	4	4	4	3.40E+06	1.70E+06	1.60E+06	1.60E+06	2.1	1.1	1.0
L2HGDH	4	4	4	4	4	4	1.90E+06	8.80E+05	1.60E+06	8.80E+05	2.2	1.0	1.8
MRRF	7	7	2	2	3	3	3.90E+06	1.20E+06	9.70E+05	9.70E+05	4.0	1.2	1.0
EFTUD2	3	3	4	5	3	3	2.50E+05	7.60E+05	2.60E+05	2.50E+05	1.0	3.0	1.0
POLG	4	5	2	2	4	4	1.50E+06	4.30E+05	7.00E+05	4.30E+05	3.5	1.0	1.6
KIF5B	3	3	7	7	0	0	1.30E+06	1.90E+06	NF	1.30E+06	1.0	1.5	0.0
SRPRA	2	2	2	2	5	5	5.30E+05	5.80E+05	8.60E+05	5.30E+05	1.0	1.1	1.6
VDAC2	7	17	7	15	7	15	2.00E+08	1.30E+08	1.00E+08	1.00E+08	2.0	1.3	1.0

Ighg1	6	14	6	10	5	11	1.50E+08	1.20E+08	1.20E+08	1.20E+08	1.3	1.0	1.0
ACTA2	5	9	5	11	4	10	6.50E+07	7.00E+07	2.90E+07	2.90E+07	2.2	2.4	1.0
RPS6	6	9	5	8	5	10	1.40E+07	1.50E+07	1.50E+07	1.40E+07	1.0	1.1	1.1
RPSA	6	8	6	8	7	10	3.30E+07	2.60E+07	2.10E+07	2.10E+07	1.6	1.2	1.0
MTCH2	7	9	6	8	6	8	2.40E+07	1.60E+07	1.30E+07	1.30E+07	1.8	1.2	1.0
SCAMP3	6	8	7	9	6	7	8.60E+06	5.90E+06	4.20E+06	4.20E+06	2.0	1.4	1.0
RPS19	6	7	7	10	6	7	1.20E+07	1.30E+07	6.40E+06	6.40E+06	1.9	2.0	1.0
RAB5C	7	8	5	7	4	8	7.00E+06	5.50E+06	5.60E+06	5.50E+06	1.3	1.0	1.0
RPS7	5	7	5	8	6	7	1.30E+07	1.60E+06	2.10E+06	1.60E+06	8.1	1.0	1.3
PCK2	6	7	6	7	5	8	6.30E+06	4.10E+06	3.10E+06	3.10E+06	2.0	1.3	1.0
SFXN3	6	7	6	8	6	7	1.00E+07	8.00E+06	5.30E+06	5.30E+06	1.9	1.5	1.0
RPS16	7	8	4	6	6	8	1.80E+07	2.10E+07	1.30E+07	1.30E+07	1.4	1.6	1.0
CYC1	3	7	5	6	5	8	2.10E+07	6.40E+06	6.30E+06	6.30E+06	3.3	1.0	1.0
RPS2	6	8	5	7	6	6	2.00E+07	1.30E+07	9.20E+06	9.20E+06	2.2	1.4	1.0
DECR1	5	6	5	5	6	10	4.80E+06	3.30E+06	5.70E+06	3.30E+06	1.5	1.0	1.7
MARCKS	4	6	4	5	6	9	7.80E+06	8.60E+06	1.30E+07	7.80E+06	1.0	1.1	1.7
DDX21	5	5	7	9	6	6	2.00E+06	4.90E+06	2.20E+06	2.00E+06	1.0	2.5	1.1
ERP29	7	7	6	6	7	7	5.60E+06	4.00E+06	3.00E+06	3.00E+06	1.9	1.3	1.0
RPL13A	6	7	4	6	5	7	1.20E+07	1.20E+07	9.40E+06	9.40E+06	1.3	1.3	1.0
RAB14	6	6	6	7	5	6	7.60E+06	4.50E+06	3.10E+06	3.10E+06	2.5	1.5	1.0
PTCD3	6	7	6	6	6	6	4.40E+06	3.50E+06	2.20E+06	2.20E+06	2.0	1.6	1.0
GADD45GIP1	3	3	6	8	6	8	2.10E+06	5.90E+06	1.20E+07	2.10E+06	1.0	2.8	5.7
LDHA	6	7	7	7	5	5	7.40E+06	7.70E+06	3.10E+06	3.10E+06	2.4	2.5	1.0
RPL13	3	4	7	7	7	8	8.60E+06	1.30E+07	9.30E+06	8.60E+06	1.0	1.5	1.1
SUCLA2	6	9	3	3	6	7	9.00E+06	2.20E+06	2.60E+06	2.20E+06	4.1	1.0	1.2
SEC22B	6	6	4	6	6	6	3.40E+06	2.40E+06	1.70E+06	1.70E+06	2.0	1.4	1.0
ZC3HAV1	5	6	5	5	7	7	2.50E+06	1.30E+07	1.20E+07	2.50E+06	1.0	5.2	4.8
ECSIT	5	6	5	5	7	7	1.00E+06	6.60E+05	7.90E+05	6.60E+05	1.5	1.0	1.2
RUVBL1	5	6	7	7	5	5	2.40E+06	2.00E+06	7.60E+05	7.60E+05	3.2	2.6	1.0
MRPL39	7	8	4	4	6	6	5.10E+06	1.90E+06	2.90E+06	1.90E+06	2.7	1.0	1.5
SF3B3	6	7	3	5	4	5	2.00E+06	8.70E+05	6.00E+05	6.00E+05	3.3	1.5	1.0
SCFD1	5	5	4	4	6	8	1.50E+06	1.50E+06	1.10E+06	1.10E+06	1.4	1.4	1.0
CAT	5	5	3	4	6	8	2.00E+06	3.20E+06	4.30E+06	2.00E+06	1.0	1.6	2.2
DNAJC10	6	7	4	4	6	6	2.80E+06	7.20E+05	1.10E+06	7.20E+05	3.9	1.0	1.5
PCCA	6	8	0	0	7	9	3.00E+06	NF	2.60E+06	2.60E+06	1.2	0.0	1.0
GNAI2	6	6	5	6	4	4	3.40E+06	3.00E+06	2.40E+06	2.40E+06	1.4	1.3	1.0
IPO7	3	3	6	7	6	6	1.40E+06	2.70E+06	1.00E+06	1.00E+06	1.4	2.7	1.0
UBA1	3	3	6	7	6	6	4.20E+05	1.10E+06	4.90E+05	4.20E+05	1.0	2.6	1.2
KIF14	5	5	6	6	4	5	8.90E+05	2.00E+06	9.60E+05	8.90E+05	1.0	2.2	1.1
HNRNPL	6	10	4	4	2	2	3.10E+06	1.70E+06	6.80E+05	6.80E+05	4.6	2.5	1.0
UFL1	5	5	5	6	5	5	2.50E+06	2.10E+06	1.60E+06	1.60E+06	1.6	1.3	1.0
PI4K2A	5	5	5	7	4	4	2.30E+06	2.20E+06	1.20E+06	1.20E+06	1.9	1.8	1.0
ERBIN	5	5	5	6	5	5	1.20E+06	1.30E+06	1.00E+06	1.00E+06	1.2	1.3	1.0
RPS15A	7	8	4	4	4	4	1.20E+07	8.50E+06	5.10E+06	5.10E+06	2.4	1.7	1.0
UQCRFS1	6	8	4	4	3	3	5.20E+06	4.00E+06	2.60E+06	2.60E+06	2.0	1.5	1.0
CLU	6	6	4	4	5	5	1.20E+07	4.40E+06	3.50E+06	3.50E+06	3.4	1.3	1.0
STT3B	7	7	4	4	4	4	4.90E+06	3.30E+06	1.90E+06	1.90E+06	2.6	1.7	1.0
CAPZA1	4	4	6	6	4	4	2.10E+06	2.30E+06	1.20E+06	1.20E+06	1.8	1.9	1.0
NCLN	7	7	3	3	4	4	4.10E+06	2.10E+06	1.80E+06	1.80E+06	2.3	1.2	1.0
GBA1	7	8	4	4	2	2	2.20E+06	6.30E+05	6.40E+05	6.30E+05	3.5	1.0	1.0
FXR1	4	4	5	5	4	5	5.10E+06	3.60E+06	2.00E+06	2.00E+06	2.6	1.8	1.0

MTIF2	3	3	4	4	6	7	1.50E+06	1.30E+06	5.80E+06	1.30E+06	1.2	1.0	4.5
RHOT1	5	5	4	4	5	5	1.30E+06	9.60E+05	9.80E+05	9.60E+05	1.4	1.0	1.0
MTCH1	4	5	6	6	3	3	3.90E+06	4.50E+06	2.80E+06	2.80E+06	1.4	1.6	1.0
CPD	3	4	2	2	7	8	8.40E+05	5.00E+05	1.20E+06	5.00E+05	1.7	1.0	2.4
MIPEP	5	5	3	3	6	6	2.20E+06	1.40E+06	1.90E+06	1.40E+06	1.6	1.0	1.4
PCCB	4	5	3	3	5	5	2.10E+06	1.20E+06	1.30E+06	1.20E+06	1.8	1.0	1.1
HIP1	2	2	5	5	6	6	9.80E+05	1.70E+06	1.80E+06	9.80E+05	1.0	1.7	1.8
ME2	6	6	4	4	3	3	2.90E+06	8.40E+05	6.60E+05	6.60E+05	4.4	1.3	1.0
SCARB2	4	5	4	4	4	4	4.30E+06	2.50E+06	3.90E+06	2.50E+06	1.7	1.0	1.6
CCT6A	3	3	6	7	3	3	9.40E+05	5.10E+06	9.80E+05	9.40E+05	1.0	5.4	1.0
RAP1B	7	7	3	3	3	3	7.80E+06	5.60E+06	3.80E+06	3.80E+06	2.1	1.5	1.0
LMNB2	0	0	5	7	6	6	NF	4.60E+06	2.90E+06	2.90E+06	0.0	1.6	1.0
NLN	6	6	5	5	2	2	4.20E+06	2.80E+06	1.50E+06	1.50E+06	2.8	1.9	1.0
PABPC4	5	5	4	4	4	4	8.20E+06	2.90E+06	3.70E+06	2.90E+06	2.8	1.0	1.3
DNAJC11	5	5	5	5	2	2	3.90E+06	2.20E+06	4.50E+05	4.50E+05	8.7	4.9	1.0
LNPEP	4	4	5	5	3	3	1.40E+06	2.50E+06	1.00E+06	1.00E+06	1.4	2.5	1.0
CARS2	3	3	3	3	6	6	5.50E+06	4.10E+06	5.70E+06	4.10E+06	1.3	1.0	1.4
LMAN2	6	6	2	2	3	3	3.10E+06	1.00E+06	1.10E+06	1.00E+06	3.1	1.0	1.1
SMC2	0	0	6	7	4	4	NF	2.80E+06	9.10E+05	9.10E+05	0.0	3.1	1.0
TRIM21	3	3	5	5	3	3	1.30E+06	2.50E+06	1.60E+06	1.30E+06	1.0	1.9	1.2
VPS13C	5	5	1	1	3	3	6.60E+05	7.70E+04	1.80E+05	7.70E+04	8.6	1.0	2.3
H2AC8	5	10	4	14	5	14	2.20E+08	2.00E+08	1.40E+08	1.40E+08	1.6	1.4	1.0
	5	10	4	14	4	11	2.00E+07	5.10E+07	3.20E+07	2.00E+07	1.0	2.6	1.6
FKBP8	6	7	6	8	6	10	7.00E+06	5.40E+06	5.30E+06	5.30E+06	1.3	1.0	1.0
CD44	4	7	5	8	6	9	2.70E+07	1.60E+07	9.30E+06	9.30E+06	2.9	1.7	1.0
RPS17	6	8	4	6	5	9	3.20E+06	1.40E+06	1.20E+06	1.20E+06	2.7	1.2	1.0
CD63	5	7	4	6	6	10	2.80E+07	2.80E+07	2.70E+07	2.70E+07	1.0	1.0	1.0
RAB1A	4	6	4	7	5	8	7.80E+06	8.00E+06	4.30E+06	4.30E+06	1.8	1.9	1.0
GARS1	5	6	6	6	5	9	5.70E+06	1.80E+06	2.60E+06	1.80E+06	3.2	1.0	1.4
RPS14	6	8	4	7	3	6	1.50E+07	1.50E+07	1.20E+07	1.20E+07	1.3	1.3	1.0
ANXA2	5	6	6	8	4	5	2.20E+07	1.90E+07	1.10E+07	1.10E+07	2.0	1.7	1.0
CCDC47	5	5	5	8	4	6	3.20E+06	3.90E+06	1.70E+06	1.70E+06	1.9	2.3	1.0
LAMP1	5	7	5	7	5	5	3.60E+07	2.40E+07	1.90E+07	1.90E+07	1.9	1.3	1.0
SDHB	6	6	6	7	5	5	8.80E+06	7.70E+06	4.90E+06	4.90E+06	1.8	1.6	1.0
RAB2A	6	6	6	6	5	5	9.30E+06	8.20E+06	5.80E+06	5.80E+06	1.6	1.4	1.0
NPM1	5	6	5	6	5	5	2.70E+06	1.80E+06	6.80E+05	6.80E+05	4.0	2.6	1.0
MRPL11	6	7	5	5	5	5	6.70E+06	5.90E+06	3.30E+06	3.30E+06	2.0	1.8	1.0
PTBP3	4	5	6	6	4	6	4.90E+06	6.90E+06	4.30E+06	4.30E+06	1.1	1.6	1.0
NFS1	4	5	5	6	5	5	3.20E+06	2.30E+06	1.70E+06	1.70E+06	1.9	1.4	1.0
TOR1AIP1	5	5	4	6	4	5	1.50E+06	9.70E+05	5.90E+05	5.90E+05	2.5	1.6	1.0
EIF3L	6	6	5	5	4	4	3.00E+06	2.30E+06	1.30E+06	1.30E+06	2.3	1.8	1.0
CDK5RAP3	5	6	3	3	6	6	2.10E+06	6.50E+05	1.00E+06	6.50E+05	3.2	1.0	1.5
TMEM165	3	3	5	6	4	6	1.30E+06	3.20E+06	1.80E+06	1.30E+06	1.0	2.5	1.4
DDRGK1	4	4	4	4	6	7	5.00E+06	2.10E+06	3.70E+06	2.10E+06	2.4	1.0	1.8
RACGAP1	4	4	5	7	4	4	2.40E+06	3.00E+06	1.30E+06	1.30E+06	1.8	2.3	1.0
DHODH	4	5	5	5	5	5	2.70E+06	2.60E+06	2.10E+06	2.10E+06	1.3	1.2	1.0
EEF1G	5	6	5	5	4	4	9.60E+06	6.90E+06	3.00E+06	3.00E+06	3.2	2.3	1.0
OSBPL8	5	5	4	5	4	5	1.30E+06	1.70E+06	4.30E+06	1.30E+06	1.0	1.3	3.3
HEXA	5	5	5	5	5	5	2.80E+06	2.50E+06	2.00E+06	2.00E+06	1.4	1.3	1.0
MRPL9	5	6	5	5	4	4	1.30E+07	2.90E+06	1.90E+06	1.90E+06	6.8	1.5	1.0
PDE12	4	5	4	5	5	5	4.80E+06	3.70E+06	2.70E+06	2.70E+06	1.8	1.4	1.0

COL4A2	6	6	4	4	4	4	1.30E+07	6.20E+06	2.90E+06	2.90E+06	4.5	2.1	1.0
RARS2	4	5	3	3	5	6	3.10E+06	7.20E+05	1.50E+06	7.20E+05	4.3	1.0	2.1
RBM39	5	5	4	4	5	5	2.60E+06	9.00E+06	8.60E+06	2.60E+06	1.0	3.5	3.3
BAG6	3	4	4	4	6	6	6.90E+05	6.40E+05	2.00E+06	6.40E+05	1.1	1.0	3.1
GNAS	5	6	3	3	5	5	5.90E+06	5.70E+06	4.70E+06	4.70E+06	1.3	1.2	1.0
CCT4	3	4	5	5	5	5	2.00E+06	2.70E+06	1.20E+06	1.20E+06	1.7	2.3	1.0
APMAP	5	5	4	4	4	5	3.20E+06	4.10E+06	2.60E+06	2.60E+06	1.2	1.6	1.0
PA2G4	5	5	4	4	4	5	3.60E+06	2.30E+06	1.50E+06	1.50E+06	2.4	1.5	1.0
ATL3	5	5	4	4	4	4	1.80E+06	8.50E+05	5.80E+05	5.80E+05	3.1	1.5	1.0
LAMA5	2	2	5	6	5	5	5.50E+05	1.20E+06	8.70E+05	5.50E+05	1.0	2.2	1.6
ABCB10	6	7	3	3	3	3	1.80E+06	1.00E+06	8.50E+05	8.50E+05	2.1	1.2	1.0
CLPP	3	4	4	4	5	5	1.60E+06	1.50E+06	1.30E+06	1.30E+06	1.2	1.2	1.0
SRP72	4	4	3	3	6	6	2.40E+06	1.70E+06	1.70E+06	1.70E+06	1.4	1.0	1.0
ERLIN2	4	4	4	4	4	5	5.60E+06	2.70E+06	2.60E+06	2.60E+06	2.2	1.0	1.0
PPP2R1A	3	3	5	5	4	5	7.50E+05	3.50E+05	2.10E+05	2.10E+05	3.6	1.7	1.0
LSS	3	3	4	4	5	6	8.50E+05	1.70E+06	2.10E+06	8.50E+05	1.0	2.0	2.5
ACADSB	3	3	4	4	6	6	9.00E+05	1.20E+06	1.50E+06	9.00E+05	1.0	1.3	1.7
CLPTM1L	3	3	4	5	5	5	1.70E+06	1.40E+06	1.60E+06	1.40E+06	1.2	1.0	1.1
PTCD1	5	5	5	5	3	3	1.50E+06	1.20E+06	6.80E+05	6.80E+05	2.2	1.8	1.0
CLPTM1	6	6	3	3	4	4	4.30E+06	2.00E+06	1.90E+06	1.90E+06	2.3	1.1	1.0
ITGA1	4	4	6	6	3	3	2.30E+06	1.80E+06	7.00E+05	7.00E+05	3.3	2.6	1.0
SEC24C	3	3	4	4	4	6	9.40E+05	9.90E+05	1.30E+06	9.40E+05	1.0	1.1	1.4
IDH2	6	6	3	3	3	3	2.00E+06	8.50E+05	7.70E+05	7.70E+05	2.6	1.1	1.0
ABCF1	4	4	5	5	3	3	1.40E+06	1.40E+06	5.10E+05	5.10E+05	2.7	2.7	1.0
TM9SF2	4	4	4	5	3	3	3.50E+06	1.80E+06	9.10E+05	9.10E+05	3.8	2.0	1.0
AAAS	5	5	3	3	4	4	2.10E+06	2.00E+06	1.50E+06	1.50E+06	1.4	1.3	1.0
LAMTOR1	2	2	3	3	6	7	1.30E+06	9.50E+05	1.40E+06	9.50E+05	1.4	1.0	1.5
AXL	4	4	5	5	3	3	1.40E+06	2.10E+06	8.80E+05	8.80E+05	1.6	2.4	1.0
TMED10	5	5	3	3	4	4	3.40E+06	1.30E+06	1.10E+06	1.10E+06	3.1	1.2	1.0
DNM2	2	2	6	6	3	4	3.50E+05	1.80E+06	5.40E+05	3.50E+05	1.0	5.1	1.5
TMEM192	3	4	2	2	5	6	9.30E+06	4.90E+06	1.00E+07	4.90E+06	1.9	1.0	2.0
TOP1	3	3	3	3	6	6	2.10E+06	2.60E+06	6.10E+06	2.10E+06	1.0	1.2	2.9
STT3A	4	4	5	5	3	3	7.70E+06	5.00E+06	2.80E+06	2.80E+06	2.8	1.8	1.0
ACSF3	5	5	3	3	2	3	1.80E+06	1.00E+06	3.80E+04	3.80E+04	47.4	26.3	1.0
GOLIM4	4	4	3	3	4	4	6.60E+06	4.30E+06	4.60E+06	4.30E+06	1.5	1.0	1.1
TARS2	4	4	3	3	4	4	1.50E+06	6.20E+05	1.20E+06	6.20E+05	2.4	1.0	1.9
ATP6V1A	5	5	3	3	3	3	2.00E+06	7.40E+05	1.50E+06	7.40E+05	2.7	1.0	2.0
TRIP11	3	3	4	4	4	4	1.10E+06	3.40E+06	2.20E+06	1.10E+06	1.0	3.1	2.0
ERO1A	3	3	4	4	4	4	3.10E+06	2.70E+06	1.70E+06	1.70E+06	1.8	1.6	1.0
ZC3H15	6	6	3	3	2	2	9.00E+06	1.70E+06	1.60E+06	1.60E+06	5.6	1.1	1.0
PODXL	3	3	4	4	4	4	8.30E+06	9.50E+06	7.50E+06	7.50E+06	1.1	1.3	1.0
SSRP1	3	3	3	3	5	5	1.40E+06	2.20E+06	1.70E+06	1.40E+06	1.0	1.6	1.2
PIK3C2A	5	5	3	3	3	3	1.60E+06	4.60E+05	3.80E+05	3.80E+05	4.2	1.2	1.0
MRPL19	3	3	4	4	4	4	2.30E+06	4.20E+06	3.30E+06	2.30E+06	1.0	1.8	1.4
CRTAP	4	4	4	4	3	3	2.20E+06	2.20E+06	7.40E+05	7.40E+05	3.0	3.0	1.0
RAB9A	5	5	2	3	2	2	3.90E+06	2.00E+06	7.10E+05	7.10E+05	5.5	2.8	1.0
MRPS5	5	5	2	2	3	3	1.30E+06	5.00E+04	4.70E+05	5.00E+04	26.0	1.0	9.4
NPR3	2	2	5	5	3	3	1.10E+06	3.00E+06	1.50E+06	1.10E+06	1.0	2.7	1.4
INF2	4	4	3	3	3	3	1.00E+06	8.40E+05	7.80E+05	7.80E+05	1.3	1.1	1.0
COPB1	3	3	4	4	3	3	8.00E+05	1.20E+06	5.90E+05	5.90E+05	1.4	2.0	1.0
TBL2	4	4	2	2	4	4	3.20E+06	1.80E+06	3.70E+06	1.80E+06	1.8	1.0	2.1

GLG1	4	4	4	4	2	2	4.70E+06	3.50E+06	1.10E+06	1.10E+06	4.3	3.2	1.0
EIF2A	4	4	4	4	2	2	1.80E+06	1.50E+06	5.00E+05	5.00E+05	3.6	3.0	1.0
MRPL41	5	5	0	0	4	4	4.70E+06	NF	1.00E+06	1.00E+06	4.7	0.0	1.0
DDX1	5	5	2	2	2	2	9.90E+05	7.60E+05	4.60E+05	4.60E+05	2.2	1.7	1.0
PLXNB2	5	5	4	4	0	0	1.80E+06	2.30E+06	NF	1.80E+06	1.0	1.3	0.0
SVIL	0	0	5	5	2	2	NF	1.50E+06	3.40E+05	3.40E+05	0.0	4.4	1.0
EIF3D	2	2	4	4	0	0	3.40E+05	1.20E+06	NF	3.40E+05	1.0	3.5	0.0
YBX1	4	5	5	10	5	11	5.70E+06	8.10E+06	5.90E+06	5.70E+06	1.0	1.4	1.0
SLC7A5	5	8	4	8	4	8	8.50E+07	6.50E+07	4.10E+07	4.10E+07	2.1	1.6	1.0
TUBB8B	5	7	4	10	3	7	1.20E+07	1.90E+07	8.40E+06	8.40E+06	1.4	2.3	1.0
RPS8	5	8	4	7	4	6	3.20E+07	2.50E+07	2.00E+07	2.00E+07	1.6	1.3	1.0
RPL18	4	6	5	7	4	5	3.00E+07	2.20E+07	1.30E+07	1.30E+07	2.3	1.7	1.0
RAB5A	5	6	4	7	4	5	5.10E+06	1.00E+07	5.60E+06	5.10E+06	1.0	2.0	1.1
PLD3	5	5	5	6	5	6	6.10E+06	4.80E+06	4.40E+06	4.40E+06	1.4	1.1	1.0
DNAJA3	5	7	4	5	5	5	4.60E+06	3.20E+06	2.20E+06	2.20E+06	2.1	1.5	1.0
BCKDHA	4	5	4	4	5	7	1.20E+07	3.40E+06	1.40E+06	1.40E+06	8.6	2.4	1.0
MRPL22	4	5	5	6	4	5	5.80E+06	1.20E+07	3.20E+06	3.20E+06	1.8	3.8	1.0
SYNPO	5	6	4	5	4	5	4.80E+06	2.90E+06	1.20E+06	1.20E+06	4.0	2.4	1.0
H1-4	5	5	5	5	5	6	1.60E+07	1.90E+07	2.00E+07	1.60E+07	1.0	1.2	1.3
MRPS7	4	6	5	6	4	4	4.30E+06	2.30E+06	1.60E+06	1.60E+06	2.7	1.4	1.0
TMEM126A	4	6	2	3	3	6	1.40E+06	4.20E+04	2.70E+05	4.20E+04	33.3	1.0	6.4
DRG1	4	4	4	6	5	5	1.70E+06	2.30E+06	9.60E+05	9.60E+05	1.8	2.4	1.0
PRDX3	5	5	4	5	5	5	6.90E+06	6.30E+06	3.30E+06	3.30E+06	2.1	1.9	1.0
MCAT	5	5	4	5	4	4	2.20E+06	2.00E+06	1.30E+06	1.30E+06	1.7	1.5	1.0
VASN	4	4	5	5	4	5	2.50E+06	3.10E+06	1.50E+06	1.50E+06	1.7	2.1	1.0
ARFGAP1	2	2	4	5	4	7	1.10E+06	1.40E+06	2.40E+06	1.10E+06	1.0	1.3	2.2
RCC1L	4	4	5	5	4	5	1.60E+06	1.60E+06	1.00E+06	1.00E+06	1.6	1.6	1.0
DPM1	5	7	3	3	4	4	3.20E+06	1.60E+06	1.30E+06	1.30E+06	2.5	1.2	1.0
CACNA2D1	4	5	4	5	3	3	1.30E+06	1.10E+06	4.20E+05	4.20E+05	3.1	2.6	1.0
AGK	4	4	4	4	5	5	4.30E+06	2.40E+06	2.70E+06	2.40E+06	1.8	1.0	1.1
NUP155	4	4	4	4	5	5	2.20E+06	1.70E+06	1.50E+06	1.50E+06	1.5	1.1	1.0
CTNNA2	5	5	3	3	4	5	5.30E+06	4.10E+06	3.60E+06	3.60E+06	1.5	1.1	1.0
DDX17	4	4	4	6	3	3	2.50E+06	4.70E+06	1.60E+06	1.60E+06	1.6	2.9	1.0
COL1A1	3	3	5	5	4	5	4.20E+06	4.60E+06	2.80E+06	2.80E+06	1.5	1.6	1.0
MRPL44	5	5	4	4	4	4	1.90E+06	1.60E+06	1.10E+06	1.10E+06	1.7	1.5	1.0
ELAVL1	4	5	3	3	4	5	3.60E+06	1.80E+06	2.30E+06	1.80E+06	2.0	1.0	1.3
ALDOA	2	2	5	8	3	3	1.80E+06	4.80E+06	1.00E+06	1.00E+06	1.8	4.8	1.0
HNRNPC	4	6	5	7	0	0	7.30E+06	1.80E+07	NF	7.30E+06	1.0	2.5	0.0
RPS13	4	5	4	4	4	4	8.00E+06	6.80E+06	5.00E+06	5.00E+06	1.6	1.4	1.0
TCP1	3	3	5	5	5	5	1.80E+06	3.30E+06	1.60E+06	1.60E+06	1.1	2.1	1.0
RPS25	3	3	5	7	3	3	1.20E+07	1.60E+07	7.20E+06	7.20E+06	1.7	2.2	1.0
CCDC51	4	4	4	4	5	5	3.80E+06	2.80E+06	2.80E+06	2.80E+06	1.4	1.0	1.0
FAF2	4	4	5	5	4	4	6.60E+06	6.90E+06	4.20E+06	4.20E+06	1.6	1.6	1.0
NUP93	2	3	4	5	4	5	1.90E+06	3.20E+06	1.80E+06	1.80E+06	1.1	1.8	1.0
ERGIC1	3	4	4	5	3	4	1.70E+06	2.30E+06	1.90E+06	1.70E+06	1.0	1.4	1.1
ATP6V1B2	5	5	3	4	3	3	3.20E+06	1.90E+06	1.40E+06	1.40E+06	2.3	1.4	1.0
VAT1	3	3	4	4	5	5	2.20E+06	1.70E+06	1.40E+06	1.40E+06	1.6	1.2	1.0
HSD17B12	3	3	4	4	5	5	1.30E+06	2.30E+06	2.00E+06	1.30E+06	1.0	1.8	1.5
POLDIP2	5	5	3	4	3	3	5.20E+06	3.40E+06	2.10E+06	2.10E+06	2.5	1.6	1.0
DAG1	4	5	3	4	3	3	1.80E+06	2.00E+06	8.90E+05	8.90E+05	2.0	2.2	1.0
SUCLG1	5	5	2	3	3	4	5.40E+06	4.20E+06	3.90E+06	3.90E+06	1.4	1.1	1.0

RUFY1	3	4	4	4	4	4	5.80E+05	1.10E+06	3.10E+05	3.10E+05	1.9	3.5	1.0
EIF2S2	3	3	5	5	4	4	1.90E+06	4.00E+06	1.70E+06	1.70E+06	1.1	2.4	1.0
FOXRED1	3	4	3	3	4	5	2.00E+06	1.50E+06	2.30E+06	1.50E+06	1.3	1.0	1.5
PLBD2	5	5	3	3	3	4	1.50E+06	1.10E+06	7.40E+05	7.40E+05	2.0	1.5	1.0
EIF3G	4	4	4	4	2	4	4.90E+06	7.50E+05	5.00E+05	5.00E+05	9.8	1.5	1.0
MRPS31	3	3	5	5	4	4	9.90E+05	2.30E+06	1.10E+06	9.90E+05	1.0	2.3	1.1
RAB11A	3	3	4	4	5	5	6.30E+06	5.40E+06	5.00E+06	5.00E+06	1.3	1.1	1.0
SSR1	3	3	4	4	5	5	3.70E+06	2.90E+06	2.50E+06	2.50E+06	1.5	1.2	1.0
TFB2M	2	2	5	6	3	4	1.80E+06	2.60E+06	1.70E+06	1.70E+06	1.1	1.5	1.0
PPP1CC	5	5	5	5	2	2	2.90E+06	1.60E+06	5.70E+05	5.70E+05	5.1	2.8	1.0
COPG1	2	3	4	4	5	5	1.90E+06	2.70E+06	3.30E+06	1.90E+06	1.0	1.4	1.7
ANO6	2	2	5	6	4	4	5.30E+05	1.40E+06	6.70E+05	5.30E+05	1.0	2.6	1.3
SLC7A2	2	2	4	5	5	5	1.40E+06	2.50E+06	1.90E+06	1.40E+06	1.0	1.8	1.4
SCARB1	4	4	3	3	3	5	6.80E+06	5.80E+06	7.10E+06	5.80E+06	1.2	1.0	1.2
GNAI3	4	4	3	4	3	3	3.20E+06	5.60E+06	2.40E+06	2.40E+06	1.3	2.3	1.0
ATP6V0A2	4	4	3	3	4	4	9.30E+05	9.00E+05	6.80E+05	6.80E+05	1.4	1.3	1.0
HLA-B	3	3	4	4	4	4	2.90E+06	3.40E+06	2.50E+06	2.50E+06	1.2	1.4	1.0
ECI2	4	4	4	4	3	3	1.60E+06	1.10E+06	7.30E+05	7.30E+05	2.2	1.5	1.0
SAFB2	3	3	4	4	4	4	9.00E+05	2.10E+06	1.20E+06	9.00E+05	1.0	2.3	1.3
ABCD1	4	4	3	3	4	4	2.20E+06	1.50E+06	1.40E+06	1.40E+06	1.6	1.1	1.0
QSOX2	4	4	4	5	2	2	1.40E+06	8.50E+05	3.50E+05	3.50E+05	4.0	2.4	1.0
EPHB4	3	3	4	4	3	4	6.30E+05	1.60E+06	1.10E+06	6.30E+05	1.0	2.5	1.7
TAMM41	4	4	3	3	4	4	2.90E+06	3.20E+06	2.20E+06	2.20E+06	1.3	1.5	1.0
ECI1	5	6	2	2	3	3	4.80E+06	1.40E+06	2.00E+06	1.40E+06	3.4	1.0	1.4
ARCN1	4	5	3	3	3	3	7.50E+05	8.70E+05	4.10E+05	4.10E+05	1.8	2.1	1.0
FUS	3	3	4	4	4	4	1.30E+06	3.30E+06	1.70E+06	1.30E+06	1.0	2.5	1.3
TMEM33	5	5	3	3	3	3	8.00E+06	6.50E+06	3.60E+06	3.60E+06	2.2	1.8	1.0
EPHA2	2	2	5	5	4	4	7.30E+05	2.60E+06	1.10E+06	7.30E+05	1.0	3.6	1.5
ARF4	5	5	3	3	3	3	3.10E+06	1.90E+06	1.30E+06	1.30E+06	2.4	1.5	1.0
SLC25A10	3	3	4	4	4	4	5.50E+06	4.30E+06	2.90E+06	2.90E+06	1.9	1.5	1.0
ARMCX3	5	5	3	3	3	3	2.20E+06	1.50E+06	1.10E+06	1.10E+06	2.0	1.4	1.0
PRCP	4	4	2	2	4	5	3.30E+06	1.70E+06	2.50E+06	1.70E+06	1.9	1.0	1.5
PDLIM7	5	5	4	4	2	2	7.60E+06	5.10E+06	1.30E+06	1.30E+06	5.8	3.9	1.0
TMTC3	3	4	4	4	2	3	1.40E+06	5.70E+05	9.80E+04	9.80E+04	14.3	5.8	1.0
P3H3	3	4	3	3	4	4	9.30E+05	8.20E+05	6.20E+05	6.20E+05	1.5	1.3	1.0
ABHD10	5	5	1	1	4	4	2.10E+06	3.80E+05	1.20E+06	3.80E+05	5.5	1.0	3.2
GAA	3	3	2	3	3	4	4.20E+05	6.00E+05	2.60E+06	4.20E+05	1.0	1.4	6.2
ACAA1	3	3	3	3	4	4	2.60E+05	1.20E+05	5.90E+05	1.20E+05	2.2	1.0	4.9
IKBIP	3	3	4	4	3	3	4.10E+06	4.50E+06	4.10E+06	4.10E+06	1.0	1.1	1.0
MANBA	3	4	2	2	4	4	1.30E+06	3.90E+05	4.80E+05	3.90E+05	3.3	1.0	1.2
NT5DC2	4	4	4	4	2	2	2.10E+06	1.20E+06	8.20E+05	8.20E+05	2.6	1.5	1.0
BCS1L	3	3	4	4	3	3	1.10E+06	3.50E+06	1.60E+06	1.10E+06	1.0	3.2	1.5
MICU1	4	4	3	3	3	3	1.30E+06	8.00E+05	6.20E+05	6.20E+05	2.1	1.3	1.0
IGF2BP3	2	2	4	4	4	4	7.40E+05	7.80E+05	1.60E+06	7.40E+05	1.0	1.1	2.2
EIF3E	2	2	5	5	2	3	1.90E+06	3.40E+06	2.10E+06	1.90E+06	1.0	1.8	1.1
DNAJA1	3	3	2	3	3	4	3.10E+06	6.30E+06	6.40E+06	3.10E+06	1.0	2.0	2.1
CISD2	4	4	3	3	3	3	2.30E+06	1.80E+06	1.20E+06	1.20E+06	1.9	1.5	1.0
DIAPH3	4	4	3	3	2	2	4.40E+05	4.40E+05	1.50E+05	1.50E+05	2.9	2.9	1.0
MFF	4	4	3	3	2	2	2.30E+06	7.80E+05	3.20E+05	3.20E+05	7.2	2.4	1.0
MCU	4	4	3	3	2	2	2.90E+06	1.20E+06	4.60E+05	4.60E+05	6.3	2.6	1.0
SOD2	4	4	2	2	3	3	3.80E+06	1.90E+06	2.20E+06	1.90E+06	2.0	1.0	1.2

ATP6AP1	3	3	2	2	4	4	1.20E+06	4.60E+05	1.10E+06	4.60E+05	2.6	1.0	2.4
LARP1	3	3	4	4	2	2	2.30E+06	1.60E+06	5.90E+05	5.90E+05	3.9	2.7	1.0
LGALS3BP	5	5	2	2	2	2	1.60E+06	4.70E+05	3.90E+05	3.90E+05	4.1	1.2	1.0
GRPEL1	0	0	4	4	4	5	NF	2.10E+06	2.50E+06	2.10E+06	0.0	1.0	1.2
ATP13A1	3	3	3	3	2	3	1.20E+06	8.40E+05	3.90E+05	3.90E+05	3.1	2.2	1.0
EDIL3	4	4	2	2	3	3	2.20E+06	9.90E+05	1.00E+06	9.90E+05	2.2	1.0	1.0
FAM120A	4	4	2	2	3	3	1.30E+06	3.70E+05	8.30E+05	3.70E+05	3.5	1.0	2.2
TFAM	3	3	3	4	2	2	2.40E+06	4.40E+06	2.00E+06	2.00E+06	1.2	2.2	1.0
LRRC8A	4	4	2	2	3	3	1.90E+06	1.10E+06	6.50E+05	6.50E+05	2.9	1.7	1.0
CALD1	2	2	4	4	2	2	1.20E+06	2.80E+06	8.40E+05	8.40E+05	1.4	3.3	1.0
MFN2	3	3	1	1	3	4	1.40E+06	6.90E+05	6.00E+06	6.90E+05	2.0	1.0	8.7
LDHB	2	2	3	3	3	3	1.10E+06	1.80E+06	1.10E+06	1.10E+06	1.0	1.6	1.0
TMEM132A	4	4	4	4	0	0	1.60E+06	1.00E+06	NF	1.00E+06	1.6	1.0	0.0
HEXB	4	4	2	2	2	2	2.80E+06	1.10E+06	3.00E+05	3.00E+05	9.3	3.7	1.0
PTPRJ	2	2	4	4	2	2	8.10E+05	1.60E+06	2.70E+05	2.70E+05	3.0	5.9	1.0
PLIN3	0	0	5	5	2	2	NF	1.50E+06	5.00E+05	5.00E+05	0.0	3.0	1.0
RPL23	5	5	0	0	2	2	4.50E+06	NF	5.20E+06	4.50E+06	1.0	0.0	1.2
PIK3R4	0	0	4	4	3	3	NF	9.10E+05	3.80E+05	3.80E+05	0.0	2.4	1.0
P3H1	3	3	2	2	2	2	1.50E+06	6.10E+05	5.30E+05	5.30E+05	2.8	1.2	1.0
TRMT10C	0	0	2	2	5	5	NF	1.50E+06	3.30E+06	1.50E+06	0.0	1.0	2.2
PTPN1	4	4	2	2	1	1	3.30E+06	1.20E+06	4.30E+05	4.30E+05	7.7	2.8	1.0
MCM5	3	3	3	3	1	1	5.30E+05	8.90E+06	8.50E+04	8.50E+04	6.2	####	1.0
SNRNP200	3	3	2	2	1	2	4.40E+05	1.40E+05	1.40E+04	1.40E+04	31.4	10.0	1.0
MYO19	4	4	1	1	1	1	1.80E+06	3.50E+05	3.10E+05	3.10E+05	5.8	1.1	1.0
KIDINS220	3	3	1	1	2	2	5.10E+05	7.00E+04	3.00E+05	7.00E+04	7.3	1.0	4.3
MYCBP2	0	0	5	5	1	1	NF	1.10E+06	6.70E+03	6.70E+03	0.0	####	1.0
ITPR1	3	3	1	1	2	2	1.10E+06	1.40E+05	5.70E+05	1.40E+05	7.9	1.0	4.1
UGGT2	3	3	0	0	2	2	5.00E+05	NF	5.90E+05	5.00E+05	1.0	0.0	1.2
ATP11A	2	2	3	3	0	0	9.80E+05	1.40E+06	NF	9.80E+05	1.0	1.4	0.0
TTN	3	3	2	2	0	0	1.50E+06	4.30E+05	NF	4.30E+05	3.5	1.0	0.0
RPS10	4	8	4	6	3	6	1.00E+07	5.90E+06	2.60E+06	2.60E+06	3.8	2.3	1.0
UBB	4	5	4	8	4	7	2.10E+07	2.50E+07	2.00E+07	2.00E+07	1.1	1.3	1.0
SSR4	4	6	4	6	4	7	6.80E+06	5.50E+06	3.40E+06	3.40E+06	2.0	1.6	1.0
CAD	3	4	3	5	4	9	1.00E+08	1.10E+08	1.20E+08	1.00E+08	1.0	1.1	1.2
MRPL28	4	6	3	4	3	6	6.50E+05	1.00E+06	1.60E+06	6.50E+05	1.0	1.5	2.5
YWHAZ	3	3	4	7	4	5	2.50E+06	5.20E+06	1.80E+06	1.80E+06	1.4	2.9	1.0
ALDH3A2	4	6	4	4	4	5	2.50E+06	1.20E+06	1.00E+06	1.00E+06	2.5	1.2	1.0
RPL24	4	5	4	5	3	5	8.50E+06	5.50E+06	4.50E+06	4.50E+06	1.9	1.2	1.0
SLC25A4	4	6	3	4	4	5	5.90E+07	4.20E+07	3.50E+07	3.50E+07	1.7	1.2	1.0
SLC38A2	4	4	4	4	4	6	1.40E+07	1.20E+07	9.50E+06	9.50E+06	1.5	1.3	1.0
CCT7	4	4	4	6	4	4	2.30E+06	3.60E+06	2.00E+06	2.00E+06	1.2	1.8	1.0
SDCBP	3	4	4	5	4	4	1.20E+06	1.50E+06	1.10E+06	1.10E+06	1.1	1.4	1.0
OCIAD1	3	3	3	4	4	6	4.20E+06	1.20E+06	5.90E+06	1.20E+06	3.5	1.0	4.9
CHCHD3	4	4	4	4	4	5	8.80E+06	7.50E+06	7.30E+06	7.30E+06	1.2	1.0	1.0
TMPO	4	4	4	4	4	5	3.40E+06	2.60E+06	1.60E+06	1.60E+06	2.1	1.6	1.0
PCBP2	4	5	2	4	2	4	3.70E+06	5.50E+06	3.30E+06	3.30E+06	1.1	1.7	1.0
MRM3	3	4	4	6	3	3	3.40E+06	4.90E+06	1.30E+06	1.30E+06	2.6	3.8	1.0
MRPS30	3	3	3	5	2	4	9.80E+05	6.00E+05	4.50E+05	4.50E+05	2.2	1.3	1.0
ECE1	2	3	2	4	3	5	2.10E+06	1.20E+06	1.40E+06	1.20E+06	1.8	1.0	1.2
HNRNPH1	2	2	4	5	3	5	1.30E+06	3.00E+06	2.00E+06	1.30E+06	1.0	2.3	1.5
SAR1A	4	4	4	5	3	3	2.30E+06	2.00E+06	7.20E+05	7.20E+05	3.2	2.8	1.0

DAB2	3	3	4	4	4	5	2.60E+06	2.40E+06	2.70E+06	2.40E+06	1.1	1.0	1.1
NAPA	3	4	3	5	3	3	1.80E+06	8.70E+05	5.10E+05	5.10E+05	3.5	1.7	1.0
ALDH1L2	3	3	4	5	4	4	1.70E+06	1.40E+07	1.40E+06	1.40E+06	1.2	10.0	1.0
LAMP2	3	4	4	4	3	4	6.00E+06	1.60E+06	2.20E+06	1.60E+06	3.8	1.0	1.4
MAN1B1	4	4	4	4	4	4	3.90E+06	2.10E+06	2.00E+06	2.00E+06	2.0	1.1	1.0
SLC25A1	3	4	2	3	4	5	7.70E+06	5.00E+06	1.50E+07	5.00E+06	1.5	1.0	3.0
VPS45	4	5	3	3	3	4	1.30E+06	9.50E+05	4.40E+05	4.40E+05	3.0	2.2	1.0
EXD2	4	4	3	4	3	3	1.90E+06	6.50E+05	4.30E+05	4.30E+05	4.4	1.5	1.0
RPL9P9	3	3	4	4	3	4	2.70E+06	3.00E+06	1.40E+06	1.40E+06	1.9	2.1	1.0
SRPRB	4	4	3	3	3	4	3.90E+06	3.10E+06	3.40E+06	3.10E+06	1.3	1.0	1.1
YBX3	3	3	4	4	4	4	3.50E+06	5.00E+06	3.60E+06	3.50E+06	1.0	1.4	1.0
SQSTM1	2	2	4	5	3	4	3.10E+06	7.60E+06	4.90E+06	3.10E+06	1.0	2.5	1.6
PLS3	4	4	4	4	3	3	2.50E+06	2.20E+06	1.10E+06	1.10E+06	2.3	2.0	1.0
SDC4	3	4	4	4	3	3	3.10E+06	4.60E+06	1.30E+06	1.30E+06	2.4	3.5	1.0
FLVCR1	3	4	3	3	3	4	1.20E+06	7.80E+05	6.00E+05	6.00E+05	2.0	1.3	1.0
RHOG	3	4	3	3	4	4	4.00E+06	2.00E+06	1.40E+06	1.40E+06	2.9	1.4	1.0
H1-5	3	3	4	5	2	3	6.90E+06	1.20E+07	5.10E+06	5.10E+06	1.4	2.4	1.0
SCP2	2	3	3	4	3	4	4.90E+06	3.90E+06	1.10E+06	1.10E+06	4.5	3.5	1.0
TUBA1B	2	2	4	5	2	3	4.80E+06	5.50E+06	1.70E+06	1.70E+06	2.8	3.2	1.0
ISOC2	3	4	3	4	2	2	7.90E+05	2.30E+05	4.80E+04	4.80E+04	16.5	4.8	1.0
SPTLC1	4	4	3	3	3	3	2.60E+06	1.90E+06	1.50E+06	1.50E+06	1.7	1.3	1.0
RDH11	3	3	3	3	4	4	3.40E+06	3.30E+06	3.30E+06	3.30E+06	1.0	1.0	1.0
ETFDH	4	4	3	3	3	3	3.00E+06	1.40E+06	1.30E+06	1.30E+06	2.3	1.1	1.0
TRIM28	2	2	3	5	3	3	3.00E+05	1.20E+06	3.90E+05	3.00E+05	1.0	4.0	1.3
B4GALT1	3	4	3	3	3	3	1.10E+06	8.40E+05	7.40E+05	7.40E+05	1.5	1.1	1.0
SLC35A4	2	2	4	4	4	4	1.90E+06	1.60E+06	1.10E+06	1.10E+06	1.7	1.5	1.0
RPS24	3	4	3	3	3	3	2.00E+06	1.30E+06	7.20E+05	7.20E+05	2.8	1.8	1.0
SDC2	3	4	4	4	2	2	1.10E+06	2.20E+06	9.60E+05	9.60E+05	1.1	2.3	1.0
GTPBP6	3	3	4	4	3	3	7.40E+05	5.60E+05	3.90E+05	3.90E+05	1.9	1.4	1.0
MRPL14	3	3	3	3	4	4	3.50E+06	2.50E+06	1.30E+07	2.50E+06	1.4	1.0	5.2
CAND1	4	4	3	3	3	3	7.70E+05	7.20E+05	3.50E+05	3.50E+05	2.2	2.1	1.0
HARS2	3	3	2	2	3	5	2.20E+06	1.70E+06	4.00E+06	1.70E+06	1.3	1.0	2.4
VAMP7	3	3	3	4	3	3	1.10E+06	2.10E+06	7.10E+05	7.10E+05	1.5	3.0	1.0
SLC16A1	3	3	3	3	3	4	9.90E+06	5.10E+06	8.40E+06	5.10E+06	1.9	1.0	1.6
PNPLA6	3	4	4	4	2	2	1.30E+06	4.10E+05	2.60E+05	2.60E+05	5.0	1.6	1.0
PRDX4	4	4	3	3	3	3	1.20E+07	5.70E+06	4.90E+06	4.90E+06	2.4	1.2	1.0
HTRA2	4	4	3	3	3	3	1.40E+06	7.60E+05	7.70E+05	7.60E+05	1.8	1.0	1.0
PPA2	3	3	3	3	3	4	4.60E+06	5.40E+06	7.00E+06	4.60E+06	1.0	1.2	1.5
ASAH1	3	3	2	3	3	4	1.50E+06	2.30E+06	1.30E+06	1.30E+06	1.2	1.8	1.0
RAB32	2	2	4	4	3	4	1.10E+06	1.70E+06	1.40E+06	1.10E+06	1.0	1.5	1.3
FASTKD3	3	3	2	3	3	4	7.60E+05	3.70E+05	3.70E+05	3.70E+05	2.1	1.0	1.0
RPL10A	4	5	2	2	3	3	7.50E+06	3.00E+06	2.20E+06	2.20E+06	3.4	1.4	1.0
ACP2	4	4	3	3	3	3	3.10E+06	1.20E+06	1.00E+06	1.00E+06	3.1	1.2	1.0
NRAS	3	3	4	4	3	3	2.30E+06	2.40E+06	1.40E+06	1.40E+06	1.6	1.7	1.0
GHITM	3	3	3	4	2	3	5.20E+06	7.40E+06	5.50E+06	5.20E+06	1.0	1.4	1.1
PTRH2	3	3	4	4	2	2	1.70E+06	1.00E+06	5.60E+05	5.60E+05	3.0	1.8	1.0
FAT1	0	0	4	5	2	4	NF	4.10E+05	2.30E+05	2.30E+05	0.0	1.8	1.0
PTPRS	3	3	4	5	1	1	4.20E+05	7.90E+05	6.60E+04	6.60E+04	6.4	12.0	1.0
ATP1B3	2	2	4	4	2	3	4.80E+06	4.20E+06	4.40E+06	4.20E+06	1.1	1.0	1.0
COL18A1	4	5	2	2	2	2	1.60E+06	8.20E+05	1.00E+06	8.20E+05	2.0	1.0	1.2
FYN	4	5	2	2	1	2	2.20E+06	1.70E+05	9.10E+04	9.10E+04	24.2	1.9	1.0

MRPS23	4	4	2	2	3	3	5.10E+06	1.30E+06	1.70E+06	1.30E+06	3.9	1.0	1.3
ERAP1	3	3	3	3	3	3	6.80E+05	3.70E+05	2.60E+05	2.60E+05	2.6	1.4	1.0
SNX9	2	2	3	4	2	3	9.60E+04	1.60E+06	2.40E+05	9.60E+04	1.0	16.7	2.5
RUVBL2	3	3	4	4	2	2	1.10E+06	1.80E+06	3.70E+05	3.70E+05	3.0	4.9	1.0
STX7	3	3	3	3	3	3	2.10E+06	1.80E+06	1.40E+06	1.40E+06	1.5	1.3	1.0
EMC2	2	2	2	2	4	5	6.80E+05	5.30E+05	1.10E+06	5.30E+05	1.3	1.0	2.1
FAM162A	4	4	3	3	2	2	6.60E+06	3.70E+06	3.10E+06	3.10E+06	2.1	1.2	1.0
FASTKD1	3	3	2	2	4	4	1.20E+06	4.00E+05	8.20E+05	4.00E+05	3.0	1.0	2.1
GLT8D1	3	3	3	3	3	3	8.30E+05	6.60E+05	3.90E+05	3.90E+05	2.1	1.7	1.0
LMAN1	2	2	2	2	4	5	2.20E+06	1.80E+06	3.90E+06	1.80E+06	1.2	1.0	2.2
PRDX1	2	2	4	4	3	3	4.50E+06	5.10E+06	3.20E+06	3.20E+06	1.4	1.6	1.0
HARS1	3	3	2	2	4	4	1.60E+06	1.30E+06	2.20E+06	1.30E+06	1.2	1.0	1.7
SLC16A3	2	2	3	3	4	4	2.00E+06	7.10E+06	5.80E+06	2.00E+06	1.0	3.6	2.9
ATPAF1	4	4	3	3	2	2	2.30E+06	1.10E+06	8.20E+05	8.20E+05	2.8	1.3	1.0
SEC61A1	3	4	2	2	3	3	4.40E+06	2.70E+06	3.20E+06	2.70E+06	1.6	1.0	1.2
MRPS34	4	4	3	3	2	2	3.90E+06	1.70E+06	7.00E+05	7.00E+05	5.6	2.4	1.0
DAGLB	3	3	2	2	3	3	3.30E+05	2.10E+05	2.90E+05	2.10E+05	1.6	1.0	1.4
ARMCX2	2	2	3	3	3	3	1.30E+06	6.40E+05	5.50E+05	5.50E+05	2.4	1.2	1.0
RTN4	2	2	3	4	2	2	5.30E+05	2.00E+06	1.80E+05	1.80E+05	2.9	11.1	1.0
NSUN2	4	4	2	2	2	2	9.10E+05	3.80E+05	1.80E+05	1.80E+05	5.1	2.1	1.0
APOO	4	4	2	2	2	2	5.20E+06	3.20E+06	2.30E+06	2.30E+06	2.3	1.4	1.0
UPF1	4	4	2	2	2	2	3.10E+06	1.80E+06	7.30E+05	7.30E+05	4.2	2.5	1.0
SDC1	1	1	4	4	3	3	2.30E+06	6.30E+06	4.50E+06	2.30E+06	1.0	2.7	2.0
EIF4A3	4	4	2	2	2	2	2.80E+06	1.20E+06	5.50E+05	5.50E+05	5.1	2.2	1.0
DHCR7	4	4	2	2	2	2	2.90E+06	2.30E+06	1.20E+06	1.20E+06	2.4	1.9	1.0
HTATIP2	4	4	2	2	2	2	3.70E+06	1.60E+06	1.10E+06	1.10E+06	3.4	1.5	1.0
RAB8A	2	2	3	3	3	3	8.50E+05	1.80E+06	1.50E+06	8.50E+05	1.0	2.1	1.8
AP3D1	3	3	3	3	2	2	6.80E+05	7.60E+05	2.20E+05	2.20E+05	3.1	3.5	1.0
EIF2S1	1	1	3	3	4	4	1.50E+06	2.00E+06	1.90E+06	1.50E+06	1.0	1.3	1.3
LARP4	2	2	2	2	3	4	1.00E+06	6.70E+05	7.20E+05	6.70E+05	1.5	1.0	1.1
TMEM214	3	3	3	3	2	2	1.40E+06	1.40E+06	4.80E+05	4.80E+05	2.9	2.9	1.0
RPL27	3	3	2	2	3	3	5.70E+06	3.50E+06	2.90E+06	2.90E+06	2.0	1.2	1.0
CTTN	3	3	3	3	1	2	1.30E+06	8.10E+05	5.30E+05	5.30E+05	2.5	1.5	1.0
TECR	4	4	2	2	2	2	3.40E+06	2.70E+06	2.10E+06	2.10E+06	1.6	1.3	1.0
GTPBP4	1	1	3	4	3	3	2.40E+05	1.10E+06	3.60E+05	2.40E+05	1.0	4.6	1.5
NUP160	1	1	3	3	3	4	7.60E+03	1.20E+06	9.30E+05	7.60E+03	1.0	####	####
G3BP2	2	2	2	2	3	3	1.20E+06	2.50E+05	1.30E+06	2.50E+05	4.8	1.0	5.2
ENG	3	3	2	2	2	2	5.50E+06	5.50E+06	3.60E+05	3.60E+05	15.3	15.3	1.0
NDUFA10	2	3	0	0	4	4	4.00E+06	NF	1.80E+06	1.80E+06	2.2	0.0	1.0
PYCR2	3	3	2	2	2	2	3.10E+06	1.00E+06	1.40E+06	1.00E+06	3.1	1.0	1.4
ABCD3	3	3	2	2	2	2	3.90E+06	1.50E+06	1.10E+06	1.10E+06	3.5	1.4	1.0
NAGLU	3	4	2	2	1	1	7.00E+05	2.90E+05	2.70E+05	2.70E+05	2.6	1.1	1.0
PRKAR2A	2	2	3	3	2	2	3.60E+05	4.30E+05	4.10E+05	3.60E+05	1.0	1.2	1.1
DDX6	3	3	2	2	2	2	7.90E+05	4.20E+05	2.30E+05	2.30E+05	3.4	1.8	1.0
TARDBP	4	4	1	1	2	2	2.90E+06	2.30E+05	9.10E+05	2.30E+05	12.6	1.0	4.0
GPAM	3	3	2	2	2	2	4.90E+05	1.80E+05	1.60E+05	1.60E+05	3.1	1.1	1.0
TCIRG1	3	4	0	0	3	3	2.10E+06	NF	9.70E+05	9.70E+05	2.2	0.0	1.0
THNSL1	3	3	3	3	1	1	3.00E+05	4.20E+05	1.00E+05	1.00E+05	3.0	4.2	1.0
ANXA1	1	1	3	3	3	3	3.40E+05	1.10E+06	2.80E+05	2.80E+05	1.2	3.9	1.0
CPVL	1	1	3	3	3	3	1.20E+05	5.70E+05	6.40E+05	1.20E+05	1.0	4.8	5.3
EIF5B	2	2	3	3	2	2	3.80E+05	9.90E+05	2.60E+05	2.60E+05	1.5	3.8	1.0

ATP5PD	2	2	3	3	2	2	9.00E+05	1.50E+06	8.30E+05	8.30E+05	1.1	1.8	1.0
NIPSNAP1	2	2	2	2	3	3	1.50E+06	1.60E+06	1.50E+06	1.50E+06	1.0	1.1	1.0
ITPR3	2	2	2	3	2	2	7.80E+05	3.50E+05	1.50E+05	1.50E+05	5.2	2.3	1.0
NDUFAF7	3	3	2	2	2	2	7.90E+05	1.30E+06	4.20E+05	4.20E+05	1.9	3.1	1.0
CHD4	0	0	4	5	2	2	NF	1.60E+06	2.20E+05	2.20E+05	0.0	7.3	1.0
STEAP3	2	2	2	2	3	3	1.80E+06	1.50E+06	1.10E+06	1.10E+06	1.6	1.4	1.0
AIMP2	4	5	2	2	0	0	6.90E+05	2.50E+05	NF	2.50E+05	2.8	1.0	0.0
RPL23A	3	4	3	3	0	0	9.80E+06	3.90E+06	NF	3.90E+06	2.5	1.0	0.0
RAN	4	4	3	3	0	0	3.80E+06	3.30E+06	NF	3.30E+06	1.2	1.0	0.0
TXNDC5	3	3	2	2	2	2	2.20E+06	3.60E+05	1.40E+06	3.60E+05	6.1	1.0	3.9
LBR	2	2	2	2	2	2	1.10E+06	1.80E+06	3.00E+05	3.00E+05	3.7	6.0	1.0
TGFBI	2	2	3	3	1	1	4.40E+05	9.90E+05	1.50E+05	1.50E+05	2.9	6.6	1.0
PYCR1	2	3	1	1	1	2	6.30E+05	1.00E+05	9.10E+05	1.00E+05	6.3	1.0	9.1
HCCS	3	3	2	2	1	1	2.10E+06	8.80E+05	6.20E+05	6.20E+05	3.4	1.4	1.0
PGK1	1	1	3	3	2	2	2.30E+05	3.60E+05	3.90E+05	2.30E+05	1.0	1.6	1.7
MRPS28	1	1	2	2	3	3	2.60E+05	7.60E+05	1.00E+06	2.60E+05	1.0	2.9	3.8
RSL1D1	3	3	2	2	1	1	6.20E+06	4.20E+05	1.10E+05	1.10E+05	56.4	3.8	1.0
GNA13	3	3	2	2	1	1	6.10E+05	5.70E+05	2.50E+05	2.50E+05	2.4	2.3	1.0
ARHGAP21	1	1	3	4	1	1	7.70E+05	5.40E+05	8.60E+04	8.60E+04	9.0	6.3	1.0
TXNRD2	3	4	0	0	2	2	2.50E+06	NF	2.40E+05	2.40E+05	10.4	0.0	1.0
BAZ1B	0	0	4	4	2	2	NF	1.60E+06	6.10E+05	6.10E+05	0.0	2.6	1.0
MRPL46	2	2	2	2	2	2	1.10E+06	1.60E+06	9.10E+05	9.10E+05	1.2	1.8	1.0
CORO1B	4	5	1	1	0	0	1.00E+06	8.70E+04	NF	8.70E+04	11.5	1.0	0.0
MRPL3	3	3	1	1	2	2	2.00E+06	6.20E+05	1.40E+06	6.20E+05	3.2	1.0	2.3
GCDH	4	4	2	2	0	0	2.40E+06	8.40E+05	NF	8.40E+05	2.9	1.0	0.0
MRPS2	1	1	2	2	3	3	6.30E+05	2.20E+06	2.00E+06	6.30E+05	1.0	3.5	3.2
RPS11	2	2	4	4	0	0	4.40E+07	3.90E+07	NF	3.90E+07	1.1	1.0	0.0
PDHX	0	0	4	4	2	2	NF	2.00E+06	9.50E+05	9.50E+05	0.0	2.1	1.0
EPHB2	0	0	4	4	1	1	NF	4.50E+05	7.30E+04	7.30E+04	0.0	6.2	1.0
FKBP10	3	3	2	2	0	0	1.70E+06	7.50E+05	NF	7.50E+05	2.3	1.0	0.0
UBXN4	3	3	2	2	0	0	3.50E+06	6.30E+06	NF	3.50E+06	1.0	1.8	0.0
SLC27A2	2	2	0	0	3	3	2.30E+05	NF	8.80E+05	2.30E+05	1.0	0.0	3.8
HIBCH	1	1	0	0	4	4	2.10E+05	NF	1.10E+06	2.10E+05	1.0	0.0	5.2
MRPL45	4	4	0	0	0	0	1.60E+06	NF	NF	1.60E+06	1.0	0.0	0.0
CTPS1	1	1	3	3	0	0	1.10E+04	1.40E+06	NF	1.10E+04	1.0	####	0.0
SLC25A6	3	5	2	5	2	4	4.70E+07	2.90E+07	2.80E+07	2.80E+07	1.7	1.0	1.0
ILVBL	3	4	3	5	2	3	2.50E+05	1.70E+05	6.50E+04	6.50E+04	3.8	2.6	1.0
MPST	3	5	3	3	3	3	1.60E+06	4.40E+05	2.50E+05	2.50E+05	6.4	1.8	1.0
ATP5MF	3	5	2	3	2	3	1.30E+07	7.10E+06	2.50E+06	2.50E+06	5.2	2.8	1.0
GSR	3	3	3	4	3	4	1.90E+06	1.30E+06	1.10E+06	1.10E+06	1.7	1.2	1.0
ITCH	3	5	2	3	2	3	1.20E+06	3.60E+05	2.10E+05	2.10E+05	5.7	1.7	1.0
RDX	3	5	3	3	3	3	5.30E+05	6.40E+05	3.80E+05	3.80E+05	1.4	1.7	1.0
ATP5MG	3	3	3	4	3	4	1.30E+07	9.30E+06	7.10E+06	7.10E+06	1.8	1.3	1.0
CFL1	3	3	2	3	3	4	3.10E+06	5.80E+06	1.90E+06	1.90E+06	1.6	3.1	1.0
RBMX	2	3	3	4	2	3	3.70E+06	4.70E+06	2.00E+06	2.00E+06	1.9	2.4	1.0
GFM2	3	3	3	3	3	4	6.60E+05	5.50E+05	3.80E+05	3.80E+05	1.7	1.4	1.0
C1QBP	2	3	3	3	3	4	3.90E+06	4.50E+06	3.30E+06	3.30E+06	1.2	1.4	1.0
RPL31	3	4	2	3	2	3	6.80E+06	5.40E+06	2.70E+06	2.70E+06	2.5	2.0	1.0
RAB3D	1	3	1	2	3	5	1.70E+07	1.90E+07	2.20E+07	1.70E+07	1.0	1.1	1.3
RPS23	2	3	3	3	3	4	6.70E+06	6.20E+06	6.30E+06	6.20E+06	1.1	1.0	1.0
SRSF3	3	4	3	3	3	3	4.40E+06	1.80E+06	9.10E+05	9.10E+05	4.8	2.0	1.0

FAHD1	3	5	2	2	2	2	7.50E+05	3.00E+05	1.20E+05	1.20E+05	6.3	2.5	1.0
MRPL49	3	3	3	3	3	3	2.50E+06	1.90E+06	1.50E+06	1.50E+06	1.7	1.3	1.0
GEMIN5	3	3	3	3	3	3	1.20E+06	1.20E+06	7.20E+05	7.20E+05	1.7	1.7	1.0
PGRMC2	3	3	3	3	3	3	3.40E+06	2.70E+06	1.50E+06	1.50E+06	2.3	1.8	1.0
LACTB	3	3	3	3	3	3	1.80E+06	1.00E+06	9.00E+05	9.00E+05	2.0	1.1	1.0
FAR1	3	4	2	3	2	2	1.50E+06	1.50E+06	8.00E+05	8.00E+05	1.9	1.9	1.0
RDH13	3	4	1	2	2	3	2.00E+06	3.20E+05	7.50E+05	3.20E+05	6.3	1.0	2.3
RMDN3	2	2	2	3	3	4	1.60E+06	2.10E+06	1.60E+06	1.60E+06	1.0	1.3	1.0
CXADR	3	3	3	3	3	3	1.10E+06	5.50E+05	4.00E+05	4.00E+05	2.8	1.4	1.0
MICOS13	3	3	3	3	3	3	1.80E+06	8.40E+05	7.50E+05	7.50E+05	2.4	1.1	1.0
TMX1	3	3	3	3	3	3	4.10E+06	2.80E+06	2.70E+06	2.70E+06	1.5	1.0	1.0
SLC25A19	3	3	3	3	3	3	9.40E+06	1.30E+07	8.30E+06	8.30E+06	1.1	1.6	1.0
HNRNPD	3	3	2	3	2	2	2.80E+06	5.00E+06	1.70E+06	1.70E+06	1.6	2.9	1.0
MICU2	3	3	2	2	3	3	1.10E+06	6.70E+05	6.20E+05	6.20E+05	1.8	1.1	1.0
PPT1	3	3	2	2	3	3	4.10E+06	5.90E+06	3.90E+06	3.90E+06	1.1	1.5	1.0
CCT8	3	3	3	3	2	2	1.50E+06	1.90E+06	3.40E+05	3.40E+05	4.4	5.6	1.0
RAB10	3	3	2	3	1	2	1.70E+06	1.20E+06	1.80E+05	1.80E+05	9.4	6.7	1.0
NUDT19	2	3	2	2	2	3	7.30E+05	4.20E+05	1.90E+05	1.90E+05	3.8	2.2	1.0
WDR1	2	2	3	3	3	3	1.50E+06	2.00E+06	1.30E+06	1.30E+06	1.2	1.5	1.0
GSTK1	2	2	3	3	3	3	1.20E+06	2.30E+06	1.70E+06	1.20E+06	1.0	1.9	1.4
AUP1	3	4	1	2	2	2	7.60E+05	3.80E+05	3.10E+05	3.10E+05	2.5	1.2	1.0
SLIRP	3	3	3	3	2	2	1.00E+07	5.50E+06	2.60E+06	2.60E+06	3.8	2.1	1.0
RPL12	3	3	3	3	2	2	7.90E+06	1.00E+07	4.20E+06	4.20E+06	1.9	2.4	1.0
SUMF2	2	2	3	4	2	2	3.90E+05	8.30E+05	3.90E+05	3.90E+05	1.0	2.1	1.0
HACD3	3	3	2	3	2	2	2.70E+06	3.10E+06	5.20E+05	5.20E+05	5.2	6.0	1.0
MTX1	2	2	3	3	3	3	1.10E+06	1.30E+06	7.20E+05	7.20E+05	1.5	1.8	1.0
TRNT1	3	3	3	3	2	2	8.00E+05	3.40E+05	2.70E+05	2.70E+05	3.0	1.3	1.0
PUS1	3	3	3	3	2	2	1.60E+06	1.30E+06	6.00E+05	6.00E+05	2.7	2.2	1.0
CAV1	0	0	3	4	2	4	NF	6.20E+06	3.00E+06	3.00E+06	0.0	2.1	1.0
ATP6V0D1	2	2	3	3	3	3	2.00E+06	2.20E+06	2.00E+06	2.00E+06	1.0	1.1	1.0
TMCO1	3	3	3	3	2	2	3.10E+06	1.30E+06	1.10E+06	1.10E+06	2.8	1.2	1.0
PPIF	0	0	3	4	3	4	NF	5.30E+06	4.40E+06	4.40E+06	0.0	1.2	1.0
EARS2	2	2	3	3	3	3	1.10E+06	2.10E+06	2.50E+06	1.10E+06	1.0	1.9	2.3
MTARC1	3	3	1	1	2	3	6.90E+05	5.10E+04	6.70E+04	5.10E+04	13.5	1.0	1.3
GPC1	2	2	3	3	2	2	1.50E+06	2.60E+06	1.00E+06	1.00E+06	1.5	2.6	1.0
OXSM	2	2	3	3	2	2	6.10E+05	6.60E+05	5.00E+05	5.00E+05	1.2	1.3	1.0
IMPDH2	2	2	3	3	2	2	6.60E+05	1.40E+06	7.90E+05	6.60E+05	1.0	2.1	1.2
MCM7	3	3	2	2	2	2	4.70E+05	2.30E+05	1.30E+05	1.30E+05	3.6	1.8	1.0
MTOR	2	2	2	2	3	3	2.10E+05	1.70E+05	1.40E+05	1.40E+05	1.5	1.2	1.0
Igh-6	1	1	2	2	3	4	5.40E+05	5.00E+05	4.10E+05	4.10E+05	1.3	1.2	1.0
KIF11	1	1	3	4	2	2	4.40E+05	9.10E+05	3.60E+05	3.60E+05	1.2	2.5	1.0
MACROD1	2	2	2	2	3	3	3.80E+06	2.50E+06	2.50E+06	2.50E+06	1.5	1.0	1.0
PFN1	2	2	3	3	2	2	1.80E+06	2.20E+06	1.10E+06	1.10E+06	1.6	2.0	1.0
RPL22	2	2	3	3	2	2	1.00E+07	1.10E+07	6.70E+06	6.70E+06	1.5	1.6	1.0
RPUSD3	2	2	2	2	3	3	4.40E+05	2.90E+05	4.30E+05	2.90E+05	1.5	1.0	1.5
RPL17	3	3	2	2	2	2	3.70E+06	2.90E+06	1.40E+06	1.40E+06	2.6	2.1	1.0
ACOT13	3	3	2	2	2	2	5.10E+06	3.70E+06	2.30E+06	2.30E+06	2.2	1.6	1.0
RAB18	2	2	2	2	3	3	1.50E+06	1.50E+06	1.40E+06	1.40E+06	1.1	1.1	1.0
TARS1	2	2	3	3	2	2	5.50E+05	1.20E+06	4.40E+05	4.40E+05	1.3	2.7	1.0
CRYZ	2	2	2	2	3	3	1.70E+06	1.20E+06	7.70E+06	1.20E+06	1.4	1.0	6.4
HNRNPH3	3	3	1	1	1	3	1.70E+06	1.00E+05	8.30E+04	8.30E+04	20.5	1.2	1.0

GLA	2	2	2	2	3	3	8.50E+05	4.90E+05	9.80E+05	4.90E+05	1.7	1.0	2.0
GALNT7	2	2	2	2	3	3	1.70E+06	1.60E+06	1.20E+06	1.20E+06	1.4	1.3	1.0
ERP44	3	3	2	2	2	2	2.30E+06	1.00E+06	1.10E+06	1.00E+06	2.3	1.0	1.1
CIT	0	0	3	4	3	3	NF	9.90E+05	8.70E+05	8.70E+05	0.0	1.1	1.0
MRPS18B	2	3	2	2	2	2	3.90E+06	1.60E+06	9.60E+05	9.60E+05	4.1	1.7	1.0
HNRNPCL1	2	2	3	3	2	2	5.60E+06	5.70E+06	2.80E+06	2.80E+06	2.0	2.0	1.0
MAP7	3	3	2	2	2	2	2.20E+06	1.40E+06	1.60E+06	1.40E+06	1.6	1.0	1.1
USP10	3	4	0	0	3	3	1.10E+06	NF	3.40E+05	3.40E+05	3.2	0.0	1.0
GGCX	2	2	2	2	3	3	6.30E+05	5.70E+05	4.70E+05	4.70E+05	1.3	1.2	1.0
ALDH6A1	2	2	2	2	3	3	1.10E+06	5.50E+05	9.00E+05	5.50E+05	2.0	1.0	1.6
EIF3F	3	4	1	1	1	2	1.00E+06	4.50E+05	1.20E+06	4.50E+05	2.2	1.0	2.7
SF3B1	3	3	2	2	2	2	1.40E+06	1.00E+06	6.20E+05	6.20E+05	2.3	1.6	1.0
BCAT2	3	5	0	0	2	2	2.60E+06	NF	9.60E+05	9.60E+05	2.7	0.0	1.0
ANKRD17	0	0	3	4	1	2	NF	6.80E+05	6.00E+04	6.00E+04	0.0	11.3	1.0
CCT5	2	2	2	2	1	2	4.10E+05	4.90E+05	1.60E+05	1.60E+05	2.6	3.1	1.0
PLCD3	3	3	3	3	0	0	3.40E+05	2.40E+05	NF	2.40E+05	1.4	1.0	0.0
DNAJA2	1	1	3	3	2	2	6.70E+05	5.00E+05	2.20E+05	2.20E+05	3.0	2.3	1.0
SLC7A1	2	2	1	1	2	3	5.80E+05	2.20E+05	4.00E+05	2.20E+05	2.6	1.0	1.8
RAI14	3	3	1	1	2	2	1.70E+06	1.40E+05	4.10E+05	1.40E+05	12.1	1.0	2.9
NT5C3A	1	1	2	2	3	3	3.80E+05	7.00E+05	1.40E+06	3.80E+05	1.0	1.8	3.7
MTX2	3	3	2	2	1	1	3.20E+06	4.00E+05	2.20E+05	2.20E+05	14.5	1.8	1.0
RPL15	3	3	2	2	1	1	4.30E+06	3.30E+06	1.10E+06	1.10E+06	3.9	3.0	1.0
COBL	2	2	2	3	1	1	5.20E+05	3.20E+05	1.20E+05	1.20E+05	4.3	2.7	1.0
MFN1	3	3	1	1	1	2	1.30E+06	6.00E+04	9.90E+04	6.00E+04	21.7	1.0	1.7
PHGDH	3	3	3	3	0	0	2.40E+06	2.70E+06	NF	2.40E+06	1.0	1.1	0.0
BCL2L13	3	3	2	2	1	1	1.20E+06	9.10E+05	5.30E+05	5.30E+05	2.3	1.7	1.0
FKBP2	0	0	3	3	2	3	NF	1.30E+06	6.70E+05	6.70E+05	0.0	1.9	1.0
HSPB1	3	3	2	2	1	1	1.00E+06	7.20E+05	1.10E+05	1.10E+05	9.1	6.5	1.0
PRKACA	2	2	2	2	2	2	3.40E+05	6.00E+05	3.90E+05	3.40E+05	1.0	1.8	1.1
VAPB	3	3	2	2	1	1	1.50E+07	2.90E+06	1.30E+06	1.30E+06	11.5	2.2	1.0
CAPRIN1	2	2	1	1	3	3	1.50E+06	8.80E+05	1.30E+06	8.80E+05	1.7	1.0	1.5
ERLIN1	2	2	1	1	3	3	1.60E+06	1.30E+06	1.90E+06	1.30E+06	1.2	1.0	1.5
COQ8B	1	1	2	2	3	3	5.10E+05	2.60E+05	2.50E+05	2.50E+05	2.0	1.0	1.0
MTERF3	2	2	1	1	3	3	1.60E+06	2.60E+05	7.10E+05	2.60E+05	6.2	1.0	2.7
MTO1	3	3	1	1	2	2	1.30E+06	5.50E+04	3.30E+05	5.50E+04	23.6	1.0	6.0
LAMTOR2	2	2	1	1	3	3	5.50E+05	2.30E+05	4.70E+05	2.30E+05	2.4	1.0	2.0
EMC3	0	0	3	3	3	3	NF	7.50E+05	6.30E+05	6.30E+05	0.0	1.2	1.0
NCAPD2	1	1	3	3	1	2	9.50E+04	2.70E+05	4.10E+04	4.10E+04	2.3	6.6	1.0
TOP2B	2	2	2	2	2	2	5.20E+05	3.90E+05	1.80E+05	1.80E+05	2.9	2.2	1.0
MRPL17	3	3	1	1	2	2	1.80E+06	1.00E+06	1.20E+06	1.00E+06	1.8	1.0	1.2
NDC1	1	1	2	2	2	3	3.60E+04	5.10E+05	7.40E+05	3.60E+04	1.0	14.2	20.6
SCPEP1	2	2	2	2	2	2	1.10E+06	6.00E+05	8.30E+05	6.00E+05	1.8	1.0	1.4
MRPL12	0	0	3	3	2	3	NF	4.90E+05	3.40E+05	3.40E+05	0.0	1.4	1.0
BCAR1	0	0	3	3	3	3	NF	5.30E+05	6.80E+05	5.30E+05	0.0	1.0	1.3
HNRNPAB	3	3	1	1	1	2	4.30E+05	2.20E+05	3.40E+05	2.20E+05	2.0	1.0	1.5
SLC25A18	2	2	2	2	2	2	2.00E+06	1.20E+06	9.80E+05	9.80E+05	2.0	1.2	1.0
CYB5R1	2	2	2	2	2	2	1.50E+06	1.20E+06	6.70E+05	6.70E+05	2.2	1.8	1.0
FAM98A	2	2	1	1	2	2	1.80E+06	3.30E+05	3.70E+05	3.30E+05	5.5	1.0	1.1
PGRMC1	1	1	1	1	2	3	4.80E+05	3.20E+05	8.10E+05	3.20E+05	1.5	1.0	2.5
HNRNPUL1	1	1	2	2	2	2	8.20E+05	1.20E+06	7.70E+05	7.70E+05	1.1	1.6	1.0
MUC13	2	2	1	1	2	2	1.60E+06	5.40E+05	8.10E+05	5.40E+05	3.0	1.0	1.5

TUBB4B	0	0	3	4	1	1	NF	3.30E+06	5.90E+05	5.90E+05	0.0	5.6	1.0
APLP2	2	2	1	1	2	2	8.50E+05	5.00E+05	4.20E+05	4.20E+05	2.0	1.2	1.0
AK3	2	2	1	1	2	2	4.90E+05	4.80E+05	6.90E+05	4.80E+05	1.0	1.0	1.4
RAP2A	2	2	1	2	1	1	6.10E+05	2.00E+04	3.10E+04	2.00E+04	30.5	1.0	1.6
CDC5L	0	0	3	3	2	2	NF	3.70E+05	1.30E+05	1.30E+05	0.0	2.8	1.0
YES1	2	2	2	3	0	0	3.10E+05	9.90E+05	NF	3.10E+05	1.0	3.2	0.0
MRPS25	2	2	1	1	2	2	9.60E+05	7.70E+05	1.10E+06	7.70E+05	1.2	1.0	1.4
SLC29A1	0	0	2	2	3	3	NF	1.30E+06	8.80E+05	8.80E+05	0.0	1.5	1.0
XPO5	1	1	3	3	1	1	1.90E+05	4.30E+05	8.80E+04	8.80E+04	2.2	4.9	1.0
CDK5RAP1	2	2	2	2	1	1	1.10E+06	6.10E+05	2.40E+05	2.40E+05	4.6	2.5	1.0
BAIAP2	0	0	3	3	2	2	NF	3.80E+05	6.40E+04	6.40E+04	0.0	5.9	1.0
EDC4	2	2	2	2	1	1	2.30E+05	2.40E+05	8.50E+04	8.50E+04	2.7	2.8	1.0
MARS2	2	2	1	1	2	2	4.40E+05	9.80E+04	1.70E+05	9.80E+04	4.5	1.0	1.7
NDFIP1	1	1	2	2	2	2	1.70E+05	4.00E+05	3.30E+05	1.70E+05	1.0	2.4	1.9
PKP3	1	1	2	2	2	2	1.40E+05	1.90E+05	2.40E+05	1.40E+05	1.0	1.4	1.7
IMMT	0	0	2	3	2	2	NF	2.10E+06	9.90E+05	9.90E+05	0.0	2.1	1.0
SLC39A14	0	0	3	3	2	2	NF	3.80E+06	2.30E+06	2.30E+06	0.0	1.7	1.0
PDS5A	1	1	3	3	1	1	1.30E+05	3.00E+05	5.60E+04	5.60E+04	2.3	5.4	1.0
CHST14	2	2	2	2	1	1	8.30E+05	1.00E+06	2.70E+05	2.70E+05	3.1	3.7	1.0
SMC4	1	1	3	3	1	1	4.50E+05	1.60E+06	2.70E+05	2.70E+05	1.7	5.9	1.0
NAT10	3	3	2	2	0	0	3.50E+05	6.00E+04	NF	6.00E+04	5.8	1.0	0.0
CADM1	1	1	2	2	2	2	3.80E+05	7.70E+05	4.70E+05	3.80E+05	1.0	2.0	1.2
GAPVD1	0	0	3	3	2	2	NF	3.50E+05	2.20E+05	2.20E+05	0.0	1.6	1.0
GNA11	2	2	2	2	1	1	9.00E+05	1.10E+06	3.20E+05	3.20E+05	2.8	3.4	1.0
CAPZB	1	1	2	2	2	2	2.60E+06	3.10E+06	6.40E+05	6.40E+05	4.1	4.8	1.0
GGH	2	2	1	1	2	2	6.40E+05	1.20E+05	7.00E+05	1.20E+05	5.3	1.0	5.8
ARL8A	2	2	1	1	2	2	1.20E+06	9.60E+05	1.60E+06	9.60E+05	1.3	1.0	1.7
ADGRE5	1	1	3	3	1	1	7.30E+05	1.30E+06	4.20E+05	4.20E+05	1.7	3.1	1.0
TOMM20	3	3	1	1	1	1	6.20E+06	1.70E+04	3.60E+06	1.70E+04	####	1.0	####
VPS13A	2	2	2	2	1	1	1.00E+06	4.60E+05	1.90E+05	1.90E+05	5.3	2.4	1.0
ALDH1L1	3	3	1	1	1	1	1.30E+06	3.40E+05	2.90E+05	2.90E+05	4.5	1.2	1.0
SEMA3C	0	0	3	3	2	2	NF	2.10E+06	2.50E+05	2.50E+05	0.0	8.4	1.0
ATP1B1	3	3	2	2	0	0	1.10E+07	7.70E+06	NF	7.70E+06	1.4	1.0	0.0
SPECC1	2	2	1	1	2	2	3.40E+05	2.40E+05	2.10E+05	2.10E+05	1.6	1.1	1.0
ATP5ME	2	2	2	2	1	1	7.60E+06	3.50E+06	2.90E+06	2.90E+06	2.6	1.2	1.0
MPC2	2	2	2	2	1	1	6.80E+05	5.30E+05	3.50E+05	3.50E+05	1.9	1.5	1.0
RPL5	2	2	3	3	0	0	2.60E+06	6.70E+06	NF	2.60E+06	1.0	2.6	0.0
SLC35B2	1	1	2	2	2	2	1.10E+06	1.80E+06	6.10E+05	6.10E+05	1.8	3.0	1.0
KIF5C	0	0	0	0	3	5	NF	NF	4.60E+06	4.60E+06	0.0	0.0	1.0
RPL35A	2	2	1	1	2	2	1.80E+06	1.10E+06	1.10E+06	1.10E+06	1.6	1.0	1.0
SEL1L	1	1	2	2	1	1	1.30E+05	1.90E+05	4.10E+04	4.10E+04	3.2	4.6	1.0
SUN2	3	4	0	0	0	0	2.60E+05	NF	NF	2.60E+05	1.0	0.0	0.0
STX4	2	2	2	2	0	0	6.90E+05	4.50E+05	NF	4.50E+05	1.5	1.0	0.0
RAB34	2	2	0	0	2	2	1.40E+06	NF	5.50E+05	5.50E+05	2.5	0.0	1.0
SEPTIN2	2	2	1	1	1	1	2.90E+05	6.80E+04	5.30E+04	5.30E+04	5.5	1.3	1.0
DBT	0	0	3	3	1	1	NF	6.20E+05	8.20E+03	8.20E+03	0.0	75.6	1.0
ACLY	0	0	2	2	2	2	NF	1.80E+05	1.40E+05	1.40E+05	0.0	1.3	1.0
DNM1L	1	1	1	1	2	2	1.40E+04	1.70E+04	1.40E+05	1.40E+04	1.0	1.2	10.0
RPLP2	0	0	1	1	3	3	NF	3.50E+05	3.70E+05	3.50E+05	0.0	1.0	1.1
EPHA7	0	0	1	1	3	3	NF	2.80E+05	5.70E+05	2.80E+05	0.0	1.0	2.0
LPCAT1	2	2	2	2	0	0	1.20E+06	8.20E+05	NF	8.20E+05	1.5	1.0	0.0

DHX15	0	0	2	2	2	2	NF	2.50E+05	1.70E+05	1.70E+05	0.0	1.5	1.0
PKP4	2	2	2	2	0	0	1.20E+05	2.60E+05	NF	1.20E+05	1.0	2.2	0.0
MRPL55	2	2	2	2	0	0	8.80E+05	3.80E+05	NF	3.80E+05	2.3	1.0	0.0
ATAD1	2	2	0	0	1	2	6.40E+05	NF	1.20E+06	6.40E+05	1.0	0.0	1.9
MCM3	1	1	3	3	0	0	6.80E+04	3.30E+05	NF	6.80E+04	1.0	4.9	0.0
NELFB	0	0	3	3	1	1	NF	4.20E+05	4.50E+04	4.50E+04	0.0	9.3	1.0
TAP1	2	2	1	1	1	1	5.70E+05	9.20E+05	4.90E+04	4.90E+04	11.6	18.8	1.0
RPS21	1	1	2	2	1	1	2.80E+05	7.70E+05	2.50E+05	2.50E+05	1.1	3.1	1.0
RPL38	3	4	0	0	0	0	7.40E+06	NF	NF	7.40E+06	1.0	0.0	0.0
MRPL13	3	3	1	1	0	0	1.30E+06	1.60E+05	NF	1.60E+05	8.1	1.0	0.0
NUP188	0	0	2	2	2	2	NF	5.90E+05	3.10E+05	3.10E+05	0.0	1.9	1.0
EHD2	2	2	2	2	0	0	5.10E+05	9.20E+05	NF	5.10E+05	1.0	1.8	0.0
ACSL1	0	0	1	1	3	3	NF	3.60E+05	3.70E+05	3.60E+05	0.0	1.0	1.0
BET1	3	3	0	0	0	0	7.40E+05	NF	NF	7.40E+05	1.0	0.0	0.0
HUWE1	3	3	0	0	0	0	6.00E+04	NF	NF	6.00E+04	1.0	0.0	0.0
MFSD12	3	3	0	0	0	0	2.40E+06	NF	NF	2.40E+06	1.0	0.0	0.0
SLC4A7	0	0	3	3	0	0	NF	2.90E+05	NF	2.90E+05	0.0	1.0	0.0
RSU1	3	3	0	0	0	0	2.10E+05	NF	NF	2.10E+05	1.0	0.0	0.0
SMC1A	0	0	3	3	0	0	NF	2.20E+06	NF	2.20E+06	0.0	1.0	0.0
STAT3	0	0	3	3	0	0	NF	6.60E+05	NF	6.60E+05	0.0	1.0	0.0
VARS1	2	2	0	0	1	1	2.50E+05	NF	2.30E+05	2.30E+05	1.1	0.0	1.0
RPL30	2	2	1	1	0	0	1.30E+06	3.60E+05	NF	3.60E+05	3.6	1.0	0.0
NUP107	2	2	1	1	0	0	4.20E+05	3.30E+05	NF	3.30E+05	1.3	1.0	0.0
ITSN1	0	0	3	3	0	0	NF	1.80E+06	NF	1.80E+06	0.0	1.0	0.0
CCPG1	0	0	1	1	2	2	NF	4.40E+05	5.30E+05	4.40E+05	0.0	1.0	1.2
SHMT1	2	5	2	7	2	5	7.40E+06	3.00E+06	1.60E+06	1.60E+06	4.6	1.9	1.0
H3C12	2	5	2	5	2	6	8.60E+07	8.00E+07	4.90E+07	4.90E+07	1.8	1.6	1.0
RPS26	2	4	2	4	2	3	9.60E+06	9.20E+06	5.10E+06	5.10E+06	1.9	1.8	1.0
VAPA	2	3	2	2	2	5	5.20E+06	1.60E+06	3.30E+06	1.60E+06	3.3	1.0	2.1
IFITM1	1	2	2	4	2	3	4.80E+06	5.60E+06	3.40E+06	3.40E+06	1.4	1.6	1.0
RPL11	2	3	2	3	2	3	1.10E+07	1.10E+07	7.30E+06	7.30E+06	1.5	1.5	1.0
MRPL21	2	2	2	4	2	3	2.00E+06	1.30E+06	1.30E+06	1.30E+06	1.5	1.0	1.0
RPL8	2	3	2	3	2	3	5.00E+06	3.40E+06	2.10E+06	2.10E+06	2.4	1.6	1.0
RPL19	1	3	2	3	2	3	5.30E+06	4.50E+06	3.00E+06	3.00E+06	1.8	1.5	1.0
SERBP1	2	2	2	3	2	3	1.30E+06	1.70E+06	9.80E+05	9.80E+05	1.3	1.7	1.0
ARL6IP5	2	2	2	4	2	2	1.10E+06	7.10E+05	6.40E+05	6.40E+05	1.7	1.1	1.0
SNRPD2	2	2	2	4	2	2	3.40E+05	6.30E+05	1.20E+05	1.20E+05	2.8	5.3	1.0
COX7A2	2	3	2	3	2	2	3.40E+06	3.20E+06	2.10E+06	2.10E+06	1.6	1.5	1.0
RPS27	1	2	2	3	2	3	5.70E+06	6.10E+06	4.70E+06	4.70E+06	1.2	1.3	1.0
MRPL20	2	2	2	3	2	3	1.50E+06	3.10E+06	2.10E+06	1.50E+06	1.0	2.1	1.4
SLC44A1	2	2	2	4	2	2	9.10E+05	1.40E+06	3.70E+05	3.70E+05	2.5	3.8	1.0
YWHAQ	2	3	2	3	2	2	2.50E+06	3.20E+06	1.60E+06	1.60E+06	1.6	2.0	1.0
PCBP1	2	3	2	2	2	3	2.20E+06	1.70E+06	1.20E+06	1.20E+06	1.8	1.4	1.0
GCAT	2	3	2	2	2	2	8.40E+05	2.50E+05	2.10E+05	2.10E+05	4.0	1.2	1.0
COX7A2L	2	2	2	2	2	3	7.40E+05	4.80E+05	5.60E+05	4.80E+05	1.5	1.0	1.2
SPR	2	2	2	2	2	3	5.10E+05	2.00E+05	1.70E+05	1.70E+05	3.0	1.2	1.0
GLS	2	2	2	2	2	3	1.10E+06	9.80E+05	9.40E+05	9.40E+05	1.2	1.0	1.0
TRABD	2	2	2	2	2	3	1.60E+06	9.40E+05	7.30E+05	7.30E+05	2.2	1.3	1.0
EIF2S3	1	1	2	3	2	3	3.40E+05	8.20E+05	4.90E+05	3.40E+05	1.0	2.4	1.4
IBA57	2	2	2	3	2	2	6.30E+05	7.40E+05	3.80E+05	3.80E+05	1.7	1.9	1.0
XPNPEP3	2	2	2	2	2	3	8.10E+05	6.30E+05	6.50E+05	6.30E+05	1.3	1.0	1.0

ARL1	2	2	2	2	2	3	5.70E+05	3.20E+05	2.50E+05	2.50E+05	2.3	1.3	1.0
MRPL43	2	2	2	2	2	3	3.30E+06	2.70E+06	3.00E+06	2.70E+06	1.2	1.0	1.1
MRPL40	2	3	1	2	2	2	1.70E+06	1.50E+05	2.30E+05	1.50E+05	11.3	1.0	1.5
SRP68	2	2	1	2	1	3	6.90E+05	5.50E+05	6.90E+05	5.50E+05	1.3	1.0	1.3
SCO2	1	2	2	2	2	3	7.50E+05	2.70E+05	2.70E+05	2.70E+05	2.8	1.0	1.0
FLAD1	2	3	2	2	2	2	5.90E+04	5.20E+04	2.50E+04	2.50E+04	2.4	2.1	1.0
NDUFA12	2	2	2	2	2	3	6.20E+05	7.60E+05	9.10E+05	6.20E+05	1.0	1.2	1.5
RPS28	2	2	2	3	2	2	3.30E+06	4.10E+06	1.50E+06	1.50E+06	2.2	2.7	1.0
IDE	2	2	2	2	2	3	5.10E+05	1.70E+05	2.30E+05	1.70E+05	3.0	1.0	1.4
PTPMT1	2	2	1	2	2	3	1.40E+06	6.80E+04	7.00E+05	6.80E+04	20.6	1.0	10.3
PARL	2	3	1	1	2	3	1.10E+06	8.40E+04	6.70E+05	8.40E+04	13.1	1.0	8.0
VAMP2	2	2	2	2	2	2	4.10E+06	2.60E+06	1.90E+06	1.90E+06	2.2	1.4	1.0
MRPL2	2	2	1	2	2	2	1.40E+06	6.30E+05	7.30E+05	6.30E+05	2.2	1.0	1.2
LGMN	1	1	2	2	2	3	8.10E+05	5.00E+05	3.20E+05	3.20E+05	2.5	1.6	1.0
MGST3	2	2	2	2	2	2	1.60E+06	5.00E+05	3.80E+05	3.80E+05	4.2	1.3	1.0
B3GAT3	2	2	2	2	2	2	3.00E+05	1.90E+05	1.10E+05	1.10E+05	2.7	1.7	1.0
HIP1R	2	2	2	2	2	2	5.70E+05	3.70E+05	2.30E+05	2.30E+05	2.5	1.6	1.0
RER1	2	2	2	2	2	2	1.70E+06	1.30E+06	1.30E+06	1.30E+06	1.3	1.0	1.0
TWF1	2	2	2	2	2	2	8.20E+05	6.20E+05	3.30E+05	3.30E+05	2.5	1.9	1.0
NSDHL	2	2	2	3	1	1	1.20E+06	6.50E+05	8.20E+04	8.20E+04	14.6	7.9	1.0
CYB5B	2	3	1	1	2	2	3.10E+06	1.50E+06	1.40E+06	1.40E+06	2.2	1.1	1.0
PTDSS1	2	2	2	2	2	2	1.10E+06	4.80E+05	3.30E+05	3.30E+05	3.3	1.5	1.0
RALA	2	2	2	2	2	2	1.70E+06	1.60E+06	1.40E+06	1.40E+06	1.2	1.1	1.0
FAM114A2	1	1	2	2	2	3	2.10E+05	1.10E+05	1.20E+05	1.10E+05	1.9	1.0	1.1
RETSAT	1	1	2	3	2	2	9.90E+04	1.80E+05	8.20E+04	8.20E+04	1.2	2.2	1.0
NPEPPS	2	2	2	2	2	2	2.90E+05	2.30E+05	1.60E+05	1.60E+05	1.8	1.4	1.0
ARF3	2	2	2	2	2	2	7.40E+05	8.10E+05	4.50E+05	4.50E+05	1.6	1.8	1.0
STXBP3	2	2	2	2	2	2	1.00E+06	1.00E+06	5.40E+05	5.40E+05	1.9	1.9	1.0
GNAI1	2	2	2	2	2	2	8.80E+05	1.00E+06	9.20E+05	8.80E+05	1.0	1.1	1.0
SH3BP4	2	2	2	2	2	2	2.70E+05	2.10E+05	1.50E+05	1.50E+05	1.8	1.4	1.0
MRM1	2	3	1	1	2	2	3.20E+05	1.70E+05	2.00E+05	1.70E+05	1.9	1.0	1.2
CCAR2	2	2	2	2	2	2	3.70E+06	7.40E+06	1.20E+06	1.20E+06	3.1	6.2	1.0
MRPL23	2	2	2	2	2	2	1.50E+06	1.30E+06	9.90E+05	9.90E+05	1.5	1.3	1.0
DDB1	2	2	2	2	2	2	6.10E+05	5.50E+05	4.60E+05	4.60E+05	1.3	1.2	1.0
PRDX2	2	2	2	2	2	2	1.30E+05	1.00E+05	3.70E+04	3.70E+04	3.5	2.7	1.0
MANF	2	3	2	2	1	1	6.10E+05	3.60E+05	1.00E+05	1.00E+05	6.1	3.6	1.0
BAK1	2	2	2	2	2	2	8.40E+05	5.40E+05	5.50E+05	5.40E+05	1.6	1.0	1.0
MRPL47	2	2	2	2	2	2	6.30E+05	7.20E+05	6.40E+05	6.30E+05	1.0	1.1	1.0
CPA4	2	2	2	2	2	2	8.60E+05	6.20E+05	7.70E+05	6.20E+05	1.4	1.0	1.2
NDUFB11	2	2	2	2	2	2	3.20E+06	2.50E+06	2.20E+06	2.20E+06	1.5	1.1	1.0
H2AZ1	1	1	2	2	2	3	1.80E+06	3.30E+06	3.80E+06	1.80E+06	1.0	1.8	2.1
SLC38A1	2	2	2	2	2	2	4.70E+06	6.80E+06	4.90E+06	4.70E+06	1.0	1.4	1.0
TMED9	2	2	2	2	2	2	6.40E+06	4.50E+06	3.30E+06	3.30E+06	1.9	1.4	1.0
SEC11A	2	2	2	2	2	2	1.80E+06	1.20E+06	9.40E+05	9.40E+05	1.9	1.3	1.0
RAB6A	2	2	2	2	2	2	3.00E+06	2.20E+06	2.20E+06	2.20E+06	1.4	1.0	1.0
NDUFB3	2	2	2	2	2	2	2.00E+06	1.60E+06	1.50E+06	1.50E+06	1.3	1.1	1.0
SLC38A5	2	2	2	2	2	2	1.30E+06	9.60E+05	8.80E+05	8.80E+05	1.5	1.1	1.0
BCKDK	2	2	2	2	2	2	1.60E+06	9.00E+05	9.50E+05	9.00E+05	1.8	1.0	1.1
TIMM23	1	1	2	2	2	2	1.70E+06	1.50E+06	8.40E+05	8.40E+05	2.0	1.8	1.0
GIGYF2	2	2	2	2	1	1	1.10E+06	6.40E+05	9.50E+03	9.50E+03	####	67.4	1.0
NACA	2	2	2	2	1	1	1.80E+06	2.20E+06	4.60E+05	4.60E+05	3.9	4.8	1.0

TIMM17A	1	1	1	1	2	3	6.60E+05	1.40E+05	1.40E+05	1.40E+05	4.7	1.0	1.0
PCBP3	1	1	2	3	1	1	4.70E+05	1.10E+06	3.80E+05	3.80E+05	1.2	2.9	1.0
RAB1B	1	1	1	1	2	3	1.90E+05	9.00E+04	1.90E+05	9.00E+04	2.1	1.0	2.1
MRPL48	2	2	1	1	2	2	1.50E+06	8.10E+05	9.40E+05	8.10E+05	1.9	1.0	1.2
OXNAD1	1	1	2	2	2	2	2.10E+05	2.50E+05	2.50E+05	2.10E+05	1.0	1.2	1.2
NDUFA5	2	2	1	1	2	2	9.10E+05	3.00E+05	6.20E+05	3.00E+05	3.0	1.0	2.1
GPRC5A	1	1	2	2	2	2	7.80E+05	1.20E+06	7.30E+05	7.30E+05	1.1	1.6	1.0
ECHDC1	2	2	1	1	2	2	6.50E+05	1.90E+05	4.10E+05	1.90E+05	3.4	1.0	2.2
FASTKD5	2	2	2	2	1	1	5.20E+05	3.70E+05	1.40E+04	1.40E+04	37.1	26.4	1.0
HLA-A	2	2	2	3	0	0	2.90E+06	1.90E+06	NF	1.90E+06	1.5	1.0	0.0
SPART	1	1	2	2	2	2	2.80E+05	1.20E+06	1.10E+07	2.80E+05	1.0	4.3	39.3
SCAMP1	1	1	2	2	2	2	6.90E+05	7.80E+05	9.10E+05	6.90E+05	1.0	1.1	1.3
TMEM43	2	2	1	1	1	2	6.40E+05	1.10E+05	1.30E+05	1.10E+05	5.8	1.0	1.2
WDR11	1	1	2	2	2	2	9.60E+04	4.30E+05	2.20E+05	9.60E+04	1.0	4.5	2.3
UBR4	0	0	2	3	2	2	NF	4.10E+05	1.20E+05	1.20E+05	0.0	3.4	1.0
ADD1	1	1	2	2	2	2	1.60E+05	2.90E+05	1.90E+05	1.60E+05	1.0	1.8	1.2
LYRM4	1	1	2	2	2	2	9.10E+05	8.50E+05	7.80E+05	7.80E+05	1.2	1.1	1.0
MTHFD1	2	2	1	1	2	2	7.90E+05	4.30E+05	4.70E+05	4.30E+05	1.8	1.0	1.1
NARS2	2	2	1	1	2	2	7.40E+05	4.40E+05	6.30E+05	4.40E+05	1.7	1.0	1.4
DAD1	2	2	1	1	2	2	4.80E+06	1.40E+06	2.70E+06	1.40E+06	3.4	1.0	1.9
SLC49A4	1	1	2	2	2	2	4.10E+05	4.40E+05	5.20E+05	4.10E+05	1.0	1.1	1.3
NOP9	0	0	2	3	2	2	NF	9.30E+04	5.00E+04	5.00E+04	0.0	1.9	1.0
TMEM59	1	1	2	2	2	2	6.90E+05	1.10E+06	8.60E+05	6.90E+05	1.0	1.6	1.2
SNX27	2	2	1	1	2	2	9.20E+05	1.10E+05	6.90E+05	1.10E+05	8.4	1.0	6.3
GANAB	2	2	2	2	1	1	7.00E+05	9.60E+05	4.50E+05	4.50E+05	1.6	2.1	1.0
NAGA	2	2	1	1	2	2	8.40E+05	2.20E+05	5.50E+05	2.20E+05	3.8	1.0	2.5
GATD3B	1	1	2	2	2	2	4.30E+05	7.00E+05	4.10E+05	4.10E+05	1.0	1.7	1.0
MRPL18	2	2	2	2	1	1	1.10E+06	6.80E+05	3.40E+05	3.40E+05	3.2	2.0	1.0
FIS1	1	1	2	2	2	2	2.50E+05	5.40E+05	3.50E+05	2.50E+05	1.0	2.2	1.4
RPL35	1	1	2	2	2	2	2.30E+06	3.80E+06	2.50E+06	2.30E+06	1.0	1.7	1.1
SLC1A4	2	2	2	2	1	1	1.50E+06	4.20E+05	1.60E+05	1.60E+05	9.4	2.6	1.0
MYADM	1	1	2	2	2	2	1.50E+06	1.20E+06	8.00E+05	8.00E+05	1.9	1.5	1.0
OSBPL9	2	2	1	1	2	2	7.50E+05	1.90E+05	3.90E+05	1.90E+05	3.9	1.0	2.1
SNRPD3	2	2	1	1	2	2	1.40E+06	6.90E+04	1.00E+06	6.90E+04	20.3	1.0	14.5
MGAT1	2	2	1	1	1	2	1.20E+06	5.20E+05	8.50E+05	5.20E+05	2.3	1.0	1.6
SLC2A3	1	1	2	2	2	2	6.60E+05	5.00E+05	3.90E+05	3.90E+05	1.7	1.3	1.0
SLC26A2	1	2	2	2	0	0	1.20E+05	2.50E+05	NF	1.20E+05	1.0	2.1	0.0
H2AX	2	2	1	1	1	1	1.50E+05	3.20E+04	4.80E+04	3.20E+04	4.7	1.0	1.5
FDFT1	1	1	2	2	1	1	5.30E+05	9.50E+05	3.70E+05	3.70E+05	1.4	2.6	1.0
ZMPSTE24	1	1	2	3	0	0	5.00E+04	2.70E+05	NF	5.00E+04	1.0	5.4	0.0
NPLOC4	1	1	2	2	1	1	6.10E+04	6.20E+04	1.90E+04	1.90E+04	3.2	3.3	1.0
SPTB	2	2	1	1	1	1	7.10E+06	1.10E+06	6.00E+05	6.00E+05	11.8	1.8	1.0
RANBP6	2	2	1	1	1	1	5.60E+04	1.80E+04	1.30E+04	1.30E+04	4.3	1.4	1.0
RPA1	0	0	2	3	1	1	NF	5.60E+05	4.10E+04	4.10E+04	0.0	13.7	1.0
ATP6V1H	2	2	1	1	1	1	3.90E+05	1.20E+05	1.10E+05	1.10E+05	3.5	1.1	1.0
RPS20	1	1	1	1	2	2	4.80E+06	3.50E+06	6.10E+06	3.50E+06	1.4	1.0	1.7
HLA-C	2	2	0	0	2	2	9.50E+05	NF	6.20E+05	6.20E+05	1.5	0.0	1.0
FMR1	2	2	1	1	1	1	3.80E+05	4.20E+05	2.40E+05	2.40E+05	1.6	1.8	1.0
EFR3A	1	1	2	3	0	0	3.60E+04	3.40E+05	NF	3.60E+04	1.0	9.4	0.0
GNL3	2	2	2	2	0	0	1.30E+05	5.70E+04	NF	5.70E+04	2.3	1.0	0.0
MGAT4B	1	1	2	2	1	1	1.10E+05	2.10E+05	6.90E+04	6.90E+04	1.6	3.0	1.0

MACF1	0	0	2	2	2	2	NF	3.10E+05	3.50E+05	3.10E+05	0.0	1.0	1.1
HNRNPF	1	1	2	2	1	1	3.70E+05	7.40E+05	7.40E+04	7.40E+04	5.0	10.0	1.0
RPL14	2	2	1	1	1	1	5.50E+06	7.10E+05	3.90E+05	3.90E+05	14.1	1.8	1.0
MAIP1	2	2	1	1	1	1	1.10E+06	3.90E+05	8.40E+04	8.40E+04	13.1	4.6	1.0
RPL10	2	2	1	1	1	1	7.30E+05	8.60E+05	4.60E+05	4.60E+05	1.6	1.9	1.0
MLEC	2	2	1	1	1	1	6.10E+05	3.40E+05	2.20E+05	2.20E+05	2.8	1.5	1.0
NAXE	1	1	1	1	2	2	2.30E+05	7.20E+04	2.40E+05	7.20E+04	3.2	1.0	3.3
GPI	1	1	2	2	1	1	3.70E+05	5.40E+05	3.60E+05	3.60E+05	1.0	1.5	1.0
YWHAE	1	1	2	2	1	1	5.00E+04	5.50E+05	2.30E+04	2.30E+04	2.2	23.9	1.0
HMGCL	2	2	1	1	1	1	2.90E+05	2.30E+05	2.20E+05	2.20E+05	1.3	1.0	1.0
ANXA5	1	1	1	2	1	1	2.30E+05	2.20E+04	6.60E+03	6.60E+03	34.8	3.3	1.0
HLA-A	2	2	1	1	1	1	1.50E+06	1.40E+06	8.90E+05	8.90E+05	1.7	1.6	1.0
PRAF2	2	2	1	1	1	1	1.40E+06	7.20E+05	5.40E+05	5.40E+05	2.6	1.3	1.0
TMEM237	1	1	1	1	2	2	7.30E+05	5.90E+05	4.30E+07	5.90E+05	1.2	1.0	72.9
XPO7	0	0	2	3	1	1	NF	2.20E+05	4.50E+04	4.50E+04	0.0	4.9	1.0
CAP1	1	1	1	1	2	2	3.40E+05	3.40E+05	2.70E+05	2.70E+05	1.3	1.3	1.0
FLII	1	1	2	2	1	1	1.00E+05	1.40E+05	5.90E+04	5.90E+04	1.7	2.4	1.0
PACSIN3	1	1	2	2	1	1	3.90E+05	5.60E+05	2.30E+05	2.30E+05	1.7	2.4	1.0
AKR7A2	1	1	1	1	2	2	1.60E+05	7.90E+04	2.40E+05	7.90E+04	2.0	1.0	3.0
MTFP1	1	1	1	1	2	2	6.00E+05	4.80E+05	5.40E+05	4.80E+05	1.3	1.0	1.1
TPP1	2	2	1	1	1	1	1.30E+06	1.10E+05	9.40E+04	9.40E+04	13.8	1.2	1.0
SNTB2	2	2	1	1	1	1	7.70E+05	6.60E+05	3.80E+05	3.80E+05	2.0	1.7	1.0
SNAP23	0	0	2	2	2	2	NF	1.30E+06	5.70E+05	5.70E+05	0.0	2.3	1.0
NDUFA2	2	2	0	0	2	2	1.20E+06	NF	7.30E+05	7.30E+05	1.6	0.0	1.0
MKKS	1	1	0	0	2	3	4.10E+05	NF	8.40E+05	4.10E+05	1.0	0.0	2.0
PRXL2A	1	1	2	2	1	1	1.80E+05	2.70E+05	1.60E+05	1.60E+05	1.1	1.7	1.0
HMOX2	2	2	1	1	1	1	2.40E+05	1.00E+05	8.00E+04	8.00E+04	3.0	1.3	1.0
RRM1	1	1	2	2	1	1	1.50E+05	2.70E+05	1.60E+05	1.50E+05	1.0	1.8	1.1
TRUB2	1	1	1	1	2	2	2.10E+05	1.40E+05	1.50E+05	1.40E+05	1.5	1.0	1.1
ATP2C1	2	2	0	0	2	2	2.60E+05	NF	8.50E+04	8.50E+04	3.1	0.0	1.0
SPCS3	1	1	2	2	1	1	8.60E+05	1.20E+06	6.70E+05	6.70E+05	1.3	1.8	1.0
CHDH	1	1	1	1	2	2	1.70E+05	1.60E+05	1.60E+05	1.60E+05	1.1	1.0	1.0
ADGRG1	1	1	1	1	2	2	1.60E+05	1.40E+05	2.30E+05	1.40E+05	1.1	1.0	1.6
TMEM70	2	2	1	1	1	1	2.10E+06	3.20E+05	3.20E+05	3.20E+05	6.6	1.0	1.0
GTPBP8	1	1	1	1	2	2	7.50E+04	2.10E+04	1.20E+05	2.10E+04	3.6	1.0	5.7
TEFM	2	2	1	1	1	1	2.50E+05	3.20E+04	5.10E+04	3.20E+04	7.8	1.0	1.6
CDS2	0	0	2	2	2	2	NF	1.60E+05	1.70E+05	1.60E+05	0.0	1.0	1.1
SLC12A9	0	0	2	2	2	2	NF	4.10E+04	3.40E+04	3.40E+04	0.0	1.2	1.0
SLC5A6	1	1	2	2	1	1	6.80E+05	6.20E+05	3.00E+05	3.00E+05	2.3	2.1	1.0
SYNJ2BP	2	2	2	2	0	0	1.30E+06	5.50E+05	NF	5.50E+05	2.4	1.0	0.0
CDKAL1	1	1	1	1	2	2	1.00E+05	1.10E+05	2.50E+05	1.00E+05	1.0	1.1	2.5
MRPL54	2	2	1	1	1	1	1.30E+06	7.20E+05	6.60E+05	6.60E+05	2.0	1.1	1.0
SEC23A	0	0	2	2	1	2	NF	3.80E+04	1.50E+04	1.50E+04	0.0	2.5	1.0
EXOC2	1	1	2	2	1	1	4.40E+04	1.50E+06	8.60E+04	4.40E+04	1.0	34.1	2.0
PLK1	1	1	2	2	1	1	4.40E+05	4.80E+05	2.20E+05	2.20E+05	2.0	2.2	1.0
CASK	1	1	1	1	2	2	2.60E+05	3.00E+05	3.70E+05	2.60E+05	1.0	1.2	1.4
MRPL58	2	2	1	1	1	1	1.80E+06	9.90E+05	8.00E+05	8.00E+05	2.3	1.2	1.0
NUP85	2	2	1	2	0	0	6.10E+05	3.20E+05	NF	3.20E+05	1.9	1.0	0.0
IGF2BP1	1	1	2	2	1	1	3.80E+05	3.90E+05	9.10E+04	9.10E+04	4.2	4.3	1.0
SLC6A15	0	0	1	1	2	3	NF	1.30E+05	5.10E+05	1.30E+05	0.0	1.0	3.9
SAFB	0	0	2	2	2	2	NF	1.00E+06	5.30E+05	5.30E+05	0.0	1.9	1.0

TMEM14C	1	1	1	1	1	2	7.60E+03	1.20E+04	1.90E+04	7.60E+03	1.0	1.6	2.5
DNMT1	1	1	2	3	0	0	1.40E+05	9.80E+04	NF	9.80E+04	1.4	1.0	0.0
DDX39A	0	0	2	2	2	2	NF	7.70E+05	4.90E+05	4.90E+05	0.0	1.6	1.0
CD9	1	1	1	1	2	2	3.70E+06	3.00E+06	2.40E+06	2.40E+06	1.5	1.3	1.0
ITGA3	1	1	2	2	1	1	5.90E+05	7.10E+05	3.30E+05	3.30E+05	1.8	2.2	1.0
CAPZA2	2	2	1	1	1	1	2.00E+06	1.40E+06	1.20E+06	1.20E+06	1.7	1.2	1.0
MRPL10	2	2	1	1	1	1	1.10E+06	7.70E+05	5.70E+05	5.70E+05	1.9	1.4	1.0
STX12	1	1	1	1	2	2	2.90E+05	3.60E+05	3.50E+05	2.90E+05	1.0	1.2	1.2
LETMD1	1	1	1	1	2	2	4.20E+05	3.90E+05	4.30E+05	3.90E+05	1.1	1.0	1.1
RBM14	2	2	1	1	1	1	5.90E+05	2.30E+05	2.60E+05	2.30E+05	2.6	1.0	1.1
NDUFS7	2	3	1	1	0	0	2.40E+06	7.60E+05	NF	7.60E+05	3.2	1.0	0.0
PPIA	0	0	2	2	2	2	NF	2.00E+06	9.90E+05	9.90E+05	0.0	2.0	1.0
ACACB	0	0	2	2	2	2	NF	3.50E+05	2.90E+05	2.90E+05	0.0	1.2	1.0
RPL27A	0	0	2	2	2	2	NF	4.20E+06	2.20E+06	2.20E+06	0.0	1.9	1.0
SPRYD4	2	2	1	1	1	1	1.30E+06	4.70E+05	3.90E+05	3.90E+05	3.3	1.2	1.0
RPL36	2	2	1	1	1	1	1.10E+06	5.70E+05	1.70E+05	1.70E+05	6.5	3.4	1.0
RAC1	1	1	1	2	1	1	5.90E+05	1.00E+06	3.30E+05	3.30E+05	1.8	3.0	1.0
MRPS16	0	0	2	2	2	2	NF	2.90E+06	1.60E+06	1.60E+06	0.0	1.8	1.0
WARS2	1	1	1	1	2	2	5.10E+05	4.20E+04	3.50E+05	4.20E+04	12.1	1.0	8.3
MPP7	0	0	2	2	2	2	NF	6.60E+05	4.40E+05	4.40E+05	0.0	1.5	1.0
LIMS1	1	1	2	2	1	1	3.60E+05	1.40E+06	2.60E+05	2.60E+05	1.4	5.4	1.0
GPX8	2	2	1	1	1	1	2.50E+06	3.20E+05	1.90E+05	1.90E+05	13.2	1.7	1.0
NDUFA13	0	0	2	2	2	2	NF	1.20E+06	8.50E+05	8.50E+05	0.0	1.4	1.0
UFD1	2	2	2	2	0	0	7.70E+05	5.00E+05	NF	5.00E+05	1.5	1.0	0.0
SYNGR2	1	1	1	1	2	2	7.20E+05	5.70E+05	9.80E+05	5.70E+05	1.3	1.0	1.7
BCKDHB	1	1	1	1	1	1	8.50E+04	1.00E+04	1.10E+04	1.00E+04	8.5	1.0	1.1
CKB	1	1	1	1	1	1	7.60E+03	2.50E+05	1.60E+05	7.60E+03	1.0	32.9	21.1
YWHAG	0	0	1	1	2	2	NF	7.50E+04	2.20E+06	7.50E+04	0.0	1.0	29.3
ACTR1A	1	1	1	1	1	1	8.20E+04	3.90E+05	1.90E+05	8.20E+04	1.0	4.8	2.3
HSD17B11	1	1	1	1	1	1	1.30E+05	3.20E+05	1.60E+05	1.30E+05	1.0	2.5	1.2
PLEKHG3	2	2	1	1	0	0	2.70E+05	5.50E+04	NF	5.50E+04	4.9	1.0	0.0
PNPLA4	1	1	1	1	1	1	2.30E+05	2.50E+05	6.30E+04	6.30E+04	3.7	4.0	1.0
M6PR	2	2	0	0	1	1	7.90E+05	NF	1.70E+05	1.70E+05	4.6	0.0	1.0
STRAP	0	0	2	2	1	1	NF	4.50E+05	1.30E+05	1.30E+05	0.0	3.5	1.0
GUF1	2	2	0	0	1	1	3.70E+05	NF	6.40E+04	6.40E+04	5.8	0.0	1.0
AFAP1	0	0	2	2	1	1	NF	9.30E+05	3.70E+05	3.70E+05	0.0	2.5	1.0
DDX50	1	2	1	1	0	0	7.20E+05	8.70E+05	NF	7.20E+05	1.0	1.2	0.0
RAB35	2	2	1	1	0	0	3.80E+05	4.50E+04	NF	4.50E+04	8.4	1.0	0.0
AP1B1	2	2	0	0	1	1	1.80E+05	NF	6.00E+04	6.00E+04	3.0	0.0	1.0
DHX36	1	1	1	1	1	1	2.10E+05	7.40E+04	1.80E+04	1.80E+04	11.7	4.1	1.0
TGFB1	1	1	1	1	1	1	1.30E+05	2.50E+04	1.30E+05	2.50E+04	5.2	1.0	5.2
PRPF31	2	2	1	1	0	0	9.30E+05	5.20E+04	NF	5.20E+04	17.9	1.0	0.0
CNTNAP1	2	3	0	0	0	0	7.30E+05	NF	NF	7.30E+05	1.0	0.0	0.0
NIPSNAP3A	2	2	0	0	1	1	5.60E+05	NF	7.70E+04	7.70E+04	7.3	0.0	1.0
IQGAP3	1	1	2	2	0	0	1.10E+04	6.50E+04	NF	1.10E+04	1.0	5.9	0.0
VPS53	2	2	1	1	0	0	7.50E+04	1.60E+04	NF	1.60E+04	4.7	1.0	0.0
ATPAF2	2	2	1	1	0	0	4.30E+05	2.00E+04	NF	2.00E+04	21.5	1.0	0.0
ARF6	0	0	2	2	1	1	NF	5.30E+05	4.40E+04	4.40E+04	0.0	12.0	1.0
POGLUT2	1	1	1	1	1	1	3.70E+05	3.30E+04	3.00E+04	3.00E+04	12.3	1.1	1.0
NIF3L1	1	1	1	1	1	1	2.30E+05	2.90E+05	2.50E+05	2.30E+05	1.0	1.3	1.1
LUZP1	1	1	2	2	0	0	2.20E+05	5.60E+05	NF	2.20E+05	1.0	2.5	0.0

GRHPR	2	2	1	1	0	0	1.30E+06	3.30E+05	NF	3.30E+05	3.9	1.0	0.0
DHX29	1	1	2	2	0	0	1.30E+04	1.80E+05	NF	1.30E+04	1.0	13.8	0.0
NUDT9	2	2	1	1	0	0	7.60E+05	3.20E+05	NF	3.20E+05	2.4	1.0	0.0
ATP6AP2	1	1	1	2	0	0	9.00E+04	1.90E+06	NF	9.00E+04	1.0	21.1	0.0
ITM2C	0	0	1	1	2	2	NF	1.80E+05	4.40E+05	1.80E+05	0.0	1.0	2.4
TRMT61B	2	2	1	1	0	0	6.70E+05	1.70E+05	NF	1.70E+05	3.9	1.0	0.0
SPNS1	2	2	0	0	1	1	1.20E+06	NF	6.10E+05	6.10E+05	2.0	0.0	1.0
PPP2R1B	0	0	2	2	1	1	NF	1.50E+05	2.40E+04	2.40E+04	0.0	6.3	1.0
AHCY	1	1	2	2	0	0	5.30E+05	1.20E+06	NF	5.30E+05	1.0	2.3	0.0
PPP1CA	1	1	1	1	1	1	7.60E+05	6.40E+05	2.80E+05	2.80E+05	2.7	2.3	1.0
ITGA5	0	0	1	1	2	2	NF	1.10E+06	6.40E+05	6.40E+05	0.0	1.7	1.0
NOP2	0	0	2	2	1	1	NF	2.60E+05	7.00E+04	7.00E+04	0.0	3.7	1.0
FNDC3A	1	2	0	0	1	1	9.10E+05	NF	5.50E+04	5.50E+04	16.5	0.0	1.0
TEX10	1	1	2	2	0	0	6.80E+03	6.40E+04	NF	6.80E+03	1.0	9.4	0.0
ITM2B	1	1	2	2	0	0	6.30E+05	8.20E+05	NF	6.30E+05	1.0	1.3	0.0
P3H4	1	1	0	0	2	2	1.80E+05	NF	4.10E+05	1.80E+05	1.0	0.0	2.3
IVD	2	2	0	0	1	1	2.00E+06	NF	1.10E+06	1.10E+06	1.8	0.0	1.0
TMEM11	1	1	2	2	0	0	5.20E+05	1.10E+06	NF	5.20E+05	1.0	2.1	0.0
LAPTM4A	2	3	0	0	0	0	5.30E+05	NF	NF	5.30E+05	1.0	0.0	0.0
PARD3	0	0	1	1	2	2	NF	1.10E+05	4.60E+05	1.10E+05	0.0	1.0	4.2
MYBBP1A	0	0	2	2	1	1	NF	2.10E+05	4.00E+04	4.00E+04	0.0	5.3	1.0
NPTN	1	1	2	2	0	0	1.00E+06	7.50E+05	NF	7.50E+05	1.3	1.0	0.0
TMEM263	0	0	1	1	2	2	NF	2.20E+05	6.50E+05	2.20E+05	0.0	1.0	3.0
MCM2	1	1	2	2	0	0	4.60E+05	7.90E+05	NF	4.60E+05	1.0	1.7	0.0
EPB41	2	2	1	1	0	0	2.00E+06	4.40E+05	NF	4.40E+05	4.5	1.0	0.0
CNNM3	0	0	1	1	2	2	NF	1.70E+05	1.20E+05	1.20E+05	0.0	1.4	1.0
ATXN10	0	0	2	2	1	1	NF	4.50E+05	1.40E+05	1.40E+05	0.0	3.2	1.0
RALGAPA1	1	1	2	2	0	0	5.60E+04	1.60E+05	NF	5.60E+04	1.0	2.9	0.0
	1	1	1	1	1	1	8.50E+05	3.40E+05	6.20E+05	3.40E+05	2.5	1.0	1.8
TST	1	1	0	0	2	2	4.60E+05	NF	9.90E+05	4.60E+05	1.0	0.0	2.2
SRSF1	0	0	2	2	1	1	NF	2.90E+05	1.70E+05	1.70E+05	0.0	1.7	1.0
POGLUT3	0	0	2	2	1	1	NF	2.70E+05	2.90E+04	2.90E+04	0.0	9.3	1.0
NSUN5	2	2	1	1	0	0	4.70E+05	3.80E+05	NF	3.80E+05	1.2	1.0	0.0
PSMC2	0	0	2	2	1	1	NF	2.90E+05	2.80E+04	2.80E+04	0.0	10.4	1.0
TUBAL3	0	0	2	2	1	1	NF	1.40E+06	3.00E+05	3.00E+05	0.0	4.7	1.0
SPTBN5	0	0	1	1	2	2	NF	2.80E+04	2.00E+05	2.80E+04	0.0	1.0	7.1
SLC12A6	0	0	2	2	1	1	NF	5.60E+05	1.30E+05	1.30E+05	0.0	4.3	1.0
	0	0	0	0	2	2	NF	NF	4.00E+05	4.00E+05	0.0	0.0	1.0
MRPS10	1	1	0	0	1	1	1.80E+05	NF	8.80E+04	8.80E+04	2.0	0.0	1.0
CTSA	0	0	0	0	2	2	NF	NF	1.70E+06	1.70E+06	0.0	0.0	1.0
TPRN	0	0	1	1	1	1	NF	9.20E+04	2.20E+05	9.20E+04	0.0	1.0	2.4
PSMA7	0	0	2	2	0	0	NF	4.20E+05	NF	4.20E+05	0.0	1.0	0.0
FUBP3	0	0	0	0	2	2	NF	NF	4.00E+05	4.00E+05	0.0	0.0	1.0
RFC4	0	0	2	2	0	0	NF	4.10E+05	NF	4.10E+05	0.0	1.0	0.0
UTRN	0	0	2	2	0	0	NF	7.50E+04	NF	7.50E+04	0.0	1.0	0.0
MRPS36	0	0	2	2	0	0	NF	2.10E+05	NF	2.10E+05	0.0	1.0	0.0
MT-ND1	0	0	0	0	2	2	NF	NF	5.80E+05	5.80E+05	0.0	0.0	1.0
MGAT2	2	2	0	0	0	0	6.30E+05	NF	NF	6.30E+05	1.0	0.0	0.0
ACACA	2	2	0	0	0	0	9.00E+04	NF	NF	9.00E+04	1.0	0.0	0.0
ARL8B	1	1	1	1	0	0	1.00E+05	9.00E+04	NF	9.00E+04	1.1	1.0	0.0
COX11	1	1	0	0	1	1	4.40E+03	NF	2.10E+04	4.40E+03	1.0	0.0	4.8

EIF3M	2	2	0	0	0	0	3.50E+05	NF	NF	3.50E+05	1.0	0.0	0.0
P3H2	0	0	2	2	0	0	NF	6.60E+05	NF	6.60E+05	0.0	1.0	0.0
ABCC5	0	0	1	1	1	1	NF	3.40E+05	9.70E+04	9.70E+04	0.0	3.5	1.0
EIF1AX	2	2	0	0	0	0	9.70E+05	NF	NF	9.70E+05	1.0	0.0	0.0
HMGA1	0	0	2	2	0	0	NF	1.40E+06	NF	1.40E+06	0.0	1.0	0.0
MCM4	0	0	2	2	0	0	NF	4.40E+05	NF	4.40E+05	0.0	1.0	0.0
SF3A1	0	0	2	2	0	0	NF	1.90E+05	NF	1.90E+05	0.0	1.0	0.0
PSMC3	0	0	2	2	0	0	NF	1.80E+05	NF	1.80E+05	0.0	1.0	0.0
GALNS	2	2	0	0	0	0	2.80E+05	NF	NF	2.80E+05	1.0	0.0	0.0
HAX1	0	0	2	2	0	0	NF	4.50E+05	NF	4.50E+05	0.0	1.0	0.0
MRPS21	0	0	1	1	1	1	NF	4.50E+05	3.00E+05	3.00E+05	0.0	1.5	1.0
CPNE8	0	0	2	2	0	0	NF	4.20E+05	NF	4.20E+05	0.0	1.0	0.0
TPI1	0	0	2	2	0	0	NF	5.70E+05	NF	5.70E+05	0.0	1.0	0.0
TKT	0	0	2	2	0	0	NF	3.60E+05	NF	3.60E+05	0.0	1.0	0.0
HINT2	2	2	0	0	0	0	4.30E+05	NF	NF	4.30E+05	1.0	0.0	0.0
CPOX	0	0	1	1	1	1	NF	1.30E+05	5.70E+04	5.70E+04	0.0	2.3	1.0
ATP13A3	0	0	0	0	2	2	NF	NF	4.20E+05	4.20E+05	0.0	0.0	1.0
UMPS	0	0	2	2	0	0	NF	2.50E+05	NF	2.50E+05	0.0	1.0	0.0
ANKFY1	0	0	2	2	0	0	NF	1.60E+05	NF	1.60E+05	0.0	1.0	0.0
PSMD3	0	0	2	2	0	0	NF	4.30E+05	NF	4.30E+05	0.0	1.0	0.0
NECTIN2	0	0	2	2	0	0	NF	8.20E+04	NF	8.20E+04	0.0	1.0	0.0
LRP10	0	0	2	2	0	0	NF	2.70E+05	NF	2.70E+05	0.0	1.0	0.0
PVR	2	2	0	0	0	0	1.10E+06	NF	NF	1.10E+06	1.0	0.0	0.0
PBXIP1	2	2	0	0	0	0	1.30E+05	NF	NF	1.30E+05	1.0	0.0	0.0
MAVS	1	1	1	1	0	0	1.20E+05	3.10E+05	NF	1.20E+05	1.0	2.6	0.0
NOP56	0	0	2	2	0	0	NF	5.40E+05	NF	5.40E+05	0.0	1.0	0.0
KPNA3	0	0	0	0	2	2	NF	NF	5.90E+04	5.90E+04	0.0	0.0	1.0
NDUFB10	2	2	0	0	0	0	1.70E+06	NF	NF	1.70E+06	1.0	0.0	0.0
DIAPH1	0	0	2	2	0	0	NF	4.90E+05	NF	4.90E+05	0.0	1.0	0.0
MRPL52	2	2	0	0	0	0	1.50E+05	NF	NF	1.50E+05	1.0	0.0	0.0
CYFIP1	0	0	2	2	0	0	NF	2.90E+05	NF	2.90E+05	0.0	1.0	0.0
RAB11FIP1	2	2	0	0	0	0	2.70E+05	NF	NF	2.70E+05	1.0	0.0	0.0
HNRNPUL2	1	1	1	1	0	0	4.80E+05	2.10E+05	NF	2.10E+05	2.3	1.0	0.0
CEMIP2	2	2	0	0	0	0	4.70E+05	NF	NF	4.70E+05	1.0	0.0	0.0
MRPS11	0	0	0	0	2	2	NF	NF	4.00E+05	4.00E+05	0.0	0.0	1.0
DHRS4	0	0	0	0	2	2	NF	NF	4.00E+05	4.00E+05	0.0	0.0	1.0
DUS2	0	0	1	1	1	1	NF	6.00E+04	1.80E+05	6.00E+04	0.0	1.0	3.0
SYNM	1	1	1	1	0	0	5.10E+05	6.90E+04	NF	6.90E+04	7.4	1.0	0.0
IDI1	1	1	1	1	0	0	2.50E+05	2.40E+04	NF	2.40E+04	10.4	1.0	0.0
ALPI	2	2	0	0	0	0	2.60E+06	NF	NF	2.60E+06	1.0	0.0	0.0
DCBLD1	2	2	0	0	0	0	5.00E+05	NF	NF	5.00E+05	1.0	0.0	0.0
LSG1	0	0	2	2	0	0	NF	5.40E+05	NF	5.40E+05	0.0	1.0	0.0
LYRM7	0	0	0	0	2	2	NF	NF	4.40E+05	4.40E+05	0.0	0.0	1.0
ALDH5A1	2	2	0	0	0	0	9.00E+05	NF	NF	9.00E+05	1.0	0.0	0.0
NOL11	0	0	2	2	0	0	NF	4.90E+05	NF	4.90E+05	0.0	1.0	0.0
CERS2	2	2	0	0	0	0	1.10E+06	NF	NF	1.10E+06	1.0	0.0	0.0
GATB	0	0	0	0	2	2	NF	NF	3.30E+05	3.30E+05	0.0	0.0	1.0
TMEM97	0	0	2	2	0	0	NF	7.10E+05	NF	7.10E+05	0.0	1.0	0.0
HSDL1	2	2	0	0	0	0	1.40E+06	NF	NF	1.40E+06	1.0	0.0	0.0
LONP2	1	1	1	1	0	0	1.10E+05	6.70E+04	NF	6.70E+04	1.6	1.0	0.0
TBC1D10B	0	0	2	2	0	0	NF	1.30E+05	NF	1.30E+05	0.0	1.0	0.0

VPS4B	0	0	2	2	0	0	NF	1.80E+05	NF	1.80E+05	0.0	1.0	0.0
PIP4P1	2	2	0	0	0	0	1.20E+06	NF	NF	1.20E+06	1.0	0.0	0.0
NDUFAF2	2	2	0	0	0	0	2.70E+05	NF	NF	2.70E+05	1.0	0.0	0.0
ISCA1	0	0	0	0	2	2	NF	NF	1.20E+06	1.20E+06	0.0	0.0	1.0
PLD1	0	0	2	2	0	0	NF	3.00E+05	NF	3.00E+05	0.0	1.0	0.0
COX15	2	2	0	0	0	0	2.20E+06	NF	NF	2.20E+06	1.0	0.0	0.0
SNX3	0	0	0	0	2	2	NF	NF	2.20E+05	2.20E+05	0.0	0.0	1.0
PPP1R9B	0	0	2	2	0	0	NF	4.10E+05	NF	4.10E+05	0.0	1.0	0.0
NIPSNAP2	2	2	0	0	0	0	5.20E+06	NF	NF	5.20E+06	1.0	0.0	0.0
NLRX1	2	2	0	0	0	0	2.30E+05	NF	NF	2.30E+05	1.0	0.0	0.0
TM9SF3	0	0	2	2	0	0	NF	2.80E+06	NF	2.80E+06	0.0	1.0	0.0
DNAJB1	0	0	0	0	2	2	NF	NF	1.50E+05	1.50E+05	0.0	0.0	1.0
A2M	0	0	0	0	2	2	NF	NF	9.50E+05	9.50E+05	0.0	0.0	1.0
EIF4G3	2	2	0	0	0	0	1.00E+06	NF	NF	1.00E+06	1.0	0.0	0.0
OSBPL11	0	0	2	2	0	0	NF	4.40E+05	NF	4.40E+05	0.0	1.0	0.0
SORBS2	2	2	0	0	0	0	1.20E+06	NF	NF	1.20E+06	1.0	0.0	0.0
PIGT	0	0	0	0	2	2	NF	NF	8.90E+05	8.90E+05	0.0	0.0	1.0
TRMU	2	2	0	0	0	0	7.00E+05	NF	NF	7.00E+05	1.0	0.0	0.0
LEMD2	0	0	2	2	0	0	NF	5.30E+05	NF	5.30E+05	0.0	1.0	0.0
RPAP3	0	0	2	2	0	0	NF	5.20E+05	NF	5.20E+05	0.0	1.0	0.0
CD59	1	3	1	4	1	3	5.40E+06	6.80E+06	2.50E+06	2.50E+06	2.2	2.7	1.0
RAB13	1	2	1	3	1	3	1.10E+07	2.20E+07	1.20E+07	1.10E+07	1.0	2.0	1.1
NEFL	1	2	1	4	1	2	1.10E+08	2.30E+08	5.20E+07	5.20E+07	2.1	4.4	1.0
BST2	1	2	1	2	1	3	2.40E+06	2.60E+06	2.60E+06	2.40E+06	1.0	1.1	1.1
CDC42	1	1	1	3	1	2	5.90E+05	6.40E+05	3.40E+05	3.40E+05	1.7	1.9	1.0
KCT2	1	2	1	2	1	2	8.80E+05	1.10E+06	8.70E+05	8.70E+05	1.0	1.3	1.0
GNB3	1	2	1	2	1	2	4.60E+06	4.90E+06	3.60E+06	3.60E+06	1.3	1.4	1.0
PAM16	1	2	1	2	1	1	4.00E+05	6.90E+05	2.00E+05	2.00E+05	2.0	3.5	1.0
RPL29	1	2	1	1	1	2	6.70E+06	4.20E+06	6.20E+06	4.20E+06	1.6	1.0	1.5
AIMP1	1	1	1	2	1	2	3.20E+05	8.90E+05	4.40E+05	3.20E+05	1.0	2.8	1.4
COPG2	1	1	1	2	1	2	7.90E+05	3.90E+05	3.20E+05	3.20E+05	2.5	1.2	1.0
MT-ATP6	1	2	1	1	1	2	5.60E+06	3.40E+06	5.20E+06	3.40E+06	1.6	1.0	1.5
MFSD10	1	1	1	1	1	3	7.60E+03	6.40E+03	1.90E+04	6.40E+03	1.2	1.0	3.0
PSEN1	1	2	1	1	1	1	5.80E+05	2.40E+05	1.60E+05	1.60E+05	3.6	1.5	1.0
RPL37A	1	1	1	2	1	1	1.40E+06	3.50E+06	9.60E+05	9.60E+05	1.5	3.6	1.0
MARCHF5	1	1	1	1	1	2	3.80E+05	1.70E+05	9.30E+04	9.30E+04	4.1	1.8	1.0
TIMM17B	1	1	1	2	1	1	4.60E+05	3.40E+05	1.50E+05	1.50E+05	3.1	2.3	1.0
IST1	1	2	0	0	1	2	1.80E+05	NF	4.70E+04	4.70E+04	3.8	0.0	1.0
RHBDD2	1	1	1	1	1	2	5.20E+05	1.30E+05	2.00E+05	1.30E+05	4.0	1.0	1.5
RPL21	1	1	1	1	1	2	3.40E+06	2.50E+06	2.10E+06	2.10E+06	1.6	1.2	1.0
SCAMP2	1	1	1	1	1	2	3.70E+05	2.20E+05	3.20E+05	2.20E+05	1.7	1.0	1.5
FCHO2	0	0	1	2	1	2	NF	1.00E+05	1.10E+05	1.00E+05	0.0	1.0	1.1
NDUFB4	1	1	1	2	1	1	4.80E+05	9.70E+05	4.10E+05	4.10E+05	1.2	2.4	1.0
DIDO1	1	2	1	1	1	1	2.00E+04	1.60E+04	9.30E+03	9.30E+03	2.2	1.7	1.0
KRTCAP2	1	2	1	1	1	1	1.10E+06	6.30E+05	4.20E+05	4.20E+05	2.6	1.5	1.0
LRRC47	1	1	1	2	1	1	7.10E+04	2.20E+05	2.90E+04	2.90E+04	2.4	7.6	1.0
STX10	1	1	1	2	1	1	4.10E+05	6.30E+05	2.40E+05	2.40E+05	1.7	2.6	1.0
PLP2	1	2	1	1	1	1	6.90E+06	3.50E+06	1.50E+06	1.50E+06	4.6	2.3	1.0
RAB29	1	1	1	1	1	2	7.50E+05	5.10E+05	6.70E+05	5.10E+05	1.5	1.0	1.3
TIMMDC1	1	1	1	2	1	1	4.40E+05	4.10E+05	1.70E+05	1.70E+05	2.6	2.4	1.0
SYPL1	1	1	1	1	1	2	2.80E+06	1.80E+06	2.80E+06	1.80E+06	1.6	1.0	1.6

TMEM65	1	2	1	1	1	1	1.10E+06	2.80E+05	2.60E+05	2.60E+05	4.2	1.1	1.0
ECPAS	1	1	1	1	1	2	1.40E+05	1.30E+05	7.00E+04	7.00E+04	2.0	1.9	1.0
MRPS15	1	1	1	2	1	1	8.00E+05	1.50E+06	6.80E+05	6.80E+05	1.2	2.2	1.0
FARSA	1	1	1	1	1	2	3.80E+05	2.40E+05	3.70E+05	2.40E+05	1.6	1.0	1.5
SEPTIN6	1	1	1	1	1	2	2.90E+05	2.50E+05	3.00E+05	2.50E+05	1.2	1.0	1.2
CD55	1	1	1	2	1	1	1.80E+06	5.20E+06	1.60E+06	1.60E+06	1.1	3.3	1.0
TOMM7	1	2	1	2	0	0	4.00E+05	3.70E+05	NF	3.70E+05	1.1	1.0	0.0
PLPP2	1	1	1	1	1	1	7.00E+04	1.90E+04	1.30E+04	1.30E+04	5.4	1.5	1.0
RHOC	1	1	1	1	1	1	2.20E+05	2.10E+05	9.50E+04	9.50E+04	2.3	2.2	1.0
HNRNPC	1	1	1	1	1	1	2.40E+05	1.80E+05	9.50E+04	9.50E+04	2.5	1.9	1.0
BRI3BP	1	1	1	1	1	1	8.10E+04	6.80E+04	6.20E+04	6.20E+04	1.3	1.1	1.0
DAZAP1	1	2	1	1	0	0	2.90E+05	1.10E+05	NF	1.10E+05	2.6	1.0	0.0
SERINC1	1	1	1	1	1	1	7.60E+05	9.40E+05	5.80E+05	5.80E+05	1.3	1.6	1.0
SDR39U1	1	1	1	1	1	1	1.50E+05	8.40E+04	8.90E+04	8.40E+04	1.8	1.0	1.1
FUNDC1	1	1	1	1	1	1	4.50E+04	2.90E+04	8.30E+03	8.30E+03	5.4	3.5	1.0
TIMM13	1	1	1	1	1	1	3.80E+05	5.60E+05	3.50E+05	3.50E+05	1.1	1.6	1.0
TMEM106C	0	0	1	1	1	2	NF	7.50E+04	2.30E+05	7.50E+04	0.0	1.0	3.1
CD276	1	1	1	1	1	1	7.10E+04	1.80E+05	1.30E+05	7.10E+04	1.0	2.5	1.8
ALG1	1	1	1	1	1	1	2.40E+05	4.90E+04	4.70E+04	4.70E+04	5.1	1.0	1.0
OCIAD2	1	1	1	1	1	1	2.00E+05	9.00E+04	6.10E+04	6.10E+04	3.3	1.5	1.0
IER3IP1	1	1	1	1	1	1	4.80E+05	2.80E+05	2.60E+05	2.60E+05	1.8	1.1	1.0
VRK2	1	1	1	1	1	1	8.90E+04	4.60E+04	6.10E+04	4.60E+04	1.9	1.0	1.3
LMO7	1	1	1	1	1	1	1.20E+06	8.90E+05	4.50E+05	4.50E+05	2.7	2.0	1.0
MAN2B1	1	1	1	1	1	1	1.00E+05	6.50E+04	3.80E+04	3.80E+04	2.6	1.7	1.0
BDH1	1	1	1	1	1	1	1.90E+05	1.20E+05	8.70E+04	8.70E+04	2.2	1.4	1.0
PLSCR1	0	0	1	1	1	2	NF	1.60E+04	1.40E+04	1.40E+04	0.0	1.1	1.0
PCK1	1	1	1	1	1	1	5.00E+05	3.40E+05	2.00E+05	2.00E+05	2.5	1.7	1.0
EVA1B	1	1	1	1	1	1	4.80E+04	2.20E+04	2.20E+04	2.20E+04	2.2	1.0	1.0
NCKAP1	1	1	1	1	1	1	2.60E+05	2.20E+05	1.50E+05	1.50E+05	1.7	1.5	1.0
CHCHD2	1	1	1	1	1	1	2.70E+06	1.00E+06	9.10E+05	9.10E+05	3.0	1.1	1.0
LYZ	1	1	1	1	1	1	3.50E+05	5.00E+05	5.50E+05	3.50E+05	1.0	1.4	1.6
SLC1A3	1	1	1	1	1	1	1.50E+05	1.40E+05	5.70E+04	5.70E+04	2.6	2.5	1.0
BAX	1	1	1	1	1	1	2.70E+05	1.50E+05	1.20E+05	1.20E+05	2.3	1.3	1.0
TRA2B	0	0	1	1	1	2	NF	3.20E+05	3.00E+05	3.00E+05	0.0	1.1	1.0
MAGT1	1	1	1	1	1	1	2.10E+05	8.50E+04	5.30E+04	5.30E+04	4.0	1.6	1.0
MSMO1	1	1	0	0	1	2	2.20E+06	NF	2.70E+06	2.20E+06	1.0	0.0	1.2
ATP5F1D	1	1	1	1	1	1	6.00E+05	7.00E+05	5.00E+05	5.00E+05	1.2	1.4	1.0
STAG2	1	1	1	1	1	1	1.10E+04	1.60E+04	6.60E+03	6.60E+03	1.7	2.4	1.0
SCD	1	1	1	1	1	1	1.50E+06	2.10E+06	1.30E+06	1.30E+06	1.2	1.6	1.0
DYSF	1	1	1	1	1	1	8.80E+05	6.80E+05	3.40E+05	3.40E+05	2.6	2.0	1.0
B4GAT1	1	1	1	1	1	1	3.00E+05	3.70E+05	2.30E+05	2.30E+05	1.3	1.6	1.0
CTSZ	1	1	1	1	1	1	3.00E+06	1.30E+06	1.80E+06	1.30E+06	2.3	1.0	1.4
ADAR	1	1	1	1	1	1	6.90E+05	2.30E+05	1.80E+05	1.80E+05	3.8	1.3	1.0
CCDC134	1	1	1	1	1	1	9.70E+04	8.10E+04	6.90E+04	6.90E+04	1.4	1.2	1.0
TMBIM1	1	1	1	1	1	1	3.40E+05	4.00E+05	2.10E+05	2.10E+05	1.6	1.9	1.0
CLCC1	0	0	1	2	1	1	NF	1.60E+05	8.10E+04	8.10E+04	0.0	2.0	1.0
SSR3	1	1	1	1	1	1	1.80E+06	8.00E+05	6.50E+05	6.50E+05	2.8	1.2	1.0
GOLPH3	1	1	1	1	1	1	3.60E+05	1.70E+05	1.30E+05	1.30E+05	2.8	1.3	1.0
USP39	1	1	1	1	1	1	5.10E+04	6.80E+04	6.20E+04	5.10E+04	1.0	1.3	1.2
BACE2	1	1	1	1	1	1	4.40E+05	4.80E+05	3.20E+05	3.20E+05	1.4	1.5	1.0
RAVER1	1	1	1	1	1	1	8.10E+04	1.20E+05	8.70E+04	8.10E+04	1.0	1.5	1.1

EIF6	1	1	1	1	1	1	1.80E+05	6.40E+04	5.20E+04	5.20E+04	3.5	1.2	1.0
YWHAH	1	1	1	1	1	1	3.10E+05	4.50E+05	2.50E+05	2.50E+05	1.2	1.8	1.0
SLC30A1	1	1	1	1	1	1	4.10E+05	3.00E+05	1.60E+05	1.60E+05	2.6	1.9	1.0
CLASP2	1	1	1	1	1	1	1.20E+05	1.20E+05	5.60E+04	5.60E+04	2.1	2.1	1.0
PUM1	1	1	1	1	1	1	1.40E+05	1.10E+05	8.00E+04	8.00E+04	1.8	1.4	1.0
GNG12	1	1	1	1	1	1	1.00E+06	9.20E+05	4.80E+05	4.80E+05	2.1	1.9	1.0
H2AC17	1	1	1	1	1	1	4.40E+06	1.80E+06	8.90E+05	8.90E+05	4.9	2.0	1.0
PIP5K1A	1	1	1	1	1	1	5.20E+05	5.50E+05	2.80E+05	2.80E+05	1.9	2.0	1.0
TIMM22	1	1	1	1	1	1	5.50E+05	7.30E+05	4.90E+05	4.90E+05	1.1	1.5	1.0
RPS15	1	1	1	1	1	1	5.30E+03	8.60E+03	3.00E+03	3.00E+03	1.8	2.9	1.0
CTPS2	1	1	1	1	1	1	2.70E+05	2.20E+05	1.30E+05	1.30E+05	2.1	1.7	1.0
HLA-H	1	1	1	1	1	1	4.90E+06	4.30E+06	3.30E+06	3.30E+06	1.5	1.3	1.0
RAB12	1	1	1	1	1	1	1.90E+05	1.00E+05	8.40E+04	8.40E+04	2.3	1.2	1.0
GNB1	1	1	1	1	1	1	5.20E+06	2.00E+06	1.40E+06	1.40E+06	3.7	1.4	1.0
DCTN1	0	0	1	1	1	2	NF	6.40E+04	4.60E+04	4.60E+04	0.0	1.4	1.0
ARPC4	1	1	1	1	1	1	3.30E+05	3.60E+05	1.70E+05	1.70E+05	1.9	2.1	1.0
TNPO1	1	1	1	1	1	1	2.60E+05	2.30E+05	2.30E+05	2.30E+05	1.1	1.0	1.0
NEGR1	1	1	1	1	1	1	1.50E+06	7.20E+05	7.60E+05	7.20E+05	2.1	1.0	1.1
METTL7A	1	1	1	1	1	1	2.50E+05	1.90E+05	1.30E+05	1.30E+05	1.9	1.5	1.0
ADCY9	1	1	1	1	1	1	3.00E+05	2.70E+05	1.90E+05	1.90E+05	1.6	1.4	1.0
VANGL1	1	1	1	1	1	1	2.50E+05	3.30E+05	1.40E+05	1.40E+05	1.8	2.4	1.0
RABAC1	1	1	1	1	1	1	3.50E+05	3.20E+05	1.90E+05	1.90E+05	1.8	1.7	1.0
RPLP1	1	1	1	1	1	1	2.70E+05	2.20E+05	1.30E+05	1.30E+05	2.1	1.7	1.0
CSTF3	1	1	1	1	1	1	5.70E+03	1.80E+04	1.10E+04	5.70E+03	1.0	3.2	1.9
GFAP	1	2	1	1	0	0	1.90E+06	2.10E+05	NF	2.10E+05	9.0	1.0	0.0
	1	1	1	1	1	1	1.30E+05	2.00E+05	1.20E+05	1.20E+05	1.1	1.7	1.0
AGMAT	1	1	1	2	0	0	4.60E+05	4.30E+05	NF	4.30E+05	1.1	1.0	0.0
RP2	1	1	1	1	1	1	4.00E+05	2.50E+05	1.90E+05	1.90E+05	2.1	1.3	1.0
WASHC5	1	1	1	1	1	1	1.10E+05	9.20E+04	5.40E+04	5.40E+04	2.0	1.7	1.0
BZW1	1	1	1	1	1	1	6.80E+05	4.20E+05	4.70E+05	4.20E+05	1.6	1.0	1.1
KLHL9	1	1	1	1	1	1	1.30E+05	8.00E+04	5.00E+04	5.00E+04	2.6	1.6	1.0
PRSS3	1	1	1	1	1	1	8.50E+05	8.30E+05	1.00E+06	8.30E+05	1.0	1.0	1.2
TMEM126B	1	1	1	1	1	1	1.10E+05	3.60E+04	1.40E+04	1.40E+04	7.9	2.6	1.0
NME6	1	1	1	1	1	1	2.60E+05	9.00E+04	8.90E+04	8.90E+04	2.9	1.0	1.0
GOLGA7	1	1	1	1	1	1	2.00E+05	1.80E+05	1.30E+05	1.30E+05	1.5	1.4	1.0
MRPL27	1	1	1	1	1	1	1.40E+06	1.00E+06	7.50E+05	7.50E+05	1.9	1.3	1.0
STON2	1	1	1	1	1	1	1.20E+05	1.40E+05	1.10E+05	1.10E+05	1.1	1.3	1.0
PRKCI	1	1	1	1	1	1	1.40E+05	9.60E+04	3.40E+05	9.60E+04	1.5	1.0	3.5
SCAMP4	1	1	1	1	1	1	1.40E+06	1.30E+06	8.90E+05	8.90E+05	1.6	1.5	1.0
DHCR24	1	1	1	1	1	1	1.40E+06	1.40E+06	1.00E+06	1.00E+06	1.4	1.4	1.0
CSNK1G2	1	1	1	1	1	1	2.30E+06	1.00E+06	3.70E+05	3.70E+05	6.2	2.7	1.0
TMEM109	0	0	1	1	1	2	NF	1.60E+06	2.50E+06	1.60E+06	0.0	1.0	1.6
ALG3	1	1	1	1	1	1	1.30E+05	8.00E+04	5.80E+04	5.80E+04	2.2	1.4	1.0
NDUFS5	1	1	1	1	1	1	6.80E+05	4.60E+05	3.10E+05	3.10E+05	2.2	1.5	1.0
UNC45A	1	1	1	1	1	1	1.10E+05	1.60E+05	1.30E+05	1.10E+05	1.0	1.5	1.2
FAU	1	1	1	1	1	1	2.00E+06	3.80E+06	1.80E+06	1.80E+06	1.1	2.1	1.0
ABCF2	1	1	1	1	1	1	4.90E+05	3.60E+05	2.10E+05	2.10E+05	2.3	1.7	1.0
PNKD	1	1	1	1	1	1	1.90E+05	2.00E+05	1.30E+05	1.30E+05	1.5	1.5	1.0
LRP1	1	2	1	1	0	0	1.80E+05	7.90E+04	NF	7.90E+04	2.3	1.0	0.0
TNPO2	0	0	1	1	1	2	NF	4.50E+05	4.60E+05	4.50E+05	0.0	1.0	1.0
RPS29	1	1	1	1	1	1	1.00E+06	5.50E+05	5.50E+05	5.50E+05	1.8	1.0	1.0

SLC39A1	1	1	1	2	0	0	3.50E+05	8.70E+05	NF	3.50E+05	1.0	2.5	0.0
LAMTOR3	1	1	1	1	1	1	1.10E+06	2.80E+05	3.30E+05	2.80E+05	3.9	1.0	1.2
BLMH	1	1	1	1	1	1	3.30E+05	2.70E+05	2.00E+05	2.00E+05	1.7	1.4	1.0
ATP5MK	1	1	1	1	1	1	4.00E+06	2.70E+06	2.30E+06	2.30E+06	1.7	1.2	1.0
SLC25A36	1	1	1	1	1	1	5.80E+05	4.40E+05	5.00E+05	4.40E+05	1.3	1.0	1.1
RCE1	1	1	1	1	1	1	5.50E+04	5.30E+04	3.30E+04	3.30E+04	1.7	1.6	1.0
B4GALT5	1	1	1	1	1	1	1.20E+06	3.70E+05	2.30E+05	2.30E+05	5.2	1.6	1.0
COQ6	1	1	1	1	1	1	2.10E+05	1.30E+05	1.00E+05	1.00E+05	2.1	1.3	1.0
CEPT1	1	1	1	1	1	1	4.50E+05	4.60E+05	2.60E+05	2.60E+05	1.7	1.8	1.0
MYDGF	1	1	1	1	1	1	6.40E+05	9.00E+05	1.00E+06	6.40E+05	1.0	1.4	1.6
AGRN	1	1	1	1	1	1	1.90E+05	1.20E+05	8.90E+04	8.90E+04	2.1	1.3	1.0
HACD2	1	1	1	1	1	1	7.50E+05	6.10E+05	4.20E+05	4.20E+05	1.8	1.5	1.0
TSPAN6	1	1	1	1	1	1	6.90E+05	6.50E+05	4.00E+05	4.00E+05	1.7	1.6	1.0
NDUFA4	1	1	1	1	1	1	4.00E+06	2.70E+06	2.30E+06	2.30E+06	1.7	1.2	1.0
COX5B	1	1	1	1	1	1	6.70E+05	6.90E+05	4.70E+05	4.70E+05	1.4	1.5	1.0
CCDC127	1	2	0	0	1	1	1.20E+06	NF	2.30E+05	2.30E+05	5.2	0.0	1.0
SEC61B	1	1	1	1	1	1	5.10E+06	2.60E+06	2.60E+06	2.60E+06	2.0	1.0	1.0
NRIP1	1	1	1	2	0	0	1.00E+06	3.80E+06	NF	1.00E+06	1.0	3.8	0.0
CNN3	0	0	1	3	0	0	NF	8.00E+06	NF	8.00E+06	0.0	1.0	0.0
MT-ND5	0	0	1	1	1	1	NF	6.70E+04	2.10E+04	2.10E+04	0.0	3.2	1.0
GDI2	0	0	1	1	1	1	NF	1.10E+05	9.30E+04	9.30E+04	0.0	1.2	1.0
VAMP3	0	0	1	1	1	1	NF	2.70E+05	2.00E+05	2.00E+05	0.0	1.4	1.0
FAM3C	0	0	1	1	1	1	NF	1.60E+05	1.60E+05	1.60E+05	0.0	1.0	1.0
H2AC20	0	0	1	2	0	0	NF	3.10E+04	NF	3.10E+04	0.0	1.0	0.0
CUL3	1	2	0	0	0	0	1.40E+05	NF	NF	1.40E+05	1.0	0.0	0.0
PLPP1	1	1	0	0	1	1	2.30E+05	NF	4.10E+04	4.10E+04	5.6	0.0	1.0
RRP12	1	1	1	1	0	0	6.90E+03	8.20E+03	NF	6.90E+03	1.0	1.2	0.0
EXOG	0	0	1	1	1	1	NF	2.40E+05	2.40E+05	2.40E+05	0.0	1.0	1.0
GBF1	1	1	1	1	0	0	6.50E+04	4.60E+04	NF	4.60E+04	1.4	1.0	0.0
SLC25A22	0	0	0	0	1	2	NF	NF	3.80E+05	3.80E+05	0.0	0.0	1.0
CHD3	1	1	1	1	0	0	3.20E+04	2.30E+04	NF	2.30E+04	1.4	1.0	0.0
GDI1	1	1	1	1	0	0	1.30E+05	2.90E+04	NF	2.90E+04	4.5	1.0	0.0
NXF1	1	1	1	1	0	0	1.80E+05	6.60E+04	NF	6.60E+04	2.7	1.0	0.0
HSPG2	0	0	1	1	1	1	NF	1.50E+05	1.00E+05	1.00E+05	0.0	1.5	1.0
UQCC3	0	0	1	1	1	1	NF	2.70E+05	2.10E+05	2.10E+05	0.0	1.3	1.0
CBARP	0	0	1	1	1	1	NF	4.90E+04	2.80E+04	2.80E+04	0.0	1.8	1.0
BUD23	0	0	1	1	1	1	NF	9.40E+04	8.00E+04	8.00E+04	0.0	1.2	1.0
DMAC1	1	2	0	0	0	0	1.40E+06	NF	NF	1.40E+06	1.0	0.0	0.0
EPHX1	1	1	0	0	1	1	2.40E+05	NF	6.80E+04	6.80E+04	3.5	0.0	1.0
NCEH1	1	1	0	0	1	1	3.30E+05	NF	8.10E+04	8.10E+04	4.1	0.0	1.0
PSPC1	1	1	0	0	1	1	5.10E+04	NF	5.40E+03	5.40E+03	9.4	0.0	1.0
NIFK	0	0	1	1	1	1	NF	6.50E+04	5.00E+04	5.00E+04	0.0	1.3	1.0
FOXRED2	0	0	1	1	1	1	NF	2.10E+04	1.30E+04	1.30E+04	0.0	1.6	1.0
SNAP91	0	0	1	1	1	1	NF	3.80E+05	1.70E+05	1.70E+05	0.0	2.2	1.0
DHRS7B	1	1	1	1	0	0	9.00E+04	9.10E+04	NF	9.00E+04	1.0	1.0	0.0
SLC25A40	1	1	0	0	1	1	1.80E+05	NF	1.80E+04	1.80E+04	10.0	0.0	1.0
PIGS	0	0	1	1	1	1	NF	1.00E+05	1.00E+05	1.00E+05	0.0	1.0	1.0
ERI1	1	1	1	1	0	0	1.90E+06	2.80E+05	NF	2.80E+05	6.8	1.0	0.0
TMEM256	0	0	1	1	1	1	NF	1.40E+05	3.40E+04	3.40E+04	0.0	4.1	1.0
PUF60	0	0	1	2	0	0	NF	3.10E+04	NF	3.10E+04	0.0	1.0	0.0
MRPS14	1	1	1	1	0	0	9.10E+05	2.40E+05	NF	2.40E+05	3.8	1.0	0.0

FPGS	1	1	1	1	0	0	8.60E+04	8.60E+03	NF	8.60E+03	10.0	1.0	0.0
U2AF2	0	0	1	1	1	1	NF	1.30E+05	7.60E+04	7.60E+04	0.0	1.7	1.0
SPPL2B	0	0	1	1	1	1	NF	7.40E+04	3.50E+04	3.50E+04	0.0	2.1	1.0
USP33	1	1	1	1	0	0	1.60E+05	8.60E+03	NF	8.60E+03	18.6	1.0	0.0
DDX24	0	0	1	2	0	0	NF	1.50E+05	NF	1.50E+05	0.0	1.0	0.0
CSDE1	0	0	1	1	1	1	NF	8.10E+04	4.80E+04	4.80E+04	0.0	1.7	1.0
CHMP6	0	0	1	1	1	1	NF	1.30E+06	3.00E+05	3.00E+05	0.0	4.3	1.0
CNN2	1	1	1	1	0	0	1.50E+05	7.10E+04	NF	7.10E+04	2.1	1.0	0.0
SLC25A17	0	0	1	1	1	1	NF	4.80E+04	1.10E+05	4.80E+04	0.0	1.0	2.3
VPS26A	0	0	1	1	1	1	NF	2.20E+05	2.90E+05	2.20E+05	0.0	1.0	1.3
GSTP1	0	0	1	1	1	1	NF	3.30E+05	1.20E+05	1.20E+05	0.0	2.8	1.0
BPNT2	1	1	0	0	1	1	3.70E+05	NF	9.50E+04	9.50E+04	3.9	0.0	1.0
WDFY1	0	0	1	1	1	1	NF	2.60E+05	3.40E+05	2.60E+05	0.0	1.0	1.3
ATP6V1E1	1	1	0	0	1	1	8.70E+04	NF	5.50E+04	5.50E+04	1.6	0.0	1.0
FAM210A	0	0	1	1	1	1	NF	2.30E+05	2.10E+05	2.10E+05	0.0	1.1	1.0
PON2	1	1	0	0	1	1	8.70E+05	NF	7.80E+05	7.80E+05	1.1	0.0	1.0
ERGIC2	1	1	1	1	0	0	2.00E+05	1.40E+04	NF	1.40E+04	14.3	1.0	0.0
MINPP1	1	1	0	0	1	1	3.40E+05	NF	1.80E+05	1.80E+05	1.9	0.0	1.0
TMEM9	1	1	1	1	0	0	3.00E+05	2.80E+05	NF	2.80E+05	1.1	1.0	0.0
FXR2	0	0	1	1	1	1	NF	3.30E+05	2.20E+05	2.20E+05	0.0	1.5	1.0
SIN3A	0	0	1	1	1	1	NF	4.90E+03	1.90E+03	1.90E+03	0.0	2.6	1.0
CDC37	1	1	1	1	0	0	3.40E+04	4.40E+04	NF	3.40E+04	1.0	1.3	0.0
TSR1	1	2	0	0	0	0	1.80E+05	NF	NF	1.80E+05	1.0	0.0	0.0
MRC2	0	0	1	1	1	1	NF	1.20E+05	1.00E+05	1.00E+05	0.0	1.2	1.0
DNAJC15	1	1	0	0	1	1	5.00E+05	NF	8.10E+04	8.10E+04	6.2	0.0	1.0
TSPAN3	1	1	0	0	1	1	7.00E+05	NF	2.90E+05	2.90E+05	2.4	0.0	1.0
SDF4	0	0	1	1	1	1	NF	2.60E+05	2.60E+05	2.60E+05	0.0	1.0	1.0
ADAM10	1	1	1	1	0	0	2.30E+05	2.40E+05	NF	2.30E+05	1.0	1.0	0.0
TNPO3	1	1	1	1	0	0	1.40E+04	2.20E+04	NF	1.40E+04	1.0	1.6	0.0
PSMC6	0	0	1	1	1	1	NF	1.60E+05	3.20E+04	3.20E+04	0.0	5.0	1.0
ANKLE2	0	0	1	2	0	0	NF	6.40E+05	NF	6.40E+05	0.0	1.0	0.0
TAGLN2	0	0	1	1	1	1	NF	1.50E+05	7.40E+04	7.40E+04	0.0	2.0	1.0
ERGIC3	0	0	1	1	1	1	NF	1.10E+05	4.30E+04	4.30E+04	0.0	2.6	1.0
MPV17L2	1	1	1	1	0	0	5.60E+05	4.10E+05	NF	4.10E+05	1.4	1.0	0.0
TRIP12	1	1	1	1	0	0	7.00E+04	5.30E+04	NF	5.30E+04	1.3	1.0	0.0
GDAP2	1	2	0	0	0	0	2.30E+05	NF	NF	2.30E+05	1.0	0.0	0.0
PSMB3	0	0	1	1	1	1	NF	1.10E+05	6.70E+04	6.70E+04	0.0	1.6	1.0
ALAS1	1	1	1	1	0	0	5.90E+05	4.30E+05	NF	4.30E+05	1.4	1.0	0.0
ACOX1	0	0	0	0	1	2	NF	NF	2.70E+04	2.70E+04	0.0	0.0	1.0
MGST1	1	1	1	1	0	0	4.60E+05	5.10E+05	NF	4.60E+05	1.0	1.1	0.0
SORT1	1	1	1	1	0	0	4.90E+05	7.70E+05	NF	4.90E+05	1.0	1.6	0.0
METTL15	1	1	1	1	0	0	3.80E+05	2.50E+05	NF	2.50E+05	1.5	1.0	0.0
SLC36A1	0	0	1	1	1	1	NF	1.20E+05	7.20E+04	7.20E+04	0.0	1.7	1.0
RETREG3	1	1	1	1	0	0	1.30E+05	6.60E+04	NF	6.60E+04	2.0	1.0	0.0
SUMO3	0	0	1	1	1	1	NF	1.40E+06	1.10E+06	1.10E+06	0.0	1.3	1.0
SLC27A4	1	2	0	0	0	0	5.60E+05	NF	NF	5.60E+05	1.0	0.0	0.0
GIPC1	0	0	1	1	1	1	NF	2.80E+05	1.50E+05	1.50E+05	0.0	1.9	1.0
CPNE3	0	0	1	1	1	1	NF	1.30E+05	6.00E+04	6.00E+04	0.0	2.2	1.0
FECH	0	0	1	1	1	1	NF	4.70E+05	4.10E+05	4.10E+05	0.0	1.1	1.0
THEM4	1	1	1	1	0	0	1.50E+05	1.40E+05	NF	1.40E+05	1.1	1.0	0.0
CBR4	1	1	0	0	1	1	5.70E+05	NF	3.30E+05	3.30E+05	1.7	0.0	1.0

ALDH9A1	0	0	1	1	1	1	NF	7.80E+05	4.30E+05	4.30E+05	0.0	1.8	1.0
FAM83H	1	2	0	0	0	0	2.30E+05	NF	NF	2.30E+05	1.0	0.0	0.0
SRSF7	0	0	1	1	1	1	NF	5.10E+05	5.30E+05	5.10E+05	0.0	1.0	1.0
CLN5	0	0	1	1	1	1	NF	5.20E+05	2.30E+05	2.30E+05	0.0	2.3	1.0
PRMT5	0	0	1	1	1	1	NF	1.50E+05	4.70E+04	4.70E+04	0.0	3.2	1.0
NUP98	1	1	0	0	1	1	1.20E+05	NF	3.50E+04	3.50E+04	3.4	0.0	1.0
CHID1	1	1	1	1	0	0	1.50E+05	1.00E+05	NF	1.00E+05	1.5	1.0	0.0
XAB2	0	0	1	1	1	1	NF	9.00E+04	6.00E+04	6.00E+04	0.0	1.5	1.0
MT-ND4	0	0	1	1	1	1	NF	1.10E+05	1.00E+05	1.00E+05	0.0	1.1	1.0
EFNB1	0	0	1	1	1	1	NF	2.00E+06	1.50E+05	1.50E+05	0.0	13.3	1.0
TM9SF4	0	0	1	1	1	1	NF	1.40E+05	1.10E+05	1.10E+05	0.0	1.3	1.0
EPN2	1	1	1	1	0	0	1.20E+05	7.00E+04	NF	7.00E+04	1.7	1.0	0.0
RALYL	0	0	1	1	1	1	NF	2.60E+05	2.00E+05	2.00E+05	0.0	1.3	1.0
TTC17	0	0	1	1	1	1	NF	7.40E+04	5.70E+04	5.70E+04	0.0	1.3	1.0
PDE3A	1	1	1	1	0	0	5.00E+05	3.60E+05	NF	3.60E+05	1.4	1.0	0.0
FUCA2	0	0	1	1	1	1	NF	2.30E+05	1.80E+05	1.80E+05	0.0	1.3	1.0
NDUFV2	0	0	1	1	1	1	NF	1.80E+05	1.50E+05	1.50E+05	0.0	1.2	1.0
TANC1	0	0	1	1	1	1	NF	9.60E+03	4.10E+03	4.10E+03	0.0	2.3	1.0
METTL17	0	0	0	0	1	2	NF	NF	1.10E+05	1.10E+05	0.0	0.0	1.0
COQ3	0	0	1	1	1	1	NF	1.50E+05	1.40E+05	1.40E+05	0.0	1.1	1.0
SLC7A11	0	0	1	2	0	0	NF	1.40E+05	NF	1.40E+05	0.0	1.0	0.0
CDSN	1	1	0	0	1	1	3.80E+05	NF	2.90E+05	2.90E+05	1.3	0.0	1.0
HYCC1	1	1	1	1	0	0	4.80E+04	8.50E+04	NF	4.80E+04	1.0	1.8	0.0
TTK	0	0	1	1	1	1	NF	2.60E+05	1.90E+05	1.90E+05	0.0	1.4	1.0
UQCC1	1	1	1	1	0	0	2.60E+05	1.20E+05	NF	1.20E+05	2.2	1.0	0.0
HACL1	0	0	1	1	1	1	NF	3.20E+05	2.70E+05	2.70E+05	0.0	1.2	1.0
COA3	0	0	1	1	1	1	NF	1.10E+06	6.70E+05	6.70E+05	0.0	1.6	1.0
DNASE2	0	0	1	1	1	1	NF	5.60E+05	2.90E+05	2.90E+05	0.0	1.9	1.0
IL13RA1	0	0	1	1	1	1	NF	5.30E+04	4.10E+04	4.10E+04	0.0	1.3	1.0
SLC12A4	1	1	1	1	0	0	4.50E+05	5.90E+05	NF	4.50E+05	1.0	1.3	0.0
STIM1	0	0	1	1	1	1	NF	1.40E+05	8.40E+04	8.40E+04	0.0	1.7	1.0
ALCAM	1	1	1	1	0	0	6.60E+05	3.60E+05	NF	3.60E+05	1.8	1.0	0.0
LYPLA1	1	1	0	0	1	1	5.90E+05	NF	3.90E+05	3.90E+05	1.5	0.0	1.0
MPDU1	0	0	1	1	1	1	NF	3.30E+05	1.50E+05	1.50E+05	0.0	2.2	1.0
MT-CYB	1	1	0	0	1	1	2.30E+05	NF	1.30E+05	1.30E+05	1.8	0.0	1.0
MAGED2	0	0	1	2	0	0	NF	3.50E+05	NF	3.50E+05	0.0	1.0	0.0
TRIP13	0	0	1	1	1	1	NF	2.20E+05	1.20E+05	1.20E+05	0.0	1.8	1.0
SCAP	0	0	1	1	1	1	NF	1.30E+04	1.40E+04	1.30E+04	0.0	1.0	1.1
DPY19L1	0	0	1	1	1	1	NF	9.00E+04	6.40E+04	6.40E+04	0.0	1.4	1.0
ITGB8	1	1	0	0	1	1	9.00E+04	NF	1.80E+04	1.80E+04	5.0	0.0	1.0
DEK	0	0	1	1	1	1	NF	1.90E+05	1.10E+05	1.10E+05	0.0	1.7	1.0
PEG10	0	0	1	1	1	1	NF	3.60E+05	2.90E+05	2.90E+05	0.0	1.2	1.0
COX6C	1	1	1	1	0	0	2.40E+06	1.50E+06	NF	1.50E+06	1.6	1.0	0.0
MRPL35	1	1	0	0	1	1	6.90E+05	NF	2.20E+05	2.20E+05	3.1	0.0	1.0
HAUS5	0	0	1	1	1	1	NF	6.20E+04	3.80E+04	3.80E+04	0.0	1.6	1.0
PBK	0	0	1	1	1	1	NF	9.30E+04	7.30E+04	7.30E+04	0.0	1.3	1.0
GET3	0	0	1	1	1	1	NF	3.20E+05	9.30E+04	9.30E+04	0.0	3.4	1.0
HBP1	1	1	1	1	0	0	9.40E+04	1.70E+04	NF	1.70E+04	5.5	1.0	0.0
PDZD8	0	0	1	1	1	1	NF	3.30E+04	3.70E+04	3.30E+04	0.0	1.0	1.1
LCP1	0	0	1	1	1	1	NF	7.60E+05	3.70E+05	3.70E+05	0.0	2.1	1.0
CLASP1	1	1	0	0	1	1	1.50E+05	NF	1.00E+05	1.00E+05	1.5	0.0	1.0

TRIM32	0	0	1	1	1	1	NF	1.00E+05	6.60E+04	6.60E+04	0.0	1.5	1.0
PTGES	0	0	1	1	1	1	NF	5.00E+05	3.40E+05	3.40E+05	0.0	1.5	1.0
ZDHHC13	0	0	1	1	1	1	NF	1.60E+05	1.30E+05	1.30E+05	0.0	1.2	1.0
EMC8	0	0	1	1	1	1	NF	2.20E+05	1.90E+05	1.90E+05	0.0	1.2	1.0
HNRNPDL	1	1	1	1	0	0	4.10E+05	5.70E+04	NF	5.70E+04	7.2	1.0	0.0
SCYL2	1	1	1	1	0	0	5.90E+04	6.60E+04	NF	5.90E+04	1.0	1.1	0.0
YWHAB	0	0	1	1	1	1	NF	5.90E+05	2.90E+05	2.90E+05	0.0	2.0	1.0
NDUFA11	0	0	1	1	1	1	NF	4.30E+05	3.00E+05	3.00E+05	0.0	1.4	1.0
SLC52A2	0	0	1	1	1	1	NF	3.30E+06	2.00E+06	2.00E+06	0.0	1.7	1.0
EEF1D	0	0	1	1	1	1	NF	4.50E+05	3.20E+05	3.20E+05	0.0	1.4	1.0
TOP1MT	0	0	1	1	1	1	NF	1.20E+05	7.00E+04	7.00E+04	0.0	1.7	1.0
STXBP5	0	0	1	1	1	1	NF	1.30E+05	1.20E+05	1.20E+05	0.0	1.1	1.0
NIN	0	0	0	0	1	2	NF	NF	1.40E+06	1.40E+06	0.0	0.0	1.0
NCAPG	0	0	1	2	0	0	NF	5.90E+06	NF	5.90E+06	0.0	1.0	0.0
SFXN4	0	0	1	1	1	1	NF	3.80E+05	2.20E+05	2.20E+05	0.0	1.7	1.0
ANKH	0	0	1	1	1	1	NF	7.50E+04	4.70E+03	4.70E+03	0.0	16.0	1.0
NFU1	0	0	1	1	1	1	NF	4.90E+04	2.80E+04	2.80E+04	0.0	1.8	1.0
CDH11	0	0	1	1	1	1	NF	1.90E+05	1.20E+05	1.20E+05	0.0	1.6	1.0
MRPL53	0	0	1	1	1	1	NF	1.30E+06	1.00E+06	1.00E+06	0.0	1.3	1.0
DNAJC30	1	1	1	1	0	0	7.10E+04	5.20E+04	NF	5.20E+04	1.4	1.0	0.0
UQCR10	1	1	1	1	0	0	2.60E+06	1.30E+06	NF	1.30E+06	2.0	1.0	0.0
SLITRK4	1	2	0	0	0	0	1.30E+05	NF	NF	1.30E+05	1.0	0.0	0.0
RTCB	0	0	1	1	1	1	NF	2.30E+05	1.20E+05	1.20E+05	0.0	1.9	1.0
PRKCE	1	1	1	1	0	0	5.90E+05	4.10E+05	NF	4.10E+05	1.4	1.0	0.0
MFF	1	1	1	1	0	0	4.60E+05	2.00E+05	NF	2.00E+05	2.3	1.0	0.0
PROCR	0	0	1	1	1	1	NF	3.50E+05	2.30E+05	2.30E+05	0.0	1.5	1.0
NDUFB1	0	0	0	0	1	2	NF	NF	1.10E+06	1.10E+06	0.0	0.0	1.0
CLIC1	0	0	1	2	0	0	NF	2.80E+05	NF	2.80E+05	0.0	1.0	0.0
RPL28	1	2	0	0	0	0	1.20E+06	NF	NF	1.20E+06	1.0	0.0	0.0
UBTD2	0	0	0	0	1	2	NF	NF	6.50E+06	6.50E+06	0.0	0.0	1.0
PLEC	0	0	1	1	0	0	NF	4.20E+04	NF	4.20E+04	0.0	1.0	0.0
SPG7	1	1	0	0	0	0	1.40E+05	NF	NF	1.40E+05	1.0	0.0	0.0
PREB	1	1	0	0	0	0	9.80E+03	NF	NF	9.80E+03	1.0	0.0	0.0
RCC1	1	1	0	0	0	0	1.30E+05	NF	NF	1.30E+05	1.0	0.0	0.0
RAB21	0	0	0	0	1	1	NF	NF	7.50E+04	7.50E+04	0.0	0.0	1.0
SLC25A32	1	1	0	0	0	0	4.60E+04	NF	NF	4.60E+04	1.0	0.0	0.0
QTRT1	0	0	0	0	1	1	NF	NF	2.80E+04	2.80E+04	0.0	0.0	1.0
CFL2	0	0	1	1	0	0	NF	1.10E+05	NF	1.10E+05	0.0	1.0	0.0
TTYH3	0	0	0	0	1	1	NF	NF	1.90E+04	1.90E+04	0.0	0.0	1.0
TIMM29	1	1	0	0	0	0	1.70E+05	NF	NF	1.70E+05	1.0	0.0	0.0
GCC2	0	0	0	0	1	1	NF	NF	2.30E+04	2.30E+04	0.0	0.0	1.0
TMX3	0	0	0	0	1	1	NF	NF	7.10E+04	7.10E+04	0.0	0.0	1.0
SAR1B	0	0	1	1	0	0	NF	1.80E+04	NF	1.80E+04	0.0	1.0	0.0
DDX23	0	0	1	1	0	0	NF	4.80E+05	NF	4.80E+05	0.0	1.0	0.0
MACF1	0	0	1	1	0	0	NF	5.30E+05	NF	5.30E+05	0.0	1.0	0.0
GAK	1	1	0	0	0	0	3.60E+03	NF	NF	3.60E+03	1.0	0.0	0.0
RAB6C	0	0	0	0	1	1	NF	NF	4.60E+04	4.60E+04	0.0	0.0	1.0
CTNNAL1	1	1	0	0	0	0	9.40E+04	NF	NF	9.40E+04	1.0	0.0	0.0
CUX1	0	0	0	0	1	1	NF	NF	2.20E+04	2.20E+04	0.0	0.0	1.0
AHSA1	1	1	0	0	0	0	1.90E+05	NF	NF	1.90E+05	1.0	0.0	0.0
PDP1	0	0	0	0	1	1	NF	NF	1.60E+05	1.60E+05	0.0	0.0	1.0

NID2	0	0	1	1	0	0	NF	9.90E+04	NF	9.90E+04	0.0	1.0	0.0
MTG1	1	1	0	0	0	0	4.40E+04	NF	NF	4.40E+04	1.0	0.0	0.0
P4HA3	1	1	0	0	0	0	7.70E+03	NF	NF	7.70E+03	1.0	0.0	0.0
RHOA	0	0	0	0	1	1	NF	NF	4.10E+04	4.10E+04	0.0	0.0	1.0
PPP2R2A	1	1	0	0	0	0	8.40E+03	NF	NF	8.40E+03	1.0	0.0	0.0
DDX49	1	1	0	0	0	0	3.40E+04	NF	NF	3.40E+04	1.0	0.0	0.0
COASY	1	1	0	0	0	0	7.70E+04	NF	NF	7.70E+04	1.0	0.0	0.0
HEBP1	1	1	0	0	0	0	8.90E+03	NF	NF	8.90E+03	1.0	0.0	0.0
MYO6	0	0	1	1	0	0	NF	1.60E+05	NF	1.60E+05	0.0	1.0	0.0
ATIC	0	0	1	1	0	0	NF	9.40E+03	NF	9.40E+03	0.0	1.0	0.0
OLA1	1	1	0	0	0	0	2.20E+04	NF	NF	2.20E+04	1.0	0.0	0.0
MMAB	1	1	0	0	0	0	4.00E+05	NF	NF	4.00E+05	1.0	0.0	0.0
MEST	1	1	0	0	0	0	6.80E+03	NF	NF	6.80E+03	1.0	0.0	0.0
AHCTF1	1	1	0	0	0	0	4.00E+04	NF	NF	4.00E+04	1.0	0.0	0.0
COX5A	1	1	0	0	0	0	3.40E+03	NF	NF	3.40E+03	1.0	0.0	0.0
PDK3	0	0	0	0	1	1	NF	NF	1.20E+05	1.20E+05	0.0	0.0	1.0
VTI1B	0	0	1	1	0	0	NF	6.80E+04	NF	6.80E+04	0.0	1.0	0.0
RAB5B	1	1	0	0	0	0	6.40E+04	NF	NF	6.40E+04	1.0	0.0	0.0
GOLT1B	1	1	0	0	0	0	2.50E+05	NF	NF	2.50E+05	1.0	0.0	0.0
VMP1	1	1	0	0	0	0	3.10E+04	NF	NF	3.10E+04	1.0	0.0	0.0
SIPA1L1	1	1	0	0	0	0	7.20E+04	NF	NF	7.20E+04	1.0	0.0	0.0
ENPP1	1	1	0	0	0	0	1.20E+05	NF	NF	1.20E+05	1.0	0.0	0.0
	0	0	0	0	1	1	NF	NF	2.20E+06	2.20E+06	0.0	0.0	1.0
KDELR1	1	1	0	0	0	0	1.60E+05	NF	NF	1.60E+05	1.0	0.0	0.0
TBC1D15	0	0	1	1	0	0	NF	3.20E+04	NF	3.20E+04	0.0	1.0	0.0
TRMT2B	1	1	0	0	0	0	7.00E+04	NF	NF	7.00E+04	1.0	0.0	0.0
DGUOK	0	0	0	0	1	1	NF	NF	5.50E+04	5.50E+04	0.0	0.0	1.0
MFSD5	0	0	0	0	1	1	NF	NF	4.00E+04	4.00E+04	0.0	0.0	1.0
ATP6V1D	0	0	1	1	0	0	NF	3.20E+05	NF	3.20E+05	0.0	1.0	0.0
TYRO3	0	0	0	0	1	1	NF	NF	7.10E+03	7.10E+03	0.0	0.0	1.0
SIL1	0	0	0	0	1	1	NF	NF	2.70E+05	2.70E+05	0.0	0.0	1.0
IPO4	1	1	0	0	0	0	1.20E+04	NF	NF	1.20E+04	1.0	0.0	0.0
LARP4B	0	0	1	1	0	0	NF	1.30E+05	NF	1.30E+05	0.0	1.0	0.0
PURA	1	1	0	0	0	0	1.60E+05	NF	NF	1.60E+05	1.0	0.0	0.0
SPTLC2	1	1	0	0	0	0	6.30E+04	NF	NF	6.30E+04	1.0	0.0	0.0
RALGAPB	1	1	0	0	0	0	8.80E+04	NF	NF	8.80E+04	1.0	0.0	0.0
MSI2	0	0	0	0	1	1	NF	NF	7.10E+04	7.10E+04	0.0	0.0	1.0
RFC1	0	0	1	1	0	0	NF	3.70E+04	NF	3.70E+04	0.0	1.0	0.0
ALDH4A1	1	1	0	0	0	0	6.10E+03	NF	NF	6.10E+03	1.0	0.0	0.0
HTT	0	0	1	1	0	0	NF	1.10E+04	NF	1.10E+04	0.0	1.0	0.0
RIDA	0	0	0	0	1	1	NF	NF	4.40E+05	4.40E+05	0.0	0.0	1.0
SRSF10	1	1	0	0	0	0	9.00E+04	NF	NF	9.00E+04	1.0	0.0	0.0
UPF2	0	0	1	1	0	0	NF	4.70E+04	NF	4.70E+04	0.0	1.0	0.0
JAK1	1	1	0	0	0	0	1.10E+04	NF	NF	1.10E+04	1.0	0.0	0.0
KYAT3	0	0	0	0	1	1	NF	NF	5.50E+04	5.50E+04	0.0	0.0	1.0
ACOX3	0	0	0	0	1	1	NF	NF	9.10E+04	9.10E+04	0.0	0.0	1.0
MFF	1	1	0	0	0	0	3.50E+04	NF	NF	3.50E+04	1.0	0.0	0.0
SIPA1L3	1	1	0	0	0	0	5.50E+03	NF	NF	5.50E+03	1.0	0.0	0.0
ESPL1	0	0	0	0	1	1	NF	NF	3.60E+04	3.60E+04	0.0	0.0	1.0
HTD2	1	1	0	0	0	0	4.10E+04	NF	NF	4.10E+04	1.0	0.0	0.0
RNPS1	0	0	1	1	0	0	NF	2.20E+05	NF	2.20E+05	0.0	1.0	0.0

ANTKMT	0	0	1	1	0	0	NF	2.20E+04	NF	2.20E+04	0.0	1.0	0.0
GPT2	1	1	0	0	0	0	4.10E+05	NF	NF	4.10E+05	1.0	0.0	0.0
CD81	1	1	0	0	0	0	7.90E+05	NF	NF	7.90E+05	1.0	0.0	0.0
RAP2B	0	0	1	1	0	0	NF	1.30E+05	NF	1.30E+05	0.0	1.0	0.0
TYSND1	1	1	0	0	0	0	7.50E+03	NF	NF	7.50E+03	1.0	0.0	0.0
EEF1B2	0	0	1	1	0	0	NF	9.60E+04	NF	9.60E+04	0.0	1.0	0.0
APOOL	0	0	0	0	1	1	NF	NF	1.80E+05	1.80E+05	0.0	0.0	1.0
PIGK	1	1	0	0	0	0	1.90E+05	NF	NF	1.90E+05	1.0	0.0	0.0
DNAJC5	0	0	1	1	0	0	NF	1.10E+05	NF	1.10E+05	0.0	1.0	0.0
GJA1	0	0	0	0	1	1	NF	NF	4.80E+04	4.80E+04	0.0	0.0	1.0
EEF1A2	0	0	0	0	1	1	NF	NF	7.80E+04	7.80E+04	0.0	0.0	1.0
STX6	0	0	1	1	0	0	NF	5.00E+05	NF	5.00E+05	0.0	1.0	0.0
VPS13D	1	1	0	0	0	0	8.90E+04	NF	NF	8.90E+04	1.0	0.0	0.0
DGKE	0	0	0	0	1	1	NF	NF	2.20E+04	2.20E+04	0.0	0.0	1.0
GNAT3	1	1	0	0	0	0	6.90E+06	NF	NF	6.90E+06	1.0	0.0	0.0
L1CAM	0	0	1	1	0	0	NF	4.60E+04	NF	4.60E+04	0.0	1.0	0.0
RDH14	0	0	1	1	0	0	NF	1.20E+05	NF	1.20E+05	0.0	1.0	0.0
MRPL32	0	0	0	0	1	1	NF	NF	7.70E+05	7.70E+05	0.0	0.0	1.0
LRRC7	1	1	0	0	0	0	1.00E+05	NF	NF	1.00E+05	1.0	0.0	0.0
LRRC8D	1	1	0	0	0	0	7.00E+03	NF	NF	7.00E+03	1.0	0.0	0.0
ARRDC1	1	1	0	0	0	0	1.40E+04	NF	NF	1.40E+04	1.0	0.0	0.0
MSH6	1	1	0	0	0	0	7.10E+03	NF	NF	7.10E+03	1.0	0.0	0.0
INTS3	1	1	0	0	0	0	9.50E+03	NF	NF	9.50E+03	1.0	0.0	0.0
SEC24B	1	1	0	0	0	0	5.00E+04	NF	NF	5.00E+04	1.0	0.0	0.0
AGPAT5	0	0	0	0	1	1	NF	NF	4.40E+05	4.40E+05	0.0	0.0	1.0
UQCR11	1	1	0	0	0	0	5.70E+04	NF	NF	5.70E+04	1.0	0.0	0.0
TMEM106B	1	1	0	0	0	0	4.50E+05	NF	NF	4.50E+05	1.0	0.0	0.0
ZNF326	1	1	0	0	0	0	1.40E+05	NF	NF	1.40E+05	1.0	0.0	0.0
PAICS	0	0	1	1	0	0	NF	6.90E+04	NF	6.90E+04	0.0	1.0	0.0
ZBTB14	0	0	0	0	1	1	NF	NF	1.50E+05	1.50E+05	0.0	0.0	1.0
TXNRD1	1	1	0	0	0	0	1.80E+05	NF	NF	1.80E+05	1.0	0.0	0.0
KIF22	0	0	1	1	0	0	NF	7.60E+04	NF	7.60E+04	0.0	1.0	0.0
MRPL16	1	1	0	0	0	0	8.30E+05	NF	NF	8.30E+05	1.0	0.0	0.0
MYORG	0	0	0	0	1	1	NF	NF	2.80E+04	2.80E+04	0.0	0.0	1.0
LMBRD1	0	0	1	1	0	0	NF	1.00E+06	NF	1.00E+06	0.0	1.0	0.0
NUP214	0	0	1	1	0	0	NF	1.40E+05	NF	1.40E+05	0.0	1.0	0.0
USP7	1	1	0	0	0	0	1.80E+04	NF	NF	1.80E+04	1.0	0.0	0.0
LIG3	0	0	0	0	1	1	NF	NF	1.30E+05	1.30E+05	0.0	0.0	1.0
NDUFS4	0	0	1	1	0	0	NF	2.60E+05	NF	2.60E+05	0.0	1.0	0.0
DDX20	0	0	1	1	0	0	NF	7.70E+04	NF	7.70E+04	0.0	1.0	0.0
TRIO	0	0	1	1	0	0	NF	7.50E+04	NF	7.50E+04	0.0	1.0	0.0
TMOD3	0	0	1	1	0	0	NF	4.70E+04	NF	4.70E+04	0.0	1.0	0.0
AP2M1	0	0	0	0	1	1	NF	NF	2.20E+05	2.20E+05	0.0	0.0	1.0
CNNM4	0	0	0	0	1	1	NF	NF	4.20E+04	4.20E+04	0.0	0.0	1.0
SUGP2	0	0	1	1	0	0	NF	8.30E+03	NF	8.30E+03	0.0	1.0	0.0
MDC1	0	0	1	1	0	0	NF	1.40E+05	NF	1.40E+05	0.0	1.0	0.0
SNRPA1	0	0	1	1	0	0	NF	1.50E+04	NF	1.50E+04	0.0	1.0	0.0
SMARCA1	0	0	1	1	0	0	NF	2.50E+05	NF	2.50E+05	0.0	1.0	0.0
CAV2	0	0	1	1	0	0	NF	1.80E+06	NF	1.80E+06	0.0	1.0	0.0
COIL	0	0	0	0	1	1	NF	NF	2.50E+04	2.50E+04	0.0	0.0	1.0
AMIGO2	0	0	1	1	0	0	NF	5.30E+04	NF	5.30E+04	0.0	1.0	0.0

CLDN12	0	0	0	0	1	1	NF	NF	3.30E+04	3.30E+04	0.0	0.0	1.0
DNAAF5	0	0	1	1	0	0	NF	5.80E+04	NF	5.80E+04	0.0	1.0	0.0
PURB	0	0	0	0	1	1	NF	NF	1.80E+06	1.80E+06	0.0	0.0	1.0
MPG	0	0	0	0	1	1	NF	NF	5.40E+04	5.40E+04	0.0	0.0	1.0
FANCI	0	0	1	1	0	0	NF	6.90E+03	NF	6.90E+03	0.0	1.0	0.0
TUBB6	0	0	1	1	0	0	NF	7.10E+04	NF	7.10E+04	0.0	1.0	0.0
ITSN2	0	0	0	0	1	1	NF	NF	6.00E+03	6.00E+03	0.0	0.0	1.0
PLA2G15	0	0	0	0	1	1	NF	NF	1.10E+05	1.10E+05	0.0	0.0	1.0
WARS1	0	0	1	1	0	0	NF	8.00E+03	NF	8.00E+03	0.0	1.0	0.0
SLC19A1	0	0	1	1	0	0	NF	8.30E+04	NF	8.30E+04	0.0	1.0	0.0
PWP2	0	0	0	0	1	1	NF	NF	1.40E+04	1.40E+04	0.0	0.0	1.0
PHIP	0	0	1	1	0	0	NF	6.70E+04	NF	6.70E+04	0.0	1.0	0.0
RPS27L	1	1	0	0	0	0	5.50E+05	NF	NF	5.50E+05	1.0	0.0	0.0
EPHA3	0	0	0	0	1	1	NF	NF	1.80E+05	1.80E+05	0.0	0.0	1.0
SRRT	0	0	0	0	1	1	NF	NF	3.30E+04	3.30E+04	0.0	0.0	1.0
EIF2D	0	0	1	1	0	0	NF	5.20E+04	NF	5.20E+04	0.0	1.0	0.0
CSNK1G3	1	1	0	0	0	0	1.90E+05	NF	NF	1.90E+05	1.0	0.0	0.0
TNFAIP2	1	1	0	0	0	0	1.20E+04	NF	NF	1.20E+04	1.0	0.0	0.0
GDAP1	0	0	0	0	1	1	NF	NF	4.00E+05	4.00E+05	0.0	0.0	1.0
B3GALT6	1	1	0	0	0	0	5.20E+05	NF	NF	5.20E+05	1.0	0.0	0.0
NSUN3	1	1	0	0	0	0	2.00E+04	NF	NF	2.00E+04	1.0	0.0	0.0
CTSL	0	0	0	0	1	1	NF	NF	1.40E+05	1.40E+05	0.0	0.0	1.0
DERL1	0	0	0	0	1	1	NF	NF	1.80E+05	1.80E+05	0.0	0.0	1.0
AARS1	0	0	1	1	0	0	NF	3.90E+04	NF	3.90E+04	0.0	1.0	0.0
TMED5	1	1	0	0	0	0	1.10E+05	NF	NF	1.10E+05	1.0	0.0	0.0
TUBA4A	1	1	0	0	0	0	1.50E+05	NF	NF	1.50E+05	1.0	0.0	0.0
GSTZ1	0	0	0	0	1	1	NF	NF	5.80E+04	5.80E+04	0.0	0.0	1.0
RNMT	1	1	0	0	0	0	4.50E+04	NF	NF	4.50E+04	1.0	0.0	0.0
DOLPP1	1	1	0	0	0	0	2.10E+04	NF	NF	2.10E+04	1.0	0.0	0.0
QRSL1	1	1	0	0	0	0	2.00E+05	NF	NF	2.00E+05	1.0	0.0	0.0
NUP54	0	0	1	1	0	0	NF	1.20E+05	NF	1.20E+05	0.0	1.0	0.0
RHOBTB3	0	0	1	1	0	0	NF	3.60E+05	NF	3.60E+05	0.0	1.0	0.0
PSMD5	0	0	1	1	0	0	NF	1.90E+04	NF	1.90E+04	0.0	1.0	0.0
CYP20A1	1	1	0	0	0	0	3.60E+05	NF	NF	3.60E+05	1.0	0.0	0.0
UTP20	0	0	1	1	0	0	NF	6.90E+04	NF	6.90E+04	0.0	1.0	0.0
BMP2K	0	0	0	0	1	1	NF	NF	1.30E+05	1.30E+05	0.0	0.0	1.0
VKORC1L1	1	1	0	0	0	0	1.90E+05	NF	NF	1.90E+05	1.0	0.0	0.0
SLC36A4	0	0	0	0	1	1	NF	NF	5.20E+04	5.20E+04	0.0	0.0	1.0
NDUFB6	0	0	1	1	0	0	NF	1.70E+05	NF	1.70E+05	0.0	1.0	0.0
MKI67	0	0	1	1	0	0	NF	1.80E+05	NF	1.80E+05	0.0	1.0	0.0
PRKAA1	0	0	1	1	0	0	NF	3.70E+05	NF	3.70E+05	0.0	1.0	0.0
SRSF4	0	0	1	1	0	0	NF	1.50E+06	NF	1.50E+06	0.0	1.0	0.0
NF1	0	0	1	1	0	0	NF	8.20E+04	NF	8.20E+04	0.0	1.0	0.0
TOLLIP	1	1	0	0	0	0	1.10E+05	NF	NF	1.10E+05	1.0	0.0	0.0
RRAS	0	0	1	1	0	0	NF	2.90E+05	NF	2.90E+05	0.0	1.0	0.0
GCC1	0	0	0	0	1	1	NF	NF	1.70E+05	1.70E+05	0.0	0.0	1.0
STUB1	0	0	1	1	0	0	NF	2.30E+05	NF	2.30E+05	0.0	1.0	0.0
NIPBL	0	0	1	1	0	0	NF	7.20E+04	NF	7.20E+04	0.0	1.0	0.0
PCNX3	0	0	0	0	1	1	NF	NF	9.40E+04	9.40E+04	0.0	0.0	1.0
RMDN1	0	0	0	0	1	1	NF	NF	1.00E+06	1.00E+06	0.0	0.0	1.0
RAD21	0	0	1	1	0	0	NF	1.10E+05	NF	1.10E+05	0.0	1.0	0.0

EXOC4	0	0	1	1	0	0	NF	1.00E+05	NF	1.00E+05	0.0	1.0	0.0
API5	0	0	1	1	0	0	NF	3.60E+03	NF	3.60E+03	0.0	1.0	0.0
LARP1B	0	0	1	1	0	0	NF	2.10E+05	NF	2.10E+05	0.0	1.0	0.0
WDR36	1	1	0	0	0	0	1.40E+04	NF	NF	1.40E+04	1.0	0.0	0.0
MYO1F	1	1	0	0	0	0	9.20E+04	NF	NF	9.20E+04	1.0	0.0	0.0
POGLUT1	0	0	0	0	1	1	NF	NF	2.00E+05	2.00E+05	0.0	0.0	1.0
CPM	0	0	0	0	1	1	NF	NF	3.00E+05	3.00E+05	0.0	0.0	1.0
GOLGA4	0	0	0	0	1	1	NF	NF	1.80E+05	1.80E+05	0.0	0.0	1.0
PSMB5	0	0	1	1	0	0	NF	3.70E+05	NF	3.70E+05	0.0	1.0	0.0
ANAPC1	1	1	0	0	0	0	6.70E+03	NF	NF	6.70E+03	1.0	0.0	0.0
TPD52L2	0	0	1	1	0	0	NF	2.60E+05	NF	2.60E+05	0.0	1.0	0.0
SEC24A	0	0	0	0	1	1	NF	NF	3.20E+04	3.20E+04	0.0	0.0	1.0
MT-ND2	1	1	0	0	0	0	3.30E+05	NF	NF	3.30E+05	1.0	0.0	0.0
PRSS21	0	0	0	0	1	1	NF	NF	2.60E+05	2.60E+05	0.0	0.0	1.0
EBP	0	0	0	0	1	1	NF	NF	8.50E+05	8.50E+05	0.0	0.0	1.0
HP1BP3	1	1	0	0	0	0	3.00E+06	NF	NF	3.00E+06	1.0	0.0	0.0
RAD50	0	0	1	1	0	0	NF	1.10E+05	NF	1.10E+05	0.0	1.0	0.0
DNER	1	1	0	0	0	0	5.80E+05	NF	NF	5.80E+05	1.0	0.0	0.0
HDAC1	0	0	1	1	0	0	NF	2.40E+05	NF	2.40E+05	0.0	1.0	0.0
ABHD11	0	0	0	0	1	1	NF	NF	2.60E+05	2.60E+05	0.0	0.0	1.0
TBC1D9B	0	0	0	0	1	1	NF	NF	4.00E+03	4.00E+03	0.0	0.0	1.0
MYBL1	0	0	1	1	0	0	NF	5.80E+05	NF	5.80E+05	0.0	1.0	0.0
CCNY	0	0	1	1	0	0	NF	7.50E+04	NF	7.50E+04	0.0	1.0	0.0
UBAC2	1	1	0	0	0	0	4.90E+04	NF	NF	4.90E+04	1.0	0.0	0.0
CORO2A	0	0	1	1	0	0	NF	3.40E+04	NF	3.40E+04	0.0	1.0	0.0
HYDIN	0	0	0	0	1	1	NF	NF	1.60E+05	1.60E+05	0.0	0.0	1.0
AP2S1	1	1	0	0	0	0	9.20E+05	NF	NF	9.20E+05	1.0	0.0	0.0
NDUFS8	1	1	0	0	0	0	2.00E+05	NF	NF	2.00E+05	1.0	0.0	0.0
PIGU	0	0	0	0	1	1	NF	NF	3.10E+05	3.10E+05	0.0	0.0	1.0
ASCC3	0	0	0	0	1	1	NF	NF	2.00E+05	2.00E+05	0.0	0.0	1.0
ABCA2	0	0	0	0	1	1	NF	NF	1.60E+05	1.60E+05	0.0	0.0	1.0
SNX17	0	0	1	1	0	0	NF	3.70E+04	NF	3.70E+04	0.0	1.0	0.0
ARSB	0	0	0	0	1	1	NF	NF	1.60E+05	1.60E+05	0.0	0.0	1.0
PSMA4	0	0	0	0	1	1	NF	NF	8.90E+04	8.90E+04	0.0	0.0	1.0
GGT1	0	0	1	1	0	0	NF	3.00E+05	NF	3.00E+05	0.0	1.0	0.0
SMC3	0	0	0	0	1	1	NF	NF	1.10E+05	1.10E+05	0.0	0.0	1.0
VPS11	0	0	1	1	0	0	NF	1.10E+05	NF	1.10E+05	0.0	1.0	0.0
SLC4A11	1	1	0	0	0	0	5.60E+04	NF	NF	5.60E+04	1.0	0.0	0.0
ROR2	0	0	0	0	1	1	NF	NF	7.60E+04	7.60E+04	0.0	0.0	1.0
GNAQ	0	0	1	1	0	0	NF	2.30E+05	NF	2.30E+05	0.0	1.0	0.0
WWP2	0	0	1	1	0	0	NF	2.40E+05	NF	2.40E+05	0.0	1.0	0.0
PMS2P11	1	1	0	0	0	0	1.10E+05	NF	NF	1.10E+05	1.0	0.0	0.0
TMEM41A	1	1	0	0	0	0	5.10E+04	NF	NF	5.10E+04	1.0	0.0	0.0
NT5DC3	0	0	1	1	0	0	NF	8.00E+04	NF	8.00E+04	0.0	1.0	0.0
COL3A1	0	0	0	0	1	1	NF	NF	1.90E+05	1.90E+05	0.0	0.0	1.0
BCAM	0	0	1	1	0	0	NF	1.40E+05	NF	1.40E+05	0.0	1.0	0.0
INCENP	0	0	1	1	0	0	NF	1.00E+05	NF	1.00E+05	0.0	1.0	0.0
EIF5	1	1	0	0	0	0	6.20E+04	NF	NF	6.20E+04	1.0	0.0	0.0
SLC23A2	1	1	0	0	0	0	5.60E+04	NF	NF	5.60E+04	1.0	0.0	0.0
ZC3H7B	0	0	1	1	0	0	NF	8.00E+04	NF	8.00E+04	0.0	1.0	0.0
MT-ND6	0	0	0	0	1	1	NF	NF	4.80E+05	4.80E+05	0.0	0.0	1.0

TFCP2	0	0	0	0	1	1	NF	NF	7.10E+04	7.10E+04	0.0	0.0	1.0
FBL	0	0	1	1	0	0	NF	7.90E+05	NF	7.90E+05	0.0	1.0	0.0
FARP1	1	1	0	0	0	0	2.10E+05	NF	NF	2.10E+05	1.0	0.0	0.0
TNFRSF10B	0	0	1	1	0	0	NF	6.00E+05	NF	6.00E+05	0.0	1.0	0.0
ALG5	0	0	1	1	0	0	NF	3.30E+05	NF	3.30E+05	0.0	1.0	0.0
PPP2R5D	0	0	1	1	0	0	NF	3.10E+04	NF	3.10E+04	0.0	1.0	0.0
SLC22A4	1	1	0	0	0	0	1.20E+05	NF	NF	1.20E+05	1.0	0.0	0.0
NELFCD	1	1	0	0	0	0	5.40E+04	NF	NF	5.40E+04	1.0	0.0	0.0
SVIP	0	0	0	0	1	1	NF	NF	2.40E+05	2.40E+05	0.0	0.0	1.0
MTMR4	0	0	1	1	0	0	NF	4.90E+05	NF	4.90E+05	0.0	1.0	0.0
PFKP	0	0	1	1	0	0	NF	1.30E+05	NF	1.30E+05	0.0	1.0	0.0
TRPM7	0	0	1	1	0	0	NF	5.80E+04	NF	5.80E+04	0.0	1.0	0.0
STX16	1	1	0	0	0	0	1.70E+05	NF	NF	1.70E+05	1.0	0.0	0.0
MBOAT7	1	1	0	0	0	0	3.70E+05	NF	NF	3.70E+05	1.0	0.0	0.0
ICAM1	0	0	0	0	1	1	NF	NF	6.70E+04	6.70E+04	0.0	0.0	1.0
PI4K2B	0	0	1	1	0	0	NF	1.40E+05	NF	1.40E+05	0.0	1.0	0.0
SLC26A6	0	0	1	1	0	0	NF	9.20E+04	NF	9.20E+04	0.0	1.0	0.0
OSBPL10	0	0	1	1	0	0	NF	1.00E+05	NF	1.00E+05	0.0	1.0	0.0
DIP2B	0	0	0	0	1	1	NF	NF	3.50E+04	3.50E+04	0.0	0.0	1.0
SLC9A1	0	0	1	1	0	0	NF	1.20E+05	NF	1.20E+05	0.0	1.0	0.0
GNA14	0	0	0	0	1	1	NF	NF	7.50E+05	7.50E+05	0.0	0.0	1.0
MAP3K1	1	1	0	0	0	0	5.30E+04	NF	NF	5.30E+04	1.0	0.0	0.0
PTPRD	0	0	0	0	1	1	NF	NF	5.80E+04	5.80E+04	0.0	0.0	1.0
YIF1A	1	1	0	0	0	0	6.40E+06	NF	NF	6.40E+06	1.0	0.0	0.0
SLC46A1	1	1	0	0	0	0	4.00E+05	NF	NF	4.00E+05	1.0	0.0	0.0
ATP6V1B1	1	1	0	0	0	0	3.30E+05	NF	NF	3.30E+05	1.0	0.0	0.0
NUP133	1	1	0	0	0	0	1.90E+05	NF	NF	1.90E+05	1.0	0.0	0.0
PDCD4	1	1	0	0	0	0	1.40E+06	NF	NF	1.40E+06	1.0	0.0	0.0
RPL7L1	1	1	0	0	0	0	7.60E+04	NF	NF	7.60E+04	1.0	0.0	0.0
ATL2	0	0	0	0	1	1	NF	NF	2.50E+04	2.50E+04	0.0	0.0	1.0
NAT2	0	0	1	1	0	0	NF	3.10E+05	NF	3.10E+05	0.0	1.0	0.0
CELSR3	0	0	1	1	0	0	NF	6.10E+04	NF	6.10E+04	0.0	1.0	0.0
SUV39H2	0	0	0	0	1	1	NF	NF	5.80E+05	5.80E+05	0.0	0.0	1.0
TMED7	0	0	1	1	0	0	NF	2.70E+06	NF	2.70E+06	0.0	1.0	0.0
RPTOR	1	1	0	0	0	0	5.70E+04	NF	NF	5.70E+04	1.0	0.0	0.0
FXR1	1	1	0	0	0	0	1.30E+05	NF	NF	1.30E+05	1.0	0.0	0.0
PPFIBP1	0	0	1	1	0	0	NF	3.10E+05	NF	3.10E+05	0.0	1.0	0.0
PTGR3	1	1	0	0	0	0	1.10E+05	NF	NF	1.10E+05	1.0	0.0	0.0
LYN	0	0	1	1	0	0	NF	3.00E+05	NF	3.00E+05	0.0	1.0	0.0
VPS8	0	0	1	1	0	0	NF	2.70E+06	NF	2.70E+06	0.0	1.0	0.0
CAPN7	0	0	1	1	0	0	NF	8.50E+05	NF	8.50E+05	0.0	1.0	0.0
ATP6V1G1	0	0	0	0	1	1	NF	NF	4.50E+05	4.50E+05	0.0	0.0	1.0
NSUN4	0	0	0	0	1	1	NF	NF	2.00E+05	2.00E+05	0.0	0.0	1.0
JAGN1	0	0	1	1	0	0	NF	3.50E+05	NF	3.50E+05	0.0	1.0	0.0
XPOT	0	0	0	0	1	1	NF	NF	4.40E+04	4.40E+04	0.0	0.0	1.0
NOP58	1	1	0	0	0	0	2.60E+05	NF	NF	2.60E+05	1.0	0.0	0.0
GK	0	0	0	0	1	1	NF	NF	2.00E+05	2.00E+05	0.0	0.0	1.0
SEC62	1	1	0	0	0	0	6.90E+06	NF	NF	6.90E+06	1.0	0.0	0.0
NUMBL	1	1	0	0	0	0	3.20E+05	NF	NF	3.20E+05	1.0	0.0	0.0
FADS1	1	1	0	0	0	0	4.90E+05	NF	NF	4.90E+05	1.0	0.0	0.0
PRRC2B	0	0	1	1	0	0	NF	5.90E+04	NF	5.90E+04	0.0	1.0	0.0

ZNF395	1	1	0	0	0	0	4.80E+05	NF	NF	4.80E+05	1.0	0.0	0.0
PCNT	1	1	0	0	0	0	7.10E+05	NF	NF	7.10E+05	1.0	0.0	0.0
COMTD1	1	1	0	0	0	0	2.00E+05	NF	NF	2.00E+05	1.0	0.0	0.0
	1	1	0	0	0	0	5.40E+05	NF	NF	5.40E+05	1.0	0.0	0.0
MIX23	0	0	1	1	0	0	NF	5.50E+05	NF	5.50E+05	0.0	1.0	0.0
POLG2	0	0	0	0	1	1	NF	NF	1.70E+05	1.70E+05	0.0	0.0	1.0
LUC7L2	0	0	0	0	1	1	NF	NF	7.30E+04	7.30E+04	0.0	0.0	1.0
MED16	1	1	0	0	0	0	1.50E+05	NF	NF	1.50E+05	1.0	0.0	0.0
GPAA1	0	0	1	1	0	0	NF	2.20E+05	NF	2.20E+05	0.0	1.0	0.0
HGSNAT	0	0	0	0	1	1	NF	NF	2.40E+05	2.40E+05	0.0	0.0	1.0
VPS39	0	0	1	1	0	0	NF	1.10E+05	NF	1.10E+05	0.0	1.0	0.0
MRPL34	1	1	0	0	0	0	3.20E+05	NF	NF	3.20E+05	1.0	0.0	0.0
EI24	1	1	0	0	0	0	2.80E+05	NF	NF	2.80E+05	1.0	0.0	0.0
TCF3	0	0	0	0	1	1	NF	NF	1.40E+05	1.40E+05	0.0	0.0	1.0
FRMD4B	1	1	0	0	0	0	6.10E+05	NF	NF	6.10E+05	1.0	0.0	0.0
CDK14	0	0	1	1	0	0	NF	1.40E+06	NF	1.40E+06	0.0	1.0	0.0
SEPTIN9	0	0	1	1	0	0	NF	1.40E+05	NF	1.40E+05	0.0	1.0	0.0
ARMC8	0	0	0	0	1	1	NF	NF	6.30E+04	6.30E+04	0.0	0.0	1.0
MRPS33	0	0	0	0	1	1	NF	NF	2.60E+05	2.60E+05	0.0	0.0	1.0
MFSD1	0	0	1	1	0	0	NF	1.50E+06	NF	1.50E+06	0.0	1.0	0.0
FAM120C	0	0	1	1	0	0	NF	6.10E+04	NF	6.10E+04	0.0	1.0	0.0
ADCK1	0	0	1	1	0	0	NF	8.00E+04	NF	8.00E+04	0.0	1.0	0.0
BZW2	1	1	0	0	0	0	8.00E+04	NF	NF	8.00E+04	1.0	0.0	0.0
CREBZF	1	1	0	0	0	0	1.10E+06	NF	NF	1.10E+06	1.0	0.0	0.0
HSPA14	0	0	1	1	0	0	NF	6.00E+04	NF	6.00E+04	0.0	1.0	0.0
LDLR	0	0	1	1	0	0	NF	1.50E+06	NF	1.50E+06	0.0	1.0	0.0
G6PD	0	0	1	1	0	0	NF	7.60E+04	NF	7.60E+04	0.0	1.0	0.0
NOC3L	0	0	0	0	1	1	NF	NF	1.90E+04	1.90E+04	0.0	0.0	1.0
HEY1	0	0	0	0	1	1	NF	NF	2.10E+05	2.10E+05	0.0	0.0	1.0
TPST1	0	0	1	1	0	0	NF	6.30E+04	NF	6.30E+04	0.0	1.0	0.0
RAB38	1	1	0	0	0	0	2.60E+05	NF	NF	2.60E+05	1.0	0.0	0.0
HS2ST1	1	1	0	0	0	0	1.40E+05	NF	NF	1.40E+05	1.0	0.0	0.0
ZNF487	0	0	0	0	1	1	NF	NF	4.60E+05	4.60E+05	0.0	0.0	1.0
PPOX	1	1	0	0	0	0	7.80E+05	NF	NF	7.80E+05	1.0	0.0	0.0
ENDOG	0	0	0	0	1	1	NF	NF	2.10E+05	2.10E+05	0.0	0.0	1.0
DHX57	1	1	0	0	0	0	1.20E+05	NF	NF	1.20E+05	1.0	0.0	0.0
NEU1	0	0	1	1	0	0	NF	3.50E+05	NF	3.50E+05	0.0	1.0	0.0
DDX27	0	0	1	1	0	0	NF	1.00E+05	NF	1.00E+05	0.0	1.0	0.0
POLR3A	0	0	0	0	1	1	NF	NF	3.20E+04	3.20E+04	0.0	0.0	1.0
ABHD12	1	1	0	0	0	0	6.20E+04	NF	NF	6.20E+04	1.0	0.0	0.0
LOXL3	1	1	0	0	0	0	9.40E+05	NF	NF	9.40E+05	1.0	0.0	0.0
MZB1	0	0	1	1	0	0	NF	5.90E+05	NF	5.90E+05	0.0	1.0	0.0
VPS51	0	0	1	1	0	0	NF	2.50E+04	NF	2.50E+04	0.0	1.0	0.0
CXCL14	1	1	0	0	0	0	1.00E+06	NF	NF	1.00E+06	1.0	0.0	0.0
MGST2	1	1	0	0	0	0	6.50E+05	NF	NF	6.50E+05	1.0	0.0	0.0
AK4	0	0	1	1	0	0	NF	4.50E+05	NF	4.50E+05	0.0	1.0	0.0
THOP1	0	0	1	1	0	0	NF	3.80E+05	NF	3.80E+05	0.0	1.0	0.0
RAC2	0	0	1	1	0	0	NF	1.90E+06	NF	1.90E+06	0.0	1.0	0.0
PAFAH1B1	0	0	1	1	0	0	NF	4.30E+05	NF	4.30E+05	0.0	1.0	0.0
DDR2	1	1	0	0	0	0	1.20E+06	NF	NF	1.20E+06	1.0	0.0	0.0
TMEM115	1	1	0	0	0	0	1.80E+05	NF	NF	1.80E+05	1.0	0.0	0.0
