# Supplementary material for: SARS-CoV-2 ORF3a blocks lysosomal cholesterol egress by disrupting VPS39-regulated NPC2 trafficking and BMP metabolism
Source: Cell Rep. Author manuscript; Available in PMC 2026 Jul 17. (PMC13378056; doi:10.1016/j.celrep.2026.117544)
Supplement: 3 [file NIHMS2190776-supplement-3.pdf]

Only in W193 lysosomes		
**Gene Symbol**	**MWT (kDa)**	**Sum Intensity W193A-lyso-IP**
UBTD2	26.17	6500000
RPL23	14.86	5200000
KIF5C	109.43	4600000
MSMO1	35.19	2700000
PCCA	80.01	2600000
	18.82	2200000
NDUFA10	40.72	1800000
PURB	33.22	1800000
CTSA	54.43	1700000
NIN	243.1	1400000
ATAD1	40.72	1200000
ISCA1	14.17	1200000
HIBCH	43.45	1100000
IVD	46.62	1100000
NDUFB1	6.96	1100000
MRPL41	15.37	1000000
RMDN1	35.79	1000000
TST	33.41	990000
TCIRG1	92.91	970000
BCAT2	44.26	960000
A2M	163.19	950000
PIGT	65.66	890000
SLC27A2	70.27	880000
EBP	26.34	850000
MKKS	7.26	840000
PON2	39.36	780000
MRPL32	21.39	770000
GNA14	41.54	750000
NDUFA2	10.91	730000
HLA-C	40.62	620000
SPNS1	56.59	610000
UGGT2	174.62	590000
MT-ND1	35.64	580000
SUV39H2	4.81	580000
RAB34	29.03	550000
MT-ND6	18.61	480000
ZNF487	51.59	460000
ATP6V1G1	13.75	450000
LYRM7	11.95	440000
RIDA	14.48	440000

Only in ORF3a lysosomes		
**Gene Symbol**	**MWT (kDa)**	**Sum Intensity ORF3a-lyso-IP**
API5	58.97	3600
FANCI	149.23	6900
WARS1	53.13	8000
RRP12	143.61	8200
SUGP2	120.13	8300
FPGS	64.57	8600
USP33	106.66	8600
ATIC	64.58	9400
HTT	347.38	11000
ERGIC2	42.52	14000
SNRPA1	28.4	15000
VPS53	94.35	16000
HBP1	57.61	17000
SAR1B	22.4	18000
PSMD5	56.16	19000
ATPAF2	32.75	20000
TNPO3	104.14	22000
ANTKMT	25.11	22000
CHD3	226.45	23000
IDI1	26.3	24000
VPS51	85.99	25000
GDI1	50.55	29000
H2AC20	13.98	31000
PUF60	59.84	31000
PPP2R5D	69.95	31000
TBC1D15	32.12	32000
CORO2A	59.73	34000
RFC1	128.18	37000
SNX17	52.87	37000
AARS1	106.74	39000
PLEC	387.8	42000
CDC37	44.44	44000
RAB35	23.01	45000
GBF1	206.31	46000
L1CAM	139.92	46000
UPF2	147.72	47000
TMOD3	39.57	47000
PRPF31	55.42	52000
DNAJC30	25.95	52000
EIF2D	64.67	52000

AGPAT5	42.04	440000
ATP13A3	137.95	420000
P3H4	50.35	410000
	18.85	400000
FUBP3	61.6	400000
MRPS11	20.6	400000
DHRS4	29.52	400000
GDAP1	41.32	400000
LYPLA1	24.65	390000
SLC25A22	34.45	380000
USP10	87.08	340000
GATB	61.83	330000
CBR4	25.29	330000
CPM	50.48	300000
TSPAN3	28	290000
CDSN	51.49	290000
SIL1	52.05	270000
PRSS21	34.86	260000
ABHD11	34.67	260000
MRPS33	12.62	260000
TXNRD2	56.47	240000
SVIP	8.44	240000
HGSNAT	73.25	240000
VARS1	140.39	230000
CCDC127	30.82	230000
SNX3	18.75	220000
MRPL35	21.5	220000
AP2M1	44.84	220000
HEY1	28.99	210000
ENDOG	32.6	210000
POGLUT1	46.16	200000
ASCC3	251.3	200000
NSUN4	43.06	200000
GK	61.21	200000
COL3A1	138.48	190000
MINPP1	55.02	180000
APOOL	29.14	180000
EPHA3	110.06	180000
DERL1	28.78	180000
GOLGA4	260.98	180000
M6PR	30.97	170000
GCC1	87.76	170000
POLG2	54.88	170000
PDP1	61.02	160000
HYDIN	575.53	160000

TRIP12	220.3	53000
AMIGO2	57.9	53000
PLEKHG3	134.33	55000
GNL3	61.95	57000
HNRNPDL	46.41	57000
DNAAF5	93.46	58000
TRPM7	212.56	58000
PRRC2B	242.82	59000
NAT10	115.66	60000
HSPA14	54.76	60000
CELSR3	357.96	61000
FAM120C	120.51	61000
TPST1	42.16	63000
TEX10	105.61	64000
IQGAP3	184.58	65000
NXF1	70.14	66000
RETREG3	51.36	66000
SCYL2	103.64	66000
LONP2	94.56	67000
PHIP	206.56	67000
VTI1B	26.67	68000
SYNM	172.76	69000
PAICS	47.05	69000
UTP20	318.18	69000
EPN2	68.44	70000
CNN2	33.68	71000
TUBB6	49.82	71000
NIPBL	315.85	72000
UTRN	394.22	75000
TRIO	346.68	75000
CCNY	39.31	75000
KIF22	73.22	76000
G6PD	59.22	76000
DDX20	92.18	77000
LRP1	504.28	79000
NT5DC3	63.38	80000
ZC3H7B	109.79	80000
ADCK1	60.54	80000
NECTIN2	57.71	82000
NF1	319.17	82000
SLC19A1	64.83	83000
HYCC1	57.59	85000
CORO1B	54.2	87000
ARL8B	21.53	90000
DHRS7B	35.1	91000

ABCA2	269.66	160000
ARSB	59.65	160000
DNAJB1	38.02	150000
ZBTB14	50.92	150000
CTSL	37.54	140000
TCF3	18.07	140000
MT-CYB	42.69	130000
LIG3	112.83	130000
BMP2K	129.09	130000
PDK3	46.91	120000
METTL17	50.7	110000
PLA2G15	46.63	110000
SMC3	141.45	110000
CLASP1	169.35	100000
BPNT2	38.66	95000
PCNX3	221.9	94000
ACOX3	77.58	91000
PSMA4	29.47	89000
MRPS10	22.99	88000
ATP2C1	100.51	85000
NCEH1	45.78	81000
DNAJC15	16.37	81000
EEF1A2	50.44	78000
NIPSNAP3A	28.45	77000
ROR2	104.69	76000
RAB21	24.33	75000
LUC7L2	46.49	73000
TMX3	51.84	71000
MSI2	35.17	71000
TFCP2	57.22	71000
EPHX1	52.91	68000
ICAM1	57.79	67000
GUF1	74.28	64000
ARMC8	75.46	63000
AP1B1	104.54	60000
KPNA3	57.77	59000
GSTZ1	24.1	58000
PTPRD	214.62	58000
FNDC3A	131.77	55000
ATP6V1E1	26.13	55000
DGUOK	32.04	55000
KYAT3	51.37	55000
MPG	32.85	54000
SLC36A4	56.12	52000
GJA1	42.98	48000

SLC26A6	82.91	92000
EEF1B2	24.75	96000
DNMT1	183.05	98000
NID2	151.16	99000
CHID1	44.91	100000
EXOC4	110.43	100000
INCENP	105.36	100000
OSBPL10	83.92	100000
DDX27	89.78	100000
DAZAP1	43.36	110000
CFL2	18.72	110000
DNAJC5	22.13	110000
RAD21	71.64	110000
RAD50	153.8	110000
VPS11	107.77	110000
VPS39	101.74	110000
UQCC1	34.58	120000
RDH14	36.84	120000
NUP54	55.4	120000
SLC9A1	90.71	120000
TBC1D10B	87.14	130000
LARP4B	80.5	130000
RAP2B	20.49	130000
PFKP	85.54	130000
THEM4	27.11	140000
SLC7A11	55.39	140000
NUP214	213.49	140000
MDC1	226.53	140000
BCAM	67.36	140000
PI4K2B	54.71	140000
SEPTIN9	65.36	140000
DDX24	96.27	150000
MRPL13	20.68	160000
RALGAPA1	229.69	160000
ANKFY1	128.32	160000
MYO6	149.6	160000
TRMT61B	52.93	170000
NDUFB6	15.48	170000
DHX29	155.14	180000
PSMC3	49.17	180000
VPS4B	49.27	180000
MKI67	358.47	180000
SF3A1	88.83	190000
MFF	25.29	200000
MRPS36	11.46	210000

IST1	39.73	47000
RAB6C	28.34	46000
XPOT	109.89	44000
CNNM4	86.55	42000
PLPP1	32.14	41000
RHOA	21.75	41000
MFSD5	49.73	40000
ESPL1	233.03	36000
NUP98	197.46	35000
DIP2B	171.38	35000
CLDN12	27.09	33000
SRRT	100.6	33000
SEC24A	119.67	32000
POLR3A	155.54	32000
QTRT1	44.02	28000
MYORG	81.04	28000
ACOX1	74.38	27000
COIL	62.57	25000
ATL2	66.19	25000
GCC2	195.79	23000
CUX1	164.09	22000
DGKE	63.88	22000
COX11	31.41	21000
TTYH3	57.51	19000
NOC3L	92.49	19000
SLC25A40	38.1	18000
ITGB8	85.58	18000
PWP2	102.39	14000
TYRO3	96.84	7100
ITSN2	193.34	6000
PSPC1	58.71	5400
TBC1D9B	140.44	4000

HNRNPUL2	85.05	210000
GFAP	8.37	210000
LARP1B	105.26	210000
RNPS1	34.19	220000
GPAA1	67.58	220000
STUB1	34.83	230000
GNAQ	42.12	230000
PLCD3	89.2	240000
MRPS14	15.13	240000
ADAM10	84.09	240000
HDAC1	55.07	240000
WWP2	98.85	240000
AIMP2	35.33	250000
SLC26A2	81.61	250000
UMPS	52.19	250000
METTL15	46.09	250000
SMARCA1	122.53	250000
PKP4	131.79	260000
NDUFS4	20.1	260000
TPD52L2	22.22	260000
ZMPSTE24	54.78	270000
LRP10	76.14	270000
ERI1	40.04	280000
TMEM9	20.56	280000
CLIC1	26.91	280000
SLC4A7	135.96	290000
CYFIP1	145.09	290000
RRAS	23.47	290000
PLD1	124.11	300000
GGT1	61.37	300000
LYN	58.54	300000
MAVS	56.49	310000
NAT2	33.55	310000
PPFIBP1	113.95	310000
NUP85	74.97	320000
NUDT9	39.1	320000
ATP6V1D	28.25	320000
MCM3	90.92	330000
NUP107	106.31	330000
GRHPR	35.65	330000
ALG5	36.92	330000
EFR3A	92.86	340000
MAGED2	64.91	350000
JAGN1	21.11	350000
NEU1	45.44	350000

RPL30	12.78	360000
TKT	67.83	360000
PDE3A	124.9	360000
ALCAM	65.06	360000
RHOBTB3	69.37	360000
TOMM7	6.24	370000
PRKAA1	63.97	370000
PSMB5	28.46	370000
MRPL55	15.12	380000
NSUN5	46.66	380000
THOP1	78.79	380000
RFC4	39.66	410000
PPP1R9B	89.28	410000
MPV17L2	23.16	410000
PRKCE	83.62	410000
PSMA7	27.87	420000
CPNE8	63.07	420000
TTN	3813.65	430000
PSMD3	60.94	430000
AGMAT	37.64	430000
ALAS1	70.54	430000
PAFAH1B1	46.61	430000
EPB41	96.96	440000
MCM4	96.5	440000
OSBPL11	83.59	440000
STX4	34.16	450000
HAX1	31.6	450000
AK4	25.25	450000
DDX23	95.52	480000
DIAPH1	141.26	490000
NOL11	81.07	490000
MTMR4	133.27	490000
UFD1	34.48	500000
STX6	29.16	500000
MGST1	17.59	510000
RPAP3	75.67	520000
LEMD2	56.94	530000
MACF1	177.17	530000
NOP56	66.01	540000
LSG1	75.18	540000
SYNJ2BP	15.92	550000
MIX23	16.61	550000
LUZP1	120.2	560000
TPI1	26.65	570000
MYBL1	85.83	580000

SLC12A4	120.57	590000
MZB1	8.26	590000
TNFRSF10B	47.85	600000
ANKLE2	104.05	640000
STAT3	88.01	660000
P3H2	80.93	660000
TMEM97	20.83	710000
FKBP10	64.2	750000
NPTN	44.36	750000
NDUFS7	23.55	760000
SORT1	92.01	770000
MCM2	101.83	790000
FBL	33.76	790000
LPCAT1	59.11	820000
ITM2B	30.32	820000
GCDH	48.1	840000
CAPN7	92.59	850000
DDX50	82.51	870000
SLC39A1	34.23	870000
EHD2	61.12	920000
YES1	60.76	990000
TMEM132A	110.04	1000000
LMBRD1	61.35	1000000
TMEM11	21.53	1100000
EIF3D	63.93	1200000
AHCY	47.69	1200000
UQCR10	7.3	1300000
ATP11A	129.67	1400000
CTPS1	66.65	1400000
HMGA1	11.67	1400000
CDK14	53.02	1400000
COX6C	8.78	1500000
SRSF4	56.65	1500000
MFSD1	51.17	1500000
LDLR	95.31	1500000
ITSN1	195.3	1800000
CAV2	18.28	1800000
KIF5B	109.62	1900000
HLA-A	40.82	1900000
ATP6AP2	38.98	1900000
RAC2	21.42	1900000
SMC1A	143.14	2200000
PLXNB2	205	2300000
PHGDH	56.61	2700000
TMED7	25.16	2700000

VPS8	21.93	2700000
TM9SF3	67.84	2800000
RAN	24.41	3300000
NRIP1	126.86	3800000
RPL23A	17.68	3900000
NCAPG	114.26	5900000
UBXN4	56.74	6300000
RPL5	34.34	6700000
ATP1B1	35.04	7700000
CNN3	36.39	8000000
HNRNPC	33.65	18000000
RPS11	18.42	39000000
