# Supplementary material for: SARS-CoV-2 ORF3a blocks lysosomal cholesterol egress by disrupting VPS39-regulated NPC2 trafficking and BMP metabolism
Source: Cell Rep. Author manuscript; Available in PMC 2026 Jul 17. (PMC13378056; doi:10.1016/j.celrep.2026.117544)
Supplement: 4 [file NIHMS2190776-supplement-4.pdf]

Gene Symbol	MWT(kDa)	Sum Intensity ORF3a-lyso-IP	Sum Intensity W193A-lyso-IP	Log2 fold	
MYCBP2	513.31	1100000	6700	7.359127	1100000
MCM5	82.23	8900000	85000	6.710199	8900000
DBT	53.45	620000	8200	6.2405	620000
GIGYF2	149.98	640000	9500	6.074001	640000
CSE1L	110.35	77000000	2E+06	5.266787	77000000
FASTKD5	86.52	370000	14000	4.724027	370000
ACSF3	64.09	1000000	38000	4.717857	1000000
YWHAE	29.16	550000	23000	4.579726	550000
TAP1	80.91	920000	49000	4.23078	920000
EXOC2	104	1500000	86000	4.124482	1500000
ANKH	54.21	75000	4700	3.996158	75000
ENG	70.53	5500000	360000	3.933363	5500000
RPA1	68.1	560000	41000	3.771731	560000
EFNB1	37.98	2000000	150000	3.736966	2000000
ARF6	20.07	530000	44000	3.590417	530000
PTPRS	216.9	790000	66000	3.581315	790000
ANKRD17	274.09	680000	60000	3.5025	680000
RTN4	129.85	2000000	180000	3.473931	2000000
CDH2	99.75	19000000	2E+06	3.399931	19000000
PSMC2	48.6	290000	28000	3.372554	290000
SNRNP200	244.35	140000	14000	3.321928	140000
ALDH1L2	101.68	14000000	1E+06	3.321928	14000000
HNRNPF	45.64	740000	74000	3.321928	740000
NELFB	65.66	420000	45000	3.222392	420000
POGLUT3	58.54	270000	29000	3.218835	270000
SEMA3C	85.15	2100000	250000	3.070389	2100000
NSDHL	41.87	650000	82000	2.986744	650000
LRRC47	63.43	220000	29000	2.923379	220000
APOB	515.28	18000000	2E+06	2.906891	18000000
CHD4	217.87	1600000	220000	2.862496	1600000
NUP205	227.78	3400000	500000	2.765535	3400000
SNX9	66.55	1600000	240000	2.736966	1600000
RAB10	22.53	1200000	180000	2.736966	1200000
TGFBI	74.63	990000	150000	2.722466	990000
NCAPD2	157.08	270000	41000	2.719264	270000
HSPB1	22.77	720000	110000	2.710493	720000
ARHGAP21	217.33	540000	86000	2.650551	540000
PPP2R1B	66.17	150000	24000	2.643856	150000
CCAR2	102.84	7400000	1E+06	2.624491	7400000
EPHB2	117.42	450000	73000	2.623957	450000
LBR	70.66	1800000	300000	2.584963	1800000
HACD3	43.13	3100000	520000	2.575685	3100000
BAIAP2	60.83	380000	64000	2.569856	380000
PTPRJ	145.85	1600000	270000	2.567041	1600000
SMC4	147.09	1600000	270000	2.567041	1600000
NES	177.33	2800000	480000	2.544321	2800000
TMTC3	103.94	570000	98000	2.540108	570000
TUBB4B	49.8	3300000	590000	2.483679	3300000
CCT8	59.58	1900000	340000	2.482393	1900000
LIMS1	37.23	1400000	260000	2.428843	1400000
PDS5A	150.73	300000	56000	2.421464	300000
SNRPD2	13.52	630000	120000	2.392317	630000
MYBBP1A	148.76	210000	40000	2.392317	210000
CCT6A	57.99	5100000	980000	2.379644	5100000
GCN1	292.52	1600000	310000	2.367732	1600000
PSMC6	44.15	160000	32000	2.321928	160000
DNAJC11	63.24	2200000	450000	2.289507	2200000
XPO7	123.83	220000	45000	2.289507	220000
XPO5	136.22	430000	88000	2.288761	430000
RUVBL2	51.12	1800000	370000	2.2824	1800000
CAPZB	30.61	3100000	640000	2.276124	3100000

ALDOA	39.4	4800000	1E+06	2.263034	4800000
ISOC2	22.32	230000	48000	2.260528	230000
NACA	23.37	2200000	460000	2.257798	2200000
TUBAL3	49.88	1400000	300000	2.222392	1400000
MAIP1	32.52	390000	84000	2.215013	390000
SEL1L	88.7	190000	41000	2.212304	190000
SUCLG2	46.48	17000000	4E+06	2.199938	17000000
NEFL	61.48	2.3E+08	5E+07	2.14505	230000000
SVIL	247.59	1500000	340000	2.141356	1500000
CHMP6	23.47	1300000	300000	2.115477	1300000
PKM	57.9	16000000	4E+06	2.112475	16000000
SLC12A6	127.53	560000	130000	2.106915	560000
IGF2BP1	63.44	390000	91000	2.099536	390000
THNSL1	83.02	420000	100000	2.070389	420000
TMEM256	11.73	140000	34000	2.04182	140000
DHX36	114.69	74000	18000	2.039528	74000
PNPLA4	27.96	250000	63000	1.988504	250000
ANXA1	38.69	1100000	280000	1.974005	1100000
PDLIM7	49.81	5100000	1E+06	1.971986	5100000
RSL1D1	54.94	420000	110000	1.932886	420000
EIF5B	138.74	990000	260000	1.928917	990000
CCT2	57.45	5300000	1E+06	1.920566	5300000
SLC44A1	73.25	1400000	370000	1.91983	1400000
MRM3	46.99	4900000	1E+06	1.91427	4900000
MRPL22	23.63	12000000	3E+06	1.906891	12000000
NOP2	89.25	260000	70000	1.893085	260000
CHST14	42.97	1000000	270000	1.888969	1000000
HEXB	63.1	1100000	300000	1.874469	1100000
FERMT2	77.81	950000	260000	1.869416	950000
RPL37A	10.27	3500000	960000	1.866249	3500000
XPO1	123.31	2500000	690000	1.85726	2500000
MANF	20.69	360000	100000	1.847997	360000
ASPH	85.81	11000000	3E+06	1.827163	11000000
RUFY1	79.77	1100000	310000	1.827163	1100000
SCP2	58.96	3900000	1E+06	1.825971	3900000
HSPA4	94.27	2300000	650000	1.823122	2300000
SDC4	21.63	4600000	1E+06	1.823122	4600000
ABCC5	160.56	340000	97000	1.809478	340000
FUNDC1	17.17	29000	8300	1.80487	29000
STRAP	38.41	450000	130000	1.791413	450000
AP3D1	130.08	760000	220000	1.788496	760000
PAM16	13.82	690000	200000	1.786596	690000
ABCC4	149.43	2000000	580000	1.785875	2000000
GET3	38.77	320000	93000	1.782769	320000
GNA11	42.1	1100000	320000	1.78136	1100000
UBR4	573.48	410000	120000	1.77259	410000
DYNC1H1	532.07	18000000	5E+06	1.763933	18000000
RPL36	12.25	570000	170000	1.745427	570000
FLNB	277.99	20000000	6E+06	1.736966	20000000
DNM2	98	1800000	540000	1.736966	1800000
CALD1	93.18	2800000	840000	1.736966	2800000
ANXA5	35.91	22000	6600	1.736966	22000
LMO7	192.58	8300000	3E+06	1.731183	8300000
KPNA2	57.83	3300000	1E+06	1.722466	3300000
NPLOC4	68.08	62000	19000	1.706269	62000
CD55	41.37	5200000	2E+06	1.70044	5200000
TUBA1B	50.12	5500000	2E+06	1.693897	5500000
ATXN10	53.45	450000	140000	1.684498	450000
USO1	107.83	7700000	2E+06	1.681824	7700000
PRMT5	72.64	150000	47000	1.67423	150000
MT-ND5	66.98	67000	21000	1.673772	67000
GLG1	134.46	3500000	1E+06	1.669851	3500000
DHX9	140.87	23000000	7E+06	1.655665	23000000
CNOT1	266.77	6600000	2E+06	1.652077	6600000
NDUFAF7	49.21	1300000	420000	1.63005	1300000
ADGRE5	91.81	1300000	420000	1.63005	1300000
ITGAV	115.96	7700000	3E+06	1.62293	7700000
RPS21	9.11	770000	250000	1.62293	770000

SMC2	135.57	2800000	910000	1.621488	2800000
TRIM28	88.49	1200000	390000	1.621488	1200000
CCT5	59.63	490000	160000	1.61471	490000
GTPBP4	73.92	1100000	360000	1.611435	1100000
CFL1	18.49	5800000	2E+06	1.610053	5800000
CCT3	60.5	6100000	2E+06	1.608809	6100000
MGAT4B	63.16	210000	69000	1.605721	210000
RAC1	21.44	1000000	330000	1.599462	1000000
MYH9	226.39	42000000	1E+07	1.584963	42000000
ACTN1	102.99	33000000	1E+07	1.584963	33000000
EIF2A	64.95	1500000	500000	1.584963	1500000
PLIN3	47.05	1500000	500000	1.584963	1500000
RPL15	24.13	3300000	1E+06	1.584963	3300000
CRTAP	46.53	2200000	740000	1.571906	2200000
SMARCA5	121.83	2400000	810000	1.567041	2400000
VAMP7	24.92	2100000	710000	1.564498	2100000
SLC35B2	47.48	1800000	610000	1.561116	1800000
HNRNPD	38.41	5000000	2E+06	1.556393	5000000
DDX17	80.22	4700000	2E+06	1.554589	4700000
DIAPH3	136.84	440000	150000	1.552541	440000
EFTUD2	109.37	760000	260000	1.547488	760000
TMEM214	77.1	1400000	480000	1.544321	1400000
PCBP3	39.44	1100000	380000	1.533432	1100000
YWHAZ	27.73	5200000	2E+06	1.530515	5200000
PLOD2	84.63	4600000	2E+06	1.523562	4600000
RPS15	17.03	8600	3000	1.519374	8600
FASN	273.03	10000000	4E+06	1.514573	10000000
CDC5L	92.19	370000	130000	1.509014	370000
ATP5MF	10.91	7100000	3E+06	1.505891	7100000
EDC4	151.57	240000	85000	1.4975	240000
MSN	67.78	3100000	1E+06	1.494765	3100000
PDCD6IP	95.96	3100000	1E+06	1.494765	3100000
RAB9A	22.82	2000000	710000	1.494109	2000000
PXDN	165.17	4500000	2E+06	1.491853	4500000
PPP1CC	36.96	1600000	570000	1.489038	1600000
PTPN1	49.93	1200000	430000	1.480626	1200000
SAR1A	22.35	2000000	720000	1.473931	2000000
QARS1	87.74	3300000	1E+06	1.459432	3300000
GSTP1	23.34	330000	120000	1.459432	330000
ABCF1	95.87	1400000	510000	1.456858	1400000
RPS28	7.84	4100000	2E+06	1.450661	4100000
TARS1	83.38	1200000	440000	1.447459	1200000
CD59	14.17	6800000	3E+06	1.443607	6800000
LARP1	123.43	1600000	590000	1.439285	1600000
PRDX2	21.88	100000	37000	1.434403	100000
CSNK1G2	47.43	1000000	370000	1.434403	1000000
IPO7	119.44	2700000	1E+06	1.432959	2700000
AHNAK	628.7	2.1E+08	8E+07	1.428843	210000000
YARS2	53.17	5600000	2E+06	1.415037	5600000
COBL	135.53	320000	120000	1.415037	320000
TLN1	269.6	13000000	5E+06	1.407658	13000000
NPM1	32.55	1800000	680000	1.40439	1800000
ANXA2P2	38.63	82000000	3E+07	1.403356	82000000
DDX5	69.1	24000000	9E+06	1.399096	24000000
RUVBL1	50.2	2000000	760000	1.395929	2000000
SLC1A4	55.69	420000	160000	1.392317	420000
STX10	28.1	630000	240000	1.392317	630000
BAZ1B	170.8	1600000	610000	1.391191	1600000
CACNA2D1	124.49	1100000	420000	1.389042	1100000
ILVBL	67.82	170000	65000	1.387023	170000
RANBP2	357.97	4700000	2E+06	1.384664	4700000
MCU	39.84	1200000	460000	1.383329	1200000
GPC1	61.64	2600000	1E+06	1.378512	2600000
SIN3A	145.08	4900	1900	1.366782	4900
NCL	76.57	8500000	3E+06	1.364997	8500000
ITGA1	130.76	1800000	700000	1.36257	1800000
TMEM126B	25.93	36000	14000	1.36257	36000
FDFT1	48.08	950000	370000	1.360402	950000

MATR3	94.56	5900000	2E+06	1.359081	5900000
ERGIC3	43.19	110000	43000	1.355095	110000
EEF2	95.28	14000000	6E+06	1.347923	14000000
CDK5RAP1	67.65	610000	240000	1.345775	610000
SEC23A	86.11	38000	15000	1.341037	38000
KIF11	119.09	910000	360000	1.33787	910000
AFAP1	80.67	930000	370000	1.329705	930000
PRPF8	273.43	3500000	1E+06	1.321928	3500000
HNRNPL	64.09	1700000	680000	1.321928	1700000
LNPEP	117.27	2500000	1E+06	1.321928	2500000
FAHD1	24.83	300000	120000	1.321928	300000
ABCC1	171.48	7700000	3E+06	1.31259	7700000
LDHA	36.67	7700000	3E+06	1.31259	7700000
SUPT16H	119.84	8900000	4E+06	1.305808	8900000
PDIA6	48.09	19000000	8E+06	1.303069	19000000
VIM	53.62	3.7E+09	2E+09	1.302563	3700000000
UPF1	124.27	1800000	730000	1.302029	1800000
SLC1A3	59.53	140000	57000	1.296393	140000
TJP1	195.34	2700000	1E+06	1.295456	2700000
MFF	38.44	780000	320000	1.285402	780000
PACSIN3	48.46	560000	230000	1.283793	560000
BCKDHA	50.44	3400000	1E+06	1.280108	3400000
QSOX2	77.48	850000	350000	1.280108	850000
MRPS34	25.63	1700000	700000	1.280108	1700000
SPTBN2	271.16	24000000	1E+07	1.277534	24000000
STAG2	141.24	16000	6600	1.277534	16000
VPS13A	360.05	460000	190000	1.275634	460000
SYNPO	99.4	2900000	1E+06	1.273018	2900000
ACTA2	41.98	70000000	3E+07	1.271302	70000000
TIMMDC1	32.16	410000	170000	1.270089	410000
DRG1	40.52	2300000	960000	1.260528	2300000
PIK3R4	153.01	910000	380000	1.259867	910000
ENO1	47.14	11000000	5E+06	1.257798	11000000
AXL	98.27	2100000	880000	1.254814	2100000
HNRNPK	50.94	10000000	4E+06	1.251539	10000000
RPL12	17.81	10000000	4E+06	1.251539	10000000
FLII	144.66	140000	59000	1.24664	140000
XRCC6	69.8	9000000	4E+06	1.243926	9000000
NDUFB4	15.2	970000	410000	1.242361	970000
RPL7	29.21	26000000	1E+07	1.241008	26000000
EPHA2	108.2	2600000	1E+06	1.241008	2600000
VANGL1	59.94	330000	140000	1.237039	330000
EIF2S2	38.36	4000000	2E+06	1.234465	4000000
H1-5	22.57	12000000	5E+06	1.234465	12000000
RBMX	42.31	4700000	2E+06	1.232661	4700000
CKAP5	225.35	8200000	4E+06	1.228269	8200000
TANC1	202.09	9600	4100	1.22741	9600
XRCC5	82.65	8400000	4E+06	1.222392	8400000
GNAI3	40.51	5600000	2E+06	1.222392	5600000
ITPR3	303.91	350000	150000	1.222392	350000
PLP2	16.68	3500000	2E+06	1.222392	3500000
ATP2A1	110.18	17000000	7E+06	1.219566	17000000
SPTBN1	274.44	1.6E+08	7E+07	1.213404	160000000
IDH3B	42.16	3700000	2E+06	1.209453	3700000
RACGAP1	70.98	3000000	1E+06	1.206451	3000000
EEF1G	50.09	6900000	3E+06	1.201634	6900000
CCDC47	55.84	3900000	2E+06	1.197939	3900000
SDC2	22.15	2200000	960000	1.196397	2200000
RPL6	32.71	9600000	4E+06	1.192645	9600000
PPP1CA	37.49	640000	280000	1.192645	640000
SNAP23	23.34	1300000	570000	1.189478	1300000
CPOX	50.12	130000	57000	1.189478	130000
GNA13	44.02	570000	250000	1.189034	570000
DNAJA2	45.72	500000	220000	1.184425	500000
RPS10	18.89	5900000	3E+06	1.182203	5900000
TIMM17B	18.26	340000	150000	1.180572	340000
TUBB8B	49.54	19000000	8E+06	1.177538	19000000
CLN5	41.47	520000	230000	1.176878	520000

TPP2	138.26	9700000	4E+06	1.173648	9700000
CCT4	57.89	2700000	1E+06	1.169925	2700000
DAG1	97.38	2000000	890000	1.168123	2000000
UBA1	117.77	1100000	490000	1.16665	1100000
SNAP91	92.44	380000	170000	1.160465	380000
TUBB	49.64	21000000	9E+06	1.159657	21000000
VAPB	27.21	2900000	1E+06	1.157541	2900000
DDX21	87.29	4900000	2E+06	1.155278	4900000
RPS25	13.73	16000000	7E+06	1.152003	16000000
NUDT19	42.21	420000	190000	1.14439	420000
RHOC	21.99	210000	95000	1.14439	210000
MRPS15	29.82	1500000	680000	1.141356	1500000
TFAM	29.08	4400000	2E+06	1.137504	4400000
MPDU1	26.62	330000	150000	1.137504	330000
RETSAT	66.78	180000	82000	1.134301	180000
OPA1	111.56	14000000	6E+06	1.129283	14000000
BCS1L	47.5	3500000	2E+06	1.129283	3500000
EIF4A3	46.84	1200000	550000	1.125531	1200000
PLK1	68.21	480000	220000	1.125531	480000
EIF4A1	46.12	10000000	5E+06	1.120294	10000000
PUS1	47.44	1300000	600000	1.115477	1300000
TOP2B	183.15	390000	180000	1.115477	390000
CPNE3	60.09	130000	60000	1.115477	130000
VPS45	65.04	950000	440000	1.110424	950000
ATP13A1	132.87	840000	390000	1.106915	840000
ITGA3	116.54	710000	330000	1.105353	710000
RPL9P9	21.85	3000000	1E+06	1.099536	3000000
CLASP2	140.98	120000	56000	1.099536	120000
ACTN4	104.79	7.7E+08	4E+08	1.096862	770000000
COL4A2	167.45	6200000	3E+06	1.096215	6200000
GANAB	96.48	960000	450000	1.093109	960000
SUMF2	33.82	830000	390000	1.089637	830000
ARCN1	57.17	870000	410000	1.085391	870000
IMMT	73.21	2100000	990000	1.084889	2100000
ARPC4	19.65	360000	170000	1.082462	360000
SLIRP	12.34	5500000	3E+06	1.08092	5500000
SPPL2B	64.6	74000	35000	1.08017	74000
NSUN2	86.42	380000	180000	1.078003	380000
FAU	14.38	3800000	2E+06	1.078003	3800000
PDHX	54.09	2000000	950000	1.074001	2000000
MRPS31	45.29	2300000	1E+06	1.06413	2300000
EGFR	134.19	14000000	7E+06	1.063194	14000000
ANO6	106.1	1400000	670000	1.063194	1400000
KIF14	186.37	2000000	960000	1.058894	2000000
FLAD1	65.22	52000	25000	1.056584	52000
MIA3	213.57	5400000	3E+06	1.054448	5400000
FLNA	280.56	1.7E+08	8E+07	1.051839	170000000
RPL17	21.38	2900000	1E+06	1.050626	2900000
ECHS1	31.37	6200000	3E+06	1.047306	6200000
VASN	71.67	3100000	2E+06	1.047306	3100000
CAV1	20.46	6200000	3E+06	1.047306	6200000
SLC5A6	68.6	620000	300000	1.047306	620000
TCP1	60.31	3300000	2E+06	1.044394	3300000
TUBA3C	49.93	35000000	2E+07	1.04182	35000000
CAND1	136.29	720000	350000	1.040642	720000
LCP1	70.24	760000	370000	1.038474	760000
ACTR1A	42.59	390000	190000	1.037475	390000
EZR	69.37	18000000	9E+06	1.032421	18000000
PLOD1	83.5	10000000	5E+06	1.029146	10000000
HNRNPCL1	32.12	5700000	3E+06	1.025535	5700000
YWHAB	28.06	590000	290000	1.024662	590000
COPB1	107.07	1200000	590000	1.024248	1200000
HNRNPR	70.9	13000000	6E+06	1.022368	13000000
RPS19	16.05	13000000	6E+06	1.022368	13000000
TAGLN2	22.38	150000	74000	1.019365	150000
AIMP1	34.33	890000	440000	1.016302	890000
GPD2	80.8	18000000	9E+06	1.01612	18000000
H2AC17	14.08	1800000	890000	1.01612	1800000

RPS3A	29.93	9900000	5E+06	1.014647	9900000
PPIA	18	2000000	990000	1.0145	2000000
CTNNB1	85.44	17000000	9E+06	1	17000000
EEF1A1	50.11	78000000	4E+07	1	78000000
TUBB4A	49.55	12000000	6E+06	1	12000000
PTBP1	59.6	7200000	4E+06	1	7200000
EIF3C	105.28	6000000	3E+06	1	6000000
NPR3	59.77	3000000	2E+06	1	3000000
PLS3	70.77	2200000	1E+06	1	2200000
RPL31	14.45	5400000	3E+06	1	5400000
PFN1	15.04	2200000	1E+06	1	2200000
YWHAQ	27.75	3200000	2E+06	1	3200000
MRPL18	20.56	680000	340000	1	680000
HSD17B11	32.91	320000	160000	1	320000
DYSF	237.14	680000	340000	1	680000
TM9SF2	75.73	1800000	910000	0.984058	1800000
SRSF3	19.32	1800000	910000	0.984058	1800000
LMO7	145.33	890000	450000	0.98388	890000
RPLP0	34.25	16000000	8E+06	0.982078	16000000
CLCC1	61.98	160000	81000	0.982078	160000
EIF3B	92.42	7100000	4E+06	0.979822	7100000
PIEZO1	286.6	1200000	610000	0.976153	1200000
PIP5K1A	62.59	550000	280000	0.974005	550000
SFPQ	76.1	19000000	1E+07	0.969943	19000000
DDX28	59.54	1900000	970000	0.969943	1900000
PTPRF	212.74	1800000	920000	0.968291	1800000
WDR11	136.6	430000	220000	0.966833	430000
IBA57	38.13	740000	380000	0.961526	740000
FUS	53.39	3300000	2E+06	0.956931	3300000
FKBP2	15.64	1300000	670000	0.956279	1300000
DNASE2	39.56	560000	290000	0.949374	560000
CAVIN1	43.45	7300000	4E+06	0.941897	7300000
CAPZA1	32.9	2300000	1E+06	0.938599	2300000
DHCR7	54.45	2300000	1E+06	0.938599	2300000
GNG12	8	920000	480000	0.938599	920000
RTCB	55.17	230000	120000	0.938599	230000
DDX3X	73.2	21000000	1E+07	0.932886	21000000
PRDX3	27.68	6300000	3E+06	0.932886	6300000
RPL27A	16.55	4200000	2E+06	0.932886	4200000
HNRNPM	77.46	16000000	8E+06	0.929611	16000000
TMBIM1	34.58	400000	210000	0.929611	400000
NUP188	195.92	590000	310000	0.928447	590000
CTNNA1	100.01	19000000	1E+07	0.925999	19000000
DSG2	122.22	19000000	1E+07	0.925999	19000000
ITGB1	88.36	55000000	3E+07	0.923379	55000000
HNRNPC	31.95	180000	95000	0.921997	180000
TOMM22	15.51	10000000	5E+06	0.915936	10000000
SAFB	102.58	1000000	530000	0.915936	1000000
TOP2A	174.28	8300000	4E+06	0.915608	8300000
CDC42	21.25	640000	340000	0.912537	640000
TWF1	40.26	620000	330000	0.909802	620000
SLC2A1	54.05	30000000	2E+07	0.906891	30000000
GAPDH	36.03	15000000	8E+06	0.906891	15000000
FAR1	59.32	1500000	800000	0.906891	1500000
SHMT1	53.05	3000000	2E+06	0.906891	3000000
SLC30A1	55.26	300000	160000	0.906891	300000
KIF23	109.99	4300000	2E+06	0.902703	4300000
RPL10	24.56	860000	460000	0.902703	860000
FYN	60.72	170000	91000	0.901596	170000
MYO1B	131.9	14000000	8E+06	0.900464	14000000
NLN	80.6	2800000	2E+06	0.900464	2800000
GIPC1	36.03	280000	150000	0.900464	280000
EPPK1	555.32	97000000	5E+07	0.899473	97000000
RAB1A	22.66	8000000	4E+06	0.895663	8000000
NOP9	69.39	93000	50000	0.895303	93000
HM13	41.46	6500000	4E+06	0.893085	6500000
ECPAS	204.16	130000	70000	0.893085	130000
STXBP3	67.72	1000000	540000	0.888969	1000000

PRKDC	468.79	61000000	3E+07	0.886343	61000000
IQGAP1	189.13	33000000	2E+07	0.874469	33000000
PI4K2A	53.99	2200000	1E+06	0.874469	2200000
SPTB	246.32	1100000	600000	0.874469	1100000
RAB13	22.76	22000000	1E+07	0.874469	22000000
TRIP13	48.52	220000	120000	0.874469	220000
SPTAN1	284.36	1.3E+08	7E+07	0.872621	130000000
CLTCL1	186.91	15000000	8E+06	0.871267	15000000
MARCHF5	31.21	170000	93000	0.870232	170000
DDX6	54.38	420000	230000	0.868755	420000
MT-CO2	25.55	3100000	2E+06	0.866733	3100000
RPL14	23.42	710000	390000	0.864345	710000
OAT	48.5	10000000	6E+06	0.862496	10000000
SSBP1	17.25	20000000	1E+07	0.862496	20000000
MTX2	29.74	400000	220000	0.862496	400000
ALDH9A1	53.77	780000	430000	0.859137	780000
SLC1A5	56.56	29000000	2E+07	0.857981	29000000
MRPS16	15.34	2900000	2E+06	0.857981	2900000
NONO	54.2	6700000	4E+06	0.856636	6700000
ATP5PD	18.48	1500000	830000	0.853779	1500000
TMEM33	27.96	6500000	4E+06	0.852443	6500000
RPS24	15.41	1300000	720000	0.852443	1300000
MTX1	51.43	1300000	720000	0.852443	1300000
RPS26	13.01	9200000	5E+06	0.851137	9200000
CYP51A1	57.24	4500000	3E+06	0.847997	4500000
FXR1	69.68	3600000	2E+06	0.847997	3600000
CCT7	59.33	3600000	2E+06	0.847997	3600000
PGRMC2	23.8	2700000	2E+06	0.847997	2700000
ARF3	20.59	810000	450000	0.847997	810000
YWHAH	28.2	450000	250000	0.847997	450000
CYB5R1	34.07	1200000	670000	0.840801	1200000
SPCS3	20.3	1200000	670000	0.840801	1200000
MRPL15	33.4	3400000	2E+06	0.839535	3400000
TMPO	75.45	3400000	2E+06	0.839535	3400000
MRPL11	20.67	5900000	3E+06	0.838249	5900000
STT3A	80.48	5000000	3E+06	0.836501	5000000
RAB5A	23.64	10000000	6E+06	0.836501	10000000
PTRH2	19.18	1000000	560000	0.836501	1000000
TIMM23	21.93	1500000	840000	0.836501	1500000
FAT1	505.96	410000	230000	0.83399	410000
RPS18	17.71	16000000	9E+06	0.830075	16000000
TMEM165	34.88	3200000	2E+06	0.830075	3200000
NUP93	93.43	3200000	2E+06	0.830075	3200000
IMPDH2	55.77	1400000	790000	0.825502	1400000
ROBO1	180.82	1700000	960000	0.824428	1700000
EIF3L	66.68	2300000	1E+06	0.823122	2300000
ASAH1	44.63	2300000	1E+06	0.823122	2300000
MCM7	81.26	230000	130000	0.823122	230000
CEPT1	46.52	460000	260000	0.823122	460000
PTCD1	78.81	1200000	680000	0.819428	1200000
TOMM40	37.87	37000000	2E+07	0.817136	37000000
ALPL	57.27	8100000	5E+06	0.816288	8100000
MPST	33.16	440000	250000	0.815575	440000
MRPL46	31.69	1600000	910000	0.814133	1600000
LAMC1	177.49	5800000	3E+06	0.813587	5800000
PARP1	113.01	10000000	6E+06	0.810966	10000000
SAFB2	107.41	2100000	1E+06	0.807355	2100000
FMR1	71.13	420000	240000	0.807355	420000
CBARP	73.88	49000	28000	0.807355	49000
NFU1	28.44	49000	28000	0.807355	49000
SCRIB	174.81	11000000	6E+06	0.80408	11000000
STT3B	93.61	3300000	2E+06	0.796467	3300000
SNTB2	57.91	660000	380000	0.796467	660000
SERBP1	44.94	1700000	980000	0.794681	1700000
PKN2	111.96	2600000	2E+06	0.793549	2600000
RPL4	47.67	26000000	2E+07	0.793549	26000000
PLEC	531.47	1.9E+09	1E+09	0.788496	1900000000
ANXA2	38.58	19000000	1E+07	0.788496	19000000

B3GAT3	37.1	190000	110000	0.788496	190000
DEK	42.65	190000	110000	0.788496	190000
SFXN4	37.97	380000	220000	0.788496	380000
BASP1	22.68	1.5E+08	9E+07	0.785875	150000000
PDPR	99.3	8100000	5E+06	0.785261	8100000
CD44	81.49	16000000	9E+06	0.782769	16000000
DIDO1	243.72	16000	9300	0.782769	16000
ITGA5	114.46	1100000	640000	0.78136	1100000
BCL2L13	52.69	910000	530000	0.779874	910000
NRAS	21.22	2400000	1E+06	0.777608	2400000
ITCH	102.74	360000	210000	0.777608	360000
ABCF2	71.24	360000	210000	0.777608	360000
TOP1MT	69.83	120000	70000	0.777608	120000
EIF4G1	175.38	7700000	5E+06	0.774933	7700000
CORO1C	53.22	7700000	5E+06	0.774933	7700000
MAN2B1	113.67	65000	38000	0.77444	65000
U2AF2	53.47	130000	76000	0.77444	130000
GATD3B	28.12	700000	410000	0.771731	700000
CDC42BPB	194.19	5800000	3E+06	0.770518	5800000
NAPA	33.21	870000	510000	0.770518	870000
SRSF1	27.73	290000	170000	0.770518	290000
WASHC5	134.2	92000	54000	0.768674	92000
PCK1	69.15	340000	200000	0.765535	340000
OGDH	115.86	22000000	1E+07	0.758992	22000000
ACTB	41.71	2.2E+09	1E+09	0.758992	2200000000
COLGALT1	71.59	11000000	7E+06	0.758992	11000000
RPL18	21.62	22000000	1E+07	0.758992	22000000
LRRC8A	94.14	1100000	650000	0.758992	1100000
GLT8D1	41.91	660000	390000	0.758992	660000
CTPS2	65.64	220000	130000	0.758992	220000
RPLP1	11.51	220000	130000	0.758992	220000
EIF3A	166.47	12000000	7E+06	0.757143	12000000
PRXL2A	25.75	270000	160000	0.754888	270000
RRM1	90.01	270000	160000	0.754888	270000
CSDE1	88.83	81000	48000	0.754888	81000
RDX	68.52	640000	380000	0.752072	640000
GPX8	23.87	320000	190000	0.752072	320000
RABAC1	20.63	320000	190000	0.752072	320000
EIF2S3	51.08	820000	490000	0.742842	820000
ACSL3	80.37	9700000	6E+06	0.741932	9700000
SND1	101.93	20000000	1E+07	0.736966	20000000
ATP5PB	28.89	20000000	1E+07	0.736966	20000000
PALS2	61.08	3500000	2E+06	0.736966	3500000
RPS15A	14.83	8500000	5E+06	0.736966	8500000
PPP2R1A	65.27	350000	210000	0.736966	350000
PCBP2	38.56	5500000	3E+06	0.736966	5500000
GEMIN5	168.48	1200000	720000	0.736966	1200000
MRPS18B	29.38	1600000	960000	0.736966	1600000
	43.9	200000	120000	0.736966	200000
SLC36A1	53.04	120000	72000	0.736966	120000
STIM1	77.38	140000	84000	0.736966	140000
PLCB4	134.38	5800000	4E+06	0.728698	5800000
CTNND1	108.1	8100000	5E+06	0.72514	8100000
DHX30	133.85	38000000	2E+07	0.724366	38000000
DDX1	82.38	760000	460000	0.724366	760000
SLC39A14	54.18	3800000	2E+06	0.724366	3800000
SLC12A7	119.03	3300000	2E+06	0.722466	3300000
SLC52A2	45.75	3300000	2E+06	0.722466	3300000
AP2B1	104.49	28000000	2E+07	0.719892	28000000
MYO1C	121.61	28000000	2E+07	0.719892	28000000
IFITM1	13.96	5600000	3E+06	0.719892	5600000
TOR1AIP1	66.21	970000	590000	0.71727	970000
GPRC5A	40.22	1200000	730000	0.717066	1200000
COL1A1	138.83	4600000	3E+06	0.716207	4600000
FAF2	52.59	6900000	4E+06	0.716207	6900000
RPL22	14.78	11000000	7E+06	0.715271	11000000
PSMB3	22.93	110000	67000	0.715271	110000
COA3	11.72	1100000	670000	0.715271	1100000

CADM1	48.48	770000	470000	0.712198	770000
LDHB	36.62	1800000	1E+06	0.710493	1800000
CSTF3	82.87	18000	11000	0.710493	18000
H3C12	15.39	80000000	5E+07	0.707218	80000000
RPN1	68.53	31000000	2E+07	0.706269	31000000
HAUS5	71.64	62000	38000	0.706269	62000
IDH3A	39.57	8800000	5E+06	0.704544	8800000
TMPO	50.64	2600000	2E+06	0.70044	2600000
TSPAN6	27.55	650000	400000	0.70044	650000
SERINC1	50.46	940000	580000	0.696608	940000
POLDIP2	42.01	3400000	2E+06	0.695145	3400000
EIF3E	52.19	3400000	2E+06	0.695145	3400000
RPL8	28.01	3400000	2E+06	0.695145	3400000
SSR4	18.99	5500000	3E+06	0.693897	5500000
RPS16	16.44	21000000	1E+07	0.691878	21000000
SCD	41.5	2100000	1E+06	0.691878	2100000
FOXRED2	77.74	21000	13000	0.691878	21000
SF3B1	145.74	1000000	620000	0.68966	1000000
HSPA1A	70.01	74000000	5E+07	0.685891	74000000
ACOT13	14.95	3700000	2E+06	0.685891	3700000
HIP1R	119.31	370000	230000	0.685891	370000
B4GAT1	47.09	370000	230000	0.685891	370000
B4GALT5	45.09	370000	230000	0.685891	370000
MTCH1	41.52	4500000	3E+06	0.684498	4500000
RCE1	35.81	53000	33000	0.683526	53000
PTBP3	59.65	6900000	4E+06	0.68226	6900000
MAGT1	38.01	85000	53000	0.68147	85000
RCC1L	49.87	1600000	1E+06	0.678072	1600000
TIMM13	10.49	560000	350000	0.678072	560000
KLHL9	69.38	80000	50000	0.678072	80000
TIMM50	39.62	11000000	7E+06	0.672835	11000000
	11.77	51000000	3E+07	0.672425	51000000
PRDX1	22.1	5100000	3E+06	0.672425	5100000
RARS1	75.33	7000000	4E+06	0.669851	7000000
PTCD3	78.5	3500000	2E+06	0.669851	3500000
GAPVD1	164.88	350000	220000	0.669851	350000
ERO1A	54.36	2700000	2E+06	0.667425	2700000
LMNB2	69.91	4600000	3E+06	0.665581	4600000
SLC7A5	54.97	65000000	4E+07	0.664816	65000000
PNPT1	85.9	19000000	1E+07	0.662965	19000000
CDH11	87.91	190000	120000	0.662965	190000
HNRNPA1	38.72	3000000	2E+06	0.658963	3000000
RANGAP1	63.5	3000000	2E+06	0.658963	3000000
APMAP	46.45	4100000	3E+06	0.657112	4100000
PNPLA6	150.86	410000	260000	0.657112	410000
HSP90AB1	83.21	63000000	4E+07	0.655352	63000000
HLA-A	41.42	1400000	890000	0.65355	1400000
LMNB1	66.37	22000000	1E+07	0.652077	22000000
SDHB	31.61	7700000	5E+06	0.652077	7700000
DDX39A	49.1	770000	490000	0.652077	770000
SLC25A19	35.49	13000000	8E+06	0.647328	13000000
HPDL	39.36	3600000	2E+06	0.646363	3600000
TRIM21	54.14	2500000	2E+06	0.643856	2500000
LGMN	49.38	500000	320000	0.643856	500000
CKB	42.62	250000	160000	0.643856	250000
HNRNPUL1	95.68	1200000	770000	0.640104	1200000
IMMT	83.63	95000000	6E+07	0.639118	95000000
AFG3L2	88.53	28000000	2E+07	0.63743	28000000
FLOT1	47.33	5600000	4E+06	0.63743	5600000
NDUFS3	30.22	2800000	2E+06	0.63743	2800000
HDHD5	46.29	2800000	2E+06	0.63743	2800000
SQSTM1	47.66	7600000	5E+06	0.633218	7600000
PI4KA	236.68	990000	640000	0.629357	990000
TIMM44	51.32	17000000	1E+07	0.628031	17000000
TRIP11	227.45	3400000	2E+06	0.628031	3400000
MLEC	32.21	340000	220000	0.628031	340000
FIS1	16.93	540000	350000	0.625604	540000
UQCRFS1	29.65	4000000	3E+06	0.621488	4000000

MCAT	42.93	2000000	1E+06	0.621488	2000000
WDR1	66.15	2000000	1E+06	0.621488	2000000
PRKACA	40.56	600000	390000	0.621488	600000
PNKD	42.85	200000	130000	0.621488	200000
LRPPRC	157.81	6.3E+08	4E+08	0.619728	630000000
HK1	102.42	4600000	3E+06	0.616671	4600000
PA2G4	43.76	2300000	2E+06	0.616671	2300000
VCP	89.27	26000000	2E+07	0.612977	26000000
RPL3	46.08	13000000	9E+06	0.612977	13000000
TFB2M	45.32	2600000	2E+06	0.612977	2600000
CTTN	61.55	810000	530000	0.61193	810000
MRPL9	30.22	2900000	2E+06	0.610053	2900000
ADD1	80.9	290000	190000	0.610053	290000
COX7A2	9.39	3200000	2E+06	0.607683	3200000
PROCR	26.65	350000	230000	0.605721	350000
RPL35	14.54	3800000	3E+06	0.604071	3800000
TRIM32	71.94	100000	66000	0.599462	100000
MPC2	14.27	530000	350000	0.598637	530000
MYOF	234.56	56000000	4E+07	0.597902	56000000
PPT1	34.17	5900000	4E+06	0.597241	5900000
EXD2	70.31	650000	430000	0.596103	650000
SFXN3	35.48	8000000	5E+06	0.594008	8000000
CLTC	191.49	92000000	6E+07	0.592825	92000000
ECI2	43.56	1100000	730000	0.591535	1100000
RPL11	20.24	11000000	7E+06	0.591535	11000000
ACOT9	49.87	12000000	8E+06	0.584963	12000000
IGF2R	274.2	6000000	4E+06	0.584963	6000000
RPS4X	29.58	18000000	1E+07	0.584963	18000000
ACADM	46.56	12000000	8E+06	0.584963	12000000
COPB2	102.42	3600000	2E+06	0.584963	3600000
EIF3G	35.59	750000	500000	0.584963	750000
CISD2	15.27	1800000	1E+06	0.584963	1800000
HNRNPH1	49.2	3000000	2E+06	0.584963	3000000
RPL19	23.45	4500000	3E+06	0.584963	4500000
MYADM	35.25	1200000	800000	0.584963	1200000
GPI	63.11	540000	360000	0.584963	540000
MPP7	65.48	660000	440000	0.584963	660000
MRPS21	10.68	450000	300000	0.584963	450000
PSEN1	52.63	240000	160000	0.584963	240000
KRTCAP2	14.67	630000	420000	0.584963	630000
BACE2	56.14	480000	320000	0.584963	480000
HSPG2	468.53	150000	100000	0.584963	150000
FXR2	74.18	330000	220000	0.584963	330000
XAB2	99.95	90000	60000	0.584963	90000
ACAT1	45.17	13000000	9E+06	0.579424	13000000
TIMM22	20.02	730000	490000	0.575115	730000
AARS2	107.27	6100000	4E+06	0.573185	6100000
MAN2A1	131.06	5500000	4E+06	0.571906	5500000
PLBD2	65.43	1100000	740000	0.571906	1100000
NDUFS5	12.51	460000	310000	0.569366	460000
SLC25A10	31.26	4300000	3E+06	0.568284	4300000
RPS3	26.67	34000000	2E+07	0.563901	34000000
SLC29A1	50.19	1300000	880000	0.562936	1300000
MRPL20	17.43	3100000	2E+06	0.561879	3100000
OCIAD2	16.94	90000	61000	0.561116	90000
PRKCSH	59.39	14000000	1E+07	0.559427	14000000
ILF2	43.04	2800000	2E+06	0.559427	2800000
RAP1B	20.81	5600000	4E+06	0.559427	5600000
DHX15	90.88	250000	170000	0.556393	250000
PTGES	17.09	500000	340000	0.556393	500000
DARS2	73.52	9700000	7E+06	0.555519	9700000
COX5B	13.69	690000	470000	0.553936	690000
CANX	67.53	66000000	5E+07	0.552541	66000000
PABPC1	70.63	22000000	2E+07	0.552541	22000000
NCKAP1	128.71	220000	150000	0.552541	220000
ATL3	60.5	850000	580000	0.55141	850000
NT5DC2	60.68	1200000	820000	0.549339	1200000
ARF4	20.5	1900000	1E+06	0.547488	1900000

PLPP2	32.55	19000	13000	0.547488	19000
METTL7A	28.3	190000	130000	0.547488	190000
SCAMP4	25.71	1300000	890000	0.546634	1300000
RRBP1	152.36	16000000	1E+07	0.540568	16000000
DBN1	71.39	16000000	1E+07	0.540568	16000000
DNAJA3	52.46	3200000	2E+06	0.540568	3200000
MRPL44	37.51	1600000	1E+06	0.540568	1600000
EPHB4	108.2	1600000	1E+06	0.540568	1600000
TAMM41	51.03	3200000	2E+06	0.540568	3200000
SLC35A4	11.13	1600000	1E+06	0.540568	1600000
HTATIP2	27.03	1600000	1E+06	0.540568	1600000
PTDSS1	55.49	480000	330000	0.540568	480000
HACD2	28.35	610000	420000	0.53842	610000
RAB14	23.88	4500000	3E+06	0.537657	4500000
SF3B3	135.49	870000	600000	0.536053	870000
YME1L1	86.4	10000000	7E+06	0.535332	10000000
SLC3A2	67.95	4.2E+08	3E+08	0.534336	420000000
KPNB1	97.11	5500000	4E+06	0.533432	5500000
ESYT2	102.29	5500000	4E+06	0.533432	5500000
GFM2	86.55	550000	380000	0.533432	550000
LIMA1	85.17	52000000	4E+07	0.530515	52000000
VDAC3	30.64	52000000	4E+07	0.530515	52000000
MTHFD2	37.87	9100000	6E+06	0.530515	9100000
MRPL12	21.33	490000	340000	0.527247	490000
MACROH2A1	39.16	3600000	3E+06	0.526069	3600000
ATP1A1	112.82	92000000	6E+07	0.523562	92000000
SLC12A2	131.36	4600000	3E+06	0.523562	4600000
MRPS7	28.12	2300000	2E+06	0.523562	2300000
NPEPPS	103.21	230000	160000	0.523562	230000
GTPBP6	56.86	560000	390000	0.521953	560000
AIFM1	66.86	33000000	2E+07	0.520832	33000000
ESYT1	122.78	7600000	5E+06	0.520007	7600000
NDUFA11	14.84	430000	300000	0.519374	430000
ATP2A2	114.68	20000000	1E+07	0.514573	20000000
H2AC8	14.13	2E+08	1E+08	0.514573	200000000
RHOG	21.29	2000000	1E+06	0.514573	2000000
GNB1	37.35	2000000	1E+06	0.514573	2000000
IPO5	123.55	6700000	5E+06	0.5115	6700000
ATP2B1	134.6	5700000	4E+06	0.510962	5700000
ERAP1	107.17	370000	260000	0.509014	370000
SACM1L	66.92	2700000	2E+06	0.50696	2700000
ADCY9	150.6	270000	190000	0.50696	270000
HCCS	30.58	880000	620000	0.505235	880000
HDLBP	141.37	17000000	1E+07	0.5025	17000000
SLC25A13	74.13	51000000	4E+07	0.5025	51000000
PCBP1	37.47	1700000	1E+06	0.5025	1700000
CNNM3	76.07	170000	120000	0.5025	170000
RAB2A	23.53	8200000	6E+06	0.499571	8200000
RPS2	31.3	13000000	9E+06	0.498806	13000000
SEC22B	24.72	2400000	2E+06	0.4975	2400000
NDUFA13	16.69	1200000	850000	0.4975	1200000
ALDH18A1	87.25	1.1E+08	8E+07	0.495957	110000000
DPY19L1	77.27	90000	64000	0.491853	90000
EEF1D	31.1	450000	320000	0.491853	450000
SCAMP3	38.26	5900000	4E+06	0.490326	5900000
PDIA3	56.75	73000000	5E+07	0.489385	73000000
STOM	31.71	21000000	2E+07	0.485427	21000000
CD109	161.59	1400000	1E+06	0.485427	1400000
SDC1	32.44	6300000	5E+06	0.485427	6300000
SH3BP4	107.43	210000	150000	0.485427	210000
ATP5F1D	17.48	700000	500000	0.485427	700000
DHCR24	60.06	1400000	1E+06	0.485427	1400000
RPL13	24.25	13000000	9E+06	0.483209	13000000
GOLGA2	113.02	3900000	3E+06	0.478047	3900000
MARS1	101.05	3200000	2E+06	0.476438	3200000
APOO	22.27	3200000	2E+06	0.476438	3200000
DCTN1	139.01	64000	46000	0.476438	64000
NOMO1	134.24	8200000	6E+06	0.474909	8200000

YBX3	40.07	5000000	4E+06	0.473931	5000000
SLC38A1	54.01	6800000	5E+06	0.472753	6800000
RANBP6	124.63	18000	13000	0.469485	18000
CD276	57.2	180000	130000	0.469485	180000
GOLGA7	15.81	180000	130000	0.469485	180000
MISP	75.31	9400000	7E+06	0.467126	9400000
LAMA5	399.48	1200000	870000	0.463947	1200000
BDH1	38.13	120000	87000	0.463947	120000
RAVER1	77.8	120000	87000	0.463947	120000
ALG3	50.09	80000	58000	0.463947	80000
TFRC	84.82	2.2E+08	2E+08	0.459432	220000000
CLPX	69.18	22000000	2E+07	0.459432	22000000
LARS2	101.91	6600000	5E+06	0.459432	6600000
MOGS	91.86	2200000	2E+06	0.459432	2200000
CXADR	40	550000	400000	0.459432	550000
PUM1	126.39	110000	80000	0.459432	110000
YBX1	35.9	8100000	6E+06	0.457207	8100000
PDE12	67.31	3700000	3E+06	0.454566	3700000
VAMP2	12.65	2600000	2E+06	0.452512	2600000
TTK	97.01	260000	190000	0.452512	260000
POLRMT	138.53	41000000	3E+07	0.450661	41000000
PHB2	33.28	1.5E+08	1E+08	0.447459	150000000
ALDH7A1	58.45	15000000	1E+07	0.447459	15000000
SCFD1	72.33	1500000	1E+06	0.447459	1500000
ARMCX3	42.47	1500000	1E+06	0.447459	1500000
SDCBP	32.42	1500000	1E+06	0.447459	1500000
RPL10A	24.82	3000000	2E+06	0.447459	3000000
ABCD3	75.43	1500000	1E+06	0.447459	1500000
STEAP3	54.57	1500000	1E+06	0.447459	1500000
C1QBP	31.34	4500000	3E+06	0.447459	4500000
TMED9	27.26	4500000	3E+06	0.447459	4500000
GNB3	37.2	4900000	4E+06	0.444785	4900000
RPS13	17.21	6800000	5E+06	0.443607	6800000
HLA-B	40.43	3400000	3E+06	0.443607	3400000
LMNA	74.09	19000000	1E+07	0.440573	19000000
ATP6V1B2	56.46	1900000	1E+06	0.440573	1900000
NFS1	50.16	2300000	2E+06	0.436099	2300000
GSTK1	25.48	2300000	2E+06	0.436099	2300000
MRPL10	29.26	770000	570000	0.433897	770000
IARS2	113.72	27000000	2E+07	0.432959	27000000
BLMH	52.53	270000	200000	0.432959	270000
VAMP3	11.3	270000	200000	0.432959	270000
MRPL37	48.09	8500000	6E+06	0.432111	8500000
AGRN	217.18	120000	89000	0.431157	120000
GHITM	37.18	7400000	6E+06	0.428094	7400000
DARS1	57.1	4300000	3E+06	0.426265	4300000
ATPAF1	36.41	1100000	820000	0.423808	1100000
RPN2	69.24	16000000	1E+07	0.415037	16000000
STOML2	38.51	4800000	4E+06	0.415037	4800000
RPS9	22.58	16000000	1E+07	0.415037	16000000
ERP29	28.98	4000000	3E+06	0.415037	4000000
AAAS	59.54	2000000	2E+06	0.415037	2000000
MRPS30	50.33	600000	450000	0.415037	600000
GALNT7	75.34	1600000	1E+06	0.415037	1600000
PRAF2	19.25	720000	540000	0.415037	720000
MRPL27	16.06	1000000	750000	0.415037	1000000
HEATR1	242.22	650000	490000	0.407658	650000
NPC1	142.07	11000000	8E+06	0.40632	11000000
ATP6V0A2	98.02	900000	680000	0.40439	900000
PCK2	70.65	4100000	3E+06	0.403356	4100000
P3H3	81.79	820000	620000	0.403356	820000
OXSM	48.81	660000	500000	0.400538	660000
SDHA	72.65	58000000	4E+07	0.398549	58000000
SERPINH1	46.41	29000000	2E+07	0.398549	29000000
SLC7A2	71.63	2500000	2E+06	0.395929	2500000
MGST3	16.51	500000	380000	0.395929	500000
RP2	39.62	250000	190000	0.395929	250000
MRPL23	17.77	1300000	990000	0.393011	1300000

HSP90B1	92.41	2.1E+08	2E+08	0.392317	210000000
UFL1	89.54	2100000	2E+06	0.392317	2100000
RMDN3	52.09	2100000	2E+06	0.392317	2100000
ATP5MG	11.42	9300000	7E+06	0.389412	9300000
NNT	113.82	89000000	7E+07	0.388271	89000000
GOLPH3	33.79	170000	130000	0.387023	170000
ACO2	85.37	30000000	2E+07	0.383329	30000000
DLAT	68.95	8600000	7E+06	0.381871	8600000
HLA-H	40.87	4300000	3E+06	0.381871	4300000
DDOST	50.77	13000000	1E+07	0.378512	13000000
PGAM5	31.98	13000000	1E+07	0.378512	13000000
VDAC2	31.55	1.3E+08	1E+08	0.378512	130000000
ERBIN	158.2	1300000	1E+06	0.378512	1300000
FLVCR1	59.82	780000	600000	0.378512	780000
COQ6	50.84	130000	100000	0.378512	130000
NIFK	34.2	65000	50000	0.378512	65000
RALYL	32.31	260000	200000	0.378512	260000
MRPL53	12.1	1300000	1E+06	0.378512	1300000
EPB41L2	112.52	10000000	8E+06	0.37707	10000000
TTC17	129.48	74000	57000	0.376563	74000
RPS27	9.45	6100000	5E+06	0.376148	6100000
LETM1	83.3	35000000	3E+07	0.374396	35000000
ACADVL	70.35	22000000	2E+07	0.371969	22000000
UGGT1	177.08	2200000	2E+06	0.371969	2200000
SSRP1	81.02	2200000	2E+06	0.371969	2200000
IL13RA1	48.73	53000	41000	0.370368	53000
EMC1	111.69	6200000	5E+06	0.369234	6200000
PTGES2	41.92	4000000	3E+06	0.367732	4000000
MICU1	54.32	800000	620000	0.367732	800000
NUP160	162.02	1200000	930000	0.367732	1200000
TRABD	42.29	940000	730000	0.364764	940000
IARS1	144.41	5400000	4E+06	0.36257	5400000
HNRNPU	90.53	18000000	1E+07	0.36257	18000000
SUPV3L1	87.94	4500000	4E+06	0.36257	4500000
EIF4G2	102.3	1800000	1E+06	0.36257	1800000
STX7	29.8	1800000	1E+06	0.36257	1800000
TECR	36.01	2700000	2E+06	0.36257	2700000
ACLY	120.76	180000	140000	0.36257	180000
UQCC3	10.07	270000	210000	0.36257	270000
H4C16	11.36	7.7E+08	6E+08	0.359896	770000000
SEPTIN2	41.46	68000	53000	0.359542	68000
SLC2A3	53.89	500000	390000	0.358454	500000
HSPA9	73.63	7.3E+08	6E+08	0.356935	730000000
ARL1	20.4	320000	250000	0.356144	320000
G3BP1	52.13	11000000	9E+06	0.355095	11000000
TMEM59	36.2	1100000	860000	0.355095	1100000
ADAR	135.98	230000	180000	0.353637	230000
FUCA2	54.03	230000	180000	0.353637	230000
RPL13A	23.56	12000000	9E+06	0.352302	12000000
SEC11A	20.61	1200000	940000	0.352302	1200000
PRDX5	22.07	9300000	7E+06	0.349334	9300000
PBK	36.06	93000	73000	0.349334	93000
COPA	138.26	9800000	8E+06	0.347923	9800000
PC	129.55	14000000	1E+07	0.347923	14000000
PDHB	39.21	28000000	2E+07	0.347923	28000000
ME2	65.4	840000	660000	0.347923	840000
MRPL19	33.51	4200000	3E+06	0.347923	4200000
STON2	101.1	140000	110000	0.347923	140000
SUMO3	11.63	1400000	1E+06	0.347923	1400000
TM9SF4	74.47	140000	110000	0.347923	140000
PODXL	58.6	9500000	8E+06	0.341037	9500000
SPTLC1	52.71	1900000	2E+06	0.341037	1900000
MRPL49	19.19	1900000	2E+06	0.341037	1900000
HSP90AA1	84.61	1.1E+08	9E+07	0.338416	110000000
KCT2	29.22	1100000	870000	0.338416	1100000
POR	76.64	24000000	2E+07	0.337035	24000000
UQCRC2	48.41	24000000	2E+07	0.337035	24000000
HADH	34.27	24000000	2E+07	0.337035	24000000

ATP6V0A1	96.35	2400000	2E+06	0.337035	2400000
COX4I1	19.56	24000000	2E+07	0.337035	24000000
RPL7A	29.98	24000000	2E+07	0.337035	24000000
LAMP1	44.85	24000000	2E+07	0.337035	24000000
SLC38A2	55.99	12000000	1E+07	0.337035	12000000
TRNT1	50.1	340000	270000	0.332575	340000
CAP1	51.87	340000	270000	0.332575	340000
CLU	52.46	4400000	4E+06	0.330149	4400000
PMPCA	58.22	4900000	4E+06	0.329308	4900000
MDH2	35.48	2E+08	2E+08	0.321928	200000000
P4HA1	61.01	11000000	9E+06	0.321928	11000000
ECH1	35.79	15000000	1E+07	0.321928	15000000
GNAI2	40.43	3000000	2E+06	0.321928	3000000
RPS14	16.26	15000000	1E+07	0.321928	15000000
HEXA	60.66	2500000	2E+06	0.321928	2500000
RPS8	24.19	25000000	2E+07	0.321928	25000000
UBB	25.75	25000000	2E+07	0.321928	25000000
HMOX2	36.01	100000	80000	0.321928	100000
CD9	25.4	3000000	2E+06	0.321928	3000000
BAX	21.17	150000	120000	0.321928	150000
PEG10	80.12	360000	290000	0.311944	360000
ATP5F1A	59.71	5.7E+08	5E+08	0.309328	570000000
RPSA	32.83	26000000	2E+07	0.308122	26000000
DHODH	42.84	2600000	2E+06	0.308122	2600000
MRPL58	23.62	990000	800000	0.307429	990000
MRRF	29.26	1200000	970000	0.306978	1200000
CKAP4	65.98	21000000	2E+07	0.304855	21000000
ABCB6	93.83	3700000	3E+06	0.302563	3700000
GLS	73.41	8500000	7E+06	0.300866	8500000
MTCH2	33.31	16000000	1E+07	0.29956	16000000
DPM1	29.62	1600000	1E+06	0.29956	1600000
SSR3	21.07	800000	650000	0.29956	800000
EIF6	26.58	64000	52000	0.29956	64000
UNC45A	103.01	160000	130000	0.29956	160000
ZDHHC13	70.81	160000	130000	0.29956	160000
BSG	42.17	38000000	3E+07	0.293731	38000000
AUP1	45.76	380000	310000	0.293731	380000
SAMM50	51.94	12000000	1E+07	0.292181	12000000
SLC25A18	33.83	1200000	980000	0.292181	1200000
SLC16A3	49.44	7100000	6E+06	0.291766	7100000
KTN1	156.18	22000000	2E+07	0.289507	22000000
RPL24	17.77	5500000	5E+06	0.289507	5500000
Igh-6	49.94	500000	410000	0.286304	500000
RHOT2	68.07	3900000	3E+06	0.285402	3900000
COPG2	97.56	390000	320000	0.285402	390000
SLC25A12	74.71	34000000	3E+07	0.280108	34000000
UQCRC1	52.61	17000000	1E+07	0.280108	17000000
VAT1	41.89	1700000	1E+06	0.280108	1700000
RAB32	24.98	1700000	1E+06	0.280108	1700000
MTOR	288.71	170000	140000	0.280108	170000
GNAS	45.64	5700000	5E+06	0.278301	5700000
GGCX	87.5	570000	470000	0.278301	570000
ETFA	35.06	12000000	1E+07	0.277534	12000000
NDFIP1	24.88	400000	330000	0.277534	400000
PIK3C2A	190.56	460000	380000	0.275634	460000
ERGIC1	32.57	2300000	2E+06	0.275634	2300000
NADK2	49.4	8100000	7E+06	0.273761	8100000
ATP5PO	23.26	29000000	2E+07	0.273018	29000000
PLOD3	84.73	9900000	8E+06	0.271805	9900000
RPL27	15.79	3500000	3E+06	0.271302	3500000
ATP5ME	7.93	3500000	3E+06	0.271302	3500000
ACACB	276.37	350000	290000	0.271302	350000
SLC12A9	96.05	41000	34000	0.270089	41000
LGALS3BP	65.29	470000	390000	0.269187	470000
SPRYD4	23.11	470000	390000	0.269187	470000
HNRNPH3	36.9	100000	83000	0.268817	100000
PPIF	22.03	5300000	4E+06	0.268489	5300000
TUFM	49.84	1.2E+08	1E+08	0.263034	120000000

P4HB	57.08	7200000	6E+06	0.263034	7200000
RACK1	35.05	18000000	2E+07	0.263034	18000000
ALDH3A2	54.81	1200000	1E+06	0.263034	1200000
SLC25A4	33.04	42000000	4E+07	0.263034	42000000
ACP2	48.31	1200000	1E+06	0.263034	1200000
RAB8A	23.65	1800000	2E+06	0.263034	1800000
MRC2	166.57	120000	100000	0.263034	120000
NDUFV2	27.37	180000	150000	0.263034	180000
DDB1	126.89	550000	460000	0.257798	550000
FAM162A	17.33	3700000	3E+06	0.255257	3700000
TOMM70	67.41	31000000	3E+07	0.253757	31000000
EMC3	29.93	750000	630000	0.251539	750000
APLP2	86.9	500000	420000	0.251539	500000
GCAT	45.26	250000	210000	0.251539	250000
RPL21	18.55	2500000	2E+06	0.251539	2500000
RAB12	27.23	100000	84000	0.251539	100000
SLC25A5	32.83	8.2E+08	7E+08	0.249028	820000000
HACL1	63.69	320000	270000	0.245112	320000
GDI2	50.63	110000	93000	0.242201	110000
TMED10	24.96	1300000	1E+06	0.241008	1300000
GSR	56.22	1300000	1E+06	0.241008	1300000
TMCO1	27.06	1300000	1E+06	0.241008	1300000
SYNCRIP	69.56	6000000	5E+06	0.234465	6000000
CYB5R3	34.21	10000000	9E+06	0.234465	10000000
ABCB10	79.1	1000000	850000	0.234465	1000000
SPR	28.03	200000	170000	0.234465	200000
BUD23	31.86	94000	80000	0.232661	94000
DLD	54.14	27000000	2E+07	0.231326	27000000
CCDC134	26.54	81000	69000	0.231326	81000
ATP5MK	6.45	2700000	2E+06	0.231326	2700000
NDUFA4	9.36	2700000	2E+06	0.231326	2700000
ALDH1L1	98.77	340000	290000	0.229482	340000
HNRNPA2B1	37.41	11000000	9E+06	0.226771	11000000
TPP1	61.21	110000	94000	0.226771	110000
SHMT2	55.96	62000000	5E+07	0.226276	62000000
NOA1	78.41	7600000	7E+06	0.22556	7600000
AP2A1	107.48	8300000	7E+06	0.225292	8300000
GLUD1	61.36	42000000	4E+07	0.222392	42000000
MTHFD1L	105.72	42000000	4E+07	0.222392	42000000
ACAA2	41.9	14000000	1E+07	0.222392	14000000
KARS1	68	2100000	2E+06	0.222392	2100000
AK2	26.46	2100000	2E+06	0.222392	2100000
NCLN	62.93	2100000	2E+06	0.222392	2100000
RPS17	15.54	1400000	1E+06	0.222392	1400000
CAPZA2	32.93	1400000	1E+06	0.222392	1400000
ARMCX2	65.64	640000	550000	0.21864	640000
PRDX4	30.52	5700000	5E+06	0.21818	5700000
NDUFV1	50.78	4300000	4E+06	0.216811	4300000
NDUFS2	52.51	7900000	7E+06	0.216318	7900000
LARS1	134.38	7200000	6E+06	0.215729	7200000
SSR1	32.22	2900000	3E+06	0.214125	2900000
NIF3L1	41.94	290000	250000	0.214125	290000
HYOU1	111.27	22000000	2E+07	0.211504	22000000
EMC8	23.76	220000	190000	0.211504	220000
SLC25A24	53.32	9600000	8E+06	0.209923	9600000
GTF2I	112.35	15000000	1E+07	0.206451	15000000
CLPP	30.16	1500000	1E+06	0.206451	1500000
P3H1	83.34	610000	530000	0.202817	610000
NDUFA9	42.48	9200000	8E+06	0.201634	9200000
HSD17B12	34.3	2300000	2E+06	0.201634	2300000
FECH	47.83	470000	410000	0.197037	470000
EEA1	162.37	63000000	6E+07	0.19592	63000000
OXCT1	56.12	63000000	6E+07	0.19592	63000000
LONP1	93.24	64000000	6E+07	0.192645	64000000
ACAD9	68.72	16000000	1E+07	0.192645	16000000
SPECC1	118.51	240000	210000	0.192645	240000
RALA	23.55	1600000	1E+06	0.192645	1600000
PLSCR1	35.03	16000	14000	0.192645	16000

CTNNA2	105.25	4100000	4E+06	0.187627	4100000
PITRM1	117.34	3300000	3E+06	0.186413	3300000
CIT	231.29	990000	870000	0.186413	990000
LAP3	56.13	5800000	5E+06	0.185556	5800000
DNAJC13	254.25	2500000	2E+06	0.184425	2500000
ACOT2	53.18	2500000	2E+06	0.184425	2500000
NDUFB11	17.31	2500000	2E+06	0.184425	2500000
B4GALT1	43.89	840000	740000	0.182864	840000
TRAP1	80.06	1.7E+08	2E+08	0.180572	170000000
HSD17B10	26.91	34000000	3E+07	0.180572	34000000
NUP155	155.1	1700000	2E+06	0.180572	1700000
MYO19	109.07	350000	310000	0.175087	350000
HSPA5	72.29	1.8E+08	2E+08	0.169925	180000000
GPAM	93.74	180000	160000	0.169925	180000
MRPL47	29.43	720000	640000	0.169925	720000
HSPE1	10.92	1.1E+08	1E+08	0.16665	110000000
MICOS13	13.08	840000	750000	0.163499	840000
EPRS1	170.48	8600000	8E+06	0.159478	8600000
GANAB	106.81	77000000	7E+07	0.158262	77000000
HSPD1	61.02	3.9E+09	4E+09	0.156119	3900000000
GLB1	76.03	4900000	4E+06	0.155278	4900000
MCCC2	61.29	6900000	6E+06	0.154328	6900000
ATAD3B	72.53	79000000	7E+07	0.154034	79000000
PPIB	23.73	40000000	4E+07	0.152003	40000000
PDHA1	43.27	40000000	4E+07	0.152003	40000000
LACTB	60.65	1000000	900000	0.152003	1000000
ALDH1B1	57.21	7100000	6E+06	0.149747	7100000
ARL6IP5	21.6	710000	640000	0.149747	710000
AP2M1	49.62	9200000	8E+06	0.148523	9200000
GFM1	83.42	21000000	2E+07	0.14439	21000000
IDH2	50.88	850000	770000	0.142604	850000
CS	51.68	98000000	9E+07	0.138976	98000000
HSPA8	70.85	1.1E+08	1E+08	0.137504	110000000
MMUT	83.08	6600000	6E+06	0.137504	6600000
AP2A2	103.89	2200000	2E+06	0.137504	2200000
MRPS2	33.23	2200000	2E+06	0.137504	2200000
ATP6V0D1	40.3	2200000	2E+06	0.137504	2200000
POGLUT2	58.01	33000	30000	0.137504	33000
MT-ND4	51.55	110000	100000	0.137504	110000
CHCHD2	15.5	1000000	910000	0.136062	1000000
IKBIP	39.28	4500000	4E+06	0.134301	4500000
BRI3BP	27.82	68000	62000	0.133267	68000
USP39	65.34	68000	62000	0.133267	68000
VWA8	214.69	2300000	2E+06	0.131245	2300000
FAM210A	30.76	230000	210000	0.131245	230000
PLD3	54.67	4800000	4E+06	0.125531	4800000
SLC38A5	51.42	960000	880000	0.125531	960000
ATP6V1H	55.85	120000	110000	0.125531	120000
MRPL54	15.81	720000	660000	0.125531	720000
LYRM4	10.75	850000	780000	0.123989	850000
GNAI1	40.34	1000000	920000	0.120294	1000000
ZC3HAV1	101.37	13000000	1E+07	0.115477	13000000
STXBP5	127.49	130000	120000	0.115477	130000
MICU2	49.63	670000	620000	0.111893	670000
ATAD3A	71.32	27000000	3E+07	0.111031	27000000
RAB11A	24.38	5400000	5E+06	0.111031	5400000
NDUFS1	79.42	14000000	1E+07	0.106915	14000000
INF2	135.54	840000	780000	0.106915	840000
SUCLG1	36.23	4200000	4E+06	0.106915	4200000
ETFDH	68.45	1400000	1E+06	0.106915	1400000
TMEM65	25.48	280000	260000	0.106915	280000
IER3IP1	8.96	280000	260000	0.106915	280000
NAGLU	82.21	290000	270000	0.103093	290000
ABCD1	82.88	1500000	1E+06	0.099536	1500000
RAB18	22.96	1500000	1E+06	0.099536	1500000
CYB5B	16.68	1500000	1E+06	0.099536	1500000
COQ3	41.03	150000	140000	0.099536	150000
VPS35	91.65	3100000	3E+06	0.096215	3100000

NIPSNAP1	33.29	1600000	2E+06	0.093109	1600000
NDUFB3	11.39	1600000	2E+06	0.093109	1600000
TRA2B	33.65	320000	300000	0.093109	320000
ABCB7	82.59	3300000	3E+06	0.090198	3300000
TFB1M	39.52	1700000	2E+06	0.087463	1700000
ZC3H15	48.57	1700000	2E+06	0.087463	1700000
ILF3	95.28	3900000	4E+06	0.075949	3900000
MCAM	71.56	7900000	8E+06	0.074962	7900000
HADHB	51.26	20000000	2E+07	0.074001	20000000
VDAC1	30.75	2E+08	2E+08	0.074001	200000000
CLPTM1	76.05	2000000	2E+06	0.074001	2000000
EIF2S1	36.09	2000000	2E+06	0.074001	2000000
MAN1B1	79.53	2100000	2E+06	0.070389	2100000
PRKAR2A	45.49	430000	410000	0.068713	430000
RBM39	59.34	9000000	9E+06	0.065588	9000000
HMGCL	34.34	230000	220000	0.06413	230000
GLS	18.62	980000	940000	0.060121	980000
ALG1	52.48	49000	47000	0.060121	49000
GRSF1	53.09	1000000	960000	0.058894	1000000
COQ8B	60.03	260000	250000	0.056584	260000
ELAC2	92.16	2700000	3E+06	0.054448	2700000
ATP5F1C	32.98	27000000	3E+07	0.054448	27000000
ERLIN2	37.82	2700000	3E+06	0.054448	2700000
CD63	25.62	28000000	3E+07	0.052467	28000000
TMX1	31.77	2800000	3E+06	0.052467	2800000
MRPS27	47.58	5700000	6E+06	0.05153	5700000
SLC25A6	32.85	29000000	3E+07	0.050626	29000000
FH	54.6	32000000	3E+07	0.045804	32000000
STX12	31.62	360000	350000	0.040642	360000
CHCHD3	26.14	7500000	7E+06	0.038994	7500000
CALR	48.11	48000000	5E+07	0.030374	48000000
FKBP8	44.53	5400000	5E+06	0.026967	5400000
CYC1	35.4	6400000	6E+06	0.02272	6400000
DAP3	45.54	7200000	7E+06	0.020178	7200000
NME6	21.13	90000	89000	0.01612	90000
GOT2	47.49	91000000	9E+07	0.015942	91000000
SLC25A3	40.07	2.3E+08	2E+08	0	230000000
PRORP	67.27	2200000	2E+06	0	2200000
LRRC59	34.91	26000000	3E+07	0	26000000
SEC63	87.94	2800000	3E+06	0	2800000
MRPL1	36.89	3300000	3E+06	0	3300000
Ighg1	43.36	1.2E+08	1E+08	0	120000000
RPS6	28.66	15000000	2E+07	0	15000000
SRP72	74.56	1700000	2E+06	0	1700000
CCDC51	45.78	2800000	3E+06	0	2800000
RDH11	35.36	3300000	3E+06	0	3300000
FASTKD3	75.64	370000	370000	0	370000
MACROD1	35.48	2500000	3E+06	0	2500000
RPL35A	12.53	1100000	1E+06	0	1100000
MRPL21	22.8	1300000	1E+06	0	1300000
SCO2	29.79	270000	270000	0	270000
RER1	22.94	1300000	1E+06	0	1300000
RAB6A	23.58	2200000	2E+06	0	2200000
TIMM17A	18.01	140000	140000	0	140000
OXNAD1	34.83	250000	250000	0	250000
CHDH	65.32	160000	160000	0	160000
TMEM70	28.95	320000	320000	0	320000
BST2	19.76	2600000	3E+06	0	2600000
EVA1B	18.36	22000	22000	0	22000
TNPO1	102.29	230000	230000	0	230000
RPS29	6.67	550000	550000	0	550000
SEC61B	9.97	2600000	3E+06	0	2600000
FAM3C	24.66	160000	160000	0	160000
EXOG	41.06	240000	240000	0	240000
PIGS	61.62	100000	100000	0	100000
SDF4	41.78	260000	260000	0	260000
EDIL3	53.73	990000	1E+06	-0.0145	1000000
HTRA2	48.81	760000	770000	-0.01886	770000

GBA1	59.68	630000	640000	-0.02272	640000
DNAJA1	44.84	6300000	6E+06	-0.02272	6400000
RPS23	15.8	6200000	6E+06	-0.02308	6300000
TSFM	35.37	5900000	6E+06	-0.02425	6000000
RAB5C	23.47	5500000	6E+06	-0.026	5600000
BAK1	23.39	540000	550000	-0.02647	550000
RHOT1	70.74	960000	980000	-0.02975	980000
COL7A1	295.04	4600000	5E+06	-0.03103	4700000
TNPO2	101.32	450000	460000	-0.03171	460000
DLST	48.72	8200000	8E+06	-0.03477	8400000
ACSL1	77.89	360000	370000	-0.03953	370000
XPNPEP3	57	630000	650000	-0.04509	650000
SLC25A11	34.04	9200000	1E+07	-0.04629	9500000
CPS1	164.83	2.6E+09	3E+09	-0.05445	2700000000
SRSF7	27.35	510000	530000	-0.0555	530000
MRPS35	36.82	2200000	2E+06	-0.06413	2300000
P4HA2	60.86	2200000	2E+06	-0.06413	2300000
ATP1B3	31.49	4200000	4E+06	-0.06711	4400000
H1-4	21.85	19000000	2E+07	-0.074	20000000
SARS2	58.25	3600000	4E+06	-0.078	3800000
BCKDK	46.33	900000	950000	-0.078	950000
NEGR1	38.69	720000	760000	-0.078	760000
RPLP2	11.66	350000	370000	-0.08017	370000
HIP1	116.15	1700000	2E+06	-0.08246	1800000
SDR39U1	31.06	84000	89000	-0.08342	89000
CDS2	51.38	160000	170000	-0.08746	170000
GOLIM4	81.83	4300000	5E+06	-0.0973	4600000
TRUB2	36.67	140000	150000	-0.09954	150000
LARP4	80.55	670000	720000	-0.10384	720000
ACSL4	79.14	13000000	1E+07	-0.10692	14000000
SCAP	139.64	13000	14000	-0.10692	14000
AGPS	72.87	3800000	4E+06	-0.10962	4100000
PCCB	58.18	1200000	1E+06	-0.11548	1300000
PGK1	44.59	360000	390000	-0.11548	390000
CAD	242.83	1.1E+08	1E+08	-0.12553	120000000
FAM114A2	55.43	110000	120000	-0.12553	120000
MTHFD1	101.47	430000	470000	-0.12832	470000
MRPL4	34.9	4200000	5E+06	-0.13124	4600000
SRPRB	29.68	3100000	3E+06	-0.13327	3400000
ATP5F1B	56.52	4.1E+08	5E+08	-0.1343	450000000
LMAN2	40.2	1000000	1E+06	-0.1375	1100000
ERP44	46.94	1000000	1E+06	-0.1375	1100000
BCKDHB	43.09	10000	11000	-0.1375	11000
FCHO2	88.87	100000	110000	-0.1375	110000
LYZ	16.53	500000	550000	-0.1375	550000
LETMD1	41.76	390000	430000	-0.14086	430000
MRPL43	23.42	2700000	3E+06	-0.152	3000000
MYDGF	18.78	900000	1E+06	-0.152	1000000
BZW1	48.01	420000	470000	-0.16227	470000
FAM98A	55.24	330000	370000	-0.16506	370000
PDZD8	128.48	33000	37000	-0.16506	37000
CPVL	54.13	570000	640000	-0.16711	640000
AGK	36.87	2400000	3E+06	-0.16993	2700000
DAB2	82.4	2400000	3E+06	-0.16993	2700000
MTFP1	18	480000	540000	-0.16993	540000
MACF1	837.79	310000	350000	-0.17509	350000
RBM14	69.45	230000	260000	-0.17688	260000
TBRG4	70.69	8800000	1E+07	-0.18442	10000000
CYCS	11.74	22000000	3E+07	-0.18442	25000000
MRPL38	44.57	2200000	3E+06	-0.18442	2500000
SLC25A36	34.26	440000	500000	-0.18442	500000
GOLGB1	375.79	4200000	5E+06	-0.19265	4800000
CLPTM1L	62.19	1400000	2E+06	-0.19265	1600000
MAP7	84	1400000	2E+06	-0.19265	1600000
H2AZ1	13.54	3300000	4E+06	-0.20353	3800000
SOD2	24.73	1900000	2E+06	-0.2115	2200000
RAB3D	24.25	19000000	2E+07	-0.2115	22000000
MRPL2	33.28	630000	730000	-0.21254	730000

MRPL48	23.92	810000	940000	-0.21474	940000
PHB1	29.79	1.2E+08	1E+08	-0.22239	140000000
PMPCB	54.33	12000000	1E+07	-0.22239	14000000
ECE1	87.11	1200000	1E+06	-0.22239	1400000
COX7A2L	12.61	480000	560000	-0.22239	560000
SCAMP1	37.9	780000	910000	-0.22239	910000
NUP210	204.98	3400000	4E+06	-0.23447	4000000
MRM1	38.61	170000	200000	-0.23447	200000
LAMTOR3	13.61	280000	330000	-0.23704	330000
SUCLA2	50.29	2200000	3E+06	-0.24101	2600000
TMEM43	44.85	110000	130000	-0.24101	130000
SLC49A4	52.05	440000	520000	-0.24101	520000
SEC61A1	52.23	2700000	3E+06	-0.24511	3200000
GRPEL1	24.26	2100000	3E+06	-0.25154	2500000
EARS2	58.65	2100000	3E+06	-0.25154	2500000
COL12A1	332.94	2600000	3E+06	-0.25376	3100000
ECSIT	49.12	660000	790000	-0.25939	790000
NDUFA12	17.1	760000	910000	-0.25987	910000
HSD17B4	79.64	10000000	1E+07	-0.26303	12000000
ERAL1	48.32	2500000	3E+06	-0.26303	3000000
MRPL17	20.04	1000000	1E+06	-0.26303	1200000
SEPTIN6	49.69	250000	300000	-0.26303	300000
CCPG1	87.29	440000	530000	-0.26849	530000
PRSS3	32.51	830000	1E+06	-0.26882	1000000
MRPS9	45.81	2400000	3E+06	-0.27302	2900000
RAB7A	23.47	33000000	4E+07	-0.27753	40000000
GALNT2	64.69	14000000	2E+07	-0.28011	17000000
FASTKD2	81.41	8200000	1E+07	-0.2863	10000000
COL18A1	178.08	820000	1E+06	-0.2863	1000000
COPG1	97.66	2700000	3E+06	-0.28951	3300000
SCARB1	60.84	5800000	7E+06	-0.29177	7100000
MANBA	100.83	390000	480000	-0.29956	480000
CASK	105.06	300000	370000	-0.30256	370000
LSS	83.26	1700000	2E+06	-0.30485	2100000
CPT1A	88.31	9700000	1E+07	-0.30698	12000000
CPA4	47.32	620000	770000	-0.31259	770000
FLOT2	47.03	3700000	5E+06	-0.31411	4600000
HADHA	82.95	44000000	6E+07	-0.32193	55000000
ACADSB	47.46	1200000	2E+06	-0.32193	1500000
SRP68	70.69	550000	690000	-0.32716	690000
PKP3	87.03	190000	240000	-0.33703	240000
PABPC4	70.74	2900000	4E+06	-0.35147	3700000
ELAVL1	36.07	1800000	2E+06	-0.35364	2300000
BCAR1	93.31	530000	680000	-0.35954	680000
COL5A1	183.45	850000	1E+06	-0.37197	1100000
PPA2	37.9	5400000	7E+06	-0.3744	7000000
MRPS23	21.76	1300000	2E+06	-0.38702	1700000
WDFY1	46.29	260000	340000	-0.38702	340000
SFXN1	35.6	16000000	2E+07	-0.39232	21000000
RPS7	22.11	1600000	2E+06	-0.39232	2100000
SEC24C	118.25	990000	1E+06	-0.39301	1300000
MTARC1	37.48	51000	67000	-0.39366	67000
RAB29	23.14	510000	670000	-0.39366	670000
MRPS28	20.83	760000	1E+06	-0.39593	1000000
VPS26A	38.15	220000	290000	-0.39855	290000
VRK2	58.1	46000	61000	-0.40718	61000
CAT	59.72	3200000	4E+06	-0.42626	4300000
IDE	117.89	170000	230000	-0.4361	230000
MIPEP	80.59	1400000	2E+06	-0.44057	1900000
LAMP2	44.93	1600000	2E+06	-0.45943	2200000
CLPB	78.68	8700000	1E+07	-0.46395	12000000
DAGLB	73.68	210000	290000	-0.46566	290000
SCPEP1	50.8	600000	830000	-0.46815	830000
CTSZ	33.85	1300000	2E+06	-0.46949	1800000
CARS2	62.18	4100000	6E+06	-0.47534	5700000
ETFB	27.83	6600000	9E+06	-0.47917	9200000
PYCR2	33.62	1000000	1E+06	-0.48543	1400000
ECI1	32.8	1400000	2E+06	-0.51457	2000000

MRPS25	20.1	770000	1E+06	-0.51457	1100000
NSF	82.54	5100000	7E+06	-0.5174	7300000
NARS2	54.06	440000	630000	-0.51785	630000
AK3	25.55	480000	690000	-0.52356	690000
GARS1	83.11	1800000	3E+06	-0.53051	2600000
NDC1	76.26	510000	740000	-0.53703	740000
SCAMP2	36.63	220000	320000	-0.54057	320000
ERLIN1	39.15	1300000	2E+06	-0.54749	1900000
PRCP	55.76	1700000	3E+06	-0.55639	2500000
LAMTOR1	17.73	950000	1E+06	-0.55943	1400000
RPL29	17.74	4200000	6E+06	-0.56188	6200000
CAPRIN1	78.32	880000	1E+06	-0.56294	1300000
SRPRA	69.77	580000	860000	-0.56828	860000
RPUSD3	38.44	290000	430000	-0.56828	430000
GNS	62.04	12000000	2E+07	-0.58496	18000000
MUC13	54.57	540000	810000	-0.58496	810000
H2AX	15.14	32000	48000	-0.58496	48000
MARCKS	31.54	8600000	1E+07	-0.5961	13000000
MRPL39	38.69	1900000	3E+06	-0.61005	2900000
DNAJC10	91.02	720000	1E+06	-0.61143	1100000
MTDH	63.8	3400000	5E+06	-0.61298	5200000
MT-ATP6	24.8	3400000	5E+06	-0.61298	5200000
FOXRED1	53.78	1500000	2E+06	-0.61667	2300000
MRPL40	24.48	150000	230000	-0.61667	230000
CDK5RAP3	56.89	650000	1E+06	-0.62149	1000000
RHBDD2	39.18	130000	200000	-0.62149	200000
FARSA	57.53	240000	370000	-0.62449	370000
HNRNPAB	36.2	220000	340000	-0.62803	340000
RAP2A	20.6	20000	31000	-0.63227	31000
SYPL1	28.55	1800000	3E+06	-0.63743	2800000
SCCPDH	47.12	7700000	1E+07	-0.6401	12000000
SCARB2	54.26	2500000	4E+06	-0.64155	3900000
TMEM109	26.19	1600000	3E+06	-0.64386	2500000
TMEM14C	11.56	12000	19000	-0.66297	19000
TEFM	41.65	32000	51000	-0.67243	51000
PCYOX1	56.6	1000000	2E+06	-0.67807	1600000
MRPL28	30.14	1000000	2E+06	-0.67807	1600000
LAMB1	197.91	4700000	8E+06	-0.69334	7600000
POLG	139.47	430000	700000	-0.70302	700000
MGAT1	50.85	520000	850000	-0.70895	850000
ALDH6A1	57.8	550000	900000	-0.71049	900000
ADGRG1	77.69	140000	230000	-0.71621	230000
SLC16A1	53.91	5100000	8E+06	-0.71989	8400000
MFN1	84.11	60000	99000	-0.72247	99000
ARL8A	21.4	960000	2E+06	-0.73697	1600000
HARS1	57.37	1300000	2E+06	-0.75899	2200000
ARFGAP1	44.64	1400000	2E+06	-0.77761	2400000
SYNGR2	24.79	570000	980000	-0.78182	980000
DECR1	36.04	3300000	6E+06	-0.7885	5700000
MARS2	66.55	98000	170000	-0.79468	170000
RPS20	13.36	3500000	6E+06	-0.80145	6100000
DDRGK1	35.59	2100000	4E+06	-0.81714	3700000
L2HGDH	50.28	880000	2E+06	-0.8625	1600000
SLC7A1	67.59	220000	400000	-0.8625	400000
	92.33	340000	620000	-0.86673	620000
ALDH2	56.35	6300000	1E+07	-0.92961	12000000
DAD1	12.49	1400000	3E+06	-0.94753	2700000
MRPS22	41.25	6200000	1E+07	-0.95269	12000000
TARS2	80.99	620000	1E+06	-0.95269	1200000
GLA	48.73	490000	980000	-1	980000
NT5C3A	37.92	700000	1E+06	-1	1400000
ATP6V1A	68.26	740000	2E+06	-1.01937	1500000
GADD45GIP1	25.37	5900000	1E+07	-1.02425	12000000
EPHA7	112.02	280000	570000	-1.02554	570000
TMEM192	30.9	4900000	1E+07	-1.02915	10000000
LAMTOR2	13.5	230000	470000	-1.03103	470000
FASTKD1	97.35	400000	820000	-1.03562	820000
IGF2BP3	63.67	780000	2E+06	-1.03653	1600000

OSBPL9	83.13	190000	390000	-1.03747	390000
TBL2	49.77	1800000	4E+06	-1.03953	3700000
VAPA	27.88	1600000	3E+06	-1.04439	3300000
NDUFA5	13.45	300000	620000	-1.04731	620000
EMC2	34.81	530000	1E+06	-1.05344	1100000
RARS2	65.46	720000	2E+06	-1.05889	1500000
RAB1B	22.16	90000	190000	-1.078	190000
CPT2	73.73	2400000	5E+06	-1.08746	5100000
ECHDC1	33.68	190000	410000	-1.10962	410000
LMAN1	57.51	1800000	4E+06	-1.11548	3900000
TRMT10C	47.32	1500000	3E+06	-1.1375	3300000
FAM120A	121.81	370000	830000	-1.16559	830000
MRPL3	38.61	620000	1E+06	-1.17509	1400000
CDKAL1	65.07	110000	250000	-1.18442	250000
SLC25A17	34.54	48000	110000	-1.1964	110000
VPS13C	422.12	77000	180000	-1.22507	180000
RDH13	35.91	320000	750000	-1.22882	750000
TOP1	90.67	2600000	6E+06	-1.2303	6100000
HARS2	56.85	1700000	4E+06	-1.23447	4000000
ATP6AP1	51.99	460000	1E+06	-1.2578	1100000
TPRN	75.51	92000	220000	-1.2578	220000
CPD	152.84	500000	1E+06	-1.26303	1200000
ITM2C	30.2	180000	440000	-1.28951	440000
HSDL2	45.37	1300000	3E+06	-1.29956	3200000
PDIA4	72.89	16000000	4E+07	-1.32193	40000000
GTPBP10	42.91	760000	2E+06	-1.32193	1900000
NAGA	46.53	220000	550000	-1.32193	550000
OSBPL8	101.13	1700000	4E+06	-1.3388	4300000
PGRMC1	21.66	320000	810000	-1.33985	810000
MTPAP	66.13	3100000	8E+06	-1.34958	7900000
EIF3F	37.54	450000	1E+06	-1.41504	1200000
MTERF3	47.94	260000	710000	-1.44931	710000
RAI14	109.97	140000	410000	-1.5502	410000
TMEM263	11.74	220000	650000	-1.56294	650000
MFSD10	48.31	6400	19000	-1.56986	19000
SLC25A1	33.99	5000000	2E+07	-1.58496	15000000
DUS2	55.02	60000	180000	-1.58496	180000
AKR7A2	39.56	79000	240000	-1.60311	240000
TMEM106C	27.86	75000	230000	-1.61667	230000
BAG6	119.33	640000	2E+06	-1.64386	2000000
ABHD10	33.91	380000	1E+06	-1.65896	1200000
H2BC12	13.88	39000000	1E+08	-1.73697	130000000
NAXE	31.65	72000	240000	-1.73697	240000
PRKCI	68.22	96000	340000	-1.82443	340000
TXNDC5	47.6	360000	1E+06	-1.95936	1400000
SLC6A15	81.78	130000	510000	-1.97199	510000
CNP	47.55	3300000	1E+07	-1.97797	13000000
TARDBP	44.71	230000	910000	-1.98423	910000
ITPR1	313.73	140000	570000	-2.02554	570000
PARD3	151.33	110000	460000	-2.06413	460000
KIDINS220	196.42	70000	300000	-2.09954	300000
GAA	105.26	600000	3E+06	-2.11548	2600000
MTIF2	81.27	1300000	6E+06	-2.15754	5800000
NCSTN	78.36	2300000	1E+07	-2.2578	11000000
ACAA1	44.26	120000	590000	-2.29768	590000
OCIAD1	27.61	1200000	6E+06	-2.29768	5900000
MRPL14	15.94	2500000	1E+07	-2.37851	13000000
G3BP2	54.09	250000	1E+06	-2.37851	1300000
TGFB1	44.3	25000	130000	-2.37851	130000
GTPBP8	32.13	21000	120000	-2.51457	120000
GGH	35.94	120000	700000	-2.54432	700000
MTO1	79.91	55000	330000	-2.58496	330000
SNX27	61.23	110000	690000	-2.64909	690000
CRYZ	35.18	1200000	8E+06	-2.68182	7700000
TMEM126A	21.51	42000	270000	-2.6845	270000
SPTBN5	416.49	28000	200000	-2.8365	200000
PARL	42.16	84000	670000	-2.9957	670000
DNM1L	81.83	17000	140000	-3.04182	140000

WARS2	40.12	42000	350000	-3.05889	350000
MFN2	86.35	690000	6E+06	-3.12029	6000000
PYCR1	33.34	100000	910000	-3.18587	910000
SPART	72.79	1200000	1E+07	-3.1964	11000000
MRPS5	47.98	50000	470000	-3.23266	470000
PTPMT1	22.83	68000	700000	-3.36375	700000
SNRPD3	13.91	69000	1E+06	-3.85726	1000000
YWHAG	28.28	75000	2E+06	-4.87447	2200000
TMEM237	45.5	590000	4E+07	-6.18748	43000000
TOMM20	16.29	17000	4E+06	-7.72632	3600000
